# Ligand-controlled insertion regioselectivity accelerates copolymerisation of ethylene with methyl acrylate by cationic bisphosphine monoxide–palladium catalysts†
†Electronic supplementary information (ESI) available: Experimental procedure, NMR spectra of complexes and (co)polymers, and X-ray crystallographic data. CCDC (**3c**: 1408864, **4a**: 1408865**5a-py**: 1408866, **5c-di**: 1408867**5d-py**: 1408868). For ESI and crystallographic data in CIF or other electronic format see DOI: 10.1039/c5sc03361f


**DOI:** 10.1039/c5sc03361f

**Published:** 2015-11-03

**Authors:** Yusuke Mitsushige, Brad P. Carrow, Shingo Ito, Kyoko Nozaki

**Affiliations:** a Department of Chemistry and Biotechnology , Graduate School of Engineering , The University of Tokyo , 7-3-1 Hongo , Bunkyo-ku , Tokyo 113-8656 , Japan . Email: nozaki@chembio.t.u-tokyo.ac.jp; b Department of Chemistry , Princeton University , Princeton , New Jersey , USA

## Abstract

A new series of palladium catalysts ligated by a chelating bisphosphine monoxide bearing diarylphosphino groups (aryl-BPMO) exhibits markedly higher reactivity for ethylene/methyl acrylate copolymerisation when compared to the first generation of alkyl-BPMO–palladium catalysts that contain a dialkylphosphino moiety. Mechanistic studies suggest that the origin of this disparate catalyst behavior is a change in regioselectivity of migratory insertion of the acrylate comonomer as a function of the phosphine substituents. The best aryl-BPMO–palladium catalysts for these copolymerisations were shown to undergo exclusively 2,1-insertion, and this high regioselectivity avoids formation of a poorly reactive palladacycle intermediate. Furthermore, the aryl-BPMO–palladium catalysts can copolymerise ethylene with other industrially important polar monomers.

## Abstract 2

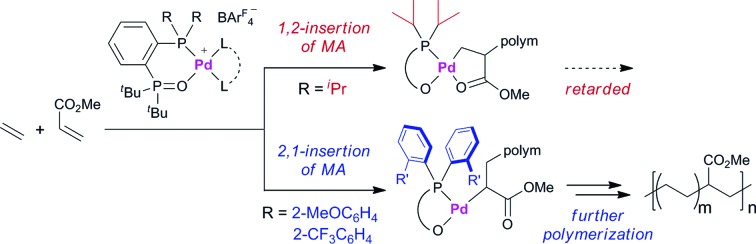
A new series of palladium catalysts ligated by a chelating bisphosphine monoxide bearing diarylphosphino groups (aryl-BPMO) exhibits markedly higher reactivity for ethylene/methyl acrylate copolymerisation.

## Introduction

**Scheme 1:**
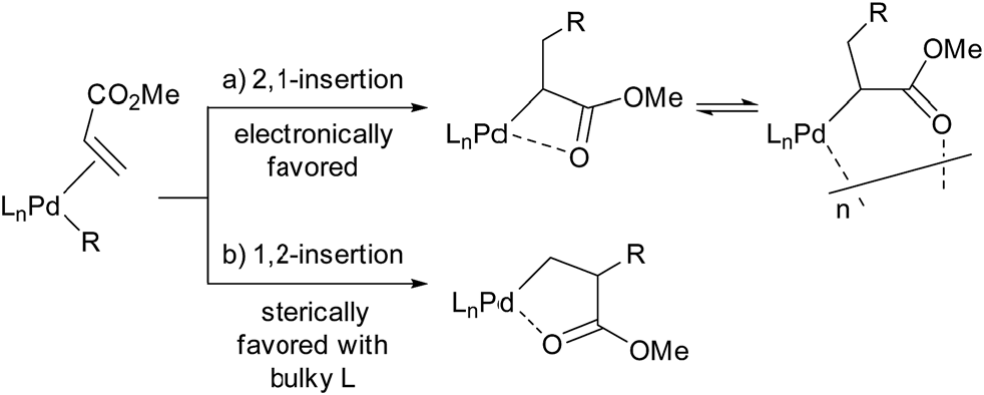
Schematic view of 2,1-insertion and 1,2-insertion of acrylates.

Migratory insertion of alkenes is a fundamental organometallic reaction involved in a number of industrial processes such as hydroformylation, hydrocyanation, hydrogenation, the Heck reaction in fine chemical synthesis, and olefin polymerisation. The regioselectivity of migratory insertion affects isomer distributions and thus product yields of catalytic processes that generate small molecules. When the alkene insertion is involved in polymerisation, regioselectivity during migratory insertion of substituted alkenes can influence the resulting material properties by affecting crystallinity,^1^ microstructure,1c,^2^ and molecular weight.^3^ Thus, appropriate control of alkene-insertion regioselectivity is an important consideration towards the development of efficient catalytic transformation of alkenes. In general, regioselectivity of alkene insertion into a palladium–carbon bond is affected by the electronic nature of alkenes. In palladium-catalysed reactions, for instance, mono-substituted alkenes bearing electron-withdrawing groups, such as acrylates or acrylonitrile, tend to undergo 2,1-insertion in their migratory insertion into a palladium–carbon bond, because the migratory group generally adds to the terminal sp^2^ carbon bearing a larger LUMO coefficient.^4^,^5^ The 2,1-insertion forms a 4-membered metallacycle or the corresponding multimer generated by intermolecular coordination (Scheme 1a). It is notable that a preferential 1,2-insertion of methyl acrylate (MA) into a palladium–carbon bond, forming a 5-membered metallacycle (Scheme 1b), was recently accomplished in a stoichiometric reaction using a palladium species ligated by an extremely bulky ligand.^6^

**Fig. 1:**
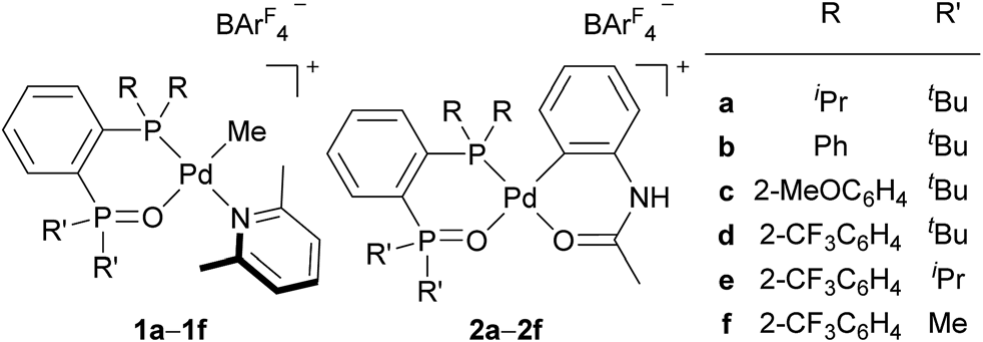
Examples of BPMO–palladium complexes. Ar^F^ = 3,5-bis(trifluoromethyl)phenyl.

In the last two decades, intensive studies have been devoted to catalyst development for coordination–insertion copolymerisation of olefins with polar vinyl monomers, aimed at the production of functionalised polyolefins.^5^ Acrylates are some of the most common polar vinyl monomers used for the copolymerisation with ethylene. Despite the importance, a limited number of papers have discussed the regioselectivity of acrylate insertion in the polymerisation processes. In the case of palladium/α-diimine catalysts, 2,1-insertion of MA selectively proceeds,^7^,^8^ although the formed 4-membered chelate complex rapidly isomerises *via* chain walking to form the corresponding 6-membered palladacycle before the next chain propagation.^7^ The same preferential 2,1-insertion of MA has been observed in the case of palladium/phosphine-sulfonate catalysts.^9^,^10^ As an exception, selective 1,2-insertion of MA was reported with a [P–SO_3_]–type ligand bearing a bulky diazaphospholidine group,^6^ although the catalyst did not promote ethylene/MA copolymerisation.6*b* Recently, ethylene/MA cooligomerization by palladium/phosphine-phosphonate catalysts was reported, in which chain-end analysis suggested coexistence of 2,1- and 1,2-insertion of MA.^11^ In this regard, we previously reported that cationic palladium complexes possessing a BPMO ligand (an analog of **1a** bearing SbF_6_^–^ in place of BAr^F^_4_^–^ (Ar^F^ = 3,5-bis(trifluoromethyl)phenyl) as a counter anion, and **2a** (Fig. 1))^12^ mediate the copolymerisation of ethylene with a number of polar monomers such as acrylonitrile, vinyl acetate, allyl acetate, and butyl vinyl ether.^13^ Rather surprisingly, however, the best of the original BPMO–palladium catalysts were not applicable to the copolymerisation of ethylene and MA; only trace amounts of copolymer were formed after 15 h at 80 °C (*vide infra*). This observation was puzzling considering that MA has been the most reactive comonomer for copolymerisation with ethylene,^5^,^14^,^15^ and we set out to understand this phenomenon with the expectation that knowledge of the mechanistic limitations of **1a** and **2a** might shed light on the unique behavior of catalysts ligated by a BPMO compared to established polymerisation catalyst classes.^16^

Here we report catalyst-controlled 1,2- and 2,1-insertion regioselectivity in the ethylene/MA copolymerisation by palladium complexes possessing a chelating bisphosphine monoxide (BPMO) ligand. Detailed mechanistic studies revealed that a 5-membered palladacycle intermediate formed *via* 1,2-insertion of acrylate retards the (co)polymerisation.^17^ Newly designed BPMO ligands possessing aryl groups on the phosphine moiety (Fig. 1) are reported here to preferentially promote 2,1-insertion of acrylate and achieve the copolymerisation of ethylene and various polar comonomers.

## Results and discussion

### Stoichiometric reactions of alkyl-BPMO–palladium complexes with methyl acrylate

**Scheme 2:**
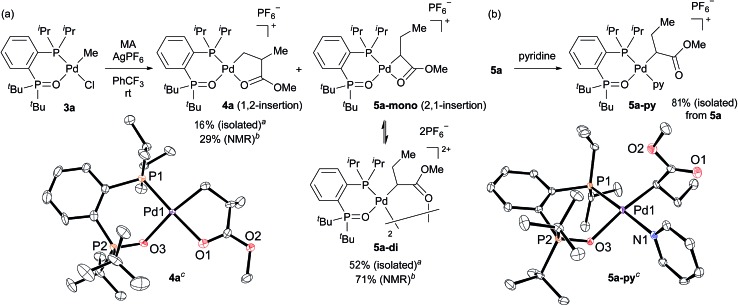
(a) Stoichiometric reaction of methyl acrylate with complex **3a** and (b) reaction of **5a** mixture with pyridine. ^*a*^ The reaction to isolate **4a** and **5a** was performed in (trifluoromethyl)benzene. ^*b*^ The reaction to determine the NMR yields was performed in dichloromethane. ^*c*^ For X-ray structures of **4a** and **5a-py**, thermal ellipsoids are shown at 50% probability. Hydrogen atoms, counter anions, and disordered fragments are omitted for clarity.

**Fig. 2:**
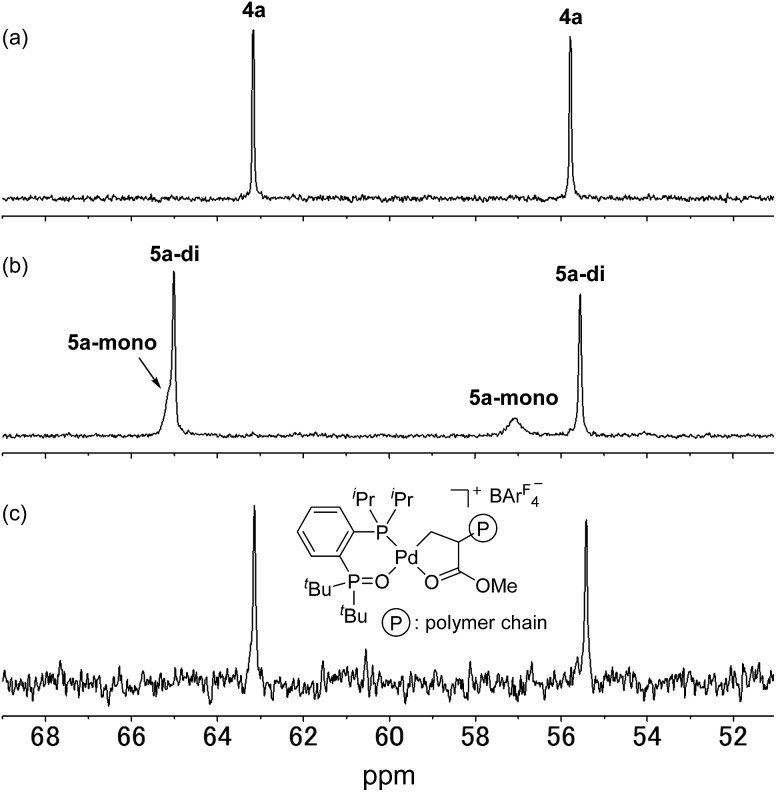
^31^P NMR spectra of (a) **4a**, (b) **5a** (3.0 × 10^–2^ M) and (c) the reaction mixture after copolymerisation of ethylene with MA catalysed by **1a** (202 MHz, 1,1,2,2-tetrachloroethane-*d*_2_).

We initially attempted to determine the organometallic product(s) from the reaction of an alkyl-BPMO–palladium complex with MA to gain insight into the structure of any potential deactivated catalyst states. Treatment of chloro(methyl)palladium complex **3a** with silver hexafluorophosphate in the presence of MA in dichloromethane for 1 hour at room temperature (Scheme 2a) led to the formation of two distinct palladium products, as observed by ^31^P NMR spectroscopy. The two products, **4a** and **5a**, were formed in 29% and 71% yield, respectively, as estimated by integration of ^1^H coupled ^31^P NMR resonances against an external PPh_3_ standard. As the rapid decomposition of **4a** during the evaporation of solvent made the isolation of **4a** and **5a** difficult, we repeated the same reaction in (trifluoromethyl)benzene, in which **4a** is soluble but **5a** is insoluble. This significant solubility difference of **4a** and **5a** in (trifluoromethyl)benzene facilitated their separation by fractional crystallization to give 16% and 52% isolated yields, respectively. Recrystallization of crude **4a** from (trifluoromethyl)benzene/diethyl ether provided single crystals suitable for X-ray analysis (Scheme 2a). The solid state structure of the 5-membered palladacycle (**4a**) was consistent with NMR spectroscopic data in solution (see Fig. S37–46†). Complex **4a** exhibited a pair of characteristic ^31^P NMR resonances at *δ*_P_ 63.2 and 55.8 ppm that correspond to the phosphine oxide and phosphine moieties of the BPMO (Fig. 2a). These characteristic resonances proved useful in determining the fate of the BPMO–palladium catalysts during ethylene/MA copolymerisations (*vide infra*). The formation of **4a** must occur by initial 1,2-insertion of MA into the Pd–C bond of **3a**, which is an uncommon regioselectivity for reactions of acrylates regardless of the nature of the transition metal complex.^6^

Unfortunately, high quality crystals of **5a** could not be obtained after repeated attempts, but a dimeric structure (**5a-di**) was suggested by low resolution X-ray diffraction data.^18^ NMR analysis of the isolated material in a 3.0 × 10^–2^ M solution in 1,1,2,2-tetrachloroethane-*d*_2_ at 25 °C exhibited two pairs of resonances at *δ*_P_ 65.1 and 57.1 ppm or 65.0 and 55.5 ppm in a 35 : 65 ratio indicative of a mixture of two BPMO–palladium complexes (Fig. 2b). These compounds were assigned as monomer **5a-mono** and dimer **5a-di**, respectively, in dynamic equilibrium at room temperature based on the correlation between the relative populations and solution concentration: the ratios of **5a-mono** : **5a-di** were 50 : 50 and 58 : 42 in 1.5 × 10^–2^ and 1.0 × 10^–2^ M solutions, respectively.^19^ Reaction of pyridine and **5a**, however, did converge to a single new species (**5a-py**) whose structure was successfully determined by X-ray analysis (Scheme 2b). This derivative complex corresponds to reaction of **3a** and MA with 2,1-insertion regioselectivity. Thus, both organometallic products **4a** and **5a** arise from migratory insertion of MA, but occur with opposite regioselectivity.

### Analysis of catalyst residue after ethylene/methyl acrylate copolymerisation

We next analysed the palladium products formed during the copolymerisation of ethylene and MA using **1a** to determine whether an analogue to palladacycles **4a** or **5a** formed by 1,2- or 2,1-insertion of MA, respectively, were also generated during catalysis.^20^ Following the reaction of ethylene and MA in the presence of **1a** in toluene for 15 h at 80 °C, the resulting mixture was analysed by electrospray ionization (ESI) mass spectrometry. Two series of ion signals corresponding to [BPMO–Pd + (ethylene)_*n*_ + MA + CH_3_] and [BPMO–Pd + (ethylene)_*m*_ + MA + H] were observed, suggesting the insertion of one MA after several consecutive insertions of ethylene. After evaporation of solvent, the non-volatile residue was analysed by ^1^H NMR spectroscopy. A characteristic resonance at *δ*_H_ 2.82 ppm (dddd, *J* = 6,6,6,6 Hz) was assigned as a methine proton alpha to a coordinating ester group (Fig. S152†), similar to the methine resonance in the isolated **4a** (Fig. S37†). This palladium product was the major species formed during the copolymerisation (>82% based on **1a**). The ^31^P NMR chemical shifts at *δ*_P_ 63.1 and 55.4 ppm (Fig. 2c) are also similar to those of **4a** (Fig. 2a).^21^ These data are consistent with 1,2-insertion of MA during attempted copolymerisation with **1a**, and we suspected that this palladacyclic intermediate that lacks an open coordination site for monomer was functioning as a kinetic trap during catalysis.

### Ethylene polymerisation initiated and catalysed by alkyl-BPMO–palladium complexes

To probe the role of palladacycles as potential kinetic traps during catalysis, the activities of isolated metallacycles **4a** and **5a-mono**/**5a-di** towards ethylene polymerisation were compared to that of typical precatalyst **3a** in the presence of silver hexafluorophosphate as a halogen scavenger (Table 1). In these experiments, the first insertion of ethylene to initiate the polymerisation occurs with a distinct palladium species in each case, but later propagation steps should occur through an identical catalytic species. At short reaction times the average reaction rate should be weighted towards the initiation phase of the polymerisation and thus qualitatively reflect how the first migratory insertion is affected by the stability of a metallacyclic complex. The results of these experiments (Table 1) clearly indicate that both palladacycle complexes react more slowly than methylpalladium precatalyst **3a**. Notably, 5-membered palladacycle **4a** is also significantly less reactive towards ethylene insertion compared to the dynamic mixture of 4-membered palladacycle **5a-mono** and dimer **5a-di**. These data suggest that any new BPMO–palladium catalyst that is active for polymerisations of acrylates likely would need to enforce high 2,1-insertion regioselectivity to avoid catalyst inhibition through formation of a stable 5-membered palladacycle.

**Table 1:** Homopolymerisation of ethylene by BPMO–palladium complexes **3a**, **4a**, and **5a**^*a*^

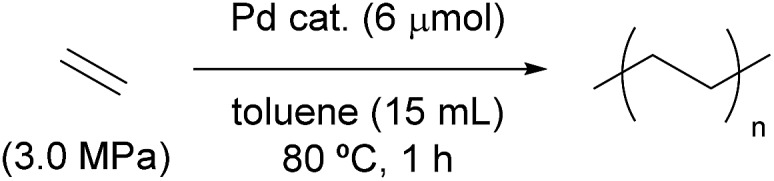					
Entry	Catalyst	Yield (g)	Activity (kg mol^–1^ h^–1^)	*M*_n_ ^*b*^ (10^3^)	*M*_w_/*M*_n_^*b*^
1	**3a** + AgPF_6_^*c*^	2.51	420	23	3.3
2	**4a**	0.05	8	30	2.3
3	**5a-mono**/**5a-di**	0.32	54	23	3.0

^*a*^Conditions: toluene (15 mL), ethylene (3.0 MPa), and palladium catalyst (6 μmol) were stirred in a 50 mL stainless autoclave for 1 h at 80 °C.

^*b*^Determined by SEC analysis using polystyrene as an internal standard and calibrated by universal calibration.

^*c*^6 μmol of AgPF_6_ was added.

### Ethylene polymerisation initiated and catalysed by aryl-BPMO–palladium complexes

Because triarylphosphines are generally air stable and can be prepared in a modular fashion from haloarene starting materials, we also developed in parallel to our mechanistic study a new series of BPMO ligands and corresponding BPMO–palladium catalysts that incorporated this structural motif. The easily derivatised BPMO framework with a diarylphosphino moiety ultimately allowed us to empirically identify several new BPMO–palladium catalysts that were similar to **1a** and **2a** in activity for ethylene polymerisation (Table 2), but several of these were also substantially more active during copolymerisations with acrylate monomers (*vide infra*).

**Table 2:** Homopolymerisation of ethylene in the presence of cationic BPMO–palladium complexes^*a*^

Entry	Catalyst	Yield (g)	Activity (kg mol^–1^ h^–1^)	*M*_n_ ^*b*^/10^3^	*M*_w_/*M*_n_^*b*^	Me br.^*c*^ (/10^3^ C)
1	**1a**	2.00	2700	31	3.1	5
2	**1b**	0.10	130	17	2.4	11
3	**1c**	1.74	2300	12	4.2	14
4	**1d**	0.79	1100	21	2.8	17
5	**2a**	2.11	2800	29	2.1	5
6	**2c**	1.40	1900	10	5.5	11
7	**2d**	1.64	2200	14	2.4	22
8	**2e**	1.75	2300	10	4.5	14
9	**2f**	1.17	1600	9.3	3.1	2

^*a*^Conditions: toluene (15 mL), ethylene (3.0 MPa), and palladium catalyst (0.75 μmol) were stirred in a 50 mL stainless autoclave for 1 h at 100 °C.

^*b*^Determined by SEC analysis using polystyrene as an internal standard and calibrated by universal calibration.

^*c*^Determined by quantitative ^13^C NMR analysis..

As we previously reported,^13^ complex **1b** with a simple diphenylphosphino group displayed modest ethylene polymerisation activity (Table 2, entry 2). In sharp contrast, BPMO–palladium complexes with *ortho*-substituted aryl groups on the phosphine exhibited markedly improved activity. Complexes **1c** and **1d** with bis(2-methoxyphenyl)phosphino and bis[2-(trifluoromethyl)phenyl]phosphino groups, respectively, promoted ethylene polymerisation with activity comparable to the best first generation catalyst **2a** (entries 3 and 4). Palladacycle analogues **2c** and **2d** performed similarly (entries 6 and 7) to the methylpalladium-type precatalysts. Notably, an increase in the methyl branching ratio was detected in polyethylenes formed by these diarylphosphino BPMO–palladium complexes (compare entries 2–4 with 1; entries 6 and 7 with 5) relative to **1a** or **2a**. Palladium complexes with the same bis[2-(trifluoromethyl)phenyl]phosphino group but differing alkyl substituents at the phosphine oxide position of the BPMO (**2d–2f**) were also evaluated (entries 7–9). Decreasing substituent size from *tert*-butyl (**2d**) to isopropyl (**2e**) to methyl (**2f**) had a small effect on the molecular weight of the resulting polyethylene, but a significant change in methyl branching ratio from 22 to 14 to 2 per 1000 carbons, respectively, was observed. This data emphasises that alteration of the substituents at the phosphine oxide ligand in a BPMO–palladium catalyst offers an additional and useful site of perturbation to tune catalyst function and the structure of the resulting polymers, which is not possible with existing catalyst families such as Drent-type palladium/phosphine-sulfonate complexes.

### Copolymerisation of ethylene and methyl acrylate

Most importantly, a significant improvement in the activity for copolymerisation of ethylene and MA was observed using BPMO–palladium catalysts that possess an *ortho*-substituted diarylphosphino moiety (Table 3). Complex **1b** with a simple diphenylphosphino fragment were inert for copolymerisation (entry 2), but dramatically improved activity was observed using **1c**, **1d**, **2c**, or **2d** (entries 3, 4, 6, and 7). The low activity of phenyl-substituted ligands could be attributed to fast chain transfer reactions, as is generally observed in related catalysts.^5^,^11^,15e Ligands bearing electron-donating MeO groups, **1c** and **2c**, gave higher copolymer molecular weights and incorporation ratios of MA than those bearing electron-withdrawing CF_3_ groups, **1d** and **2d**.^22^ It is worth noting that the molecular weight and the incorporation ratio of MA were comparable to copolymers formed using state-of-the-art Drent-type phosphine-sulfonate palladium complexes.15b–e All copolymers were linear and random as determined by ^1^H NMR and quantitative ^13^C NMR spectroscopic analysis. The identity of the phosphine oxide substituents also had an important influence on reaction rate; drastically improved activity occurred as the size of substituent decreased from *tert*-butyl to isopropyl to methyl (entries 7–10). The exceptionally high catalytic activity using complex **2f** (540 kg mol^–1^ h^–1^; entry 10),^23^,^24^ again highlights the power of exploiting steric perturbation near the oxygen atom of this chelating ligand, which is not possible with many established catalysts with [P–O]-type ancillary ligands such as a phosphine-sulfonate.

**Table 3:** Copolymerisation of ethylene and methyl acrylate in the presence of cationic BPMO–palladium complexes^*a*^

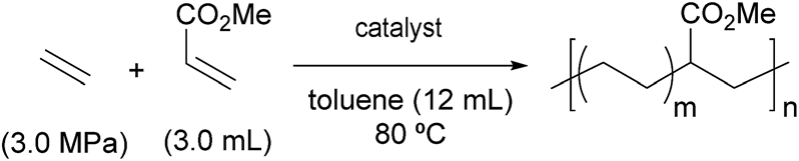							
Entry	Catalyst^*b*^	Time (h)	Yield (g)	Activity (kg mol^–1^ h^–1^)	*M*_n_ ^*c*^/10^3^	*M*_w_/*M*_n_^*c*^	Incorp.^*d*^ (mol%)
1	**1a** (10)	15	0.03	0.2	1.6	1.7	2.5
2	**1b** (10)	15	0	—	—	—	—
3	**1c** (10)	15	0.61	4.1	33	2.3	2.3
4	**1d** (10)	15	0.89	5.9	14	2.1	0.9
5	**2a** (10)	15	0.01	0.1	—	—	3.3^*e*^
6	**2c** (10)	15	0.40	2.7	24	2.8	3.4
7	**2d** (10)	15	1.37	9.1	17	2.6	1.0
8	**2e** (0.75)	15	0.37	33	17	3.6	0.9
9	**2e** (0.75)	1	0.03	41	18	2.3	1.2^*e*^
10	**2f** (0.75)	1	0.41	540	6.9	2.9	0.5

^*a*^Conditions: ethylene, palladium catalyst, and comonomer were stirred in a 50 mL stainless autoclave at 80 °C.

^*b*^Numbers in parenthesis are the amount of catalyst (μmol).

^*c*^Determined by SEC analysis using polystyrene as an internal standard and calibrated by universal calibration.

^*d*^Incorporation of MA determined by quantitative ^13^C NMR analysis.

^*e*^Incorporation of MA determined by ^1^H NMR analysis.

### Copolymerisation of ethylene and other polar monomers by aryl-BPMO–palladium complexes

We performed copolymerisation of ethylene with various polar monomers using complexes bearing 2-methoxyphenyl group (**1c**) and 2-(trifluoromethyl)phenyl group (**2d**, **2f**) (Table 4). Both catalysts **1c** and **2d** catalysed copolymerisation of ethylene and allyl acetate with comparable performance to common phosphine-sulfonate catalysts^25^ and the original alkyl-BPMO catalyst **2a**^13^ in terms of activity, molecular weight, and incorporation ratio (entries 1 and 2). In contrast, catalyst **2f** showed about 6-fold higher activity than **2d**, while maintaining similar molecular weight and incorporation ratio (entry 3). Thus, exceptionally high activity of **2f** was not limited to the copolymerization of acrylates. In the case of butyl vinyl ether, the copolymerisation using catalyst **1c** proceeded (entry 4), while no or little incorporation of vinyl ether into polyethylene was observed, along with the formation of poly(butyl vinyl ether), when **2d** or **2f** was used (entries 5 and 6). This significant difference of catalyst behaviour is probably due to the decreased electrophilicity of **1c** by electron-donating methoxy group that suppress the cationic polymerisation of butyl vinyl ether observed in entry 5 and 6. The same trend was observed in the copolymerisation of acrylonitrile (entries 7–9). Thus, catalyst **1c** could afford ethylene/acrylonitrile copolymer, but **2d** afforded only oligoethylene and **2f** afforded even no solid product. In this case, strong σ-coordination of acrylonitrile to the electrophilic palladium centre of **2d** and **2f** would prevent the copolymerisation with ethylene. This higher electron-withdrawing ability of 2-(trifluoromethyl)phenyl than 2-methoxyphenyl group may partially explain the higher activity of **1d** and **2d** toward copolymerisation of ethylene with MA compared to **1c** and **2c** (compare entries 3 and 4 in Table 3, entries 6 and 7 in Table 3).^26^

**Table 4:** Copolymerisation of ethylene and polar monomers in the presence of cationic BPMO–palladium complexes^*a*^

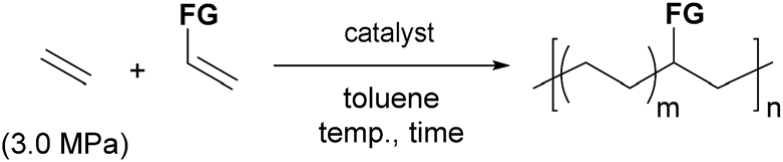											
Entry	Catalyst	FG	Comonomer (mL)	Toluene (mL)	Temperature (°C)	Time (h)	Yield (g)	Activity (kg mol^–1^ h^–1^)	*M*_n_ ^*b*^ (10^3^)	*M*_w_/*M*_n_	Incorp.^*c*^ (mol%)
1	**1c**	CH_2_OAc	3.0	12	80	12	0.35	2.9	5.1	2.0	0.9
2	**2d**	CH_2_OAc	3.0	12	80	8	0.50	6.3	6.4	2.3	0.8^*d*^
3	**2f**	CH_2_OAc	3.0	12	80	8	3.02	38	5.2	4.0	0.6
4	**1c**	OBu	5.0	10	80	26	0.20	0.8	5.7	2.2	0.7
5	**2d**	OBu	5.0	10	80	20	2.91^*e*^	15	18	2.6	0
6	**2f**	OBu	5.0	10	80	20	1.96^*e*^	9.8	11	3.8	0.1
7	**1c**	CN	2.5	2.5	100	72	0.12	0.2	1.9	3.4	2.4
8	**2d**	CN	2.5	2.5	100	72	0.09	0.1	0.4	1.6	0^*d*^
9	**2f**	CN	2.5	2.5	100	72	0	—	—	—	—

^*a*^Conditions: ethylene, palladium catalyst (10 μmol), and comonomer were stirred in a 50 mL stainless autoclave at an indicated temperature.

^*b*^Determined by SEC analysis using polystyrene as an internal standard and calibrated by universal calibration.

^*c*^Incorporation ratio of polar monomer determined by quantitative ^13^C NMR analysis.

^*d*^Determined by ^1^H NMR spectrum.

^*e*^Yield after washing with dichloromethane to remove the homopolymer of butyl vinyl ether formed as a side product.

### Origin of high copolymerisation activity

**Scheme 3:**
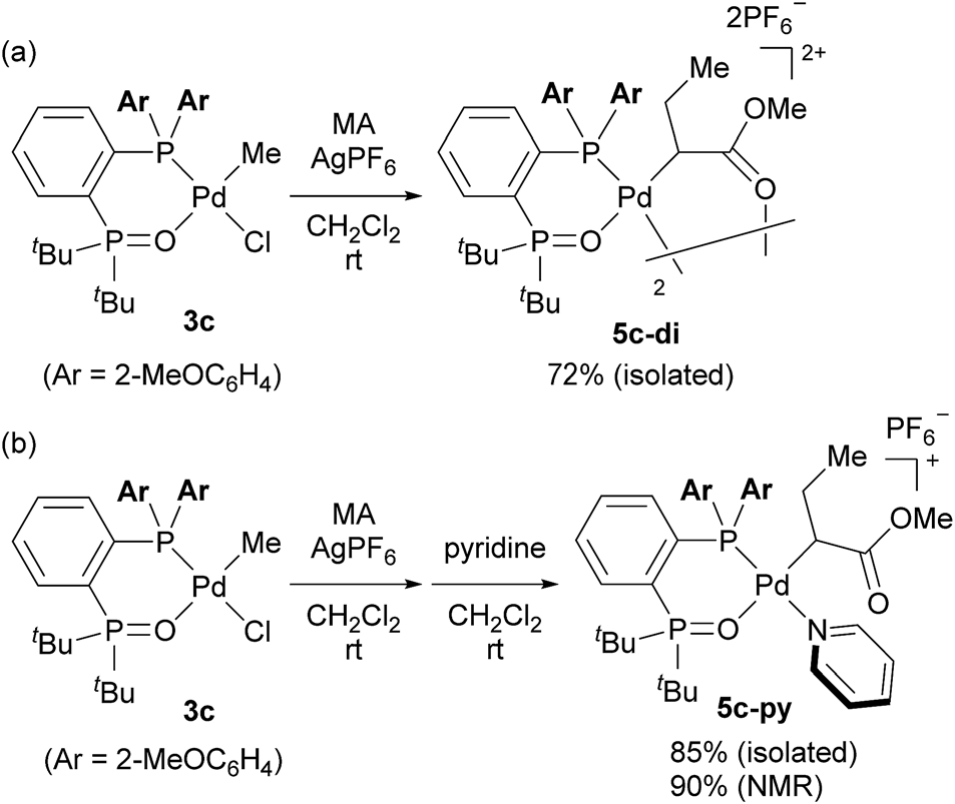
Insertion of methyl acrylate into aryl-BPMO–palladium complex **3c**.

**Fig. 3:**
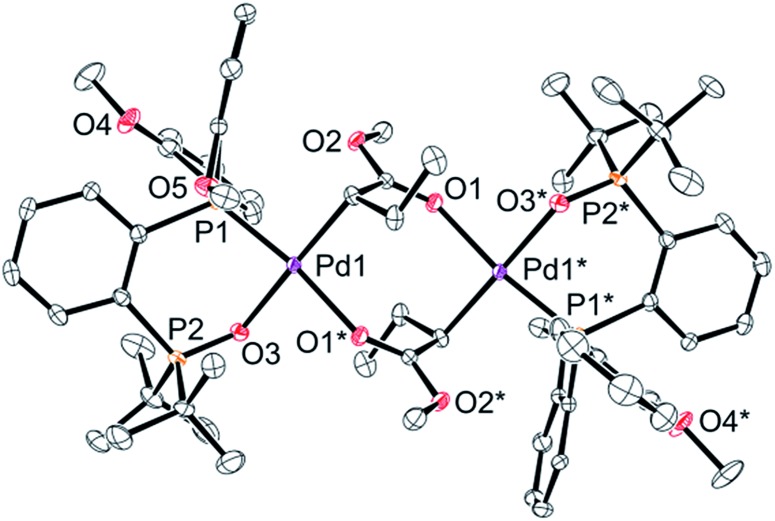
An X-ray structure of complex **5c-di**. Thermal ellipsoids are shown at 50% probability. Hydrogen atoms and counter anions are omitted for clarity.

Finally, we conducted stoichiometric experiments to understand the origin of the much higher activity of this second generation of aryl-BPMO–palladium catalysts towards ethylene/MA copolymerisation. Reaction of bis(2-methoxyphenyl)phosphine complex **3c** with silver hexafluorophosphate in the presence of MA at room temperature resulted in formation of **5c-di** in 72% isolated yield (Scheme 3a). The dimeric structure of **5c-di** was determined by single crystal X-ray analysis (Fig. 3), which verified the major palladium product from this reaction resulted from 2,1-insertion of MA. It is worth noting that the distance of Pd1–O5 is *ca.* 3.45 Å, which suggests no interaction between the methoxy group and the palladium centre.

Additionally, reaction of **3c** with silver hexafluorophosphate in the presence of MA followed by trapping with pyridine afforded **5c-py** in high overall yield (90%) as determined by ^1^H NMR yield (Scheme 3b). Importantly, a palladium complex corresponding to 1,2-insertion of MA was not detected in either case. The selective 2,1-insertion of MA was also suggested for the reaction of MA with an analogous aryl-BPMO–palladium complex with a bis[2-(trifluoromethyl)phenyl]phosphino group (**3d**; see ESI†). These experiments indicate a clear difference in insertion regioselectivity for BPMO–palladium catalysts that are, or are not, active for copolymerisation of ethylene and MA, favoring exclusive 2,1-insertion regioselectivity of MA for highly active BPMO–palladium complexes with an *ortho*-substituted diarylphosphino group.

## Conclusions

In conclusion, a new generation of cationic bisphosphine monoxide–palladium catalysts with a diarylphosphino moiety was shown to exhibit markedly improved performance for the copolymerisation of ethylene with methyl acrylate. Mechanistic studies revealed that the contrasting reactivity between these aryl-BPMO and previously reported inactive alkyl-BPMO catalysts was a shift to higher 2,1-insertion regioselectivity of methyl acrylate that avoids generation of a stable palladacycle intermediate that is poorly reactive towards additional monomer enchainment. Newly developed aryl-BPMO catalysts can also copolymerise ethylene with other industrially important polar monomers. Future studies will be directed toward revealing the reason why the higher 2,1-selectivity was observed for reaction of methyl acrylate with **3c** or **3d** as compared to **3a**.^27^

## Supplementary Material

Supplementary information

Crystal structure data
