# Supplementary material for: Ligand-controlled insertion regioselectivity accelerates copolymerisation of ethylene with methyl acrylate by cationic bisphosphine monoxide–palladium catalysts
Source: Chem Sci. 2015 Nov 3;7(1):737–44. doi: 10.1039/c5sc03361f (PMC5952993; doi:10.1039/c5sc03361f)
Supplement: Supplementary file 1 [file SC-007-C5SC03361F-s001.pdf]

Electronic Supplementary Material (ESI) for Chemical Science.
This journal is © The Royal Society of Chemistry 2015

Supplementary Information for

Ligand-Controlled Insertion Regioselectivity Accelerates Copolymerisation of Ethylene with Methyl Acrylate by Cationic Bisphosphine Monoxide–Palladium Catalysts

Yusuke Mitsushige,[a] Brad P. Carrow,[b] Shingo Ito,[a] Nozaki Kyoko,*,[a]

[a] *Department of Chemistry and Biotechnology, Graduate School of Engineering, The University of Tokyo 7-3-1 Hongo, Bunkyo-ku, Tokyo 113-8656, Japan,*

[b] *Department of Chemistry, Princeton University, Princeton, New Jersey, USA*

1. Experimental Section

1-1 General……S2

1-2 Preparation of Ligands and Catalysts……S4

1-3 General Procedure for Polymerisations……S26

1-4 Additional Data of Polymerisation……S27

2. NMR Spectra of Ligands and Catalysts……S28

3. Thermolysis of MA-Inserted Complexes……S45

4. Characterisation of MA-Inserted Complexes

4-1 NMR Spectra of 4a……S48

4-2 NMR Spectra of 5a……S53

4-3 NMR Spectra of 5a-py……S57

4-4 NMR Spectra of 5c……S63

4-5 NMR Spectra of 5c-py……S71

4-6 NMR Spectra of 5d-py……S79

5. NMR Spectra of Polymer……S83

6. Analysis of Reaction Mixture After Copolymerisation of Ethylene and MA by 1a……S109

7. Discussion on Regioselectivity of MA Insertion……S113

8. X-ray Crystallographic Analyses……S115

1. Experimental Section

1-1 General

Manipulations

All reactions were carried out using standard Schlenk techniques under argon purified by passing through a hot column packed with BASF catalyst R3-11. All copolymerisations were performed in a 50- or 300-mL stainless steel autoclave.

Instrumentation

NMR spectra were recorded on JEOL JNM-ECP500 (^{1}H: 500 MHz, ^{13}C: 126 MHz, ^{31}P: 202 MHz, ^{19}F 470 MHz) or JEOL JNM-ECS400 (^{1}H: 400 MHz, ^{13}C: 101 MHz, ^{31}P: 162 MHz, ^{19}F 376 MHz) or BRUKER Ascend500 (^{1}H: 500 MHz, ^{13}C: 126 MHz, ^{31}P: 202 MHz, ^{19}F 470 MHz) NMR spectrometers at ambient temperature unless otherwise noted. Chemical shift values for protons are referenced to the residual proton resonance of chloroform-*d* ($CDCl_3$, δ: 7.26) or 1,1,2,2-tetrachloroethane-d_2 ($C_2D_2Cl_4$, δ: 6.00), or dichloromethane-d_2 (CD_2Cl_2, δ: 5.32). Quantitative ^{13}C NMR analyses of polymers were performed in a 5-mm probe on *ca.* 15 weight% solutions of the polymers and *ca.* 0.05 M $Cr(acac)_3$ as a relaxation agent in 1,1,2,2-tetrachloroethane or $C_2D_2Cl_4$ unlocked at 120–130 °C using a 90° pulse of 9.0 μs, a spectral width of 31 kHz, a relaxation time of 5–10 s, an acquisition time = 2 s, and inverse-gated decoupling (JEOL JNM-ECS400) or using a 30° pulse of 16.8 μs, a spectral width of 30 kHz, a relaxation time of 2 s, an acquisition time = 1.1 s, and inverse-gated decoupling (BRUKER Ascend500).[i,ii] Chemical shift values for carbons are referenced to the carbon resonance of $CDCl_3$ (δ: 77.2) or 1,1,2,2-tetrachloroethane (δ: 74.2) or $C_2D_2Cl_4$ (δ: 73.8) or CD_2Cl_2 (δ: 53.8). Size exclusion chromatography (SEC) analyses were carried out with a Tosoh instrument (HLC-8121GPC/HT) equipped with two SEC columns (Tosoh TSKgel GMHHR-H(S)HT) and a refractive index (RI) detector by eluting the columns with 1,2-dichlorobenzene at 1.0 mL/min at 145 °C. Molecular weights were determined using narrow polystyrene standards and were corrected for polyethylene by universal calibration using the Mark–Houwink parameters of Rudin: K = 1.75×10^{-2} cm^3/g and α = 0.67 for polystyrene and K = 5.90×10^{-2} cm^3/g and α = 0.69 for LLDPE.[iii] X-ray crystallographic analysis was performed on a Rigaku Varimax with Saturn diffractometer. Elemental analysis was performed by the Microanalytical Laboratory, Department of Chemistry, Graduate School of Science, The University of Tokyo. GC analysis was performed by Shimadzu

[i] (a) Z. Guan, P. M. Cotts, E. F. McCord, S. J. McLain, *Science*, 1999, **283**, 2059–2062. (b) P. M. Cotts, Z. Guan, E. McCord. S. McLain, *Macromolecules*, 2000, **33**, 6945–6952.

[ii] J. C. Randall, C. J. Ruff, M. Kelchtermans, B. H. Gregory, *Macromolecules*, 1992, **25**, 2624–2633.

[iii] V. Grinshpun, A. Rudin, *Makrom. Chem., Rapid Commun.*, 1985, **6**, 219–223.

GC-2014 equipped with InertCap 5MS/Sil capillary column (0.25 ID, 0.25 μm df, 30 m) and a FID detector. High resolution mass spectra were recorded on JEOL JMS-T100LP AccuTOF LC-plus.

Materials. Anhydrous dichloromethane (DCM), diethyl ether, hexane, tetrahydrofuran (THF), and toluene were purchased from Kanto Chemical Co. Inc. (Kanto) and purified by the method of Pangborn *et al.*[iv] Ethylene (>99.9%) was purchased from Takachiho Chemical Industrial Co., Ltd., dried, and deoxygenated by passing through columns. The following reagents were purchased from Tokyo Chemical Industry, Co., Ltd. (TCI) and purified by distillation over CaH_2: allyl acetate, methyl acrylate, butyl vinyl ether, and acrylonitrile. The following reagents were purchased and used as received: butyllithium (BuLi) in hexane (Kanto), *tert*-butyllithium (^{t}BuLi) in pentane (Kanto), silver hexafluoroantimonate (TCI), silver hexafluorophosphate (Aldrich), dimethyl-phosphinic chloride (Aldrich), chlorodiisopropylphosphine (Aldrich), trichlorophosphine (TCI), sodium tetrakis[3,5-bis(trifluoromethyl)phenyl]borate ($NaBAr^F_4$, Matrix Scientific), 2-bromoanisole (TCI), 2-bromobenzotrifluoride (Aldrich), 35% aqueous hydrogen peroxide (Kanto), dehydrated pyridine (Kanto), 2,6-lutidine (TCI), methyl 2-*trans*-octenoate (TCI). The following compounds were prepared according to the procedures in literature: (2-bromophenyl)dichlorophosphine[v], di-*tert*-butylphenylphosphine oxide[vi], chlorido(cycloocta-1,5-diene)(methyl)palladium ((cod)PdMeCl)[vii], di-μ-chloride-di[κ^2-(2-acetanilido)palladium][viii], {(κ^2-*P*,*O*)-[2-($^i Pr_2$P)C_6H_4]P(O)$^t Bu_2$}PdMeCl[ix], {{(κ^2-*P*,*O*)-[2-($^i Pr_2$P)C_6H_4P(O)$^t Bu_2$]PdMe(2,6-lutidine)}$^+$$SbF_6^-$[ix], {{($\kappa^2$-*P*,*O*)-[2-($Ph_2$P)$C_6H_4$P(O)$^t Bu_2$]PdMe(2,6-lutidine)}$^+$$SbF_6^-$[ix], {{($\kappa^2$-*P*,*O*)-[2-($^i Pr_2$P)$C_6H_4$]P(O)$^t Bu_2$}Pd[($\kappa^2$-*C*,*O*)-(2-acetanilido)]}$^+$$BAr^F_4{}^-$ (**2a**)[ix].

[iv] A. B. Pangborn, M. A. Giardello, R. H. Grubbs, R. K. Rosen, F. J. Timmers, *Organometallics*, 1996, **15**, 1518–1520.
[v] C. M. Reisinger, R. J. Nowack, D. Volkmer, B. Rieger, *Dalton Trans.*, 2007, 272–278.
[vi] M. Gray, B. J. Chapell, J. Felding, N. J. Taylor, V. Snieckus, *Synlett*, 1998, 422–424.
[vii] R. E. Rülke, J. M. Ernsting, A. L. Spek, C. J. Elsevier, P. W. N. M. van Leeuwen, K. Vrieze, *Inorg. Chem.*, 1993, **32**, 5769–5778.
[viii] G.-R. Peh, E. A. B. Kantchev, J.-C. Er, J. Y. Ying, *Chem. Eur. J.*, 2010, **16**, 4010–4017.
[ix] B. P. Carrow, K. Nozaki, *J. Am. Chem. Soc.*, 2012, **134**, 8802–8805.

1-2. Preparation of Ligands and Catalysts

Di-*tert*-butyl[2-(dichlorophosphino)phenyl]phosphine oxide;

[2-(Cl_2P)C_6H_4]P(O)^{t}Bu$_2$ (S1)

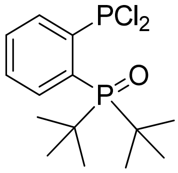

To a solution of di-*tert*-butylphenylphosphine oxide (1.4 g, 6.0 mmol, 1.0 equiv) in THF (44 mL) in a 250-mL Schlenk tube was added ^{t}BuLi (1.54 M solution in pentane, 4.1 mL, 6.3 mmol, 1.1 equiv) at −78 °C and the reaction mixture was stirred for 2 hours at −78 °C. The suspension was transferred to the solution of trichlorophophine (0.79 mL, 9.0 mmol, 1.5 equiv) in THF (30 mL) at −78 °C and the resulting mixture was stirred for 40 minutes at −78 °C. Then the dry ice bath was removed and the reaction mixture was stirred for 30 minutes at ambient temperature. After the volatile matters were removed *in vacuo*, the crude material was dissolved in THF (*ca.* 50 mL) and filtered through a pad of Celite under argon atmosphere to remove the formed lithium salts. Reprecipitation from THF/hexane gave the title product in ca. 53% yield (1.09 g, ca. 3.2 mmol) as a colorless powder. It was difficult to completely remove all LiCl salt and thus the product was used for next reactions without further purification.

^{1}H NMR ($C_2D_2Cl_4$, 500 MHz) δ: 8.61 (d, *J* = 8.2 Hz, 1H), 8.06–8.02 (m, 2H), 7.97–7.93 (m, 1H), 1.44 (d, *J* = 17.1 Hz, 18H); ^{13}C{^{1}H} NMR ($C_2D_2Cl_4$, 126 MHz) δ: 150.2 (dd, *J* = 58, 15 Hz), 135.8 (s), 133.4 (d, *J* = 10 Hz), 132.5 (dd, *J* = 21, 11 Hz), 130.0 (d, *J* = 15 Hz), 121.3 (d, *J* = 80 Hz), 37.0 (d, *J* = 40 Hz, 2C), 25.8 (s, 6C); ^{31}P{^{1}H} NMR (THF, 162 MHz) δ: 139.1 (d, *J* = 9 Hz), 72.4 (d, *J* = 9 Hz).

{2-[Bis(2-methoxyphenyl)phosphino]phenyl}di-*tert*-butylphosphine oxide;

{2-[(2-OMeC_6H_4)$_2$P]C_6H_4}P(O)^{t}Bu$_2$ (S2)

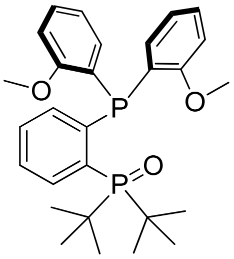

To a solution of 2-bromoanisole (0.75 mL 6.0 mmol, 2.0 equiv) in THF (40 mL) was added BuLi (1.64 M solution in hexane, 3.7 mL, 6.1 mmol, 2.0 equiv) at −78 °C and the reaction mixture was stirred for 70 minutes at −78 °C. The resulting solution was transferred to the solution of **S1** (1.02 g, 3.0 mmol, 1.0 equiv) in THF (20 mL) at −78 °C and the reaction mixture was stirred for 30 minutes

at −78 °C. Then the dry ice bath was removed and reaction mixture was stirred at ambient temperature for 90 minutes. The reaction was quenched with water (*ca.* 100 μl) and solvent was removed *in vacuo*. The crude material was dissolved into DCM and filtered through a pad of Celite to remove the formed lithium salts. The crude material was then purified by column chromatography on silica gel using DCM/MeOH (40:1) as the eluent and recrystallised from THF/hexane to afford the title product in 38% yield (543 mg, 1.1 mmol) as a colorless solid.

^{1}H NMR (CD_2Cl_2, 400 MHz) δ: 7.71–7.66 (br, 1H), 7.36–7.24 (m, 4H), 7.04 (d, *J* = 6.9Hz, 1H), 6.86 (br s, 2H), 6.80–6.77 (br, 2H), 6.58 (br s, 2H), 3.69 (br s, 6H), 1.28 (br s, 18H); ^{13}C{^{1}H} NMR ($CDCl_3$, 126 MHz) δ: 161.1 (d, *J* = 17 Hz, 2C), 146.4 (br s, 2C), 136.7 (d, *J* = 10 Hz), 136.4 (dd, *J* = 78, 23 Hz), 134.4 (br s, 2C), 131.3 (br s), 130.5 (br s), 129.4 (br s, 2C), 128.5 (br s), 126.2 (br s), 120.9 (br s, 2C), 110.2 (br s, 2C), 55.6 (s, 2C), 37.5 (br s, 2C), 27.7 (br s, 6C); ^{31}P{^{1}H} NMR ($CDCl_3$, 202 MHz) δ: 57.0 (s), −23.7; Anal. Calcd for $C_{28}H_{36}O_3P_2$, C, 69.69; H, 7.52. found: C, 69.41; H, 7.42.

{2-[Bis(2-trifluoromethylphenyl)phosphino]phenyl}di-*tert*-butylphosphine oxide; {2-[(2-$CF_3C_6H_4)_2$P]C_6H_4}P(O)^{t}Bu$_2$ (S3)

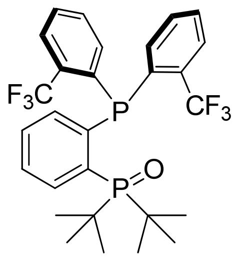

To a solution of 2-bromobenzotrifluoride (0.61 mL. 4.5 mmol, 2.3 equiv) in THF (27 mL) was added BuLi (1.60 M solution in hexane, 2.5 mL, 4.1 mmol, 2.0 equiv) at −78 °C and the reaction mixture was stirred for 2 hours at −78 °C. The resulting solution was transferred to the solution of **S1** (678 mg, 2.0 mmol, 1.0 equiv) in THF (13 mL) and the reaction mixture was stirred for 1 hour at −78 °C. Then the dry ice bath was removed and the reaction mixture was stirred for 1 hour at ambient temperature. The reaction was quenched with water (64 μl) and solvent was removed *in vacuo*. The crude material was dissolved into DCM and filtered through a pad of Celite to remove the formed lithium salt. The crude material was then purified by column chromatography on silica gel using DCM/MeOH (30:1) as the eluent and recrystallised from DCM/hexane to afford the title product in 46% yield (477 mg, 0.85 mmol) as a colorless crystal.

^{1}H NMR ($CDCl_3$, 500 MHz) δ: 7.73–7.67 (m, 3H), 7.38–7.31 (m, 6H), 7.09 (dd, *J* = 7.3, 3.7 Hz, 1H), 6.88–6.84 (m, 2H), 1.34 (d, *J* = 13.7 Hz, 9H), 1.15 (d, *J* = 13.1 Hz, 9H); ^{13}C{^{1}H} NMR ($CDCl_3$, 126 MHz) δ: 144.0 (ddq, *J* = 37 Hz, 6.4 Hz, 2.8 Hz), 140.1 (dd, *J* = 31 Hz, 2 Hz), 140.0 (d, *J* = 41 Hz) 137.3 (s), 136.8 (d, *J* = 10 Hz), 136.5 (dd, *J* = 77 Hz, 25 Hz), 136.2 (s), 134.7 (qd, *J* = 30

Hz, 28 Hz), 132.7 (dq, *J* = 26 Hz, 25 Hz), 132.1 (dd, *J* = 12, 5 Hz), 131.5 (s), 130.8 (s), 130.6 (s), 128.4 (s), 127.9 (s), 126.9 (dq, *J* = 5 Hz, 5 Hz), 126.6 (d, *J* = 11 Hz), 126.4 (dq, *J* = 5 Hz, 5 Hz), 124.4 (q, *J* = 275 Hz, 1C), 124.4 (q, *J* = 276 Hz, 1C), 37.9 (dd, *J* = 56, 2 Hz), 37.2 (d, *J* = 58 Hz), 28.3 (s, 3C), 27.1 (d, *J* = 3 Hz, 3C); $^{31}P\{^{1}H\}$ NMR ($CDCl_3$, 162 MHz) δ: 58.2 (s), −9.8 (sep, *J* = 55 Hz); ^{19}F NMR ($CDCl_3$, 376 MHz) δ: −57.3 (d, *J* = 58 Hz), −57.8 (d, *J* = 58 Hz); Anal. Calcd for $C_{28}H_{30}F_6OP_2$, C, 60.22; H, 5.41. found: C, 60.00; H, 5.37.

2-[Bis(2-trifluoromethylphenyl)phosphino]bromobenzene;
2-[(2-$CF_3C_6H_4)_2P]C_6H_4Br$ (S4)

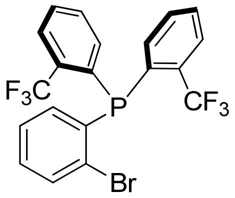

To a solution of 2-bromobenzotrifluoride (0.61 mL. 4.5 mmol, 2.0 equiv) in THF (30 mL) was added BuLi (1.60 M solution in hexane, 2.9 mL, 4.6 mmol, 2.1 equiv.) at −78 °C and the reaction mixture was stirred for 4 hours at −78 °C. The resulting solution was transferred to the solution of (2-bromophenyl)dichlorophosphine (579 mg, 2.3 mmol, 1.0 equiv) in THF (15 mL) at −78 °C and the reaction mixture was stirred for 30 minutes at −78 °C. Then the dry ice bath was removed and the resulting mixture was stirred for 1.5 hours at ambient temperature. The reaction was quenched with water (70 μL) and solvent was removed *in vacuo*. The crude material was dissolved into DCM and filtered through a pad of Celite to remove the formed lithium salt. The crude material was then purified by column chromatography on silica gel using DCM/hexane (1:1) as the eluent to afford the title product in 75% yield (802 mg, 1.7 mmol) as a colorless solid.

^{1}H NMR ($CDCl_3$, 500 MHz) δ: 7.80 (dd, *J* = 7.5, 4.1 Hz, 2H), 7.61–7.58 (m, 1H), 7.51 (dd, *J* = 7.6 Hz, 7.6 Hz, 2H), 7.45 (dd, *J* = 7.6, 7.6 Hz, 2H), 7.24–7.19 (m, 2H), 7.03–7.02 (br, 2H), 6.57 (m, 1H); $^{13}C\{^{1}H\}$ NMR ($CDCl_3$, 126 MHz) δ: 137.8 (d, *J* = 12 Hz), 136.0 (s, 2C), 135.2–134.5 (m, 5C), 133.4 (d, *J* = 2 Hz), 131.9 (s, 2C), 130.6 (s), 129.8 (s), 129.5 (s, 2C), 127.7 (s), 127.0 (dq, *J* = 5 Hz, 5 Hz, 2C) 124.3 (q, *J* = 275 Hz, 2C); $^{31}P\{^{1}H\}$ NMR ($CDCl_3$, 162 MHz) δ: −12.6 (sep, *J* = 55 Hz); ^{19}F NMR ($CDCl_3$, 376 MHz) −57.6 (br s); Anal. Calcd for $C_{20}H_{12}BrF_6P$, C, 50.34; H, 2.53. found: C, 50.25; H, 2.73.

{2-[Bis(2-trifluoromethylphenyl)phosphino]phenyl}diisopropylphosphine oxide; {2-[(2-$CF_3C_6H_4)_2P]C_6H_4\}P(O)^iPr_2$ (S5)

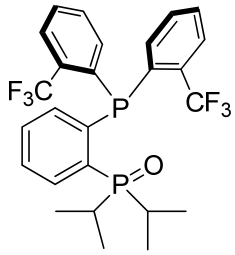

To a solution of **S4** (240 mg. 0.50 mmol, 1.0 equiv) in THF (3.0 mL) was added BuLi (1.60 M solution in hexane, 0.33 mL, 0.53 mmol, 1.1 equiv) at −78 °C and the resulting mixture was stirred for 1 hour at −78 °C. The resulting solution was transferred to the solution of chlorodiisopropylphosphine (0.24 mL, 1.5 mmol, 3.0 equiv) in THF (4.5 mL) at −78 °C and the reaction mixture was stirred for 40 minutes at −78 °C. Then the dry ice bath was removed and the reaction mixture was stirred for 40 minutes at −78 °C. After the volatile matters were removed *in vacuo* at 70 °C, the crude mixture was dissolved into DCM and filtered through a pad of Celite to remove the formed lithium salt. The crude material was dissolved into THF (ca. 5.0 mL) again and aqueous hydrogen peroxide (43 mg of 35% aqueous H_2O_2 in 1.0 mL of H_2O) was added at 0 °C and reaction mixture was stirred for 10 minutes at 0 °C. Then the reaction mixture was exposed to air and stirred for additional 2 hours at ambient temperature. Recrystallisation from DCM/hexane gave the title product in 44% yield (115 mg, 0.22 mmol) as a colorless crystal.

^{1}H NMR ($CDCl_3$, 500 MHz) δ: 8.17–8.11 (m, 1H), 7.78–7.76 (br, 2H), 7.53–7.44 (m, 3H), 7.44–7.35 (m, 3H), 7.02 (br, 1H), 7.01–6.97 (m, 1H), 6,91 (br s, 1H), 3.03 (br, 1H), 2.10 (br, 1H), 1.32 (br, 3H), 1.23 (br, 3H), 0.92 (br, 3H), 0.47 (br, 3H); ^{13}C NMR ($CDCl_3$, 126 MHz) δ: 138.5 (dd, *J* = 79, 34 Hz), 137.2 (br), 136.6 (br s, 2C), 136.3 (d, *J* = 10 Hz), 135.5 (d, *J* = 29 Hz, 2C), 135.0 (dd, *J* = 8, 8 Hz), 133.8 (br, 2C), 131.6 (s, 2C), 130.9 (d, *J* = 3 Hz), 129.2 (br s, 2C), 129.0 (d, *J* = 10 Hz), 127.0 (d, *J* = 26 Hz, 2C), 124.1 (q, *J* = 275 Hz, 2C), 29.5 (d, *J* = 65 Hz), 26.9 (d, *J* = 65 Hz), 17.0 (s), 16.6 (s), 16.3 (s, 2C); ^{31}P{^{1}H} NMR ($CDCl_3$, 202 MHz) δ: 53.6 (s), −19.9 (sep, *J* = 50 Hz); ^{19}F NMR ($CDCl_3$, 376 MHz) δ: −57.9 (d, *J* = 46 Hz).; Anal. Calcd for $C_{26}H_{26}F_6OP_2$, C, 58.87; H, 4.94. found: C, 58.68; H, 4.98.

{2-[Bis(2-trifluoromethylphenyl)phosphino]phenyl}dimethylphosphine oxide; {2-[(2-$CF_3C_6H_4)_2P]C_6H_4\}P(O)Me_2$ (S6)

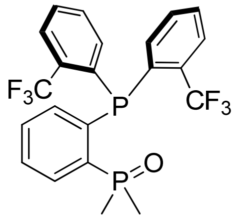

To a solution of **S4** (240 mg. 0.50 mmol, 1.0 equiv) in THF (3.0 mL) was added BuLi (1.60 M solution in hexane, 0.33 mL, 0.53 mmol, 1.1 equiv) at −78 °C and the resulting mixture was stirred for 3 hours at −78 °C. The resulting solution was transferred to the solution of dimethylphosphinic chloride (84 mg, 0.75 mmol, 1.5 equiv) in THF (2.3 mL) at −78 °C and the reaction mixture was stirred for 30 minutes at −78 °C. Then the dry ice bath was removed and the reaction mixture was stirred for 3.5 hours at ambient temperature. After the solvent was removed *in vacuo*, the crude mixture was dissolved into DCM and filtered through a pad of Celite to remove the formed lithium salts. The crude material was then purified by column chromatography on silica gel eluted with DCM/MeOH (20:1) and recrystallisation from DCM/hexane to afford the title product in 52% yield (123 mg, 0.26 mmol) as a colorless crystal.

^{1}H NMR ($CDCl_3$, 500 MHz) δ: 8.41–8.36 (m, 1H), 7.78 (br s, 2H), 7.58 (dd, *J* = 7.6 Hz, 1H), 7.50 (br s, 2H), 7.45 (dd, *J* = 7.6 Hz, 3H), 7.02–6.99 (m, 1H), 6.92 (dd, *J* = 7.1, 4.1 Hz, 2H), 2.11 (d, *J* = 13.3 Hz, 3H), 1.49 (d, *J* = 12.8 Hz, 3H); ^{13}C{^{1}H} NMR ($CDCl_3$, 126 MHz) δ: 140.8 (dd, *J* = 91, 36 Hz), 136.4 (s), 136.1 (d, *J* = 10 Hz), 135.9 (s), 135.6 (dd, *J* = 18, 12 Hz), 134.7–133.5 (m, 5C), 132.2 (s), 131.8 (d, *J* = 3 Hz), 131.8 (s), 129.9 (d, *J* = 10 Hz), 129.9 (s), 129.5 (s), 127.3 (d, *J* = 53 Hz, 2C), 124.2 (q, *J* = 281 Hz, 2C), 20.3 (dd, *J* = 71, 13 Hz), 18.7 (dd, *J* = 72, 12 Hz); ^{31}P{^{1}H} NMR ($CDCl_3$, 202 MHz) δ: 35.2 (d, *J* = 13 Hz), −20.6 (septet of doublet, *J* = 48, 9 Hz); ^{19}F NMR ($CDCl_3$, 376 MHz) δ: −57.8 (d, *J* = 46 Hz), −57.9 (d, *J* = 46 Hz); Anal. Calcd for $C_{22}H_{18}F_6OP_2$, C, 55.71; H, 3.83. found: C, 55.65; H, 3,83.

{(κ^2-*P*,*O*)-{2-[Bis(2-methoxyphenyl)phosphino]phenyl}di-*tert*-butylphosphine oxide}-(chlorido)(methyl)palladium;

{(κ^2-*P*,*O*)-{2-[(2-OMeC_6H_4)$_2$P]C_6H_4}P(O)^{t}Bu$_2$}PdMeCl (3c)

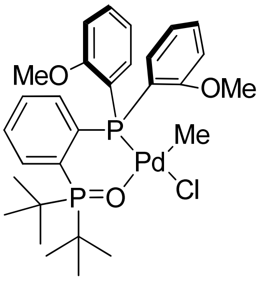

To a mixture of **S2** (250 mg, 0.53 mmol, 1.0 equiv) and (cod)PdMeCl (150 mg, 0.58 mmol, 1.1 equiv) in a 20-mL Schlenk tube was added DCM (5.0 mL) at room temperature and the reaction mixture was stirred for 1 hour at ambient temperature. The mixture was filtrated through a syringe filter and the solvent was removed *in vacuo*. The crude material was dissolved into DCM (ca. 5.0 mL) and reprecipitated from DCM/pentane to give the title product in 94% (320 mg, 0.50 mmol) yield as a green powder.

^{1}H NMR ($CDCl_3$, 500 MHz) δ: 8.52 (s, 1H), 7.64–7.61 (m, 1H), 7.53–7.42 (m, 4H), 7.37 (dd, *J* = 7.6, 7.6 Hz, 1H), 7.08 (dd, *J* = 7.5, 7.5 Hz, 1H), 6.94 (dd, *J* = 7.9, 5.2 Hz, 1H), 6.84 (dd, *J* = 7.5, 7.5 Hz, 1H), 6.76 (dd, *J* = 8.2, 3.7 Hz, 1H), 6.67 (dd, *J* = 10.2, 8.1 Hz, 1H), 3.71 (s, 3H), 3.43 (s, 3H), 1.43 (d, *J* = 14.3 Hz, 9H), 1.18 (d, *J* = 14.0 Hz, 9H), 0.31 (d, *J* = 3.1 Hz, 3H); ^{13}C{^{1}H}NMR ($CDCl_3$, 126 MHz) δ: 160.7 (s), 160.6 (s), 140.7 (d, *J* = 15 Hz), 136.9 (dd, *J* = 10, 2 Hz), 136.8 (dd, *J* = 44 Hz, 5 Hz), 133.8 (d, *J* = 4 Hz), 133.7 (dd, *J* = 74 Hz, 15 Hz), 133.5 (s), 132.6 (s), 131.0 (dd, *J* = 13, 10 Hz), 129.7 (dd, *J* = 6, 3 Hz), 128.2 (dd, *J* = 12, 2 Hz), 121.1 (d, *J* = 14 Hz), 120.5 (d, *J* = 8 Hz), 117.7 (d, *J* = 49 Hz), 117.1 (d, *J* = 52 Hz), 111.1 (d, *J* = 5 Hz), 110.9 (d, *J* = 4 Hz), 55.6 (s), 54.8 (s), 38.1 (d, *J* = 57 Hz), 37.4 (d, *J* = 58 Hz), 28.0 (s, 3C), 27.2 (s, 3C), −1.5 (s); ^{31}P{^{1}H} NMR ($CDCl_3$, 162 MHz) δ: 63.8 (s), 27.1 (s); HRMS (ESI-TOF) Calcd for $C_{29}H_{39}O_3P_2Pd$ $[M-Cl]^+$ 603.1409; found, 603.1430.

{(κ^2-*P,O*)-{2-[Bis(2-trifluoromethylphenyl)phosphino]phenyl}di-*tert*-butylphosphine oxide}-(methyl)(chlorido)palladium;

{(κ^2-*P,O*)-{2-[(2-$CF_3C_6H_4)_2$P]C_6H_4}P(O)^tBu_2}PdMeCl (3d)

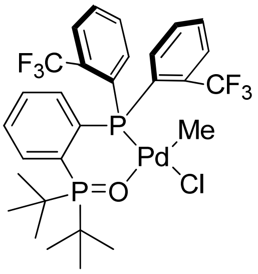

To a mixture of **S3** (280 mg, 0.50 mmol, 1.0 equiv) and (cod)PdMeCl (140 mg, 0.52 mmol, 1.0 equiv) in a 20-mL Schlenk tube was added chloroform (4.0 mL) at room temperature and the reaction mixture was stirred for 10 hours at 60 °C. The reaction mixture was filtrated through a syringe filter and the solvent was removed *in vacuo*. The crude material was dissolved into DCM (ca. 5.0 mL) and recrystallised from DCM/pentane to give the title product in 86% yield (326 mg, 0.43 mmol) as a 2:1 DCM adduct.

^{1}H NMR ($CDCl_3$, 400 MHz) δ: 10.53 (br, 1H), 7.87–7.84 (br, 2H), 7.74–7.68 (m, 4H), 7.63–7.59 (m, 2H), 7.53 (dd, *J* = 7.1, 7.1 Hz, 1H), 7.44 (dd, *J* = 7.6, 7.6 Hz, 1H), 6.90 (br, 1H), 1.50 (br, 9H), 0.88 (br, 9H), 0.38 (br, 3H); ^{13}C{^{1}H} NMR ($CDCl_3$, 126 MHz) δ: 148.0 (br), 137.0 (s), 134.9, 134.0, 133.4, 132.7, 132.6, 132.3, 131.7, 131.6, 131.2, 130.6, 129.8, 129.7, 129.1, 128.4, 126.3, 125.5, 124.2, 123.2, 122.0, (Note: The signals in the aromatic region could not be fully assigned, due to complex J-couplings.) 38.3 (d, *J* = 55 Hz), 37.6 (d, *J* = 60 Hz), 27.8 (s, 3C), 26.8 (s, 3C), −1.0 (s); ^{31}P{^{1}H} NMR ($CDCl_3$, 202 MHz) 65.3 (s), 57.1 (s); ^{19}F NMR ($CDCl_3$, 470 MHz) δ: −52.2 (d, *J* = 23 Hz), −55.7 (s); Anal. Calcd for $C_{29}H_{33}ClF_6OP_2Pd\cdot 0.5CH_2Cl_2$, C, 46.75; H, 4.52. found: C, 46.38; H, 4.55.

{{(κ^2-*P,O*)-[2-(Diisopropyl)phenyl]di-*tert*-butylphosphine oxide}(2,6-lutidine)(methyl)palla-dium}{tetrakis[3,5-bis(trifluoromethyl)phenyl]borate};

{{(κ^2-*P,O*)-[2-(^{i}Pr_2P)C_6H_4]P(O)^tBu_2}PdMe(2,6-lutidine)}$^+$BAr$^F{}_4{}^-$ (1a)

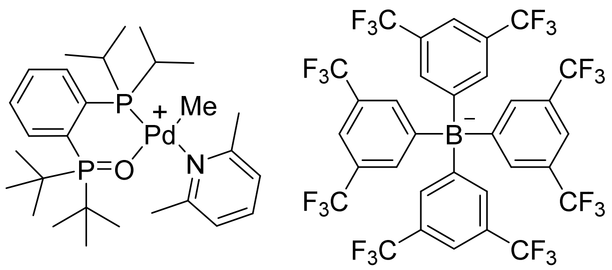

To a mixture of {{(κ^2-*P,O*)-[2-(^{i}Pr_2P)C_6H_4]P(O)^tBu_2}PdMe(2,6-lutidine)}$^+$$SbF_6^-$ (75 mg, 92 µmol,

1.0 equiv) and NaBAr^F$_4$ (82 mg, 92 μmol, 1.0 equiv) in a 20-mL Schlenk tube was added DCM (2.0 mL) and the mixture was stirred for 30 minutes at room temperature. Then the reaction mixture was filtered through a pad of Celite to remove the formed sodium salt and the DCM was removed *in vacuo* to give the title product in 94% yield (130 mg, 86 μmol).

^{1}H NMR (CDCl$_3$, 500 MHz) δ: 7.97–7.95 (m, 1H), 7.71–7.59 (m, 12H), 7.51 (s, 4H), 7.17 (d, *J* = 7.8 Hz, 2H), 3.09 (s, 6H), 2.62 (doublet of septet, *J* = 7.1, 7.1 Hz, 2H), 1.29 (d, *J* = 6.9 Hz, 6H), 1.26 (dd, *J* = 7.1, 2.5 Hz, 6H), 1.16 (d, *J* = 14.4 Hz, 18H), 0.37 (d, *J* = 2.3 Hz, 3H); ^{13}C NMR (CDCl$_3$, 126 MHz) δ: 162.2–161.5 (m, 4C), 158.2 (s, 2C), 139.1 (s), 134.9 (s, 8C), 134.6 (d, *J* = 8 Hz), 134.3 (d, *J* = 11 Hz), 132.9 (dd, *J* = 30, 4 Hz), 132.6 (dd, *J* = 13, 8 Hz), 131.8 (dd, *J* = 6, 3 Hz), 130.0 (dd, *J* = 12, 2 Hz), 129.0 (qq, *J* = 31, 3 Hz, 8C), 124.7 (q, *J* = 272 Hz, 8C), 123.2 (d, *J* = 3 Hz, 2C), 117.6 (sep, *J* = 4 Hz, 4C), 37.9 (d, *J* = 58 Hz, 2C), 28.5 (d, *J* = 25 Hz, 2C), 27.4 (s, 6C), 26.5 (s, 2C), 19.8 (d, *J* = 5 Hz, 2C), 19.1 (s, 2C), −8.0 (s); ^{31}P{^{1}H} NMR (CDCl$_3$, 202 MHz) δ: 57.6 (d, *J* = 7.5 Hz), 44.1 (d, *J* = 7.5 Hz); ^{19}F NMR (CDCl$_3$, 470 MHz) δ: −64.2 (s); HRMS (ESI-TOF) *m/z* calcd for $C_{21}H_{39}OP_2Pd$ [M−BAr^F$_4$−C_7H_9N] 475.1511; found 475.1491.

{{(*κ*^{2}-*P,O*)-[2-(Diphenylphosphino)phenyl]di-*tert*-butylphosphine oxide}(2,6-lutidine)(methyl)-palladium}{tetrakis[3,5-bis(trifluoromethyl)phenyl]borate};
{{(*κ*^{2}-*P,O*)-[2-(Ph_2P)C_6H_4]P(O)^{t}Bu$_2$}PdMe(2,6-lutidine)}$^+$BAr^F$_4$$^-$ (1b)

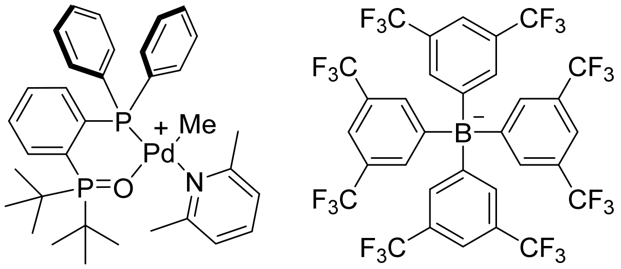

{{(*κ*^{2}-*P,O*)-[2-(Ph_2P)C_6H_4]P(O)^{t}Bu$_2$}PdMe(2,6-lutidine)}$^+$$SbF_6^-$ (44 mg, 50 μmol, 1.0 equiv) and NaBAr^F$_4$ (44 mg, 50 μmol, 1.0 equiv) were added into a 20-mL Schlenk tube and DCM (1.5 mL) was then added. The resulting mixture was stirred for 30 minutes at room temperature. Then the reaction mixture was filtered through a pad of Celite to remove the formed sodium salt and the DCM was removed *in vacuo* to give the title product in 92% yield (69 mg, 46 μmol).

^{1}H NMR (CDCl$_3$, 500 MHz) δ: 7.72–7.40 (m, 27H), 7.18 (d, *J* = 7.8 Hz, 2H), 3.10 (s, 6H), 1.03 (1d, *J* = 14.4 Hz, 18H), 0.16 (d, *J* = 3.0 Hz, 3H).^{13}C{^{1}H} NMR (CDCl$_3$, 126 MHz) δ: 162.2–161.5 (m, 4C), 158.4 (s, 2C), 139.2 (s), 138.0 (dd, *J* = 8, 3 Hz), 134.9 (s, 8C), 134.1 (s, 2C), 134.0 (s, 2C), 133.9 (dd, *J* = 42, 3 Hz), 133.4 (dd, *J* = 72, 15 Hz), 132.9 (dd, *J* = 13, 9 Hz), 132.2 (dd, *J* = 6, 3 Hz), 131.9 (d, *J* = 3 Hz, 2C), 130.3 (dd, *J* = 12, 2 Hz), 129.6–128.7 (m, 14C), 124.7 (q, *J* = 272 Hz, 8C), 123.3 (d, *J* = 4 Hz, 2C), 117.6 (sep, *J* = 4 Hz, 4C), 37.7 (d, *J* = 58 Hz, 2C), 27.0 (s, 6C), 26.7 (s, 2C),

−2.0 (s); $^{31}P\{^1H\}$ NMR ($CDCl_3$, 202 MHz) δ: 61.6 (d, *J* = 5 Hz), 35.0 (d, *J* = 5 Hz); ^{19}F NMR ($CDCl_3$, 470 MHz) δ: −62.4 (s); HRMS (ESI-TOF) *m/z* calcd for $C_{27}H_{35}OP_2Pd$ [M–BAr^F_4–C_7H_9N] 543.1198; found 543.1190.

{{(*κ*²-*P,O*)-{2-[bis(2-methoxyphenyl)phosphino]phenyl}di-*tert*-butylphosphine oxide}-(2,6-lutidine)(methyl)palladium}{tetrakis[3,5-bis(trifluoromethyl)phenyl]borate}; {{(*κ*²-*P,O*)-{2-[(2-OMeC_6H_4)$_2$P]C_6H_4}P(O)^{t}Bu$_2$}PdMe(2,6-lutidine)}$^+$$BAr^F_4$$^-$ (1c)

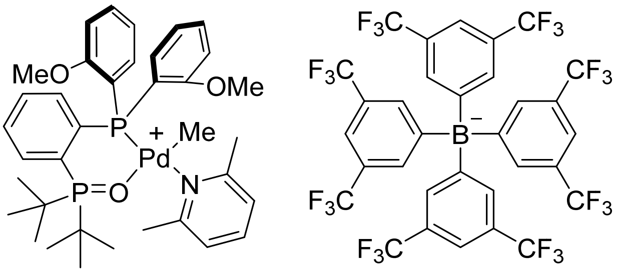

To a solution of **3c** (160 mg, 0.26 mmol, 1.0 equiv) and 2,6-lutidine (45 µL. 0.38 mmol, 1.5 equiv) in DCM (6.0 mL) was added $NaBAr^F_4$ (230 mg, 0.26 mmol, 1.0 equiv) at ambient temperature and the reaction mixture was stirred for 50 minutes at ambient temperature. Then the reaction mixture was filtrated through a pad of Celite to remove the formed sodium salt and DCM was removed *in vacuo*. The crude material was dissolved into DCM (ca. 5.0 mL) again and reprecipitated from DCM/pentane to give the title product in 88% (350 mg, 0.22 mmol) yield.

^1H NMR ($CDCl_3$, 500 MHz) δ: 8.26 (dd, *J* = 15.1, 6.9 Hz, 1H), 7.71 (s, 8H), 7.60–7.53 (m, 10H), 7.47 (dd, *J* = 7.6, 7.6 Hz, 1H), 7.18 (dd, *J* = 7.9, 7.9 Hz, 1H), 7.14–7.11 (m, 2H), 7.03 (dd, *J* = 7.9, 5.5 Hz, 1H), 6.92 (dd, *J* = 7.5, 7.5 Hz, 1H), 6.86 (dd, *J* = 8.2, 4.0 Hz, 1H), 6.65 (dd, *J* = 11.1, 8.1 Hz, 1H), 3.74 (s, 3H), 3.48 (s, 3H), 3.15 (s, 3H), 3.02 (s, 3H), 1.03 (d, *J* = 14.3 Hz, 9H), 0.98 (d, *J* = 14.6 Hz, 9H), –0.01 (d, *J* = 3.4 Hz, 3H); $^{13}C\{^1H\}$ NMR ($CDCl_3$, 126 MHz) δ: 162.5–161.3 (m, 4C), 160.6 (s), 160.1 (d, *J* = 6 Hz), 158.8 (two peaks, 2C), 139.1 (d, *J* = 18 Hz), 138.9 (s), 137.2 (dd, *J* = 9, 3 Hz), 134.9 (s, 8C), 134.7 (d, *J* = 2 Hz), 134.6 (dd, *J* = 47 Hz, 4 Hz), 134.3 (d, *J* = 4 Hz), 133.7 (s), 132.1 (dd, *J* = 74, 15 Hz), 131.6 (dd, *J* = 13, 10 Hz), 130.7 (dd, *J* = 7, 3 Hz), 129.4 (dd, *J* = 13 Hz, 2 Hz), 129.0 (qq, *J* = 30 Hz, 3 Hz, 8C), 124.7 (q, *J* = 273 Hz, 8C), 123.0 (three peaks, 2C), 121.3 (d, *J* = 15 Hz), 121.1 (d, *J* = 8 Hz), 117.6 (sep, *J* = 4 Hz, 4C), 115.7 (d, *J* = 51 Hz), 115.3 (d, *J* = 56 Hz), 111.6 (d, *J* = 4 Hz), 111.1 (d, *J* = 5 Hz), 55.3 (s), 54.9 (s), 37.8 (d, *J* = 57 Hz), 37.2 (d, *J* = 58 Hz), 27.3 (s, 3C), 26.7 (s, 3C), 26.5 (s), 26.3 (s), −2.1 (d, *J* = 2 Hz); $^{31}P\{^1H\}$ NMR ($CDCl_3$) δ: 63.1 (s), 25.9 (s); ^{19}F NMR ($CDCl_3$, 376 MHz) δ: −62.4 (s); Anal. Calcd for $C_{68}H_{60}BF_{24}NO_3P_2Pd$, C, 51.88; H, 3.84; N, 0.89. found: C, 51.69; H, 3.89; N, 0.71.

{{(κ^2-*P,O*)-{2-[bis(2-trifluoromethylphenyl)phosphino]phenyl}di-*tert*-butylphosphine oxide}-(2,6-lutidine)(methyl)palladium}{tetrakis[3,5-bis(trifluoromethyl)phenyl]borate}; {{(κ^2-*P,O*)-{2-[(2-$CF_3C_6H_4)_2$P]C_6H_4}P(O)^tBu_2}PdMe(2,6-lutidine)}$^+$BAr$^F{}_4{}^-$ (1d)

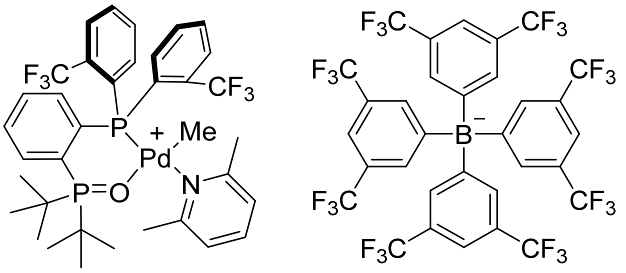

To a solution of **3d** (36 mg, 50 μmol, 1.0 equiv) and 2,6-lutidine (6.4 μL, 55 μmol, 1.1 equiv) in DCM (1.0 mL) was added NaBAr$^F{}_4$ (44 mg, 50 μmol, 1.0 equiv) at ambient temperature and the reaction mixture was stirred for 6 hours at ambient temperature. Then the reaction mixture was filtered through a pad of Celite to remove the formed sodium salt and DCM was removed *in vacuo*. The crude material was dissolved into DCM (ca. 1.5 mL) again and reprecipitated from DCM/pentane to give the title product in 67% yield (55 mg, 33 μmol).

^{1}H NMR ($CDCl_3$, 400 MHz) δ: 10.01 (br s, 1H), 7.93 (dd, *J* = 6.9, 4.6 Hz, 1H), 7.82 (dd, *J* = 7.7 Hz, 7.7 Hz, 2H), 7.69–7.62 (m, 15H), 7.53 (d, *J* = 7.3 Hz, 1H), 7.51 (s, 4H), 7.17 (d, *J* = 6.6 Hz, 2H), 6.89 (dd, *J* = 13.3, 7.7 Hz, 1H), 3.15 (s, 3H), 2.97 (s, 3H), 0.97 (d, *J* = 14.4 Hz, 9H), 0.80 (d, *J* = 14.9 Hz, 9H), 0.07 (d, *J* = 2.7 Hz, 3H); ^{13}C{^{1}H} NMR ($CDCl_3$, 126 MHz) δ: 162.5–161.3 (m, 4C), 159.0 (s), 158.8 (s), 145.4 (br s), 139.2 (s), 137.9 (s), 135.5 (s), 134.9 (s), 133.5, 133.3, 133.0, 132.9, 132.8, 132.7, 132.6, 132.4, 132.2, 132.1, 132.0, 131.6 (d, *J* = 6 Hz), 130.9 (dd, *J* = 12, 2 Hz), 129.4 (m), 129.1 (qq, *J* = 31, 3 Hz, 8C), 128.3, 125.7, 124.8, 124.7 (q, *J* = 272 Hz, 8C), 124.5, 123.8, 123.5 (two peaks), 121.6, 121.3, 117.6 (sep, *J* = 4 Hz, 4C), (Note: The signals in the aromatic region could not be fully assigned, due to complex J-couplings.) 38.0 (d, *J* = 56 Hz), 37.4 (d, *J* = 58 Hz), 27.2 (s, 3C), 26.9 (s), 26.4 (s), 26.1 (s, 3C), −2.5 (s); ^{31}P{^{1}H} NMR ($CDCl_3$, 162 MHz) δ: 65.0 (s), 55.5 (br s); ^{19}F NMR ($CDCl_3$, 376 MHz) δ: −51.7 (s), −56.0 (s), −62.4 (s); Anal. Calcd for $C_{68}H_{54}BF_{30}NOP_2Pd$, C, 49.49; H, 3.30; N, 0.85. found: C, 49.40; H, 3.40; N, 0.80.

{{(κ^2-*P,O*)-{2-[bis(2-methoxyphenyl)phosphino]phenyl}di-*tert*-butylphosphine oxide}palladium [(κ^2-*C,O*)-(2-acetanilido)]}{tetrakis[3,5-bis(trifluoromethyl)phenyl]borate}; {{(κ^2-*P,O*)-{2-[(2-OMeC_6H_4)$_2$P]C_6H_4}P(O)^{t}Bu$_2$}Pd[(κ^2-*C,O*)-(2-acetanilido)]}$^+$BAr$^F{}_4{}^-$ (2c)

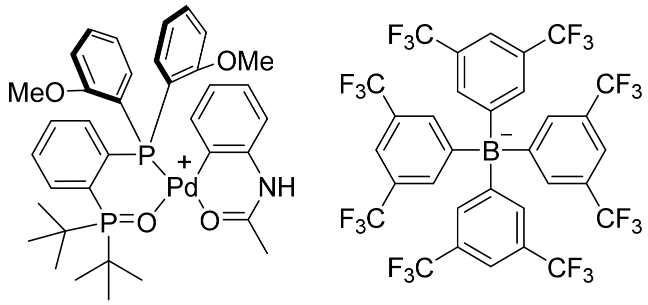

S2 (97 mg, 0.20 mmol, 1.0 equiv) and di-μ-chloride-di[κ^2-(2-acetanilido)palladium] (55 mg, 0.10 mmol, 0.50 equiv) were added into Schlenk tube and DCM (3.5 mL) was then added. The resulting mixture was stirred for 2 hours at room temperature. Then the reaction mixture was transferred to another Schlenk tube containing silver hexafluoroantimonate (69 mg, 0.20 mmol, 1.0 equiv) at −78 °C. Then the dry ice bath was removed and the resulting mixture was stirred for 2 hours at room temperature. Then the reaction mixture was filtered through a pad of Celite to remove the formed silver salt and the DCM was removed *in vacuo*. The crude material was dissolved into DCM again (ca. 4.0 mL) and reprecipitated from DCM/toluene to give the {{(κ^2-*P,O*)-{2-[(2-OMeC_6H_4)$_2$P]C_6H_4}P(O)^{t}Bu$_2$}Pd[(κ^2-*C,O*)-(2-acetanilido)]}$^+$Sb$F_6{}^-$ in 82% yield (160 mg, 0.16 mmol) as a green powder. ^{1}H NMR (CDCl_3, 400 MHz) δ: 9.36 (1H, s), 7.67–7.39 (7H, m), 7.20 (1H, dd, *J* = 7.2, 7.2 Hz), 6.90 (2H, m), 6.78–6.67 (3H, m), 6.56 (1H, br s), 6.20 (1H, dd, *J* = 7.6, 7.6 Hz), 6.07 (1H, dd, *J* = 7.5, 7.5 Hz), 3.40 (3H, s), 3.31 (3H, s), 2.42 (3H, s), 1.41 (9H, d, *J* = 14.1 Hz), 1.10 (9H, d, *J* = 12.7 Hz); ^{31}P{^{1}H} NMR (CDCl_3, 162 MHz) δ: 68.1 (s), 36.5 (br s).

Next, {{(κ^2-*P,O*)-{2-[(2-OMeC_6H_4)$_2$P]C_6H_4}P(O)^{t}Bu$_2$}Pd[(κ^2-*C,O*)-(2-acetanilido)]}$^+$Sb$F_6{}^-$ (150 mg, 0.15 mmol, 1.0 equiv) and NaBAr$^F{}_4$ (130 mg, 0.15 mmol, 1.0 equiv) were added into a 20-mL Schlenk tube and DCM (4.0 mL) was then added. The resulting mixture was stirred for 1 hour at room temperature. Then the reaction mixture was filtered through a pad of Celite to remove the formed sodium salt and the DCM was removed *in vacuo*. The crude material was dissolved into DCM (ca. 3.0 mL) again and reprecipitated from DCM/pentane to give the title product in 91% yield (210 mg, 0.13 mmol).

^{1}H NMR (CDCl_3, 500 MHz).δ: 8.78 (br s, 1H), 8.02 (s, 1H), 7.72 (s, 8H), 7.66–7.53 (m, 8H), 7.47–7.40 (m, 2H), 7.22 (dd, *J* = 7.3 Hz, 7.3 Hz, 1H), 6.94–6.93 (m, 2H), 6.73–6.72 (m, 2H), 6.57 (br, 1H), 6.50 (d, *J* = 7.9 Hz, 1H), 6.28 (dd, *J* = 7.8 Hz, 7.8 Hz, 1H), 6.16 (dd, *J* = 7.5 Hz, 7.5 Hz, 1H), 3.33 (s, 3H), 3.25 (s, 3H), 2.33 (s, 3H), 1.36 (d, *J* = 14.3 Hz, 9H), 1.08 (d, *J* = 14.6 Hz, 9H); ^{13}C{^{1}H} NMR (CDCl_3, 126 MHz) δ: 169.4 (d, *J* = 4 Hz), 162.5–161.3 (m, 4C), 161.2 (s), 160.7 (d, *J* = 5 Hz), 140.6 (s), 139.4 (d, *J* = 13 Hz), 137.1 (dd, *J* = 9, 5 Hz), 135.5 (s), 135.2 (d, *J* = 2 Hz),

134.9 (s, 8C), 134.4 [(s), (dd, J = 54, 5 Hz), total 2C], 132.3 (s), 131.4 (dd, J = 13 Hz, 10 Hz), 131.1 (dd, J = 75 Hz, 15 Hz), 131.0 (dd, J = 8, 2 Hz), 129.6 (dd, J = 11 Hz, 2 Hz), 129.0 (qq, J = 32, 3 Hz, 8C), 127.9 (s), 125.0 (s), 124.7 (q, J = 273 Hz, 8C), 124.2 (d, J = 6 Hz), 121.9 (d, J = 15 Hz), 121.0 (d, J = 9 Hz), 117.6 (sep, J = 4 Hz, 4C), 115.3 (s), 114.5 (d, J = 55 Hz), 114.0 (d, J = 62 Hz), 111.8 (d, J = 5 Hz), 111.7 (d, J = 5 Hz), 55.2 (s), 54.8 (s), 37.7 (d, J = 53 Hz), 37.3 (d, J = 55 Hz), 27.2 (s, 3C), 26.6 (s, 3C), 22.4 (d, J = 5 Hz); $^{31}P\{^1H\}$ NMR ($CDCl_3$, 202 MHz) δ: 67.6 (s), 31.4 (br s); ^{19}F NMR ($CDCl_3$, 376 MHz) δ: −62.3 (s).; Anal. Calcd for $C_{68}H_{56}BF_{24}NO_4P_2Pd$, C, 51.49; H, 3.56; N, 0.88. found: C, 51.30; H, 3.67; N, 0.78.

{{(κ^2-*P,O*)-{2-[bis(2-trifluoromethylphenyl)phosphino]phenyl}di-*tert*-butylphosphine oxide}-palladium[(κ^2-*C,O*)-(2-acetanilido)]}{tetrakis[3,5-bis(trifluoromethyl)phenyl]borate}; {{(κ^2-*P,O*)-{2-[(2-$CF_3C_6H_4)_2$P]C_6H_4}P(O)^tBu_2}Pd[(κ^2-*C,O*)-(2-acetanilido)]}$^+$$BAr^F{}_4{}^-$ (2d)

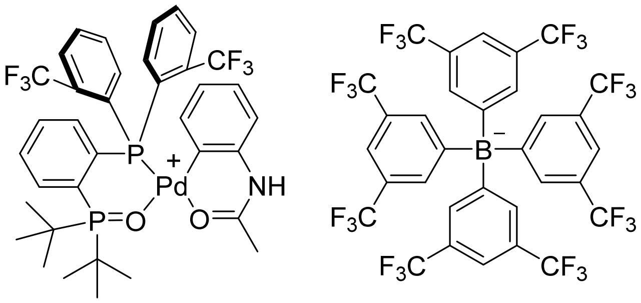

S3 (160 mg, 0.30 mmol, 1.0 equiv) and di-μ-chloride-di[κ^2-(2-acetanilido)palladium] (83 mg, 0.15 mmol, 0.50 equiv) were added into a 20-mL Schlenk tube and DCM (6.0 mL) was then added. The resulting mixture was stirred for 3 hours at room temperature. Then the reaction mixture was transferred to another 20-mL Schlenk tube containing $AgSbF_6$ (110 mg, 0.33 mmol, 1.1 equiv) at −78 °C. Then the dry ice bath was removed and the resulting mixture was stirred for 3 hours at ambient temperature. The reaction mixture was filtered through a pad of Celite to remove the formed silver salt. The crude material was dissolved into DCM (ca. 3.0 mL) and reprecipitated from DCM/toluene to give the {{(κ^2-*P,O*)-{2-[(2-$CF_3C_6H_4)_2$P]C_6H_4}P(O)^tBu_2}Pd[(κ^2-*C,O*)-(2-acetanilido)]}$^+$$SbF_6{}^-$ in 87% yield as a 1:1 toluene adduct. Toluene can be removed from product by dissolution in CH_2Cl_2 followed by evaporation of the solution. Two isomers A and B were observed in a ratio of ca. 6:1 at ambient temperature. ^1H NMR ($CDCl_3$, 400 MHz) δ: 10.50 [dd, J = 19.3, 7.4 Hz, (A, B), 1H], {[9.33 (s, A)], [9.20 (s, B)], total 1H}, {[8.10 (br s, B)], [8.04 (dd, J = 5.7, 5.7 Hz, A)], total 1H}, 7.89 [(br, (A, B), 2H], 7.77–7.49 [(m, (A, B), 7H], 7.39 [dd, J = 5.7, 5.7 Hz, (A, B), 1H], {[7.11 (dd, J = 12.9, 8.1 Hz), A], [6.94 (br s, B)], total 1H}, 6.85 [d, J = 7.9 Hz, 1H, (A, B)], 6.73 [(dd, J = 6.5, 6.5 Hz, 1H, (A, B)], 6.04–5.86 (m, 2H), 2.41 [(3H, s, (A, B)], {[1.54 (d, J = 14.5 Hz, B)], [1.46 (d, J = 14.5 Hz, A), total 9H]}, {[0.92 (d, J = 14.8 Hz, A)], [0.82 (d, J = 14.9 Hz, B)], total 9H}; $^{31}P\{^1H\}$ NMR ($CDCl_3$, 202 MHz) δ: 69.2 (s, B), 69.0 (s, A), 61.5 (q, J = 24 Hz, A), 39.7

(s, B); ^{19}F NMR ($CDCl_3$, 376 MHz) δ: −51.8 (s, B), −52.5 (d, *J* = 23 Hz, A), −55.7 (s).

Next, {{(*κ*^{2}-*P,O*)-{2-[(2-$CF_3C_6H_4)_2$P]C_6H_4}P(O)^{t}Bu$_2$}Pd[(*κ*^{2}-*C,O*)-(2-acetanilido)]}$^+$SbF_6^- (83 mg, 80 μmol, 1.0 equiv) and NaBAr$^F_4$ (71 mg, 80 μmol, 1.0 equiv) were added into the Schlenk tube and DCM (2.0 mL) was then added. The resulting mixture was stirred for 1.5 hours at room temperature. The mixture was filtered through a pad of Celite to remove the formed sodium salt and DCM was removed *in vacuo*. The crude material was dissolved into DCM (ca. 3.0 mL) again and reprecipitated from DCM/pentane to give the title product in 87% yield (120 mg, 69 μmol).

Two isomers A and B were observed in a ratio of ca. 5:1 at ambient temperature (The coalescence of signals was observed upon heating. See Figure S29). ^{1}H NMR ($CDCl_3$, 500 MHz) δ: 10.45 (dd, *J* = 19.8, 7.7 Hz, 1H), 8.09 [(s, (A, B), 1H], 8.03 [dd, *J* = 7.6, 7.6 Hz, (A, B), 1H], 7.86–7.83 (m, 2H), 7.70–7.65 (m, 11H), 7.59–7.57 (m, 2H), 7.52–7.50 (m, 5H), 7.37 [dd, *J* = 6.9, 5.5 Hz, 1H, (A, B)], {[7.12 (dd, *J* = 13.1, 8.0 Hz, A)], [6.94 (dd, *J* = 12.8, 8.2 Hz, B)], total 1H}, {[6.82 (dd, *J* = 7.1, 7.1 Hz, B)], [6.75 (dd, *J* = 7.3, 7.3 Hz, A)], total 1H}, {[6.57 (d, *J* = 6.9 Hz, B)], [6.54 (d, *J* = 7.8 Hz, A)], total 1H}, {[6.12 (dd, *J* = 7.7, 7.7 Hz)], [6.07 (dd, *J* = 7.6 Hz)], [5.93 (dd, *J* = 7.6, 7.6 Hz, B)], total 2H}, {[2.36 (s, B)], [2.34 (s, A)], total 3H}, {[1.50 (d, *J* = 14.4 Hz, B)], [1.41 (d, *J* = 14.4 Hz, A)], total 9H}, {[0.89 (d, *J* = 14.9 Hz, A)], [0.78 (d, *J* = 14.7 Hz, B)], total 9H}; ^{13}C{^{1}H} NMR ($CDCl_3$, 126 MHz) δ: 169.9 (d, *J* = 4 Hz), 169.1 (s), 162.5–161.3 (m), 146.6, 146.3, 139.4 (d, *J* = 12 Hz), 139.0, 138.4, 137.8, 136.8 (d, *J* = 5 Hz), 136.3, 135.9, 134.9, 134.0, 133.9 (two peaks), 133.7, 133.6, 133.5, 133.3, 133.0, 132.8, 132.7, 132.6 (two peaks), 132.4 (br), 132.3, 132.2, 132.1, 131.8 (three peaks), 131.7 (two peaks), 131.6 (s), 131.4, 131.3 (two peaks), 131.2 (two peaks), 131.1, 131.0, 130.9, 130.5, 130.1 (m), 129.5 (two peaks), 129.4 (four peaks), 129.0 (qq, *J* = 32, 3 Hz), 126.5, 126.2, 125.8, 125.6, 124.9, 124.7 (q, *J* = 272 Hz), 124.3 (d, *J* = 5 Hz), 124.0, 123.7, 122.7, 121.5, 120.5, 119.3, 117.6 (sep, *J* = 4 Hz), 116.7 (s), (Note: The signals in the aromatic region could not be fully assigned, due to complex J-couplings and the presence of rotamers.) {38.3 [d, *J* = 54 Hz, A], 37.9 [d, *J* = 58 Hz, A], 37.8 [d, *J* = 56 Hz, B], 37.7 [d, *J* = 52 Hz, B], total 2C}, {[27.3 (s, B)], [27.2 (s, A)], total 3C}, {[26.5 (s, B)], [26.4 (s, A), total 3C]}, 22.2 (d, *J* = 4 Hz); ^{31}P{^{1}H} NMR ($C_2D_2Cl_4$, 202 MHz) δ: 69.0 (s, B), 68.9 (s, A), 61.1 (q, *J* = 25 Hz, A), 39.3 (br, B); ^{19}F NMR ($CDCl_3$, 376 MHz) δ: −52.5 (s, B), −52.5 (d, *J* = 23 Hz, A), −55.9 [s, (A, B)], −62.3 (s); HRMS (ESI-TOF) *m/z* Calcd for $C_{36}H_{38}F_6NO_2P_2Pd$ [M–BAr$^F_4$]$^+$ 798.1317, found 798.1315.

{{(κ^2-*P,O*)-{2-[bis(2-trifluoromethylphenyl)phosphino]phenyl}diisopropylphosphine oxide}-palladium[(κ^2-*C,O*)-(2-acetanilido)]}{tetrakis[3,5-bis(trifluoromethyl)phenyl]borate}; {{(κ^2-*P,O*)-{2-[(2-$CF_3C_6H_4)_2P]C_6H_4$}P(O)^iPr_2}Pd[(κ^2-*C,O*)-(2-acetanilido)]}$^+$BAr$^F_4$$^-$ (2e)

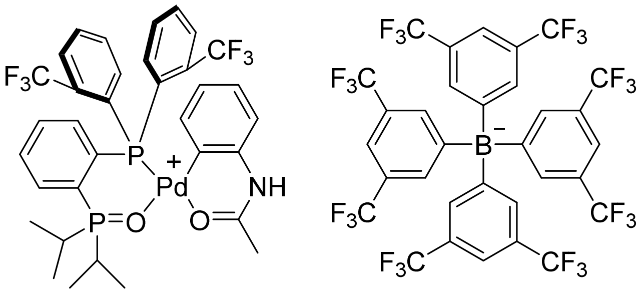

S5 (27 mg, 51 µmol, 1.0 equiv) and di-μ-chloride-di[κ^2-(2-acetanilido)palladium] (14 mg, 25 µmol, 0.50 equiv) were added into a 20-mL Schlenk tube and DCM (1.0 mL) was then added. The resulting mixture was stirred for 4.5 hours at room temperature. Then the reaction mixture was transferred to another 20-mL Schlenk tube containing NaBAr$^F_4$ (45 mg, 50 µmol, 1.0 equiv) at −78 °C. Then the dry ice bath was removed and the resulting mixture was stirred for 17.5 hours at ambient temperature. The reaction mixture was filtered through a syringe filter to remove the sodium salt and DCM was removed *in vacuo*. The crude material was purified by column chromatography on silica gel using DCM (100%) as the eluent and recrystallised from DCM/hexane to afford the desired product in 64% yield (52 mg, 32 µmol).

^{1}H NMR ($CDCl_3$, 500 MHz) δ: 10.04 (br, 1H), 8.13 (s, 1H), 7.93 (br, 1H), 7.81 (br, 2H), 7.72 (t, *J* = 2.1 Hz, 8H), 7.67–7.49 (m, 10H), 7.46–7.41 (m, 1H), 7.11 (dd, *J* = 12.8, 8.2 Hz, 1H), 6.80 (dd, *J* = 7.5 Hz, 7.5 Hz, 1H), 6.57 (d, *J* = 7.3 Hz, 1H), 6.13 (dd, *J* = 7.3 Hz, 7.3 Hz, 1H), 6.08 (dd, *J* = 7.5 Hz, 7.5 Hz, 1H), 2.35 (s, 3H), 2.31 (dqq, *J* = 6.1 Hz, 6.1 Hz, 6.1 Hz, 1H), 2.23 (dqq, *J* = 11.0 Hz, 6.7 Hz, 6.7 Hz, 1H), 1.22 (br, 3H), 1.01 (br, 3H), 0.92 (d, *J* = 15.9 Hz, 3H), 0.71 (br, 3H); ^{13}C NMR ($CDCl_3$, 126 MHz) δ: 169.4 (s), 162.5–161.3 (m, 4C), 145.2 (br s), 139.3 (d, *J* = 12 Hz), 137.0 (s), 136.9 (br s), 134.9 (s, 8C), 133.8 (s), 133.3 (d, *J* = 16 Hz), 132.8 (d, *J* = 3 Hz), 132.5 (s), 132.2–132.1 (m), 131.7 (d, *J* = 8 Hz), 131.6 (dd, *J* = 11 Hz, 11 Hz), 131.1 (dd, *J* = 80, 16 Hz), 129.9 (br s), 129.6 (br s), 129.0 (qq, *J* = 32 Hz, 3 Hz, 8C), 125.8 (s), 125.2 (br s), 125.0 (q, *J* = 263 Hz), 124.7 (q, *J* = 273 Hz, 8C), 124.4 (d, *J* = 5 Hz), 123.8 (q, 276 Hz), 117.6 (sep, *J* = 4 Hz, 4C), 116.8 (s), (Note: The signals in the aromatic region could not be fully assigned, due to complex J-couplings.) 27.3 (d, *J* = 66 Hz), 27.1 (d, *J* = 65 Hz), 22.2 (d, *J* = 5 Hz), 16.0 (d, *J* = 2 Hz), 15.7 (d, *J* = 4 Hz), 14.9 (d, *J* = 3 Hz), 14.3 (s); ^{31}P{^{1}H} NMR ($CDCl_3$, 202 MHz) δ: 69.4 (s), 59.1 (br s); ^{19}F NMR ($CDCl_3$, 376 MHz) δ: −52.5 (d, *J* = 23 Hz), −55.3 (s), −62.3 (s); HRMS (ESI-TOF) *m/z* Calcd for $C_{34}H_{34}F_6NO_2P_2Pd$ [M–BAr$^F_4$]$^+$ 770.1004, found 770.0994.

{{(κ^2-*P,O*)-{2-[bis(2-trifluoromethylphenyl)phosphino]phenyl}dimethylphosphine oxide}-palladium[(κ^2-*C,O*)-(2-acetanilido)]}{tetrakis[3,5-bis(trifluoromethyl)phenyl]borate}; {{(κ^2-*P,O*)-{2-[(2-$CF_3C_6H_4)_2$P]C_6H_4}P(O)Me_2}Pd[(κ^2-*C,O*)-(2-acetanilido)]}$^+$BAr$^F{}_4{}^-$ (2f)

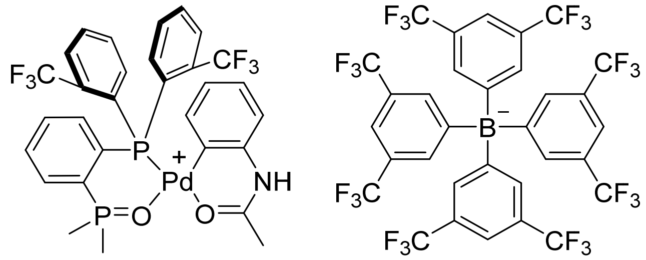

S6 (19 mg, 40 µmol, 1.0 equiv) and di-μ-chloride-di[κ^2-(2-acetanilido)palladium] (11 mg, 20 µmol, 0.50 equiv) were added into a 20-mL Schlenk tube and DCM (0.80 mL) was then added. The resulting mixture was stirred for 19 hours at room temperature. Then the reaction mixture was transferred to another 20-mL Schlenk tube containing NaBAr$^F{}_4$ (35 mg, 40 µmol, 1.0 equiv) at −78 °C. Then the dry ice bath was removed and the resulting mixture was stirred for 4 hours at ambient temperature. The reaction mixture was filtered through a syringe filter to remove the formed sodium salt and DCM was removed *in vacuo*. The crude material was purified by column chromatography on silica gel using DCM (100%) as the eluent and recrystallised from DCM/pentane to afford the desired product in 58% yield (37 mg, 23 µmol).

^{1}H NMR ($CDCl_3$, 400 MHz) δ: 9.01 (dd, *J* = 16.6, 6.2 Hz, 1H), 8.08 (s, 1H), 7.87–7.83 (m, 3H), 7.70 (s, 10H), 7.53–7.45 (m, 9H), 6.98 (dd, *J* = 12.8, 7.8 Hz, 1H), 6.87 (dd, *J* = 7.4, 7.4 Hz, 1H), 6.62 (d, *J* = 7.6 Hz, 1H), 6.21 (dd, *J* = 7.6, 7.6 Hz, 1H), 6.14 (dd, *J* = 8.0, 8.0 Hz, 1H), 2.35 (s, 3H), 1.67 (d, *J* = 13.1 Hz, 3H), 1.43 (d, *J* = 13.2 Hz, 3H); ^{13}C{^{1}H} NMR ($CDCl_3$, 126 MHz) δ: 169.2 (d, *J* = 4 Hz), 162.4–161.2 (m, 4C), 142.2 (d, *J* = 24 Hz), 139.1 (d, *J* = 14 Hz), 136.5 (s), 135.7 (d, *J* = 7 Hz), 134.9 (s, 8C), 134.7, 134.6, 134.1, 133.9, 133.8 (two peaks), 133.7, 133.3, 133.2, 132.9 (d, *J* = 2 Hz), 132.6–132.4 (m), 132.3 (s), 132.1 (two peaks), 132.0 (two peaks), 131.9 (s), 129.8–129.7 (m), 129.0 (qq, *J* = 32 Hz, 4 Hz, 8C), 128.4, 126.1, 125.1, 124.8, 124.7, 124.7 (q, *J* = 273 Hz, 8C), 124.4, 124.1, 122.6, 121.9, 117.6 (sep, *J* = 4 Hz, 4C), 117.2, (Note: The signals in the aromatic region could not be fully assigned, due to complex J-couplings.) 22.2 (d, *J* = 5 Hz), 17.9 (d, *J* = 21 Hz), 17.3 (d, *J* = 18 Hz); ^{31}P{^{1}H} NMR ($CDCl_3$, 202 MHz) δ: 55.6 (d, *J* = 8 Hz), 52.2 (br s); ^{19}F NMR ($CDCl_3$, 376 MHz) δ: −52.8 (d, *J* = 23 Hz), −54.7 (s), −62.3 (s); HRMS (ESI-TOF) *m/z* Calcd for $C_{30}H_{26}F_6NO_2P_2Pd$ [M–BAr$^F{}_4$]$^+$ 714.0378, found 714.0398.

{{(κ^2-*P*,*O*)-[2-(diisopropylphosphino)phenyl]di-*tert*-butylphosphine oxide}palladium-[(κ^2-*C*,*O*)-3-methoxy-2-methyl-3-oxopropyl]}(hexafluorophosphate);
{{(κ^2-*P*,*O*)-[2-(^{i}Pr$_2$P)C$_6$H$_4$]P(O)^{t}Bu$_2$]}Pd[(κ^2-*C*,*O*)-CH$_2$CH(Me)C(O)OMe]}$^+$PF$_6^-$ (4a)
{{(κ^2-*P*,*O*)-[2-(diisopropylphosphino)phenyl]di-*tert*-butylphosphine oxide}palladium-[(κ^2-*C*,*O*)-1-ethyl-2-methoxy-2-oxoethyl]}(hexafluorophosphate);
{{(κ^2-*P*,*O*)-[2-(^{i}Pr$_2$P)C$_6$H$_4$]P(O)^{t}Bu$_2$]}Pd[(κ^2-*C*,*O*)-CH(Et)C(O)OMe]}$^+$PF$_6^-$ (5a)

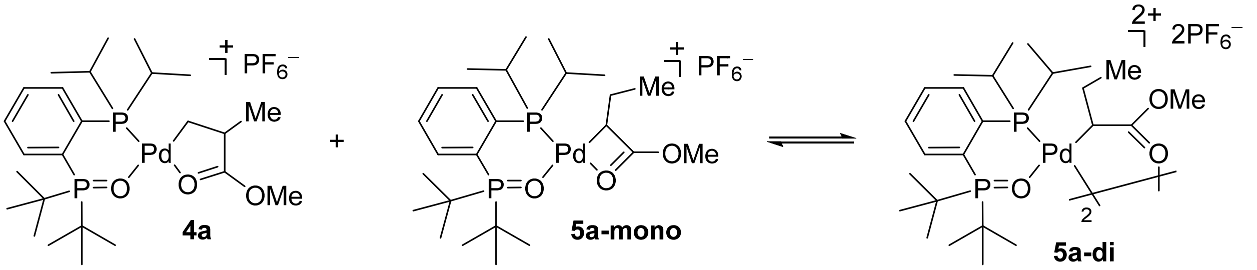

{(κ^2-*P*,*O*)-[2-(^{i}Pr$_2$P)C$_6$H$_4$]P(O)^{t}Bu$_2$}Pd(Me)(Cl) (150 mg, 0.30 mmol, 1.0 equiv) and silver hexafluorophosphate (110 mg, 0.45 mmol, 1.5 equiv) were added into a 20-mL Schlenk tube. Then a mixture of benzotrifluoride (6.8 mL) and methyl acrylate (81 μL, 0.90 mmol, 3.0 equiv) was added via syringe. The resulting mixture was stirred for 3 hours at room temperature. The mixture was filtered to separate yellow precipitates and the filtrate was added dropwise to the flask containing a mixture of diethyl ether and pentante (1:1) (*Note*: Evaporation of the filtrate resulted in the decomposition of product). The resulting white precipitates were collected and dried under vacuum to afford **4a** in 16% yield (35 mg, 0.049 mmol). The filtered yellow precipitate was dissolved into DCM and filtered again to remove silver salt. The resulting yellow solution was evaporated and reprecipitated from DCM/benzotrifluoride to give yellow powder. The yellow powder was dried under vacuum to afford **5a** in 52% yield (111 mg, 0.16 mmol).

To determine the NMR yield, {(κ^2-*P*,*O*)-[2-(^{i}Pr$_2$P)C$_6$H$_4$]P(O)^{t}Bu$_2$}PdMeCl (13 mg, 27 μmol, 1.0 equiv), silver hexafluorophosphate (10 mg, 40 μmol, 1.5 equiv), and the mixture of DCM (0.60 mL) and methyl acrylate (7.2 μL, 80 μmol, 3.0 equiv) were added into a 20-mL Schlenk tube. After stirring 1 hour at room temperature, NMR yields of **4a** and **5a** were determined to be 29% and 71% yield respectively by ^{31}P NMR measurement using triphenylphosphine in a capillary tube as an external standard.

{{(κ^2-*P*,*O*)-[2-(^{i}Pr$_2$P)C$_6$H$_4$]P(O)^{t}Bu$_2$]}Pd[(κ^2-*C*,*O*)-*C*H$_2$CH(Me)C(*O*)OMe]}$^+$PF$_6^-$ (4a)

^{1}H NMR (C$_2$D$_2$Cl$_4$, 500 MHz) δ: 7.91–7.87 (m, 1H), 7.78–7.72 (m, 3H), 3.95 (s, 3H), 2.98 (ddq, *J* = 7.1 Hz, 7.1 Hz, 7.1 Hz, 1H), 2.46 (dqq, *J* = 9.5 Hz, 7.3 Hz, 7.3 Hz, 1H), 2.38 (dqq, *J* = 7.3 Hz, 7.3 Hz, 7.3 Hz, 1H), 1.61 (ddd, *J* = 9.5 Hz, 6.8 Hz, 2.4 Hz, 1H), 1.44 (d, *J* = 14.6 Hz, 9H), 1.36–1.21 (m, 22H), 1.12 (dd, *J* = 18.0, 7.0 Hz, 3H); ^{13}C{^{1}H} NMR (C$_2$D$_2$Cl$_4$, 126 MHz) δ: 190.1 (s), 134.7 (dd, *J* = 72, 11 Hz), 133.5 (d, *J* = 9 Hz), 132.5 (dd, *J* = 13, 8 Hz), 131.6 (dd, *J* = 6 Hz, 3 Hz), 131.4

(dd, *J* = 35 Hz, 4 Hz) 130.2 (dd, *J* = 12, 2 Hz), 55.0 (s), 44.6 (s), 38.0 (d, *J* = 57 Hz), 37.3 (d, *J* = 55 Hz), 27.9 (d, *J* = 28 Hz), 27.4 (s, 6C), 27.4 (d, *J* = 28 Hz), 19.4 (s), 19.4 (d, *J* = 8 Hz), 19.4 (d, *J* = 4 Hz), 19.2 (s), 19.1 (d, *J* = 4 Hz), 18.7 (d, *J* = 3 Hz); $^{31}P\{^{1}H\}$ NMR ($C_2D_2Cl_4$, 202 MHz) δ: 63.2 (d, *J* = 5 Hz), 55.8 (d, *J* = 5 Hz), −144.6 (sep, *J* = 713 Hz); ^{19}F NMR ($CDCl_3$, 376 MHz) δ: −73.7 (d, *J* = 717 Hz); HRMS (ESI-TOF) *m/z* Calcd for $C_{25}H_{45}O_3P_2Pd$ $[M{-}PF_6]^+$ 561.1879, found 561.1889. Single crystals suitable for X-ray diffraction analysis were grown from vapor diffusion of diethyl ether into the solution of **4a** in benzotrifluoride.

{{(*κ*²-*P*,*O*)-[2-(*ⁱ*Pr₂P)C₆H₄]P(O)*ᵗ*Bu₂]}Pd[(*κ*²-*C*,*O*)-*C*H(Et)C(*O*)OMe]}⁺PF₆⁻ (5a)

There exists equilibrium between a monomer (**5a-mono**) and its dimer state (**5a-di**). The ratio of **5a-mono**:**5a-di** is 35:65 when the concentration is 3.0 × 10^{-2} M at 25 °C. ^{1}H NMR ($C_2D_2Cl_4$, 500 MHz, 3.0 × 10^{-2}, 5 °C) δ: 7.98–7.76 (m, 4H), {[3.95 (s, **5a-di**)], [3.84 (s, **5a-mono**)], total 3H}, 2.83–1.62 (m, 5H), 1.48–1.15 (m, 30H), {[0.98 (br, **5a-mono**)], [0.92 (dd, *J* = 7.0, 7.0 Hz, **5a-di**)], total 3H};$^{13}C\{^{1}H\}$ NMR ($C_2D_2Cl_4$, 126 MHz) δ: 183.6 (s), 133.0–130.5 (m), 53.7, 53.5, 37.9, 37.7, 37.4, 37.3, 28.9, 28.7, 27.9, 27.7, 27.3, 24.2 (br), 23.7, 19.2, 18.9, 18.7, 18.3, 18.0, 14.5, 14.4 (Note: The signals could not be fully assigned, due to the presence of equilibrium between monomer **5a-mono** and dimer **5a-di**.); $^{31}P\{^{1}H\}$ NMR ($C_2D_2Cl_4$, 202 MHz, 3.0 × 10^{-2} M) δ: 65.1 (s, **5a-mono**), 65.0 (s, **5a-di**), 57.1 (s, **5a-mono**) 55.5 (s, **5a-di**), −144.6 (sep, *J* = 713 Hz); ^{19}F NMR ($C_2D_2Cl_4$, 470 MHz, 3.0 × 10^{-2} M) δ: −72.5 (d, *J* = 714 Hz); HRMS (ESI-TOF) *m/z* Calcd for $C_{25}H_{45}F_6O_3P_3Pd$ $[M{-}PF_6]^+$ 561.1879, found 561.1890.

{{(*κ*²-*P*,*O*)-[2-(diisopropylphosphino)phenyl]di-*tert*-butylphosphine oxide}palladium-(1-methoxycarbonylpropyl)(pyridine)}(hexafluorophosphate); {{(*κ*²-*P*,*O*)-[2-(*ⁱ*Pr₂P)C₆H₄]P(O)*ᵗ*Bu₂]}Pd[CH(CO₂Me)CH₂CH₃](pyridine)}⁺PF₆⁻ (5a-py)

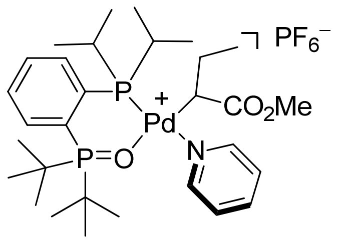

Pyridine (4.0 μL, 50 μmol, 1.1 equiv) was added into a solution of complex **5a** (32 mg, 45 μmol, 1.0 equiv) in dichloromethane (1.5 mL). The resulting mixture was stirred for 2 hours at room temperature. The solvent was removed *in vacuo* and reprecipitation from dichloromethane/pentane gave the title product in 81% (29 mg) yield.

^{1}H NMR (CD_2Cl_2, 500 MHz) δ: 8.68 (d, *J* = 4.3 Hz, 2H), 8.03 (ddd, *J* = 7.7, 7.7, 3.2 Hz, 1H), 7.95 (t, *J* = 7.6 Hz, 1H), 7.78 (dd, *J* = 6.6, 6.6 Hz, 1H), 7.72–7.67 (m, 2H), 7.53 (t, *J* = 6.4 Hz, 2H), 3.54 (s, 3H), 3.07 (dqq, *J* = 6.1, 6.1, 6.1 Hz, 1H), 2.72 (dd, *J* = 9.3, 9.3 Hz, 1H), 2.65 (dqq, *J* = 10.4 Hz,

7.0Hz, 7.0 Hz, 1H), 1.60–1.51 (m, 1H), 1.51–1.42 (m, 1H), 1.46 (dd, *J* = 13.7, 7.0 Hz, 3H), 1.38 (dd, *J* = 16.5, 6.7 Hz, 6H), 1.21–1.13 (m, 3H), 1.17 (d, *J* = 14.3 Hz, 9H), 1.11 (d, *J* = 14.6 Hz, 9H), 0.78 (dd, *J* = 7.2 Hz, 7.2 Hz, 3H); $^{13}C\{^1H\}$ NMR (CD_2Cl_2, 126 MHz) δ: 177.6 (s), 150.5 (s, 2C), 139.6 (s), 134.7 (d, *J* = 9 Hz), 133.9 (dd, *J* = 74, 10 Hz), 133.3 (dd, *J* = 13, 7 Hz), 132.6 (dd, *J* = 30, 3 Hz), 132.5 (dd, *J* = 5, 3 Hz), 130.7 (dd, *J* = 12, 3 Hz), 125.6 (s, 2C), 51.2 (s), 38.4 (d, *J* = 59 Hz), 37.4 (d, *J* = 55 Hz), 29.2 (d, *J* = 25 Hz), 27.6 (s, 3C), 27.3 (s, 3C) , 27.0 (d, *J* = 25 Hz), 26.1 (s), 25.2 (s), 20.1 (d, *J* = 2 Hz), 19.8 (d, *J* = 3 Hz), 19.2 (d, *J* = 4 Hz), 18.5 (d, *J* = 4 Hz), 14.5 (d, *J* = 2 Hz); ^{31}P NMR (CD_2Cl_2, 202 MHz) δ: 60.9 (s), 44.6 (s), −144.5 (sep, *J* = 710 Hz); ^{19}F NMR ($CDCl_3$, 376 MHz) δ: −73.4 (d, *J* = 717 Hz); HRMS (ESI-TOF) *m/z* Calcd for $C_{25}H_{45}O_3P_2Pd$ $[M-PF_6-C_5H_5N]^+$ 561.1879, found 561.1896. Single crystals suitable for X-ray diffraction analysis were grown from vapor diffusion of pentane into the solution of **5a-py** in DCM at −40 °C.

{{(*κ*²-*P,O*)-{2-[bis(2-methoxyphenyl)phosphino]phenyl}di-*tert*-butylphosphine oxide}-palladium[(*κ*²-*C,O*)-1-ethyl-2-methoxy-2-oxoethyl]}(hexafluorophosphate); {{(*κ*²-*P,O*)- {2-[$(2\text{-}OMeC_6H_4)_2P]C_6H_4\}P(O)^tBu_2\}Pd[(\kappa^2\text{-}C,O)\text{-}CH(Et)C(O)OMe]\}^+PF_6^-$ (5c)

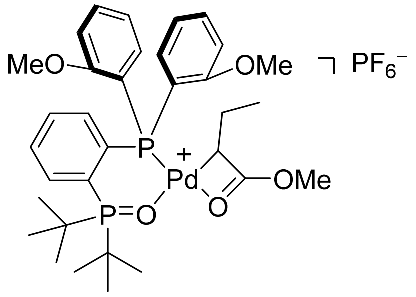

To a mixture of **3c** (64 mg, 0.10 mmol, 1.0 equiv) and silver hexafluorophosphate (38 mg, 0.15 mmol, 1.5 equiv) in a 20-mL Schlenk tube were added a mixture of DCM (2.3 mL) and methyl acrylate (27 μL, 0.30 mmol, 3.0 equiv) was added via a syringe. The resulting mixture was stirred for 4 hours at room temperature. The mixture was filtered through a syringe filter to remove the formed silver salts. The crude material was then purified by reprecipitation from DCM/pentane to give the yellow powder. To remove the residual pentane, the resulting yellow powder was dissolved into DCM and DCM was removed *in vacuo* to give the title product in 72% yield (60 mg, 0.072 mmol).

Two rotamers A and B existed with the ratio of ca. 3:1. Integral ratios of each proton signal are described assuming that only rotamers A or B exist in the system. Measured integration of proton signals can be calculated according to the following equation: (Measured intensity = described intensity × ratio of target rotamer). ^1H NMR ($C_2D_2Cl_4$, 500 MHz, 5 °C) δ: 9.08 (dd, *J* = 17.2, 7.8 Hz, B, 1H), 8.97 (dd, *J* = 17.4, 7.3 Hz, A, 1H), 7.73–7.71 [m, (A, B), 1H], 7.62–7.55 (m, 5H), 7.19 [dd, *J* = 7.3, 7.3 Hz, (A, B), 1H], 7.16–7.10 (m, 2H), 7.02 [dd, *J* = 13.3, 7.5 Hz, (A, B), 1H], 6.85–6.80

[m, (A, B), 1H], 3.77 [s, (A,B), 3H], 3.61 (s, B, 3H), 3.40 (s, B, 3H), 3.38 (s, A, 3H), 3.33 [(A, 3H) + (B, 1H)], 2.34 (br, A, 1H), 2.00 (br, A, 1H), 1.67 (ddd *J* = 10.0, 5.0, 5.0 Hz, A, 1H), 1.40 {[(d, *J* = 14.0 Hz, (A, B), 9H] + (B, 1H)}, 1.27–1.24 (B, 1H), 0.89 (dd, *J* = 7.2, 7.2 Hz, A, 3H), 0.72 [d, *J* = 15.0 Hz, (A, B), 9H], 0.31 (dd, *J* = 6.7, 6.7 Hz, B, 3H); $^{13}C\{^1H\}$ NMR ($C_2D_2Cl_4$, 126 MHz, 5 °C) δ: {[186.8 (s, B)], [183.5 (s, A)], total 1C}, {[161.0 (d, *J* = 2 Hz, A)], [160.9 (d, *J* = 3 Hz, B)], total 1C}, {[159.8 (d, *J* = 4 Hz, B)], [159.7 (d, *J* = 3 Hz, A)], total 1C}, {[141.6 (d, *J* = 24 Hz, A)], [141.1 (d, *J* = 22 Hz, B)], total 1C}, {[137.0–136.9 (m, B)], [136.7 (dd, *J* = 9, 5 Hz, A)], total 1C}, {[136.4 (s, A)], [136.3 (s, B)], total 1C}, 135.3 [s, (A, B)], {[135.1 (d, *J* = 5 Hz, A)], [134.1 (d, *J* = 5 Hz, B)], total 1C}, {[132.3 (dd, *J* = 57, 4 Hz, A)], [131.7 (dd, *J* = 58, 4 Hz, B)], total 1C}, 131.2 [dd, *J* = 12 Hz, 12 Hz, (A, B)], 130.7 [d, *J* = 8 Hz, (A, B)], 130.3 [d, *J* = 13 Hz, (A, B)], 130.2 [dd, *J* = 74, 15 Hz, (A, B)], {[121.8 (d, *J* = 10 Hz, B)], [121.6 (d, *J* = 11 Hz, A)], [121.5 (d, *J* = 17 Hz, B)], [120.9 (d, *J* = 15 Hz, A)], total 2C}, {[112.8 (d, *J* = 57 Hz, B)], [112.5 (d, *J* = 57 Hz, A)], total 1C}, 112.4 [d, *J* = 3 Hz, (A, B)], {[112.1 (d, *J* = 65 Hz, A)], [111.1 (d, *J* = 64 Hz, B)], total 1C}, {[111.6 (d, *J* = 5 Hz, B)], [111.1 (d, *J* = 5 Hz, A)], total 1C}, {[56.2 (s, A)], [56.1 (s, B)], total 1C}, {[55.2 (s, B)], [55.1 (s, A)], total 1C}, {[53.5 (s, B)], [52.8 (s, A)], total 1C}, 37.7 [d, *J* = 53 Hz, (A, B), four peaks], {[37.5 (d, *J* = 59 Hz, B)], [37.3 (d, *J* = 59 Hz, A)], total 1C}, {[35.6 (s, A)], [33.1 (s, B)], total 1C}, 27.0 [s, (A, B), 3C, two peaks], 26.7 [s, (A, B), 3C, two peaks], 24.4 [s, (A, B)], {[15.0 (d, *J* = 3 Hz, A)], [14.7 (d, *J* = 6 Hz, B)], total 1C}; $^{31}P\{^1H\}$ NMR ($C_2D_2Cl_4$, 202 MHz, 5 °C) δ: 67.4 (s, B), 66.7 (s, A), 37.4 (s, A), 34.5 (s, B), −144.6 (sep, *J* = 715 Hz); ^{19}F NMR ($C_2D_2Cl_4$, 470 MHz, 5 °C) δ: −72.3 (d, *J* = 714 Hz); HRMS (ESI-TOF) Calcd for $C_{33}H_{45}O_5P_2Pd$ $[M{-}PF_6]^+$ 689.1777, found 689.1805. Single crystals of **5c-di** suitable for X-ray diffraction analysis were obtained according to the following procedure. Compound **5c** was dissolved into acetonitrile and then acetonitrile was removed *in vacuo*. The residue was dissolved into $CHCl_3$ and single crystals were grown from vapor diffusion of $CHCl_3$ into toluene.

{{(κ^2-*P*,*O*)-{2-[bis(2-methoxyphenyl)phosphino]phenyl}di-*tert*-butylphosphine oxide}-palladium(1-methoxycarbonylpropyl)(pyridine)}(hexafluorophosphate);
{{(κ^2-*P*,*O*)-{2-[(2-OMeC$_6$H$_4$)$_2$P]C$_6$H$_4$}P(O)^{t}Bu$_2$}Pd[CH(CO$_2$Me)CH$_2$CH$_3$](pyridine)}$^+$PF$_6^-$ (5c-py)

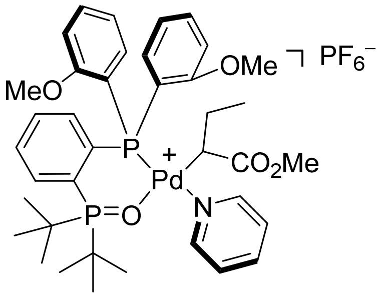

To a mixture of **3c** (26 mg, 40 µmol, 1.0 equiv) and silverhexafluorophosphate (15 mg, 60 µmol, 1.5 equiv) in a 20-mL Schlenk tube was added a mixture of DCM (0.9 mL) and methyl acrylate (11 µL, 120 µmol, 3.0 equiv) via a syringe. The resulting mixture was stirred for 3 hours at ambient temperature. Then the mixture of DCM (0.6 mL) and pyridine (3.4 µL, 1.1 equiv) was added to the Schlenk tube at ambient temperature and the suspension was stirred for 30 minutes at ambient temperature. The mixture was filtrated through a syringe filter to remove the formed silver salts. Volatile materials were removed *in vacuo* and 1,1,2,2-tetrachloroethane (13 µL, 20 mg) was added to the crude material as an internal standard. NMR yield was determined to be 90% yield and the crude material was purified by reprecipitation from DCM/pentane to give the title product in 85% (31 mg, 34 µmol) yield.

Two rotamers A and B were observed in a ratio of ca. 10:3. ^{1}H NMR (CDCl$_3$, 500 MHz, 5 °C) δ: {[9.32 (dd, *J* = 17.2, 6.6 Hz, A)], [9.13 (dd, *J* = 15.9, 7.6 Hz, B)], total 1H}, {[8.77 (dd, *J* = 4.0, 1.5 Hz, A)], [8.72 (s, B)], total 2H}, 7.95 [dd, *J* = 7.6 Hz, 7.6 Hz, (A, B), 1H], 7.73–7.70 [m, (A+B), 1H], 7.62–7.55 (m, 6H), 7.51 [dd, *J* = 7.3 Hz, 7.3 Hz, (A, B), 1H], 7.31 [dd, *J* = 7.2 Hz, 7.2 Hz, (A, B), 1H], 7.08–6.95 (m, 3H), {[6.87 (dd, *J* = 8.5, 3.7 Hz, B)], [6.84 (dd, *J* = 8.4, 3.5 Hz, A)], total 1H}, {[3.83 (s, B)], [3.70 (s, A)], total 3H}, {[3.43 (s, B)], [3.41 (s, A)], total 3H}, {[3.30 (s, A)], [3.26 (s, B)], total 3H}, {[1.74 (ddd, *J* = 10.7, 10.7 Hz, 3.0 Hz, A)], [1.63–1.55 (m), B], total 1H}, 1.13–1.11 (10H, m), 0.83 [d, *J* = 14.6 Hz, (A, B), 9H], {[0.32 (dd, *J* = 7.2 Hz, 7.2 Hz, A)], [0.18 (dd, *J* = 6.9, 6.9 Hz, B)], total 3H}; ^{13}C{^{1}H} NMR (CDCl$_3$, 126 MHz, 5 °C) δ: {[178.9 (s, B), 178.2 (d, *J* = 3 Hz, A)], total 1C}, 161.1 [d, *J* = 3 Hz, (A, B)], {[160.2 (d, *J* = 5 Hz, B)], [159.6 (d, *J* = 5 Hz, A)], total 1C}, 150.7 [s, (A+B), total 2C], {[143.1 (d, *J* = 27 Hz, A)], [141.7 (d, *J* = 25 Hz, B)], total 1C}, {[139.4 (s, B)], [139.3 (s, A)], total 1C}, {[137.2 (dd, *J* = 9, 4 Hz, A)], [137.0 (dd, *J* = 9, 3 Hz, B)], total 1C}, 135.4 [(d, *J* = 2Hz, A), (d, *J* = 2 Hz, B), four peaks], {{134.5 [d, *J* = 5 Hz, (A, B)]}, [134.0 (three peaks)], total 2C}, 133.1 [dd, *J* = 50, 4 Hz, (A, B)], {[131.2 (dd, *J* = 13, 10 Hz, B)], [131.1 (dd, *J* = 13, 10 Hz, A)], total 1C}, 130.8–130.0 (m, 2C), 129.6 [dd, *J* = 12, 2 Hz, (A, B)],

125.7 [(s, B), (d, *J* = 3Hz, A), three peaks, total 2C], {[121.6 (d, *J* = 15 Hz, B)], [121.2 (d, *J* = 9 Hz, A), (d, *J* = 11 Hz, B), three peaks], [121.1 (d, *J* = 15 Hz, A)], total 2C}, {[114.5 (d, *J* = 50 Hz, B), 114.4 (d, *J* = 51 Hz, A)], total 1C}, {[113.8 (d, *J* = 59 Hz, A)], [112.7 (d, *J* = 58 Hz, B)], total 1C}, {[111.9 (d, *J* = 4 Hz, B)], [111.6 (d, *J* = 4 Hz, A)], total 1C}, {[111.2 (d, *J* = 5 Hz, B)], [111.0 (d, *J* = 5 Hz, A)], total 1C}, {[56.0 (s, B)], [55.7 (s, A)], total 1C}, {[54.9 (s, B)], [54.8 (s, A)], total 1C}, 50.6 [s, (A, B), two peaks], 37.5 [d, *J* = 45 Hz, (A, B)], {[37.1 (d, *J* = 58 Hz, B)], [37.0 (d, *J* = 49 Hz, A)], total 1C}, {[33.5 (s, B)], [32.0 (s, A)], total 1C}, 26.7 [s, (A, B), 3C], {[26.6, (s, B), 26.5 (s, A), total 3C], {[25.4 (s, B)], [23.1 (s, A)], total 1C}, {[14.4 (d, *J* = 5 Hz, B)], [14.3 (d, *J* = 5 Hz, A)], total 1C}; $^{31}P\{^1H\}$ NMR ($CDCl_3$, 202 MHz, 5 °C) δ: 65.7 (s, B), 65.3 (s, A), 32.3 (s, A), 29.8 (s, B), −144.4 (sep, *J* = 712 Hz); ^{19}F NMR ($CDCl_3$; 470 MHz, 5 °C) δ: −73.6 (sep, *J* = 713 Hz); HRMS (ESI-TOF) Calcd for $C_{33}H_{45}O_5P_2Pd$ $[M–PF_6–C_5H_5N]^+$ 689.1777, found 689.1743.

{{(*κ*^{2}-*P,O*)-{2-[bis(2-trifluoromethylphenyl)phosphino]phenyl}di-*tert*-butylphosphine oxide}-palladium(1-methoxycarbonylpropyl)(pyridine)}(hexafluorophosphate): {{(*κ*^{2}-*P,O*)-{2-[$(2\text{-}CF_3C_6H_4)_2P$]$C_6H_4P(O)^tBu_2$}Pd[$CH(CO_2Me)CH_2CH_3$](pyridine)}$^+PF_6^-$ (5d-py)

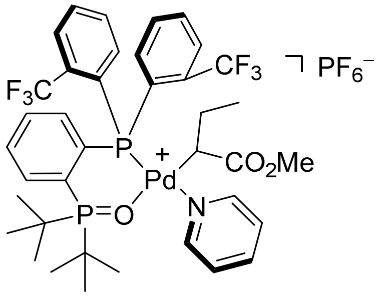

To a mixture of **3d** (20 mg, 27 μmol, 1.0 equiv) and silver hexafluorophosphate (10 mg, 40 μmol, 1.5 equiv) in a 20-mL Schlenk tube was added a mixture of DCM (0.7 mL) and methyl acrylate (7.2 μL, 80 μmol, 3.0 equiv) via a syringe. The resulting mixture was stirred for 2.5 hours at ambient temperature. The mixture was filtrated through a pad of Celite to remove the formed silver salts and the volatile materials were removed *in vacuo*. Then, a mixture of DCM (1.2 mL) and pyridine (2.4 μL, 30 μmol, 1.1 equiv) was added via a syringe and the resulting solution was stirred for 40 minutes at ambient temperature. The volatile materials were removed *in vacuo*. The crude material was dissolved into DCM (ca. 2.0 mL) and purified by reprecipitation from DCM/hextane to give the title product in 68% yield (18 mg, 18 μmol).

To determine the NMR yield, **5d-py** was directly synthesised from **3d**. A mixture of **3d** (28 mg, 40 μmol, 1.0 equiv) and silver hexafluorophosphate (15 mg, 60 μmol, 1.5 equiv) was added into a 20-mL Schlenk tube. Then a mixture of DCM (0.9 mL) and methyl acrylate (11 μL, 120 μmol, 3.0 equiv) was added via a syringe. The resulting mixture was stirred for 2 hours at ambient

temperature. Then the mixture of DCM (0.6 mL) and pyridine (3.4 μL, 1.1 equiv) was added to the Schlenk tube at ambient temperature and the suspension was stirred for 30 minutes at ambient temperature. The mixture was filtrated through a syringe filter to remove the formed silver salts. Volatile materials were removed *in vacuo* and dibromomethane (20.9 mg) was added to the crude material as an internal standard. NMR yield was determined to be 85% yield. Unfortunately, this direct two step synthesis resulted in formation of palladium black and **5d-py** decomposed during purification.

Two rotamers A and B were observed in a ratio of ca. 3:1. ^{1}H NMR ($C_2D_2Cl_4$, 500 MHz) δ: 10.37–10.34 (m, 1H), {[8.71–8.70 (m, B)], [8.65 (1H, d, *J* = 5.2 Hz, A)], total 2H}, 8.10 (br, 1H), 8.03–7.98 (m, 2H), 7.91–7.58 (m, 10H), 7.11–7.08 (m, 1H), {[3.38 (s, B)], [3.32 (s, A)], total 3H}, 1.76–1.67 (m, 1H), {[1.56 (br s, A), 1.43 (br s, B)], total 1H}, 1.27–1.17 (m, 1H), 1.04 (d, *J* = 14.3 Hz, 9H), 0.76 (d, *J* = 15.0 Hz, 9H), {[0.36 (dd, *J* = 7.2, 7.2 Hz)], [0.22 (br s)], total 3H};^{13}C{^{1}H} NMR ($CDCl_3$, 101 MHz, −50 °C) δ: 178.1, 177.3, 150.6, 149.8, 146.3, 146.0, 139.6, 137.1, 136.1 (two peaks), 135.1, 134.2, 133.9, 133.7, 133.1 (two peaks), 132.8 (two peaks), 132.7, 132.4, 132.1, 131.3, 131.2, 130.7, 130.6, 130.1, 129.6, 129.3, 129.1, 128.4, 126.4, 126.0, 125.9, 125.6, 125.2, 123.8, 123.6, 123.2, 123.1, 122.9, 122.7, 122.3, 121.3, 121.1, 120.1, 118.4, 51.1, 50.9, 37.8, 37.7, 37.4, 37.3, 36.4, 34.0, 32.6, 26.8, 26.5, 26.0, 25.7, 23.2, 14.3, 13.9 (Note: The signals could not be fully assigned, due to complex J-coupling and the presence of rotamers.); ^{31}P{^{1}H} NMR ($CDCl_3$, 202 MHz) δ: 67.1 (s), 67.0 (s), 54.9 (q, *J* = 22 Hz), 54.5–53.1 (br m), −144.2 (sep, *J* = 713 Hz); ^{19}F NMR ($CDCl_3$, 376 MHz) δ: −52.6 (d, *J* = 23 Hz), −52.9 (d, *J* = 23 Hz), −55.4 to −55.6 (m), −73.2 (d, *J* = 705 Hz); HRMS (ESI-TOF) Calcd for $C_{33}H_{39}F_6O_3P_2Pd$ $[M-PF_6-C_5H_5N]^+$ 765.1313, found 765.1302. Single crystals of **5d-py** suitable for X-ray diffraction analysis were grown from vapor diffusion of hexane into the solution of **5d-py** in THF.

1-3. General Procedure for Polymerisation

A Representative Procedure for Ethylene Polymerisation (Table 1, entry 1)

A 50 mL stainless steel autoclave was dried in an oven at 120 °C, and then allowed to cool inside the dry box. After cooling, an aliquot (1.0 mL) of a stock solution of catalyst **1a** (7.5 μmol) in CH_2Cl_2 (10 mL) was added. CH_2Cl_2 was removed *in vacuo*, after which toluene (15 mL) was added. The autoclave was then sealed, charged with ethylene (3.0 MPa), and stirred in an isothermal heating block at 100 °C for 1 hour. The reaction was quenched by addition of MeOH (ca. 20 mL). The mixture was filtered and washed with MeOH. The solids were dissolved in hot *o*-dichlorobenzene, reprecipitated in MeOH (ca. 50 mL), filtered, and dried under vacuum at 120 °C for 3 hours to afford 2.00 g polyethylene. The molecular weight and polydispersity were determined by size exclusion chromatography. The extent of branching in the polymer backbone was determined by quantitative $^{13}C\{^{1}H\}$ NMR spectroscopy.

A Representative Procedure for Copolymerisation of Ethylene and Polar Vinyl Monomers (Table 2, entry 3)

A 50 mL stainless steel autoclave was dried in an oven at 120 °C, and then allowed to cool inside the dry box. After cooling, **1c** (15.3 mg, 10 μmol), toluene (12.0 mL), and methyl acrylate (3.0 mL) were added to the autoclave. The autoclave was then sealed, charged with ethylene (3.0 MPa) and stirred in an isothermal heating block at 80 °C for 15 h. The reaction was quenched by addition of MeOH (ca. 10 mL). The mixture was filtered and washed with MeOH. The solids were dissolved in hot *o*-dichlorobenzene, reprecipitated in MeOH (ca. 50 mL), filtered, and dried under vacuum at 120 °C for 3 hours to afford 0.61 g poly(ethylene-*co*-methyl acrylate). The molecular weight and polydispersity were determined by size exclusion chromatography. The incorporation ratio of vinyl acetate and extent of branching in the polymer backbone were determined by quantitative $^{13}C\{^{1}H\}$ NMR spectroscopy.

1-4. Additional Data of Polymerisation

Table S1. Full data for the Copolymerisation of Ethylene and Methyl Acrylate in the Presence of Cationic BPMO-Pd Complexes[a]

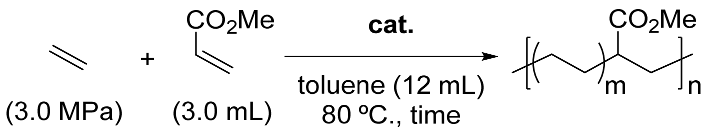

cat.[b]	time (h)	yield (g)	activity (kg mol^{-1} h^{-1})	M_n[c]	M_w/M_n	incorp.[d] (mol%)
**1a** (10)	15	0.03	0.2	1600	1.7	2.5
**1b** (10)	15	0	—	—	—	—
**1c** (10)	15	0.61	4.1	33000	2.3	2.3
**1d** (10)	15	0.89	5.9	14000	2.1	0.9
**2a** (10)	15	0.01	0.1	—	—	3.3[e]
**2c** (10)	15	0.40	2.7	24000	2.8	3.4
**2d** (10)	15	1.37	9.1	17000	2.6	1.3
**2e** (10)	15	2.95[f]	20[f]	19000	3.1	1.2
**2e** (0.75)	15	0.37	33	17000	3.6	0.9
**2e** (0.75)	1	0.03	41	18000	2.3	1.2[e]
**2f** (10)	15	4.05[f]	27[f]	8100	3.9	1.1
**2f** (0.75)	1	0.41	540	6900	2.9	0.5

[a] Conditions: ethylene, palladium catalyst, and MA were stirred in a 50 mL stainless autoclave at 80 °C. [b] Numbers in parenthesis are the amount of catalyst (μmol). [c] Determined by SEC analysis using polystyrene as an internal standard and calibrated by universal calibration. [d] Incorporation of MA determined by quantitative ^{13}C NMR analysis. [e] Determined by ^{1}H NMR analysis. [f] A saturated amount of copolymer in a stainless autoclave was obtained.

2. NMR Spectra of Ligands and Catalyst

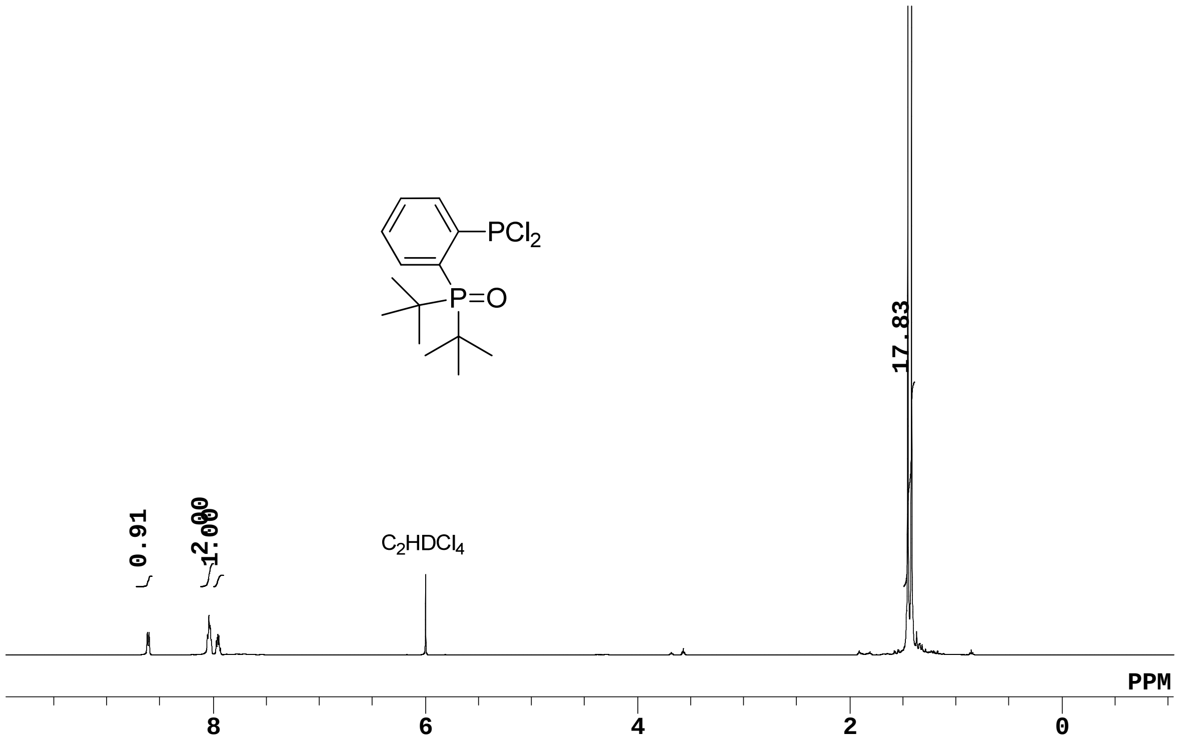

Figure S1. ^{1}H NMR spectrum ($C_2D_2Cl_4$, 500 MHz) of **S1**.

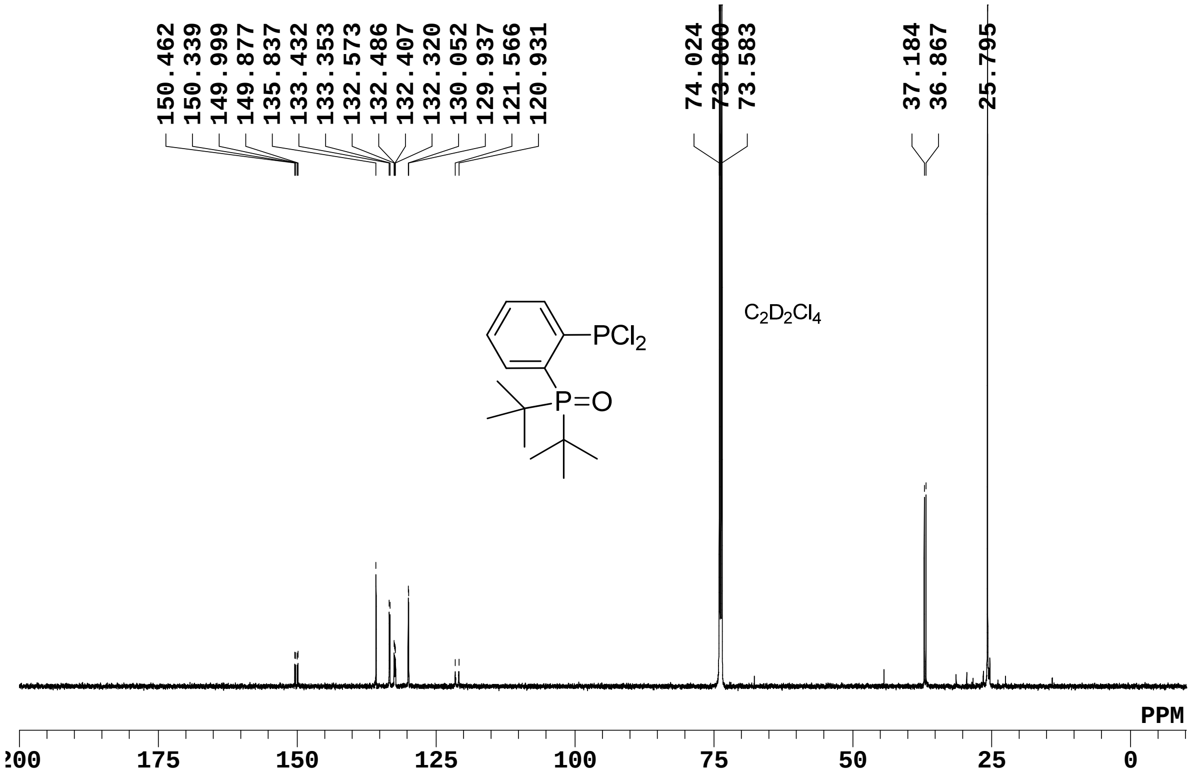

Figure S2. $^{13}C\{^{1}H\}$ NMR spectrum ($C_2D_2Cl_4$, 126 MHz) of **S1**.

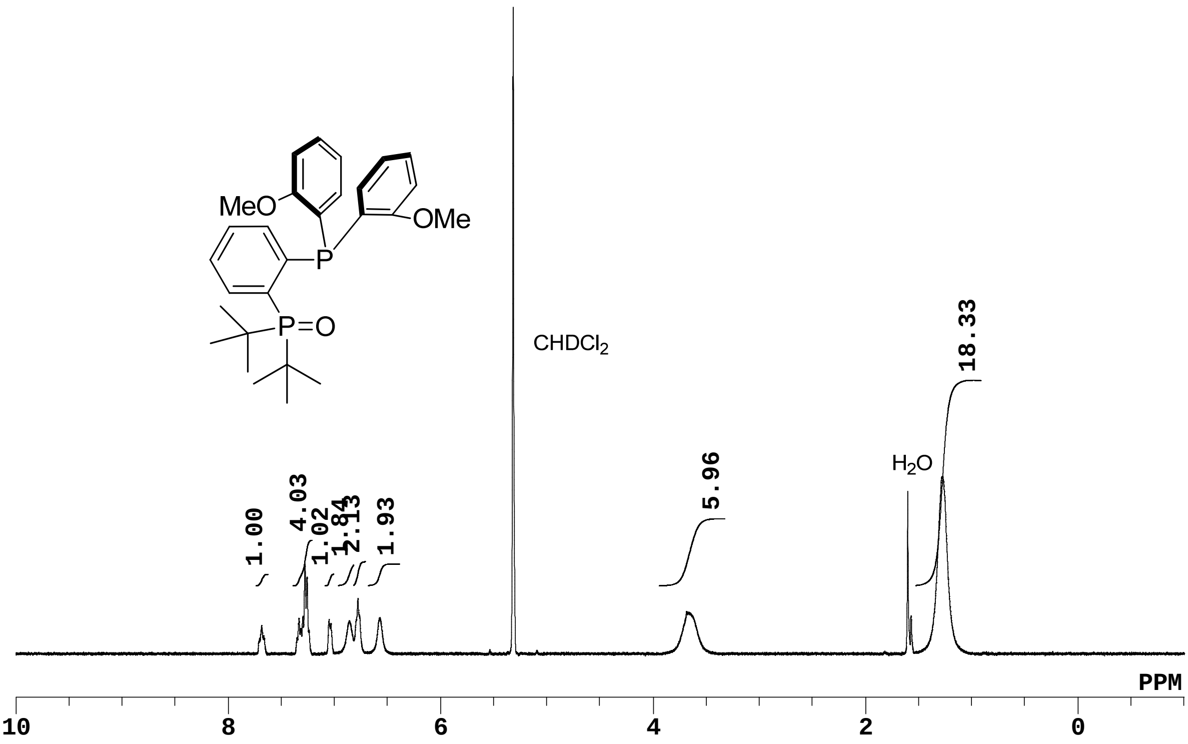

Figure S3. ^{1}H NMR spectrum (CD_2Cl_2, 400 MHz) of **S2**.

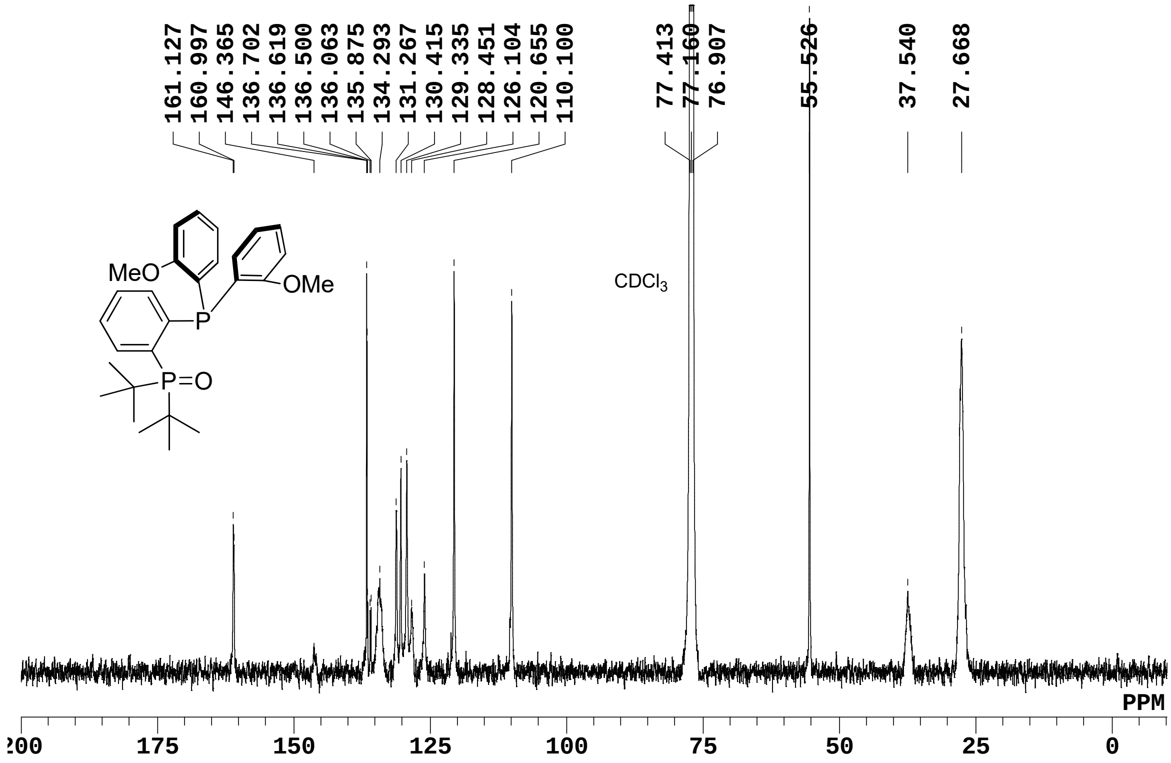

Figure S4. $^{13}C\{^{1}H\}$ NMR spectrum ($CDCl_3$, 126 MHz) of **S2**.

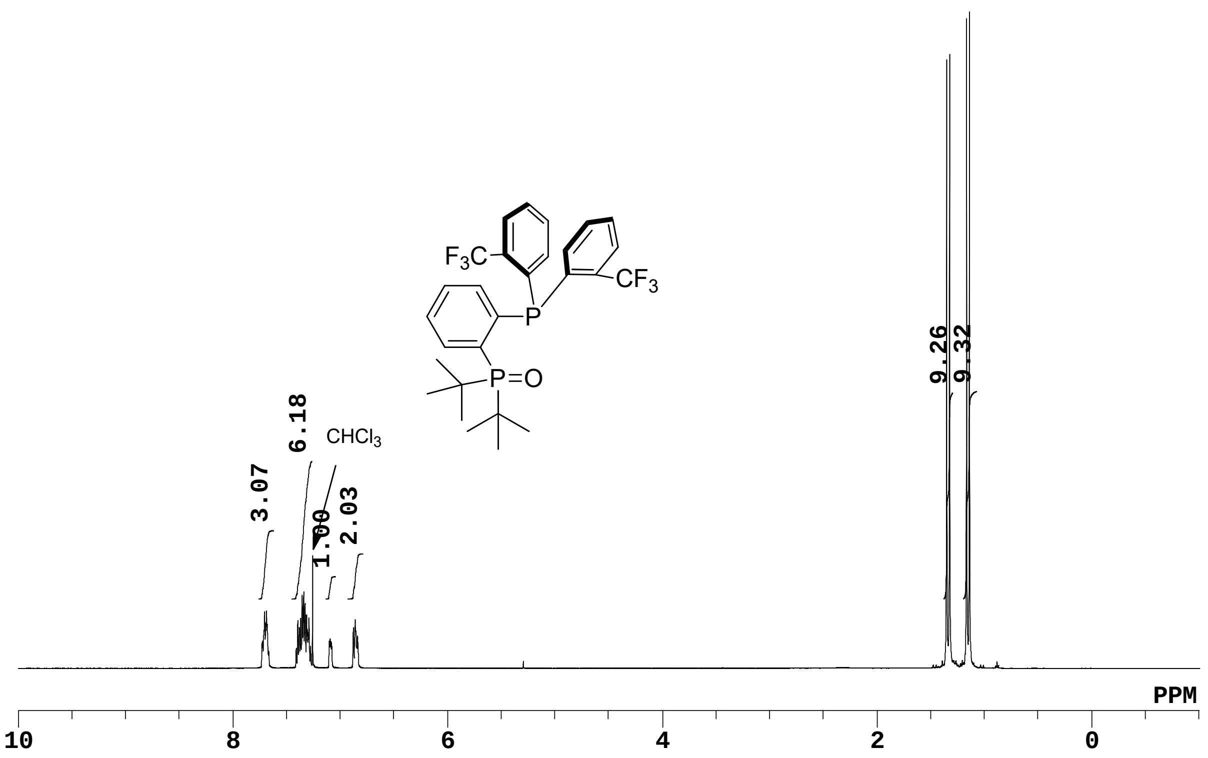

Figure S5. ^{1}H NMR spectrum ($CDCl_3$, 500 MHz) of **S3**.

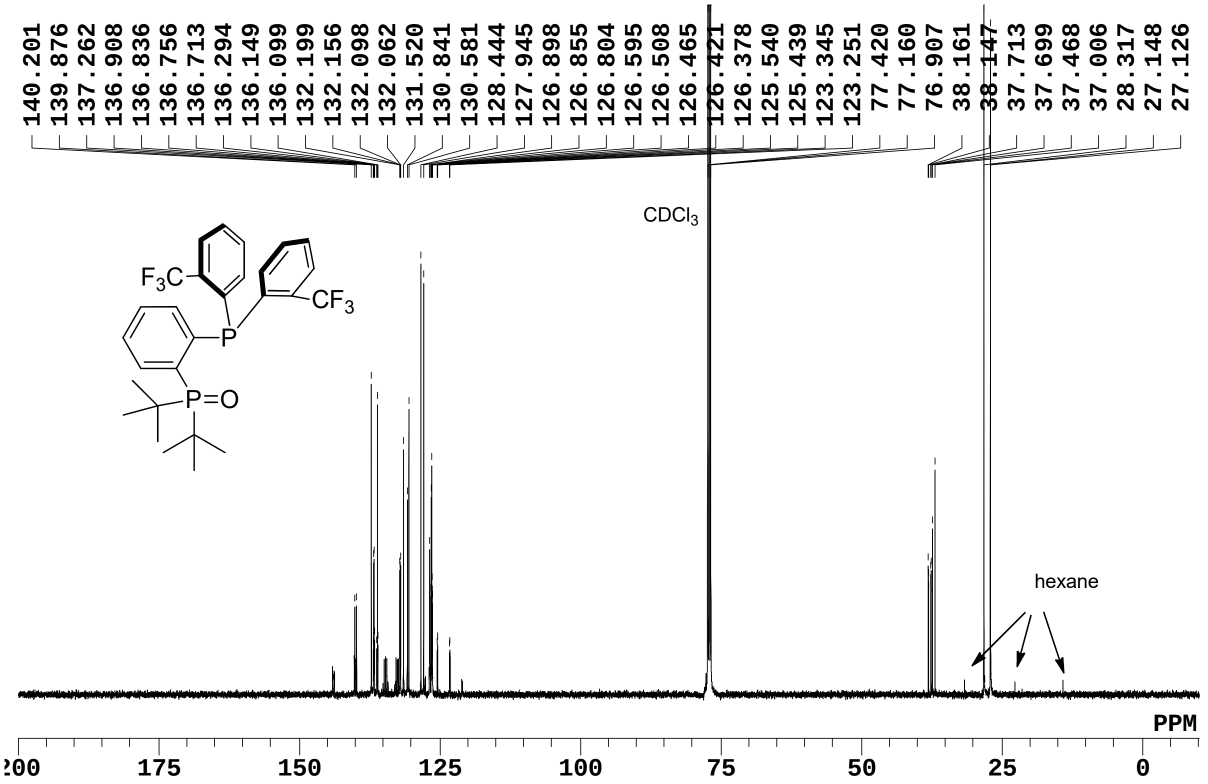

Figure S6. $^{13}C\{^{1}H\}$ NMR spectrum ($CDCl_3$, 126 MHz) of **S3**.

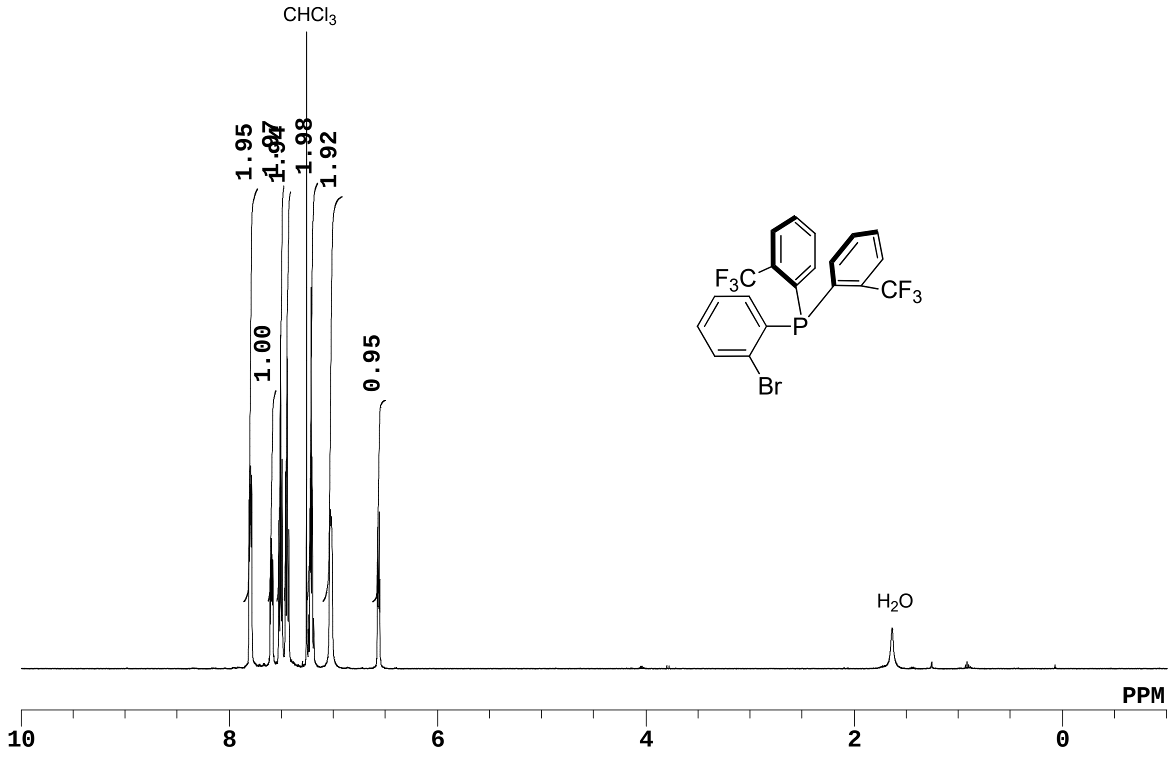

Figure S7. ^{1}H NMR spectrum ($CDCl_3$, 500 MHz) of **S4**.

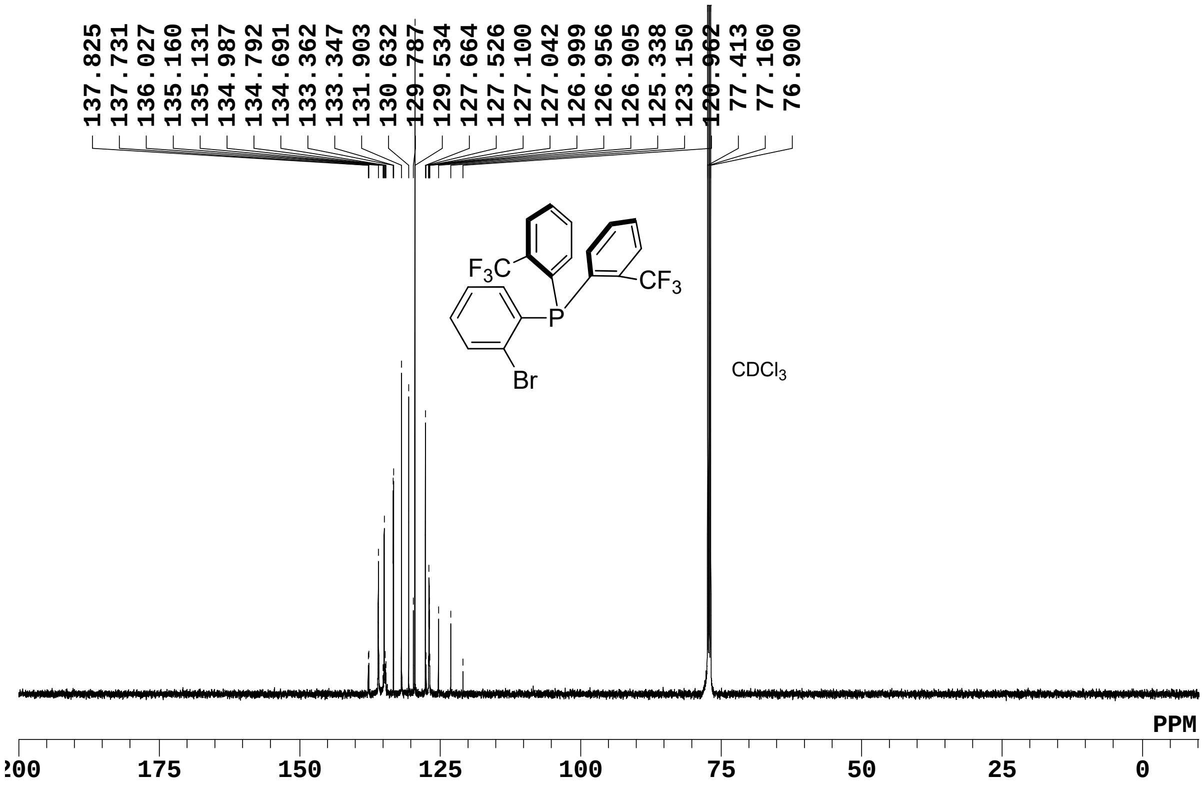

Figure S8 $^{13}C\{^{1}H\}$ NMR spectrum ($CDCl_3$, 126 MHz) of **S4**.

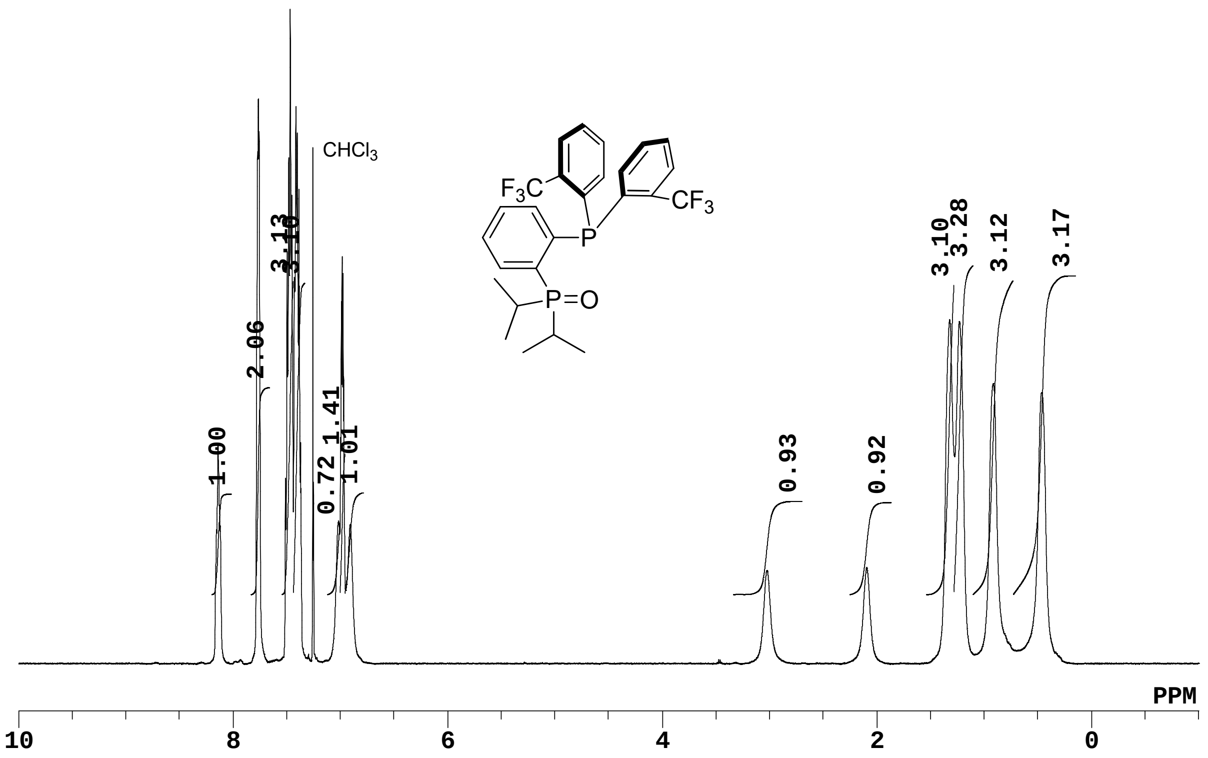

Figure S9. ^{1}H NMR spectrum ($CDCl_3$, 500 MHz) of **S5**.

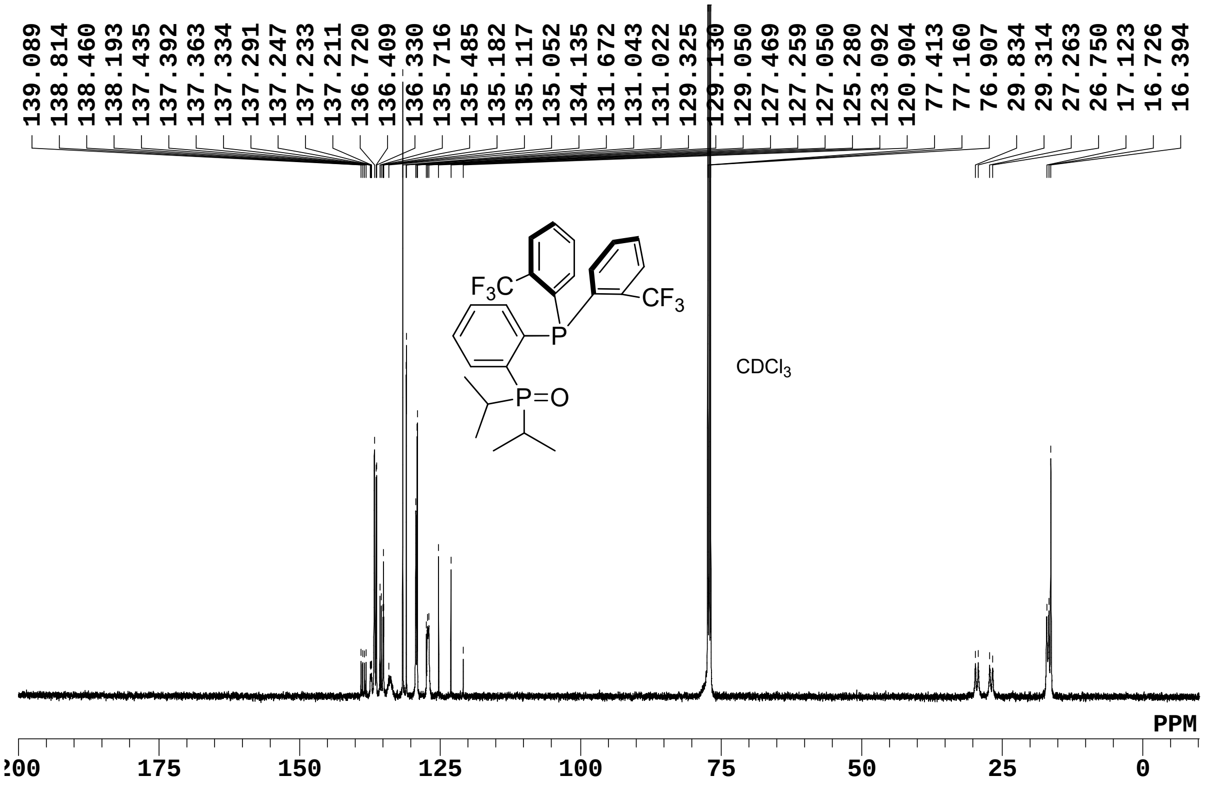

Figure S10. $^{13}C\{^{1}H\}$ NMR spectrum ($CDCl_3$, 126 MHz) of **S5**.

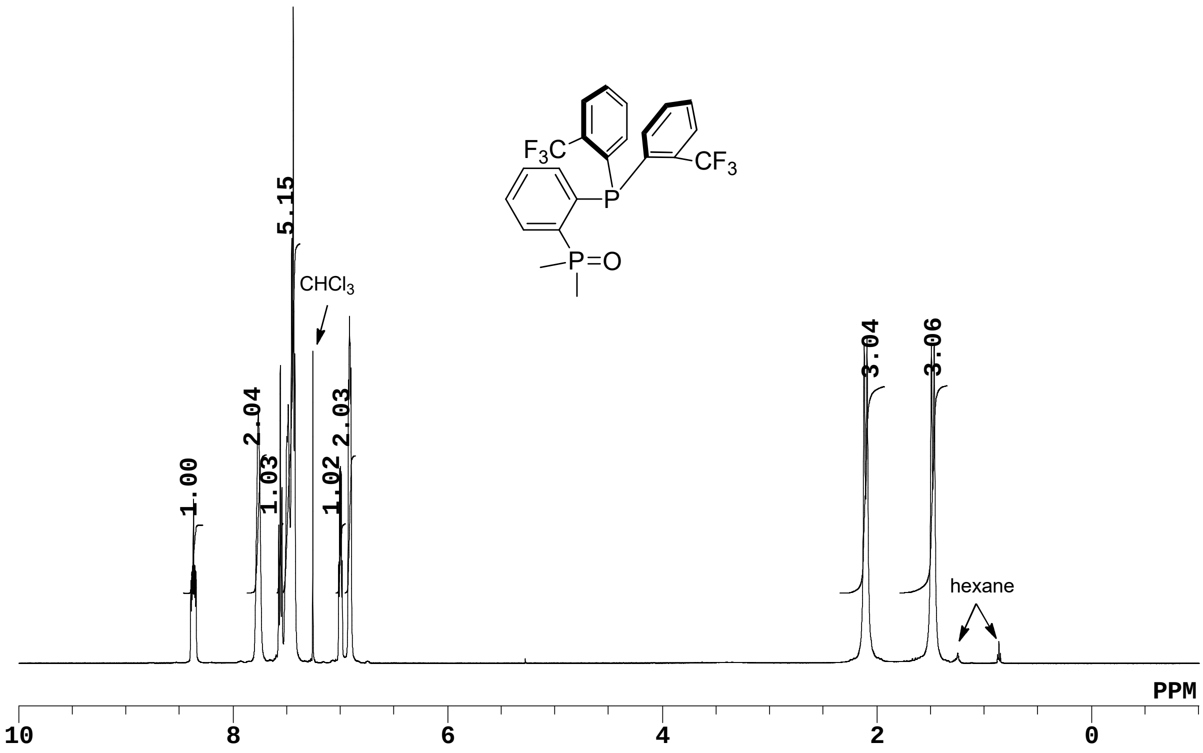

Figure S11. ^{1}H NMR spectrum (CDCl3, 500 MHz) of **S6**.

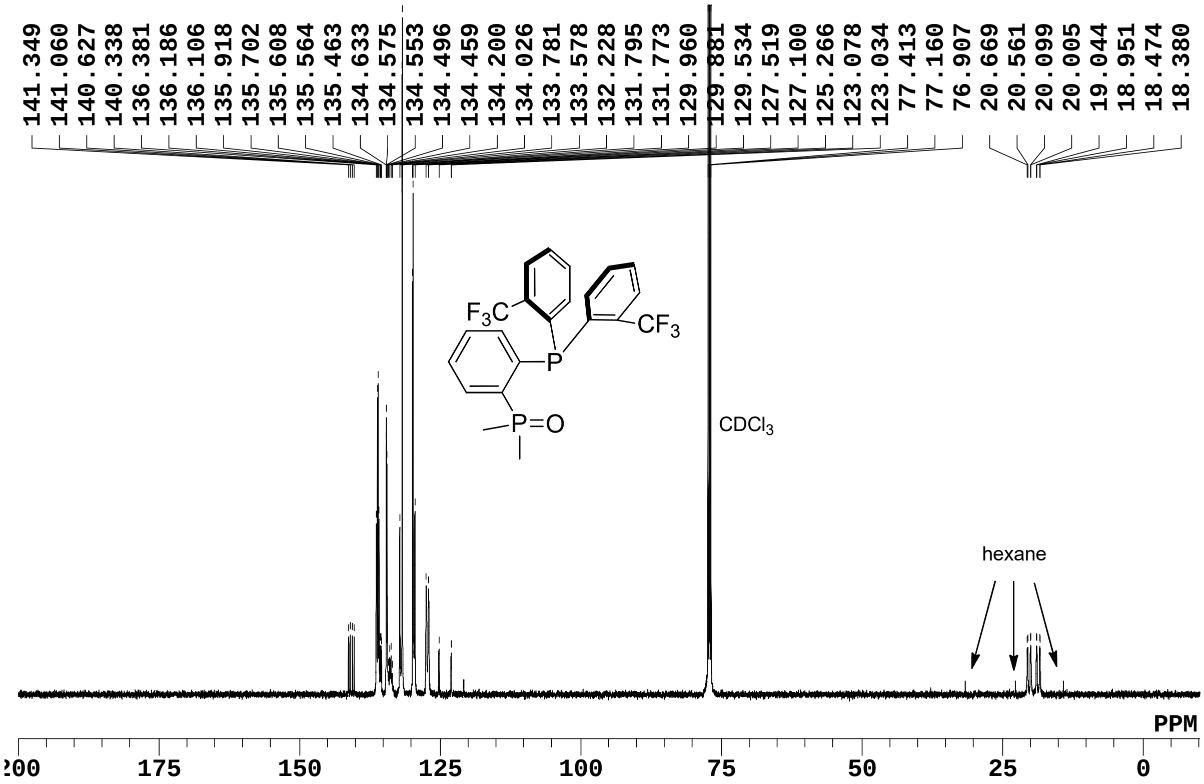

Figure S12. $^{13}C\{^1H\}$ NMR spectrum ($CDCl_3$, 126 MHz) of **S6**.

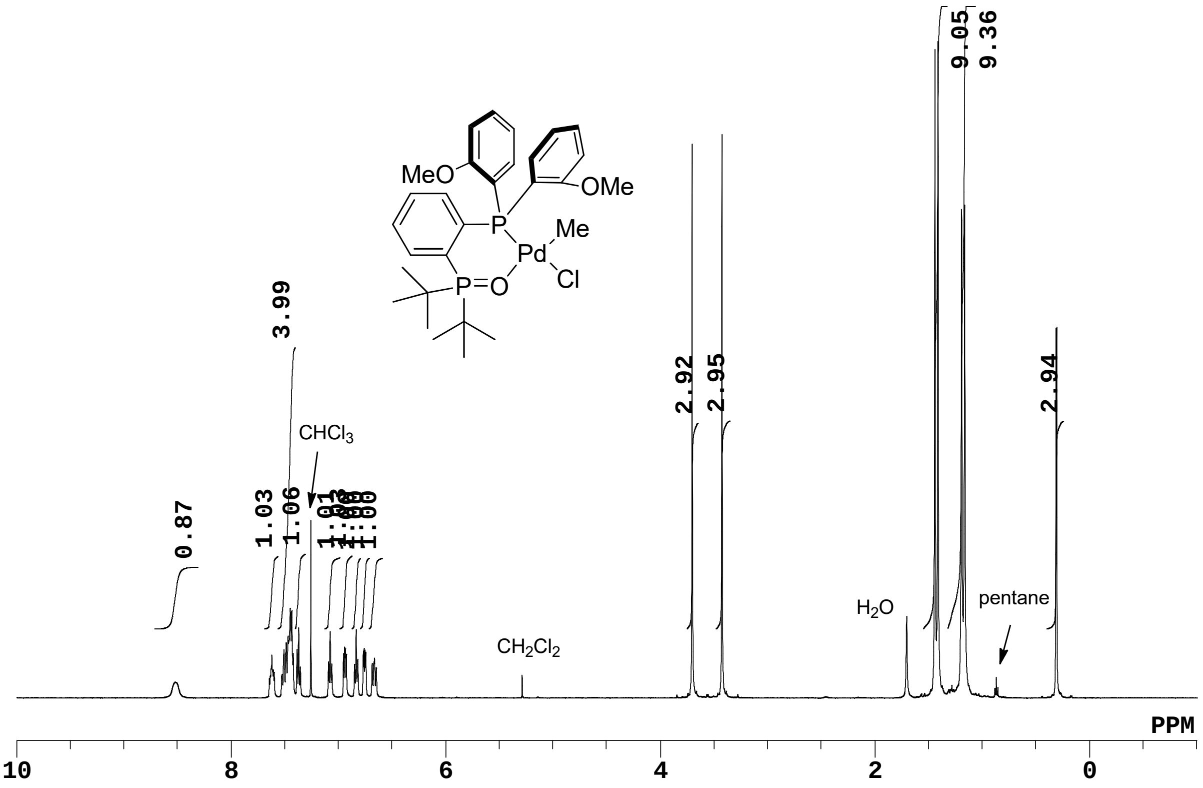

Figure S13. ^{1}H NMR spectrum ($CDCl_3$, 500 MHz) of **3c**.

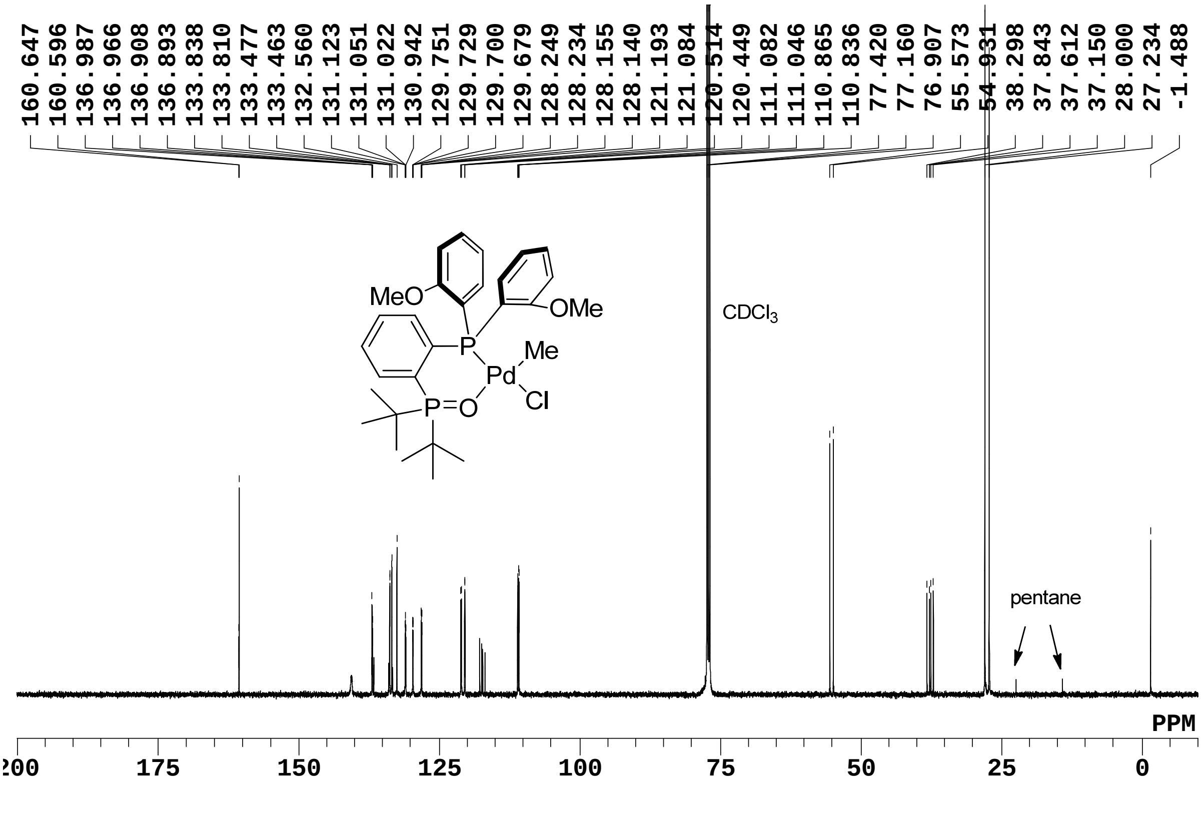

Figure S14. $^{13}C\{^{1}H\}$ NMR spectrum ($CDCl_3$, 126 MHz) of **3c**.

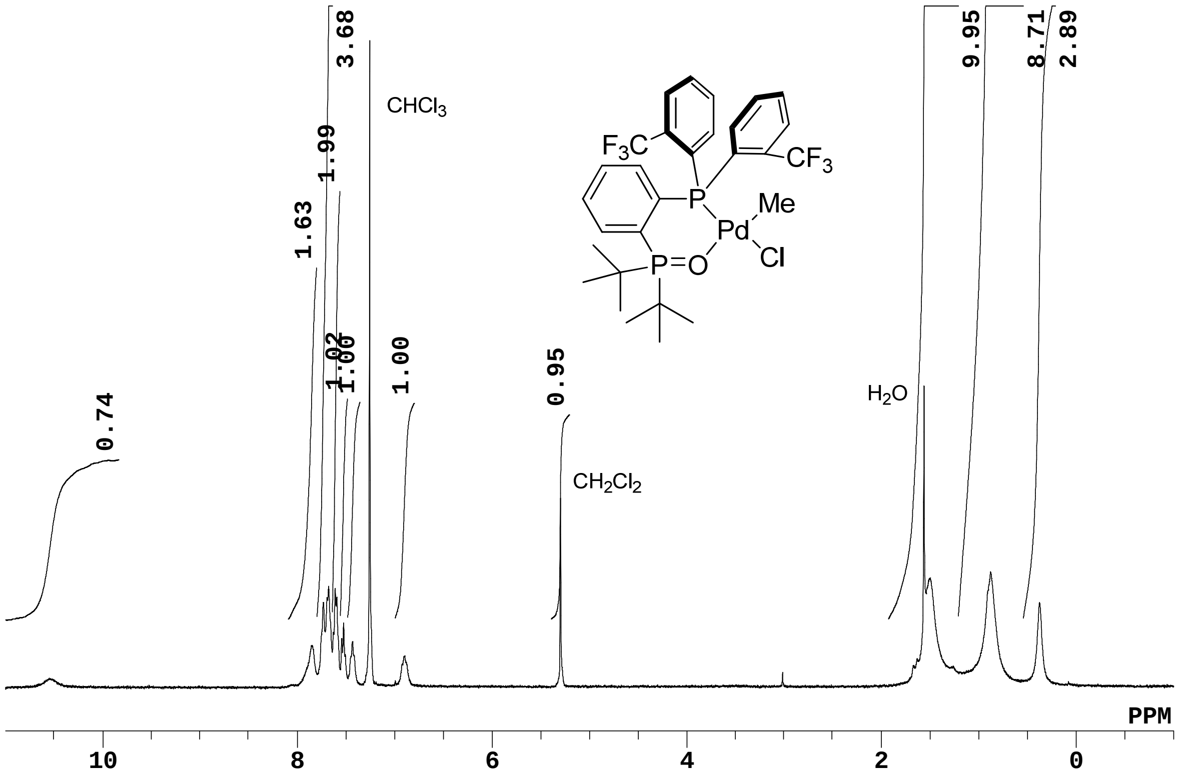

Figure S15. ^{1}H NMR spectrum ($CDCl_3$, 400 MHz) of **3d** obtained as a 2:1 DCM adduct.

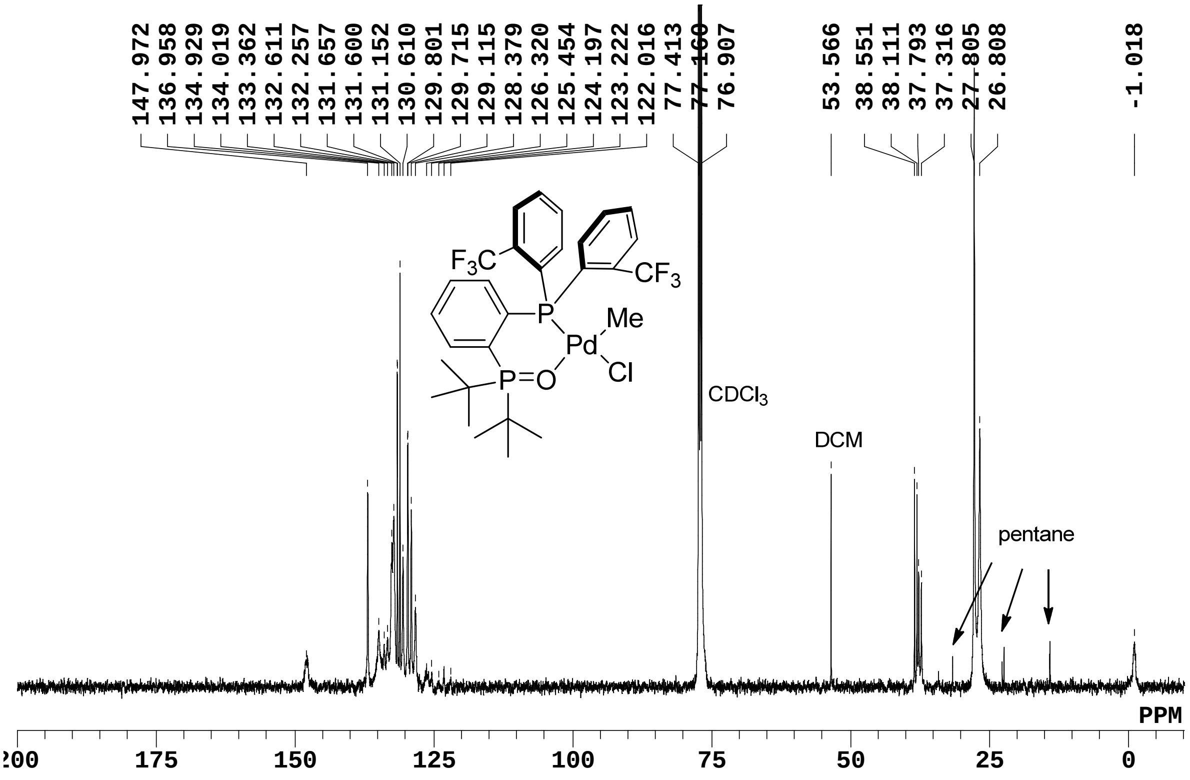

Figure S16. $^{13}C\{^1H\}$ NMR spectrum (CD_2Cl_2, 126 MHz) of **3d** obtained as a 2:1 DCM adduct.

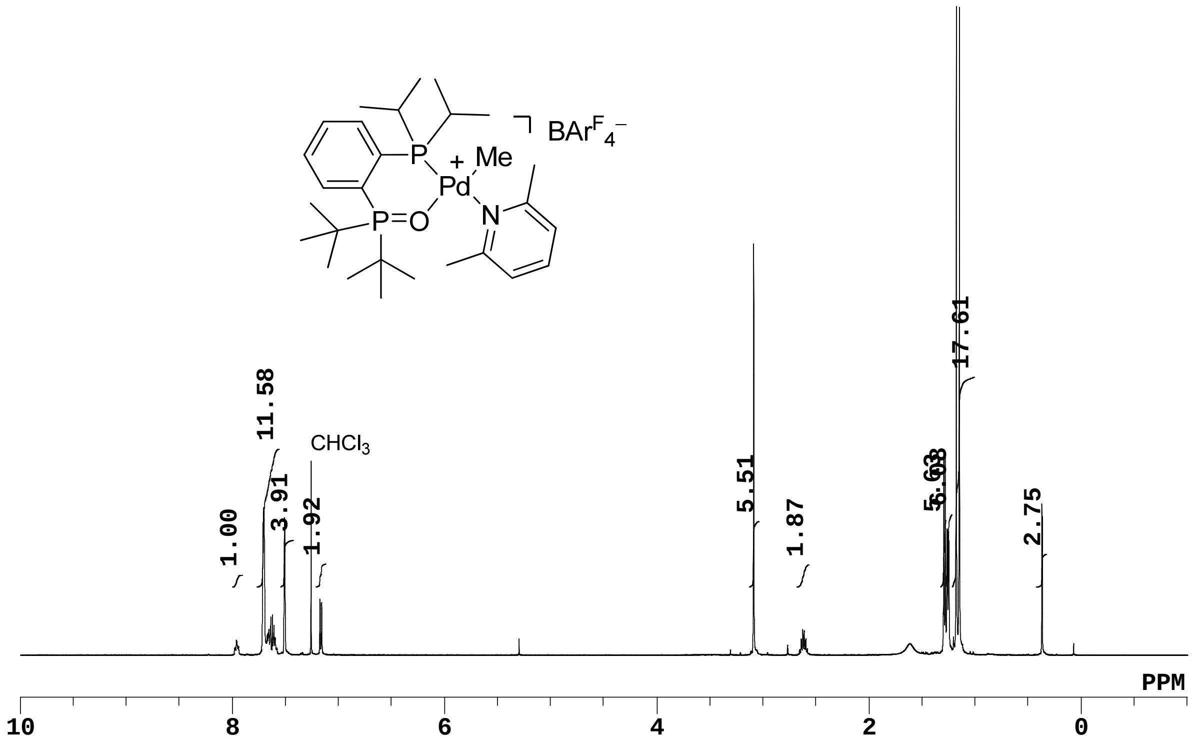

Figure S17. ^{1}H NMR spectrum ($CDCl_3$, 500 MHz) of **1a**.

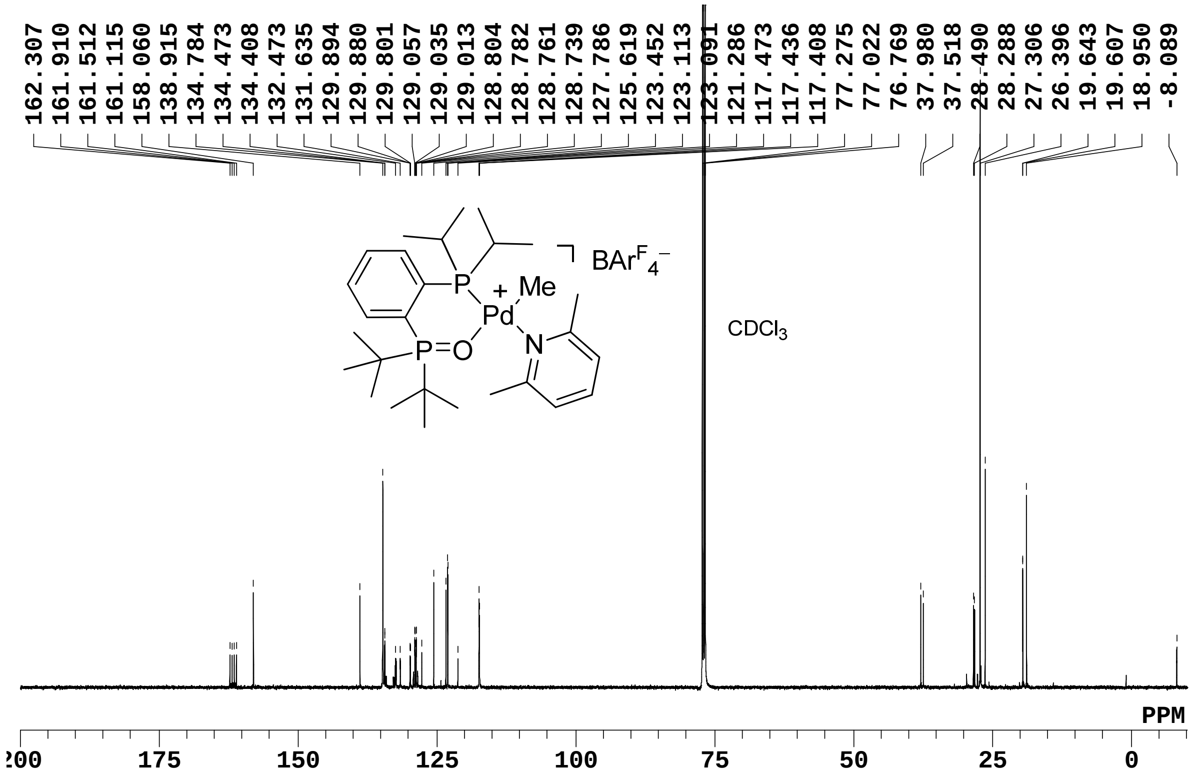

Figure S18. $^{13}C\{^{1}H\}$ NMR spectrum ($CDCl_3$, 126 MHz) of **1a**.

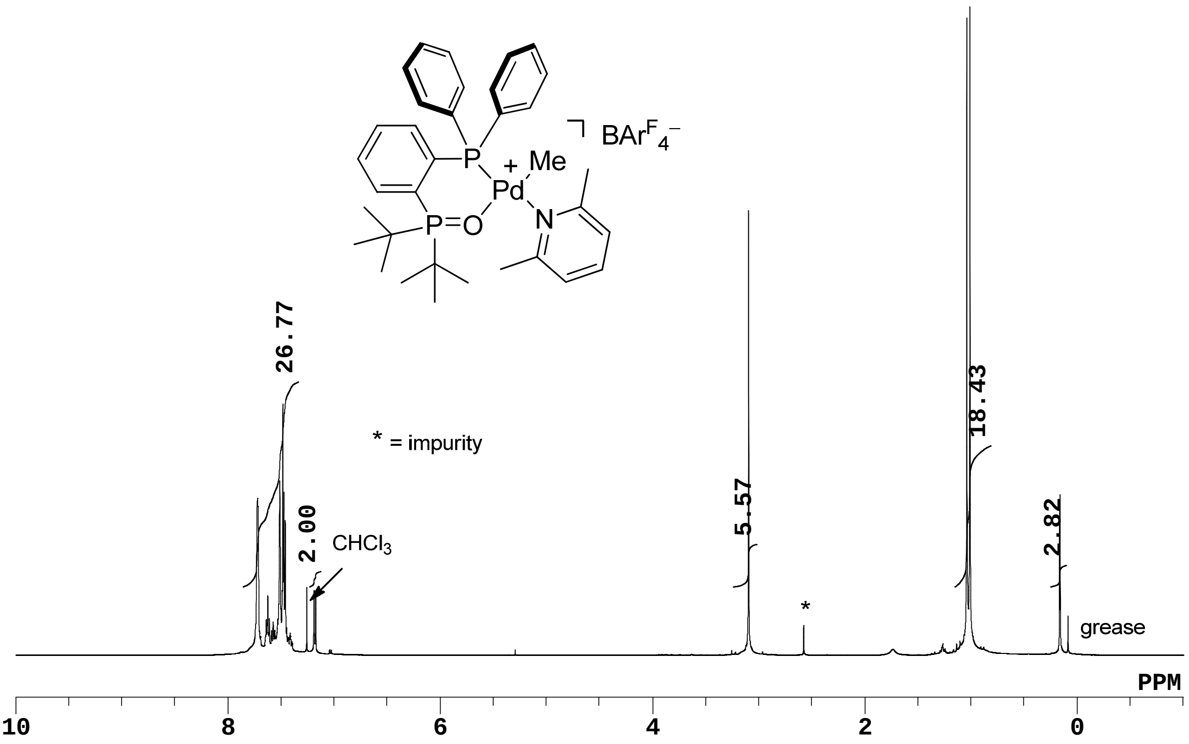

Figure S19. ^{1}H NMR spectrum ($CDCl_3$, 400 MHz) of **1b**.

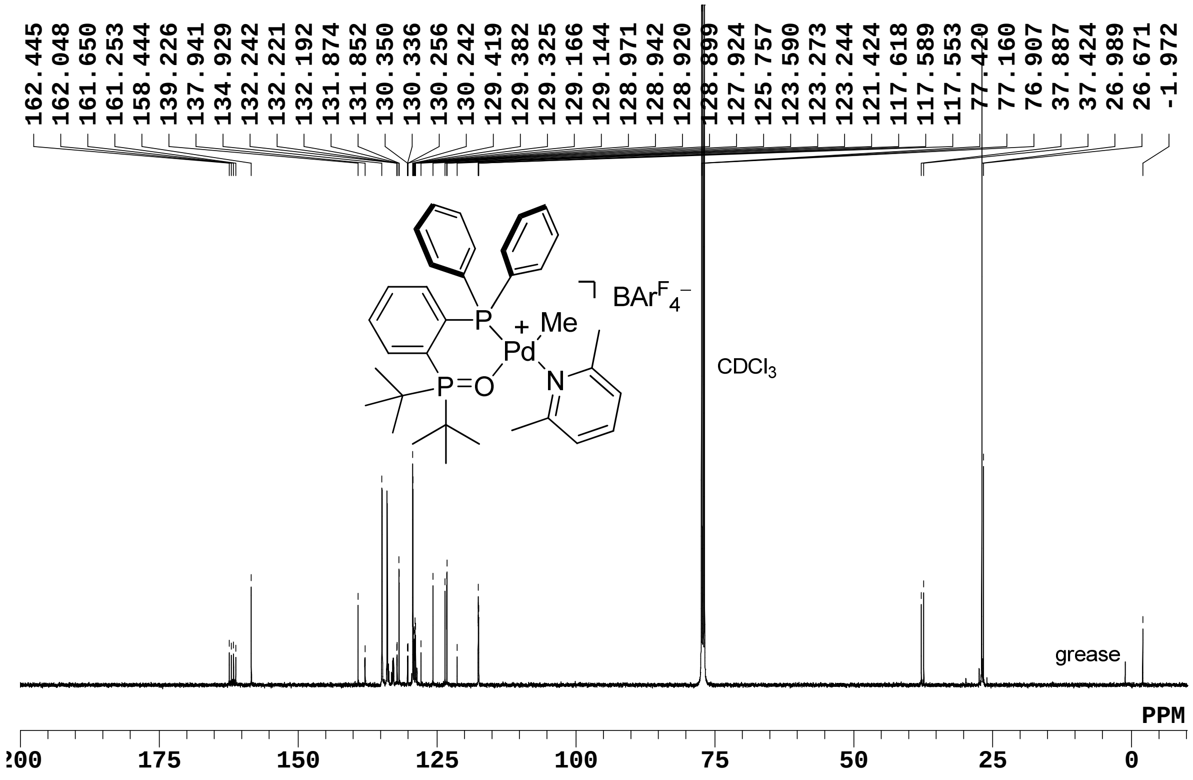

Figure S20. $^{13}C\{^{1}H\}$ NMR spectrum ($CDCl_3$, 126 MHz) of **1b**.

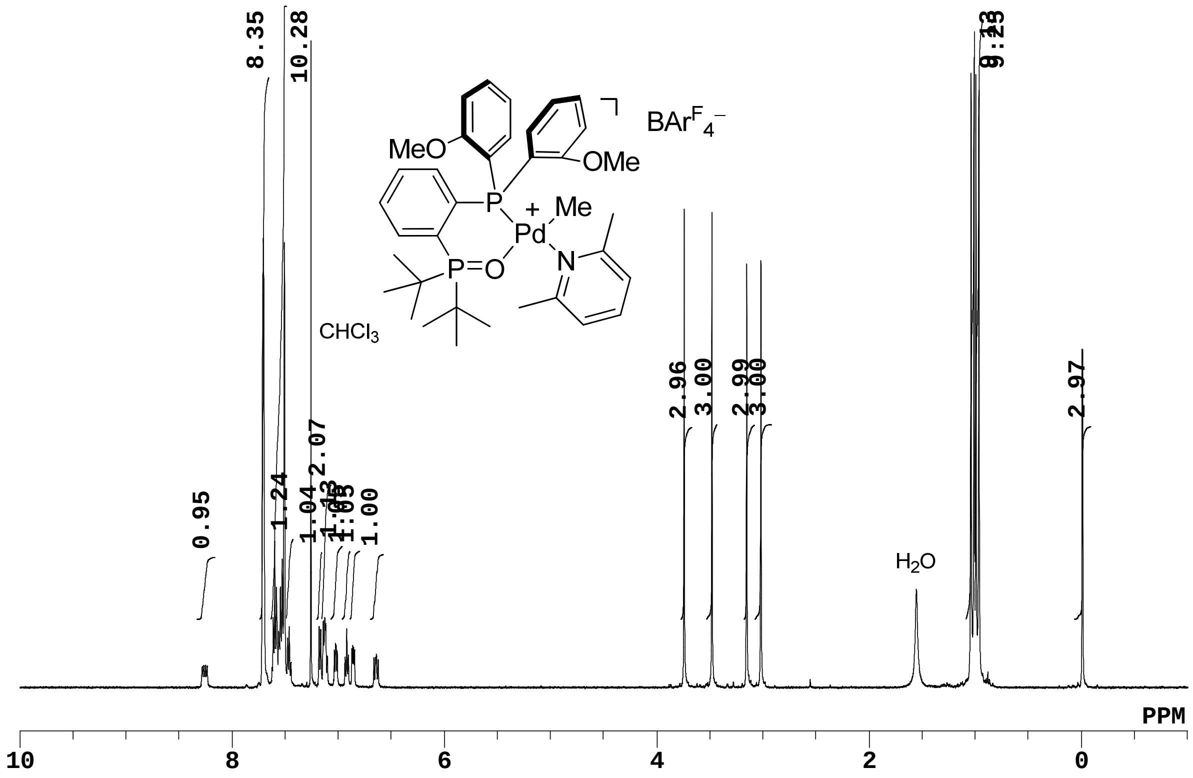

Figure S21. ^{1}H NMR spectrum ($CDCl_3$, 500 MHz) of **1c**.

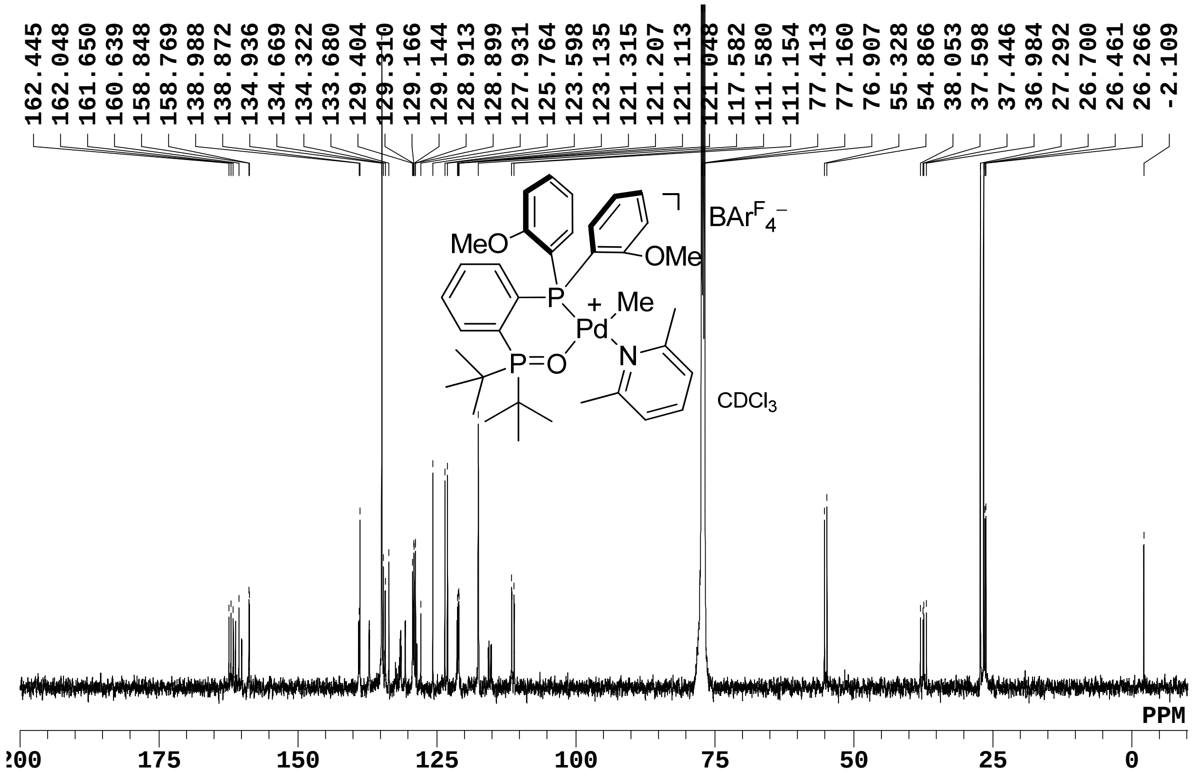

Figure S22. ^{13}C{^{1}H} NMR spectrum ($CDCl_3$, 126 MHz) of **1c**.

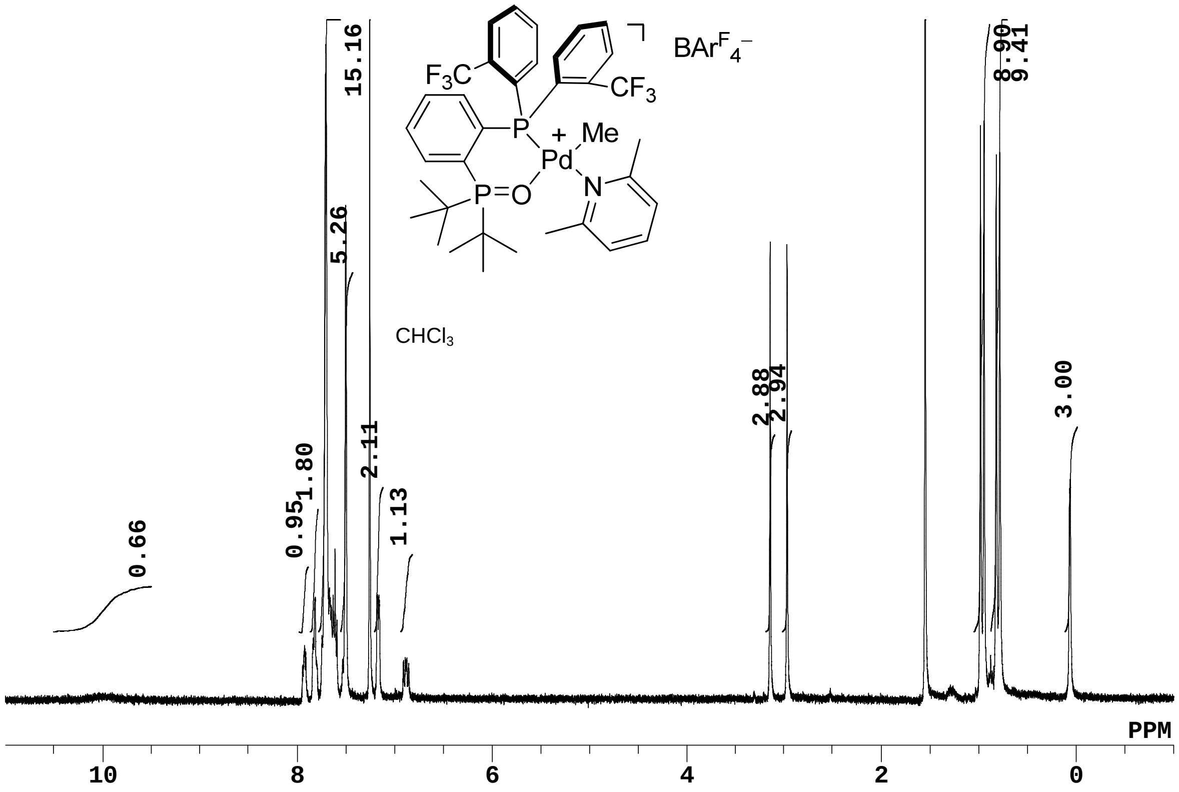

Figure S23. ^{1}H NMR spectrum ($CDCl_3$, 500 MHz) of **1d**.

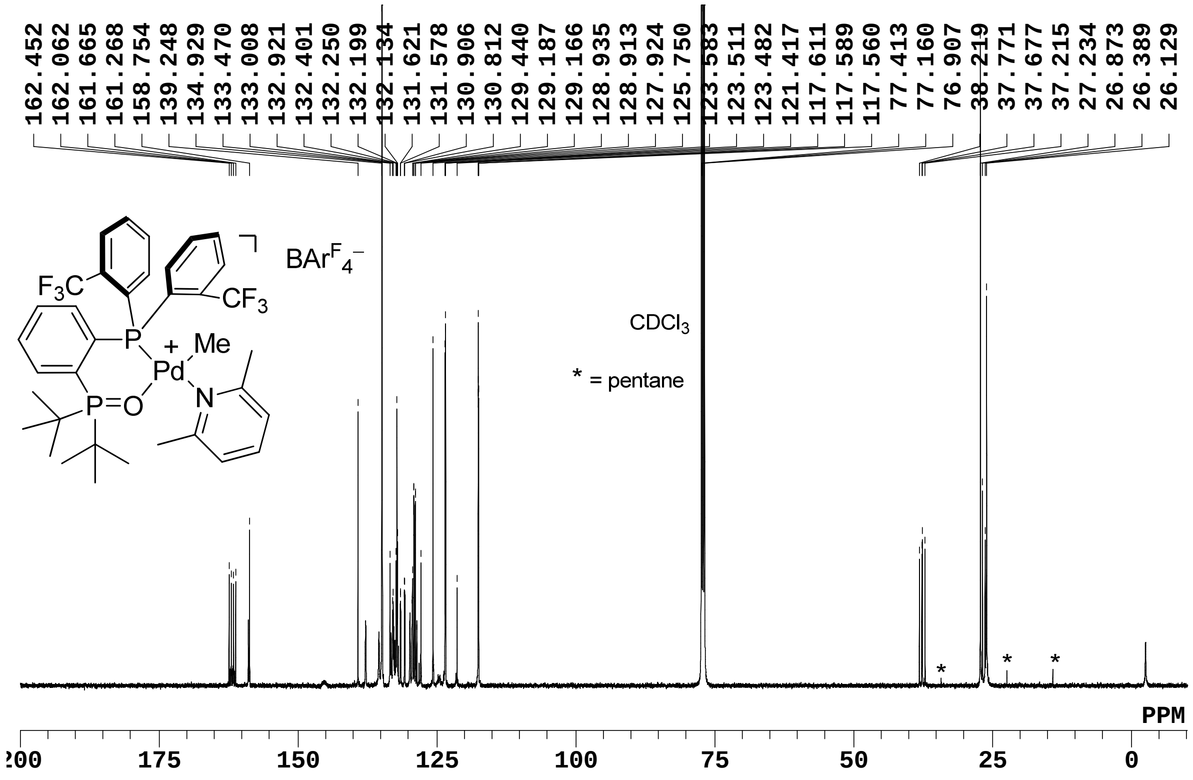

Figure S24. $^{13}C\{^{1}H\}$ NMR spectrum ($CDCl_3$, 126 MHz) of **1d**.

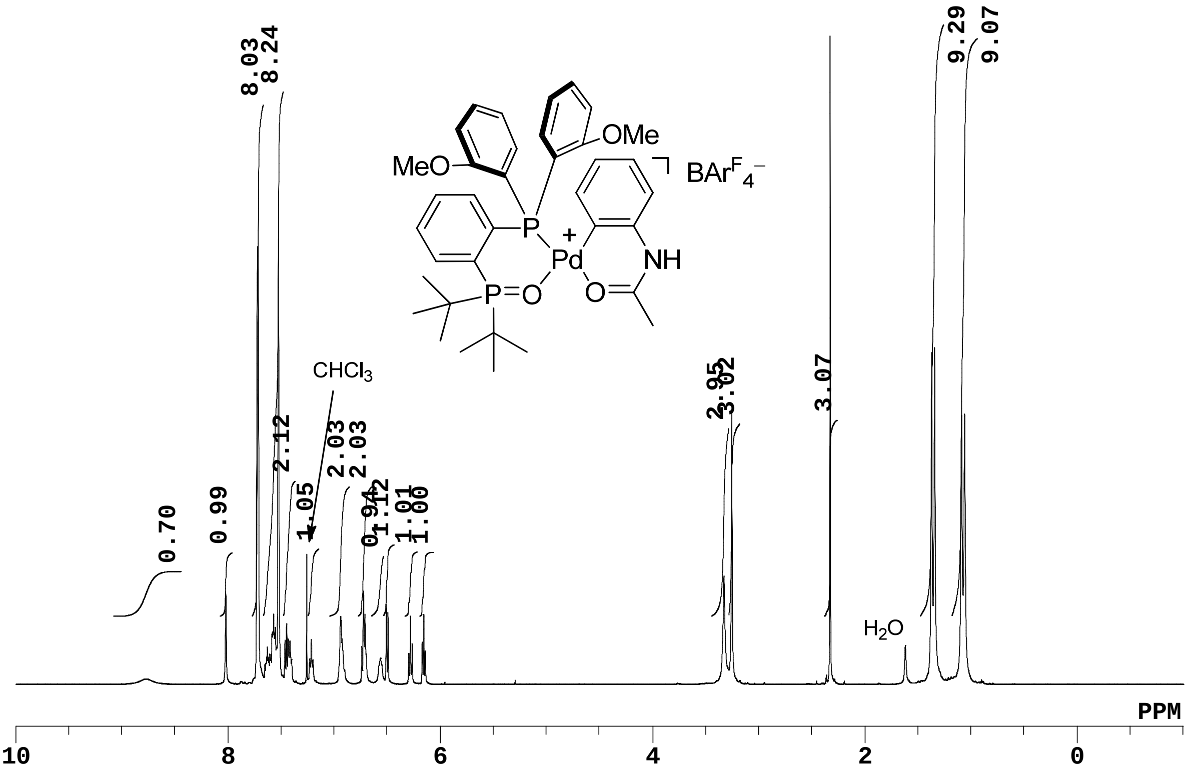

Figure S25. ^{1}H NMR spectrum (CDCl$_3$, 500 MHz) of **2c**.

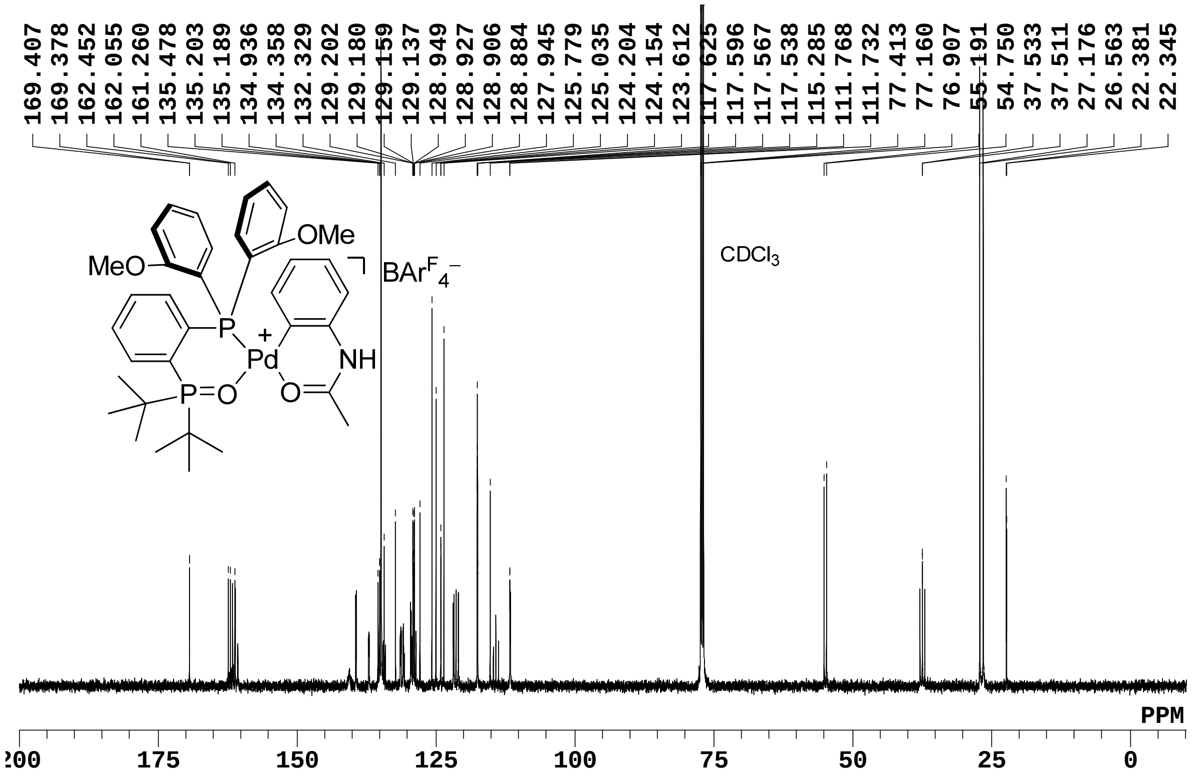

Figure S26. ^{13}C{^{1}H} NMR spectrum (CDCl$_3$, 126 MHz) of **2c**.

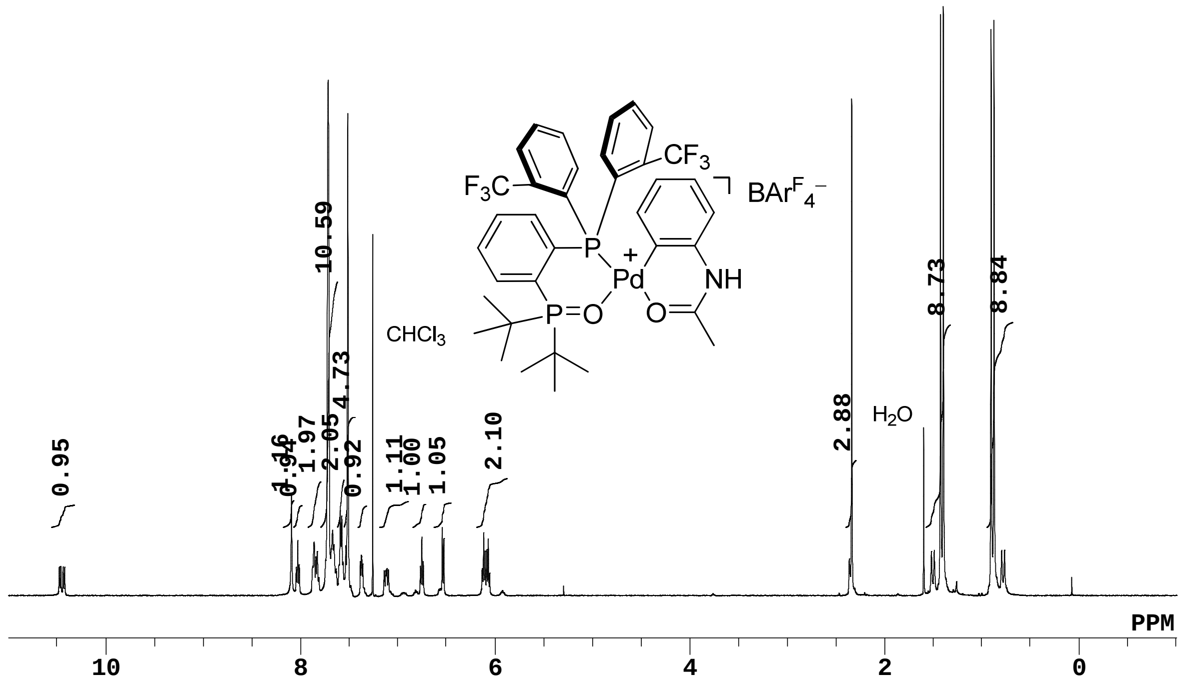

Figure S27. ^{1}H NMR spectrum ($CDCl_3$, 500 MHz) of **2d**.

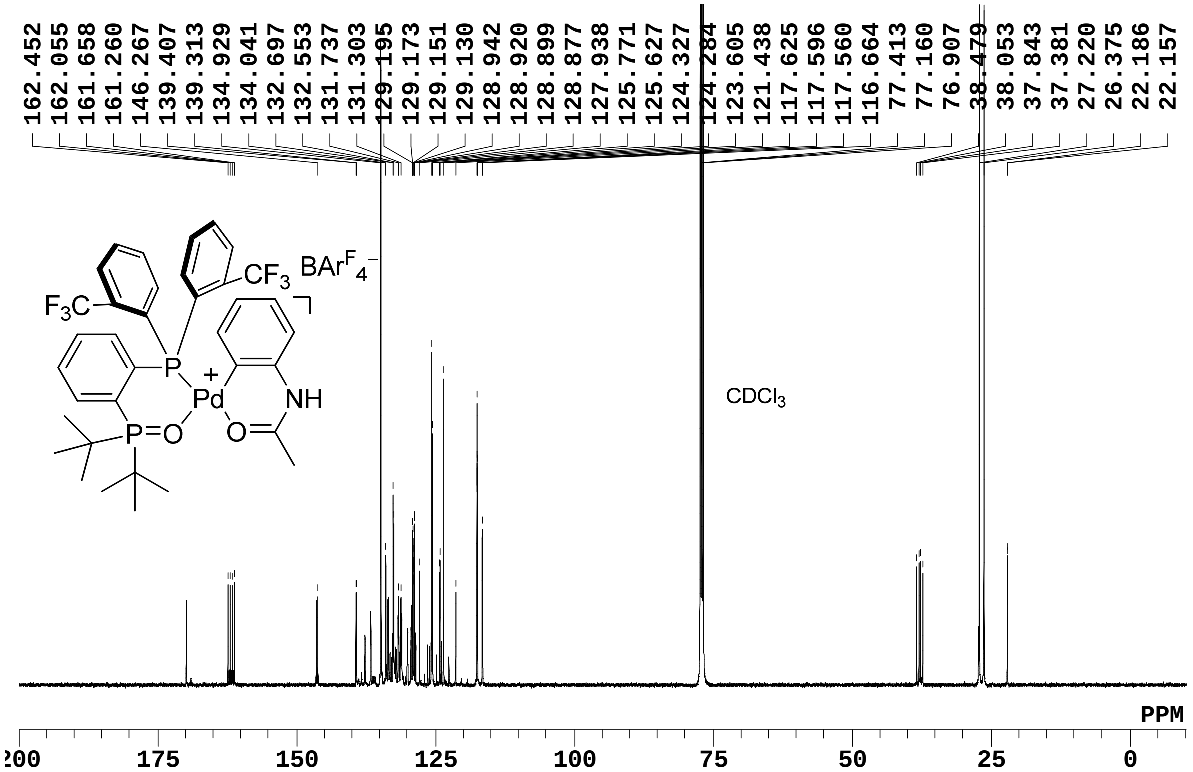

Figure S28. $^{13}C\{^{1}H\}$ NMR spectrum ($CDCl_3$,126 MHz) of **2d.**

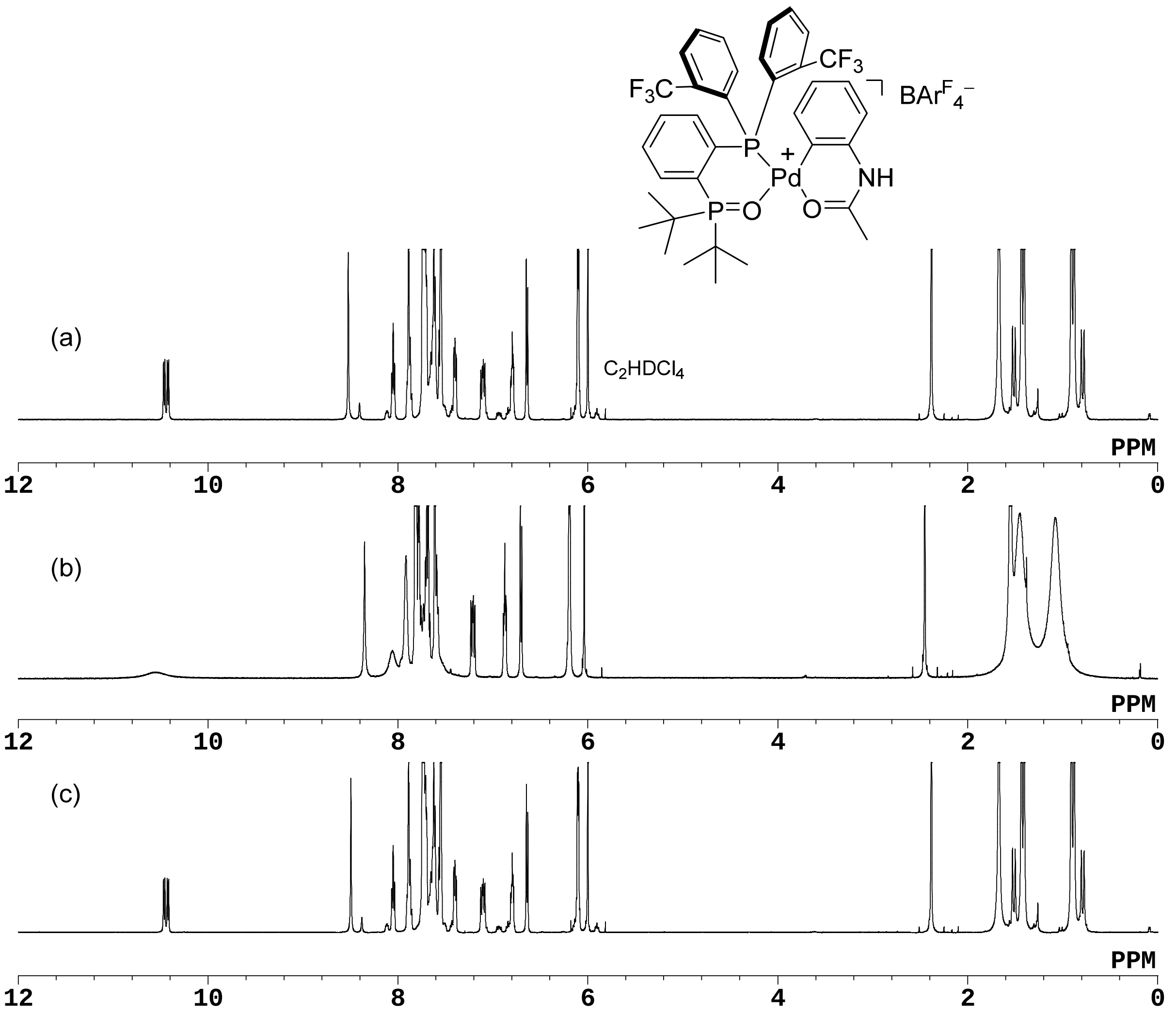

Figure S29. ^{1}H NMR spectrum ($C_2D_2Cl_4$, 500 MHz) of **2d** at (a) 25 °C, (b) 100 °C, (c) cooled to 25 °C after heating to 100 °C.

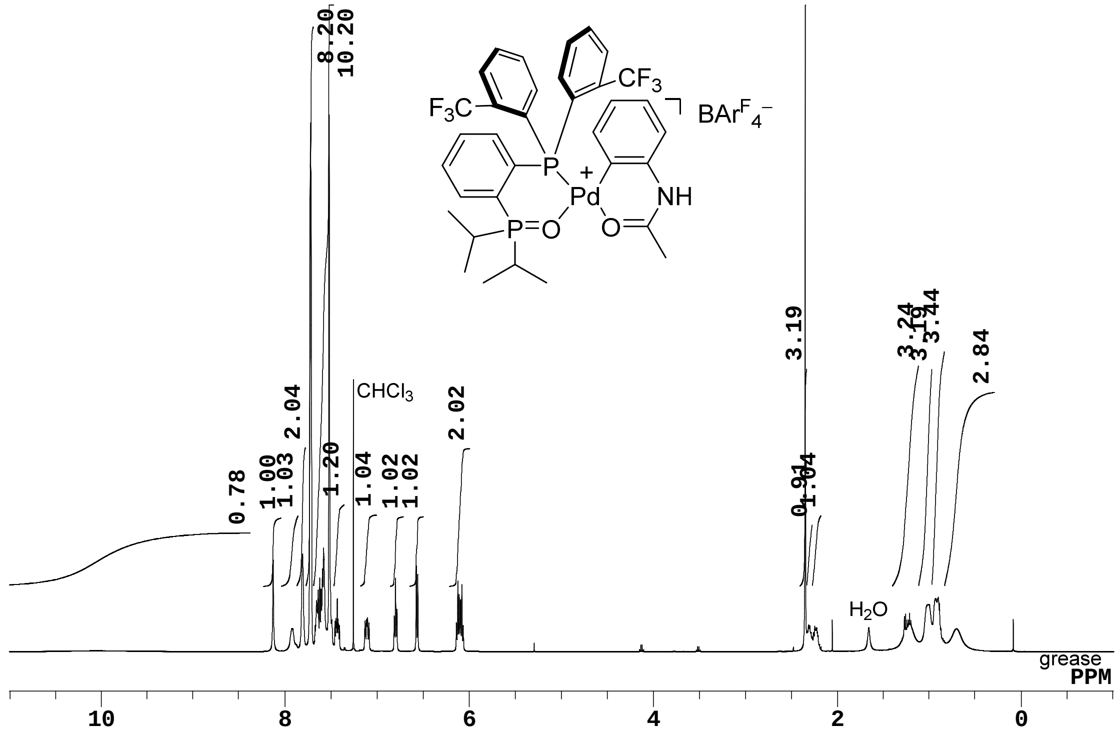

Figure S30. ^{1}H NMR spectrum ($CDCl_3$, 500 MHz) of **2e**.

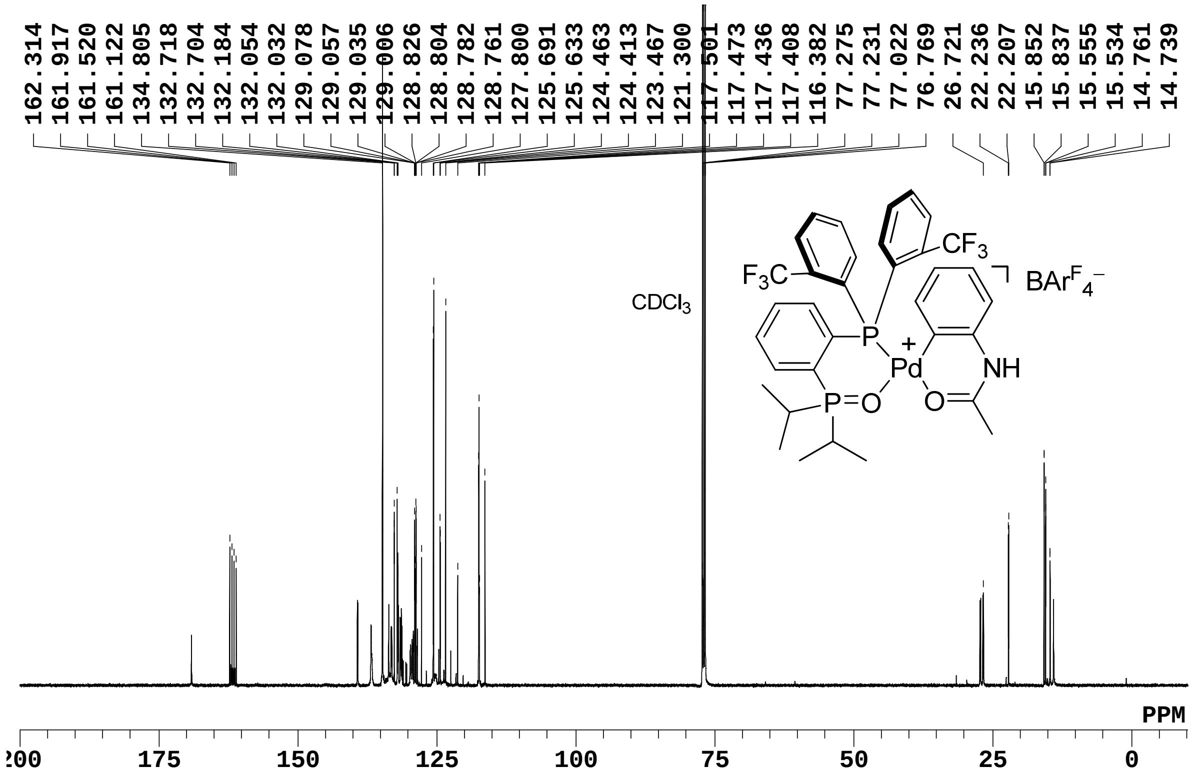

Figure S31. $^{13}C\{^{1}H\}$ NMR spectrum ($CDCl_3$, 126 MHz) of **2e**.

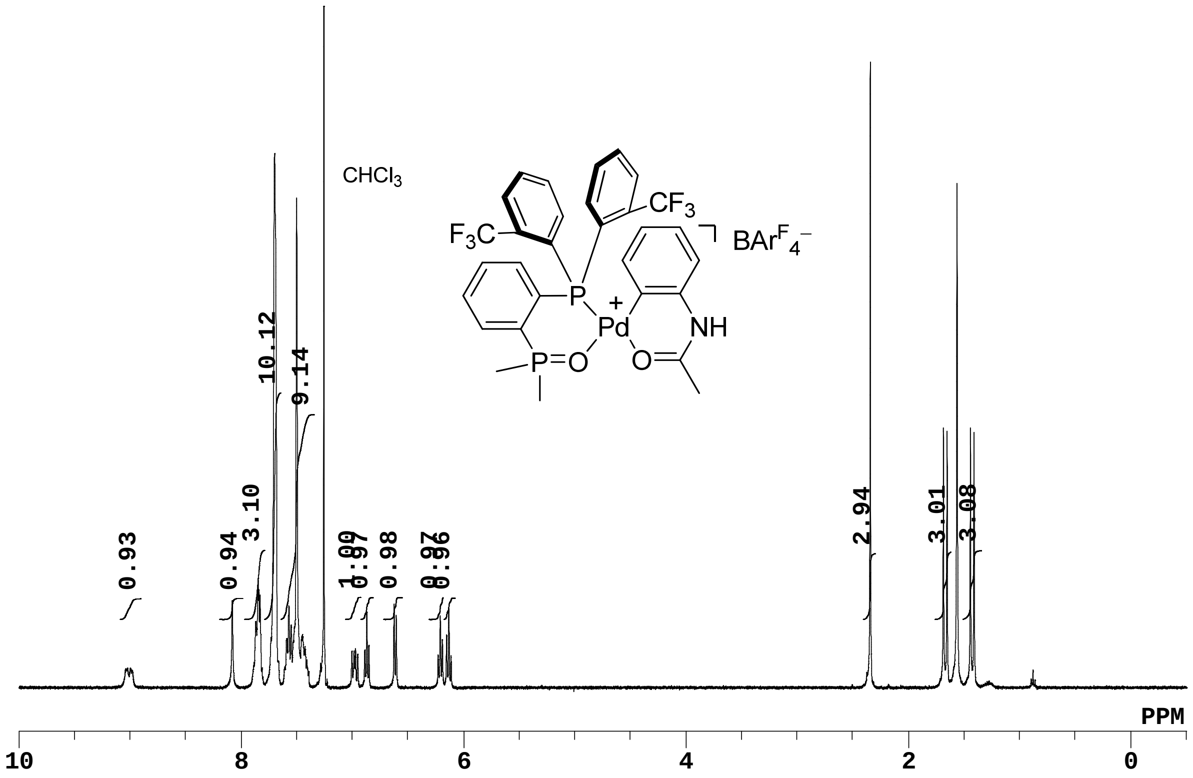

Figure S32. ^{1}H NMR spectrum ($CDCl_3$, 500 MHz) of **2f**.

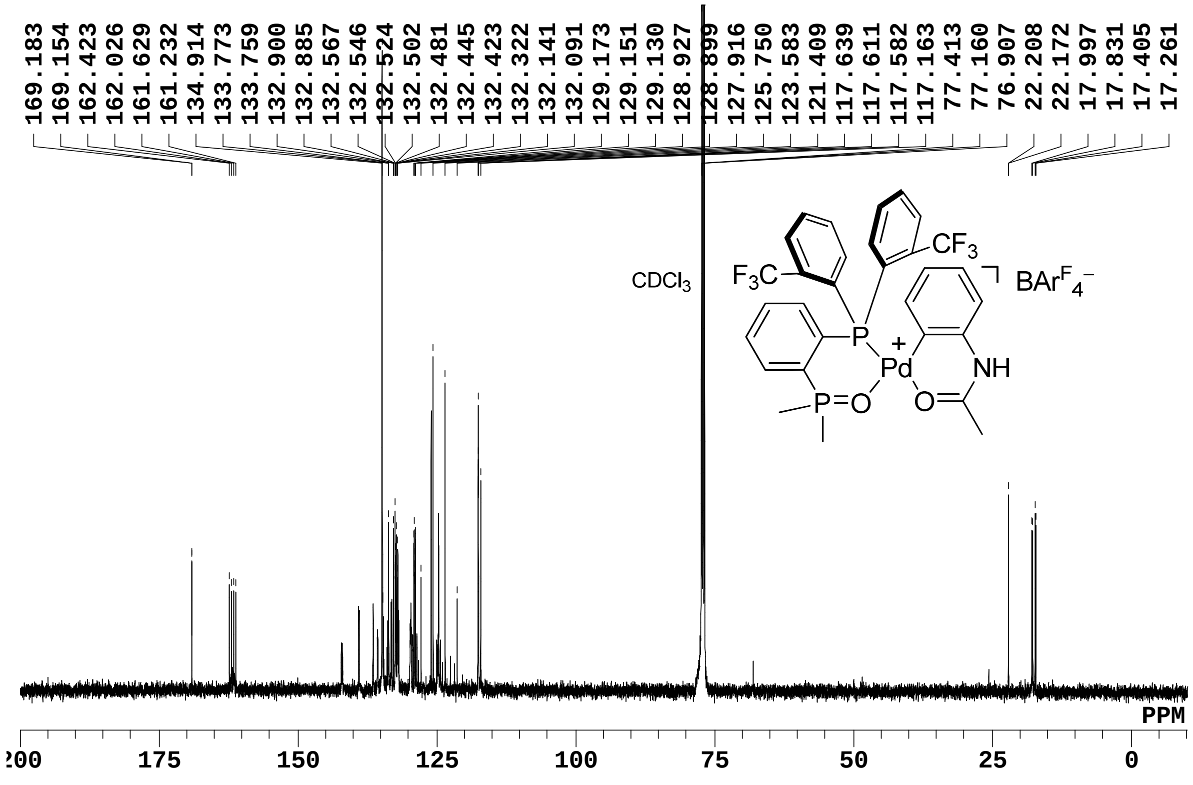

Figure S33. ^{13}C{^{1}H} NMR spectrum ($CDCl_3$, 500 MHz) of **2f**.

3. Thermolysis of MA-Inserted Complexes

Representative Procedure for Thermolysis of MA-Inserted Complexes (Figure S34)

A solution of complex **4a** (4.9 mg, 6.9 μmol) in $C_2D_2Cl_4$ (0.60 mL) was added into an NMR glass tube and then sealed. The solution was heated for 22 hours at 120 °C and NMR yield of methyl methacrylate was calculated to be 55% using the signal of residual proton of $C_2D_2Cl_4$ as an internal standard.

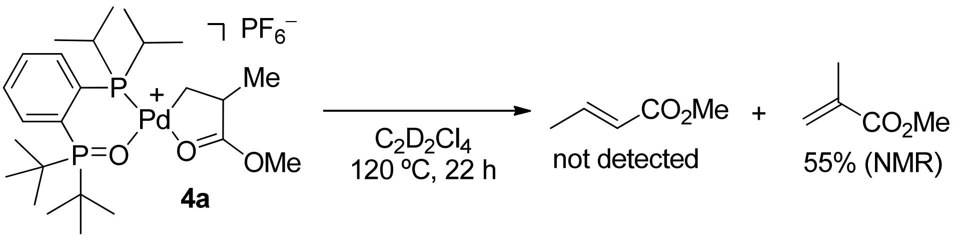

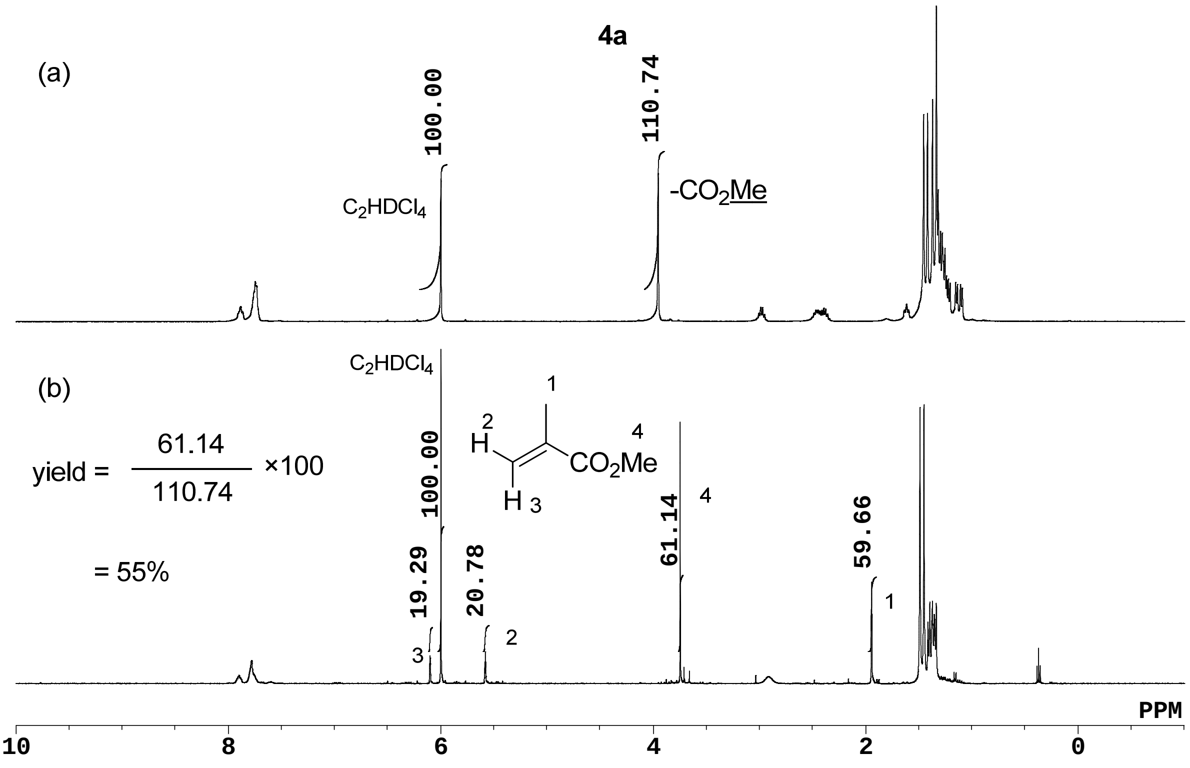

Figure S34. ^{1}H NMR spectrum of ($C_2D_2Cl_4$, 400 MHz) **4a** (a) before heating, (b) after heating.

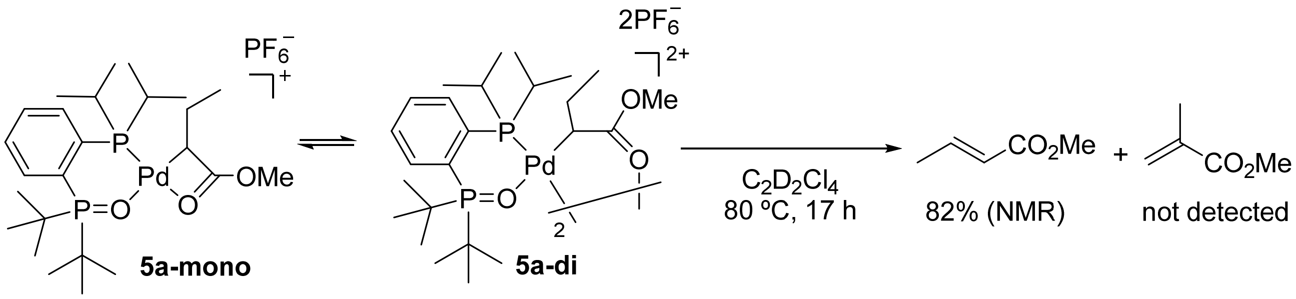

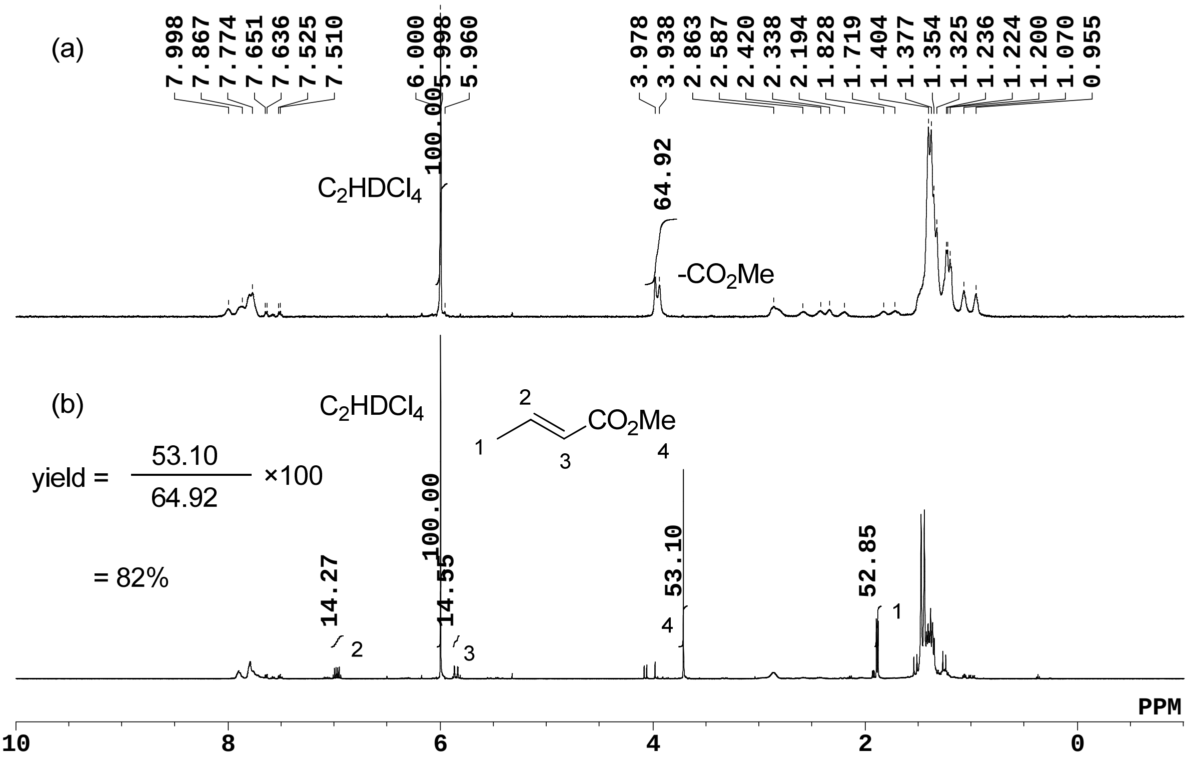

Figure S35. ^{1}H NMR spectrum ($C_2D_2Cl_4$, 500 MHz) of **5a** (a) before heating, (b) after heating.

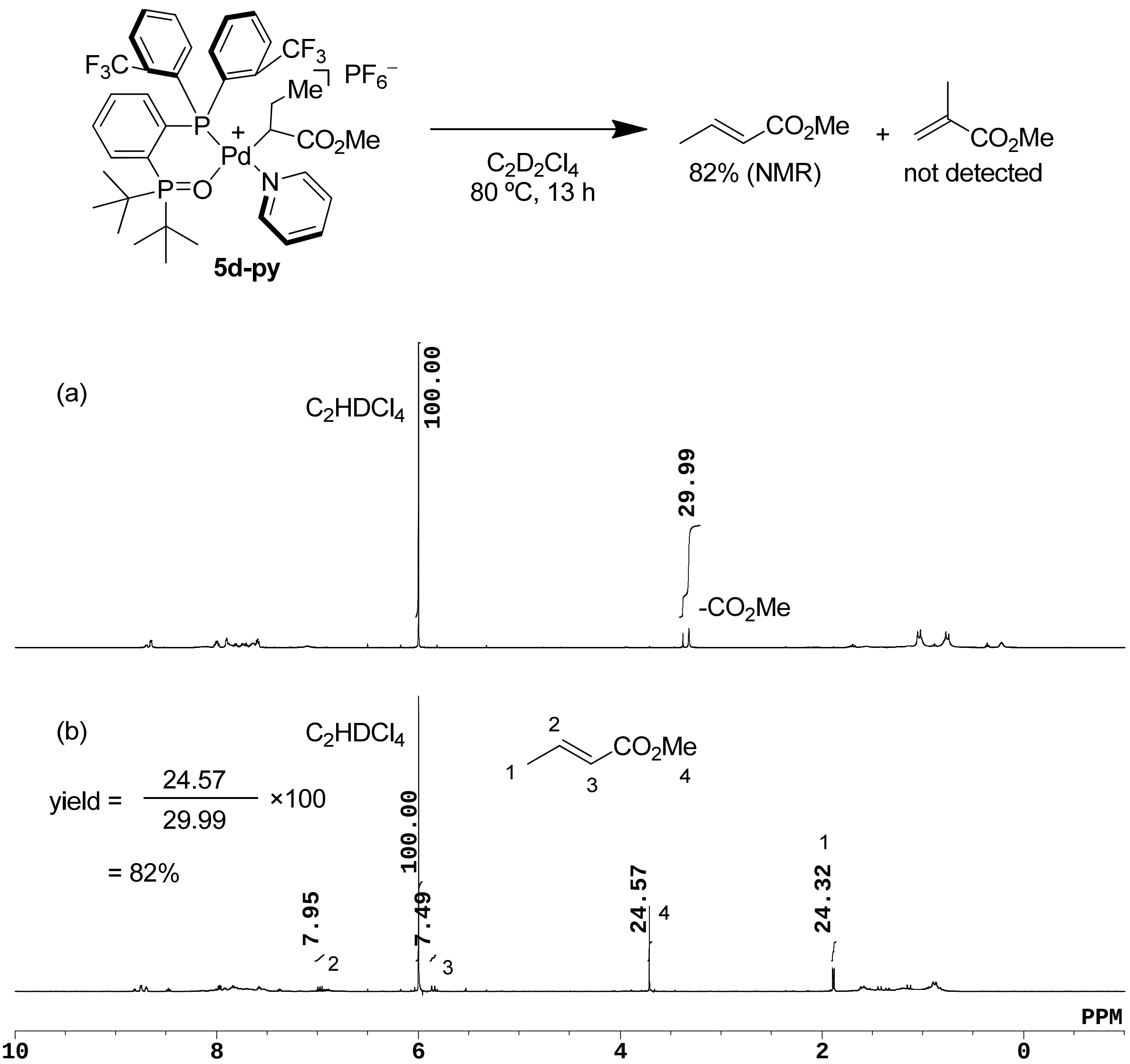

Figure S36. ^{1}H NMR spectrum ($C_2D_2Cl_4$, 500 MHz) of **5d-py** (a) before heating, (b) after heating.

4. Characterisation of MA-Inserted Complexes

4-1 NMR Spectra of 4a

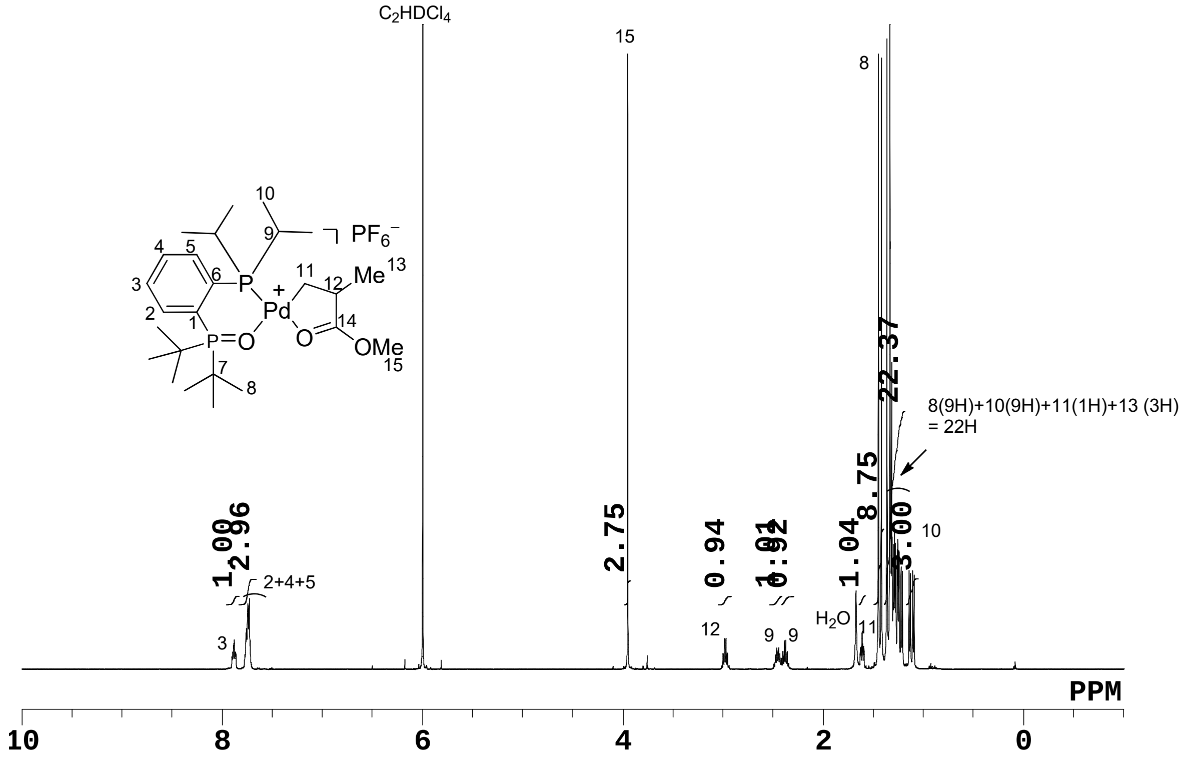

Figure S37. ^{1}H NMR spectrum ($C_2D_2Cl_4$, 500 MHz) of **4a**.

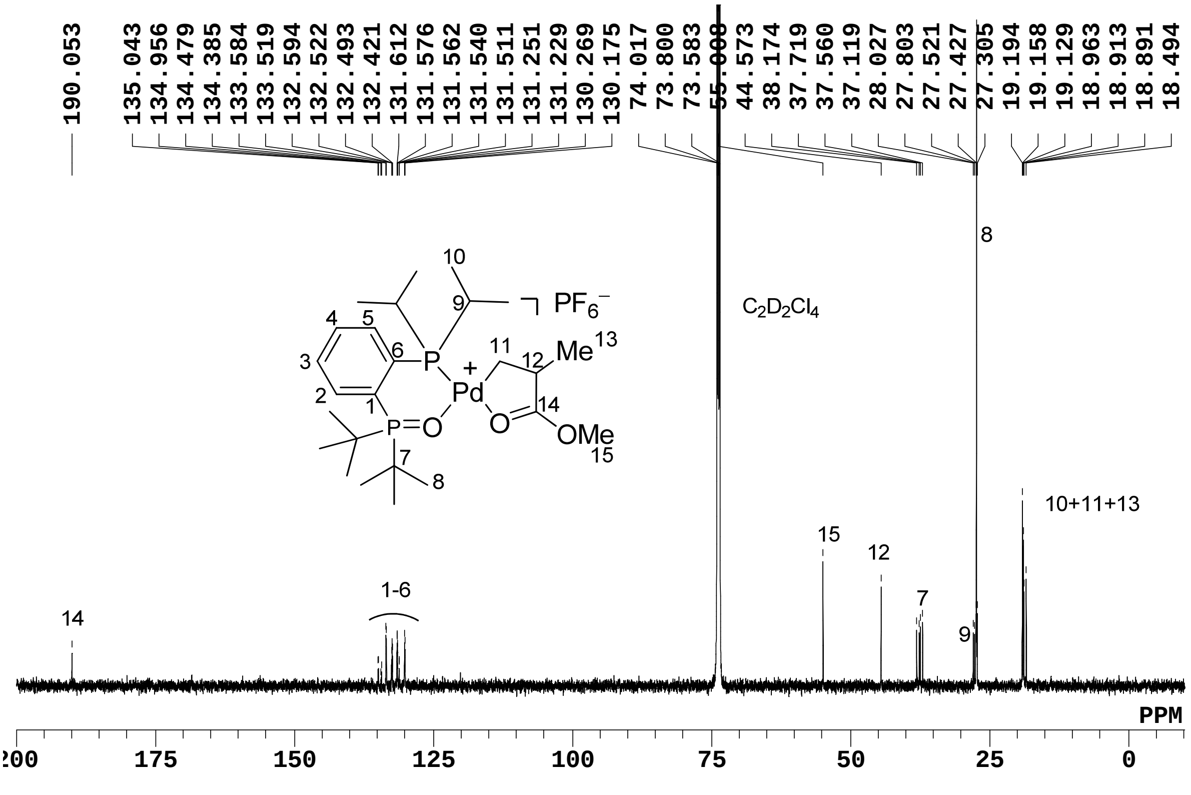

Figure S38. $^{13}C\{^1H\}$ NMR spectrum ($C_2D_2Cl_4$, 126 MHz) of **4a**.

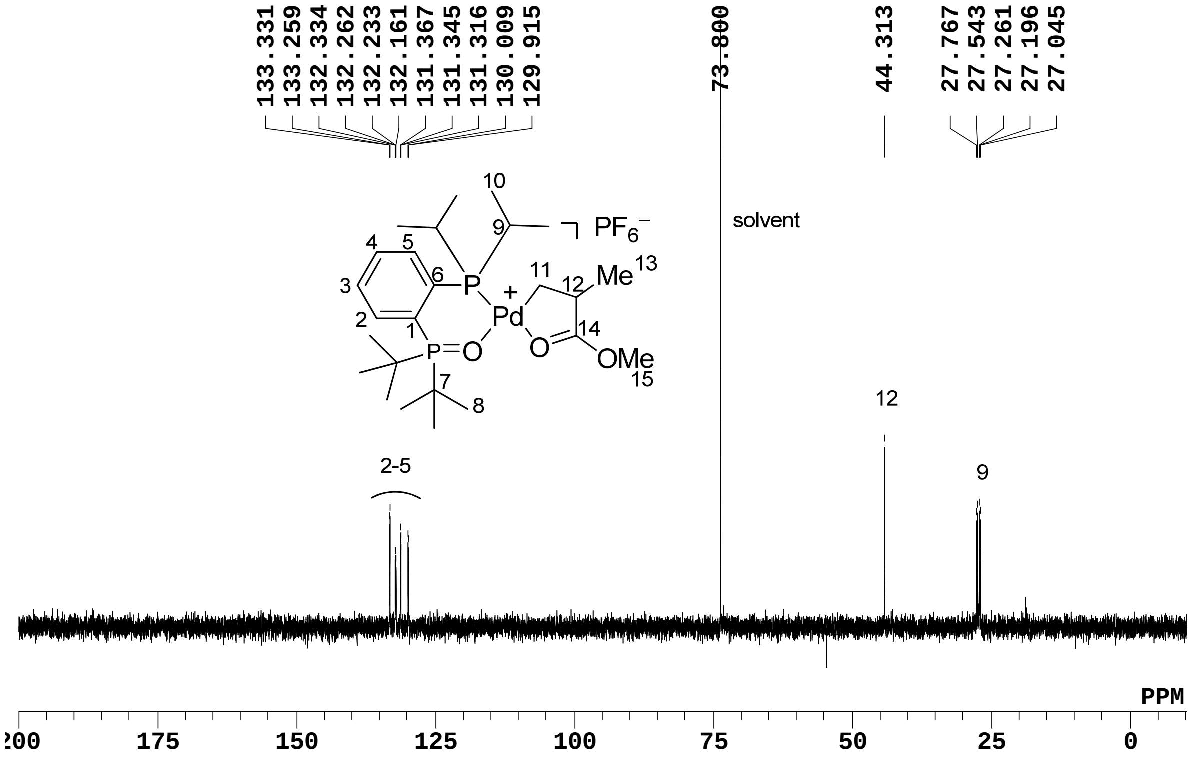

Figure S39. ^{13}C DEPT90 spectrum ($C_2D_2Cl_4$, 126 MHz) of **4a**.

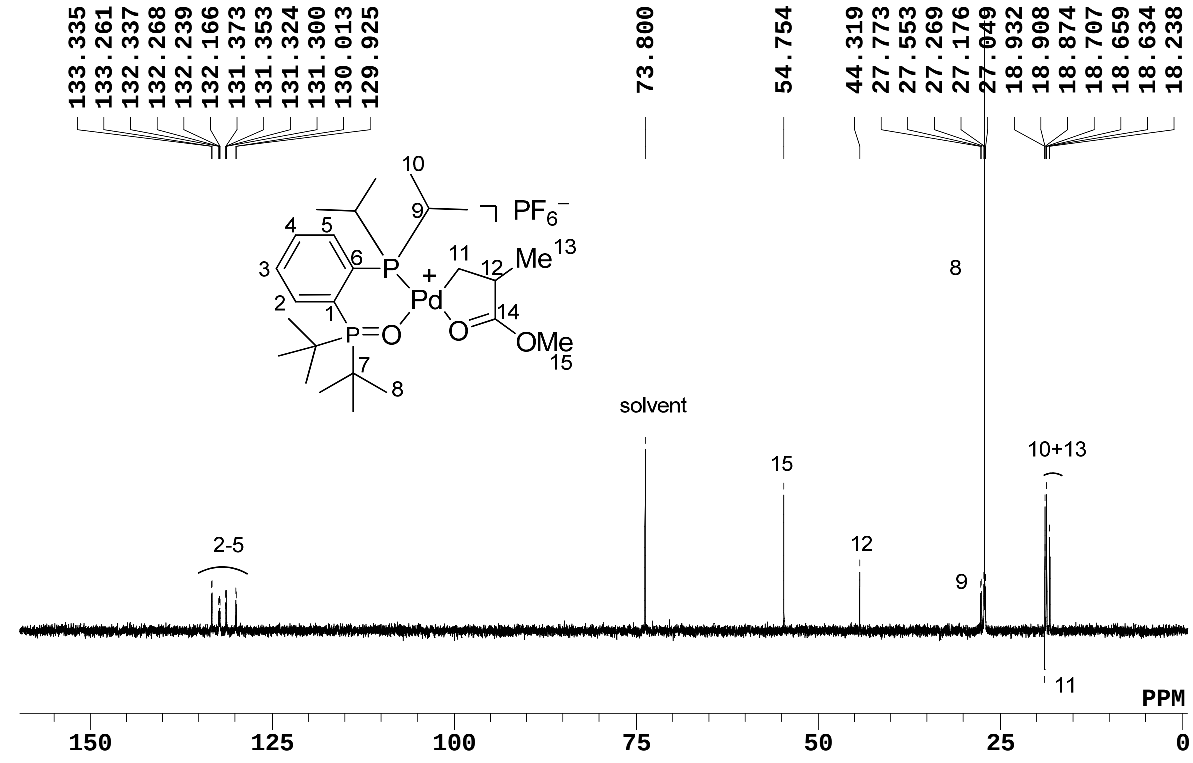

Figure S40. ^{13}C DEPT135 spectrum ($C_2D_2Cl_4$, 126 MHz) of **4a**.

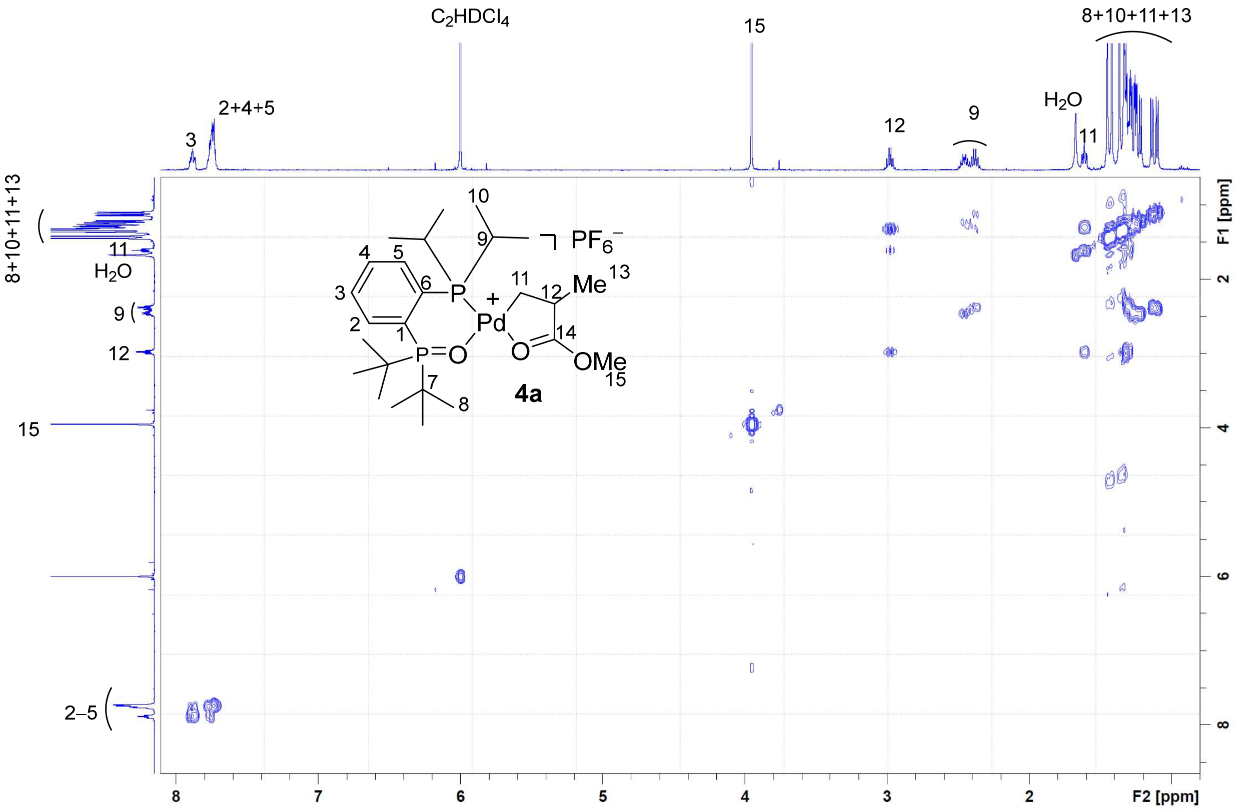

Figure S41. ^{1}H–^{1}H COSY spectrum ($C_2D_2Cl_4$, 500 MHz) of **4a**.

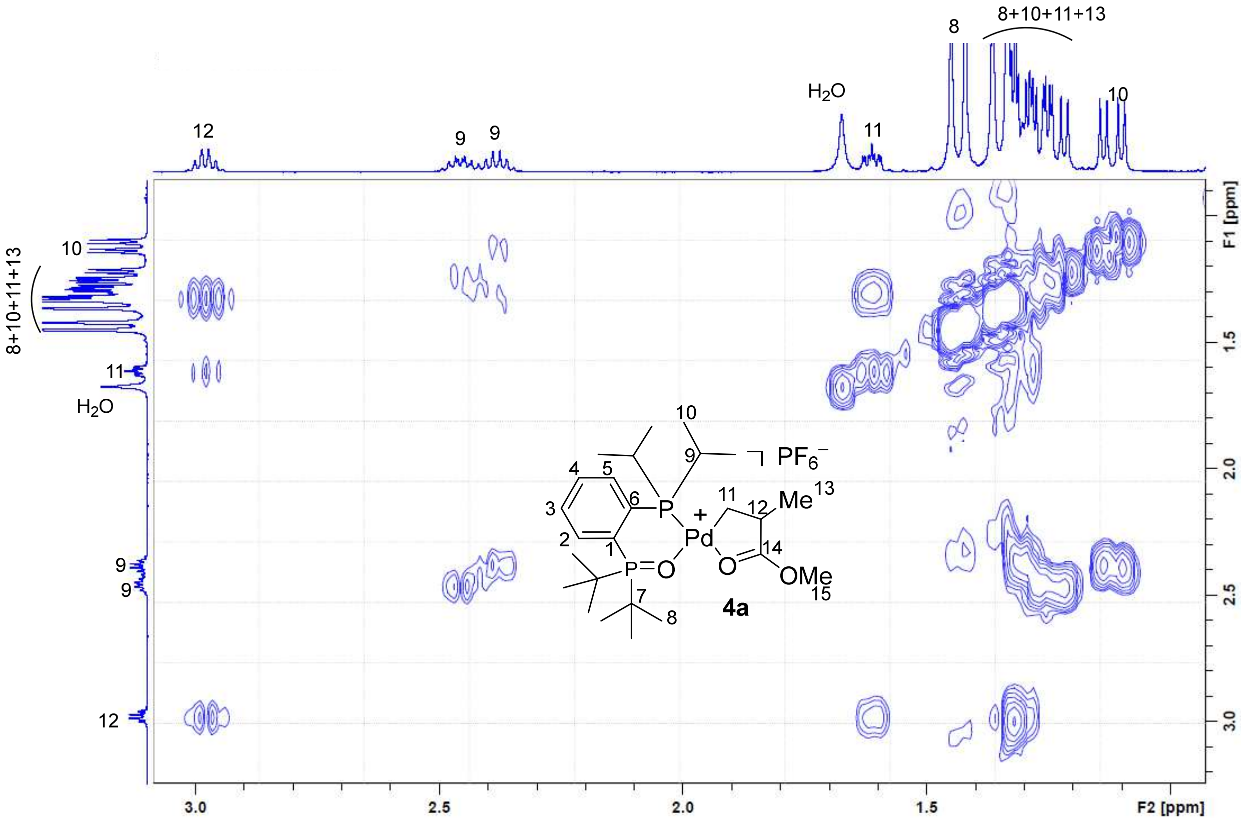

Figure S42. High field region of ^{1}H–^{1}H COSY spectrum ($C_2D_2Cl_4$, 500 MHz) of **4a**.

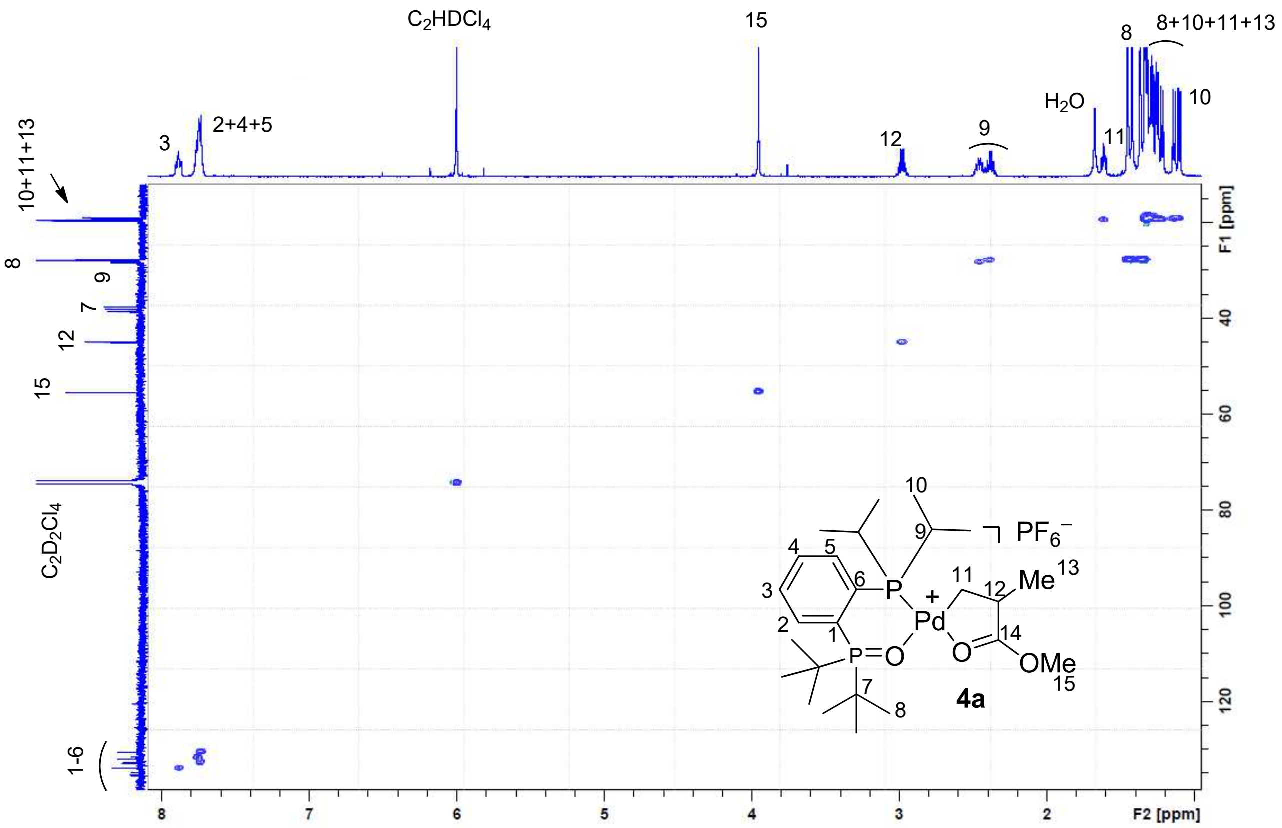

Figure S43. ^{1}H–^{13}C HSQC spectrum ($C_2D_2Cl_4$, 500 MHz for ^{1}H, 126 MHz for ^{13}C) of **4a.**

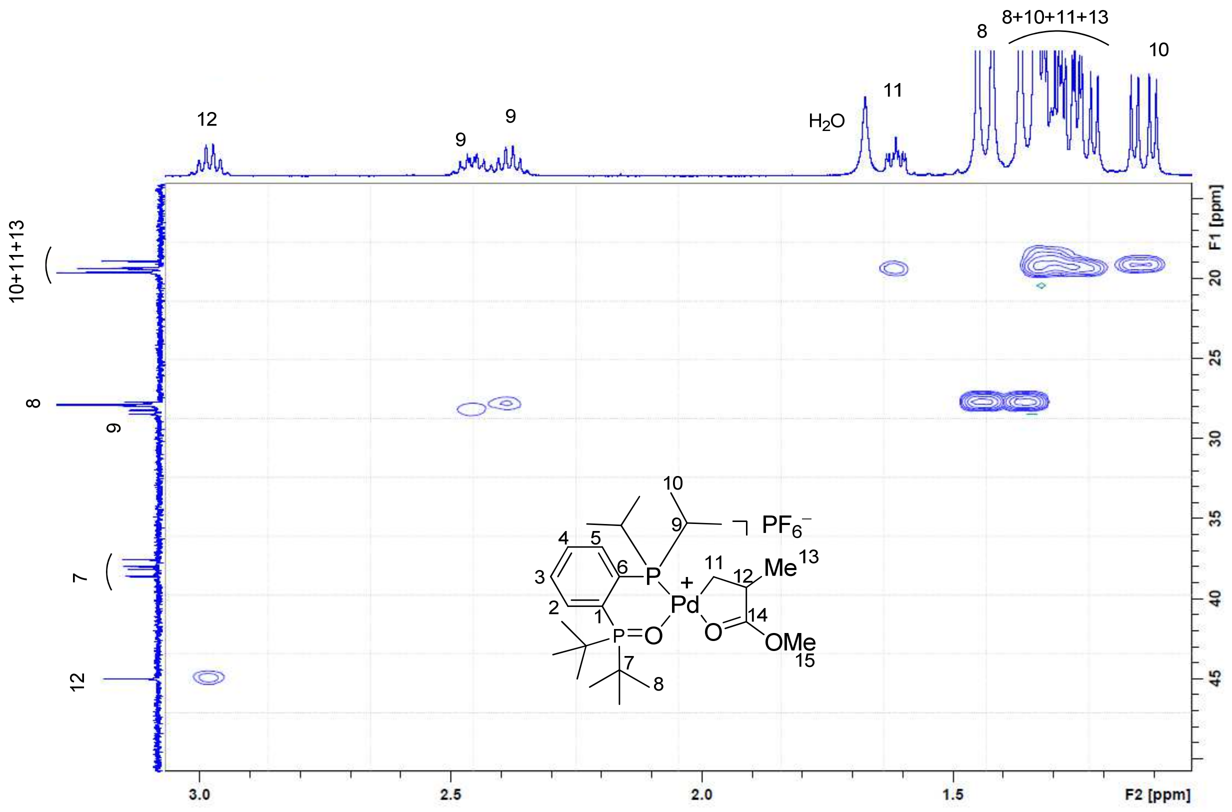

Figure S44. High field region of ^{1}H–^{13}C HSQC spectrum ($C_2D_2Cl_4$, 500 MHz for ^{1}H, 126 MHz for ^{13}C) of **4a**.

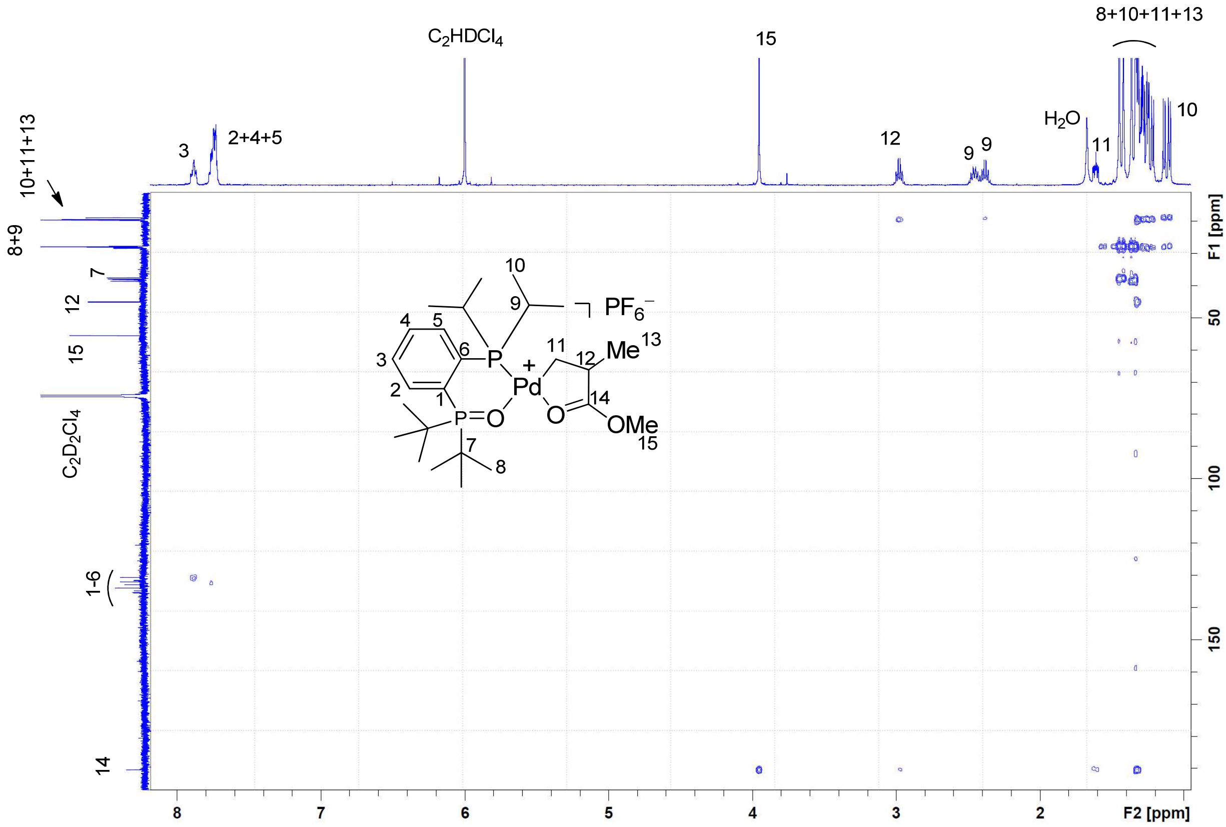

Figure S45. ^{1}H, ^{13}C HMBC spectrum ($C_2D_2Cl_4$, 500 MHz for ^{1}H, 126 MHz for ^{13}C) of **4a**.

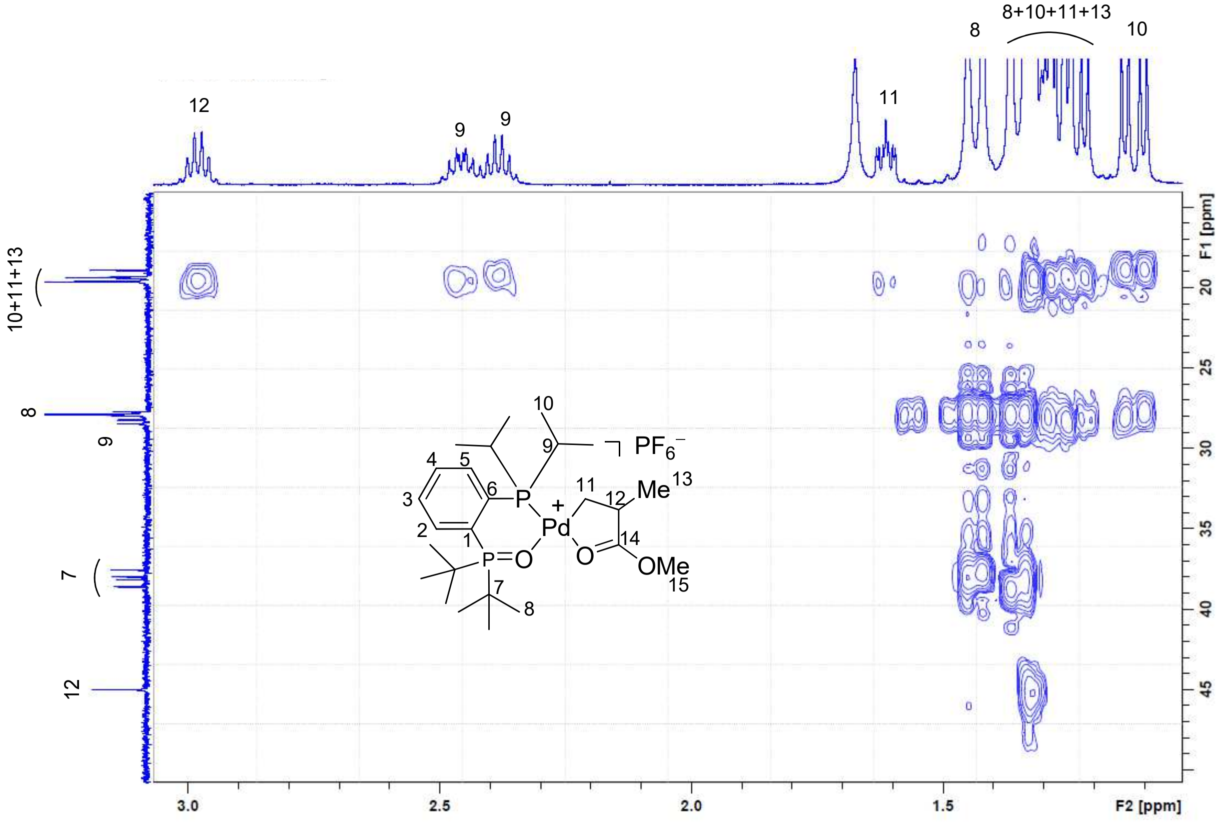

Figure S46. High field region of ^{1}H, ^{13}C HMBC spectrum ($C_2D_2Cl_4$, 500 MHz for ^{1}H, 126 MHz for ^{13}C) of **4a**.

4-2 NMR Spectra of 5a

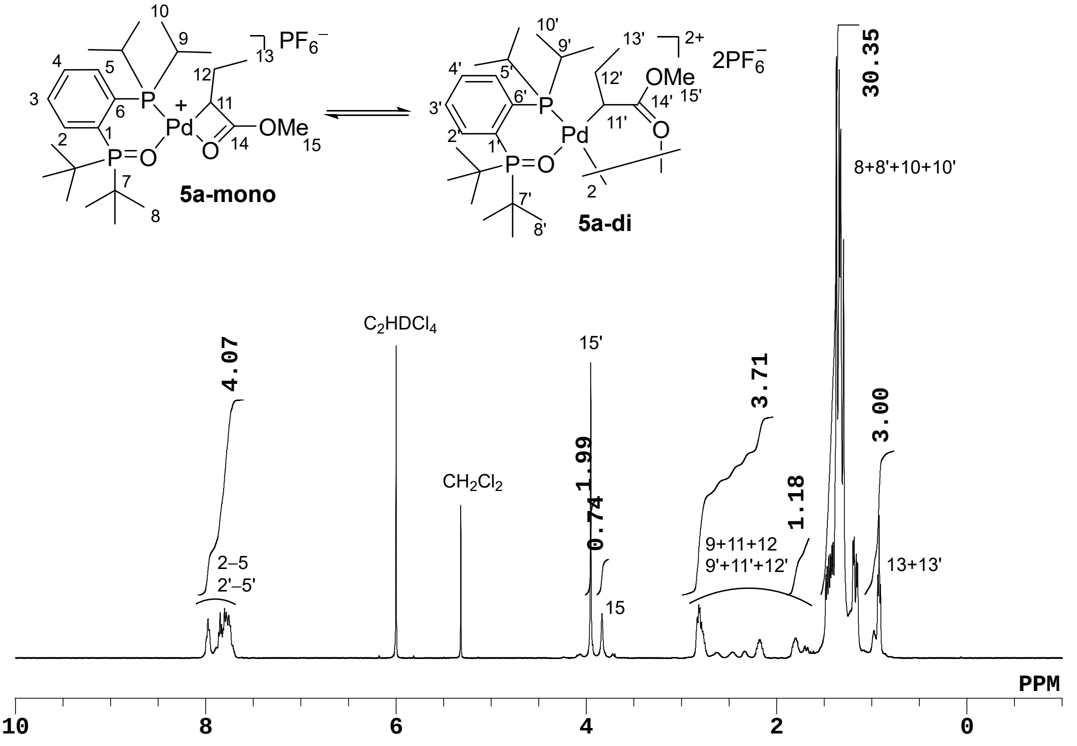

Figure S47. ^{1}H NMR spectrum ($C_2D_2Cl_4$, 500 MHz) of **5a** (3.0×10^{-2} M) at 5 °C. Equilibrium between the monomer (**5a-mono**) and its dimer state (**5a-di**) exists.

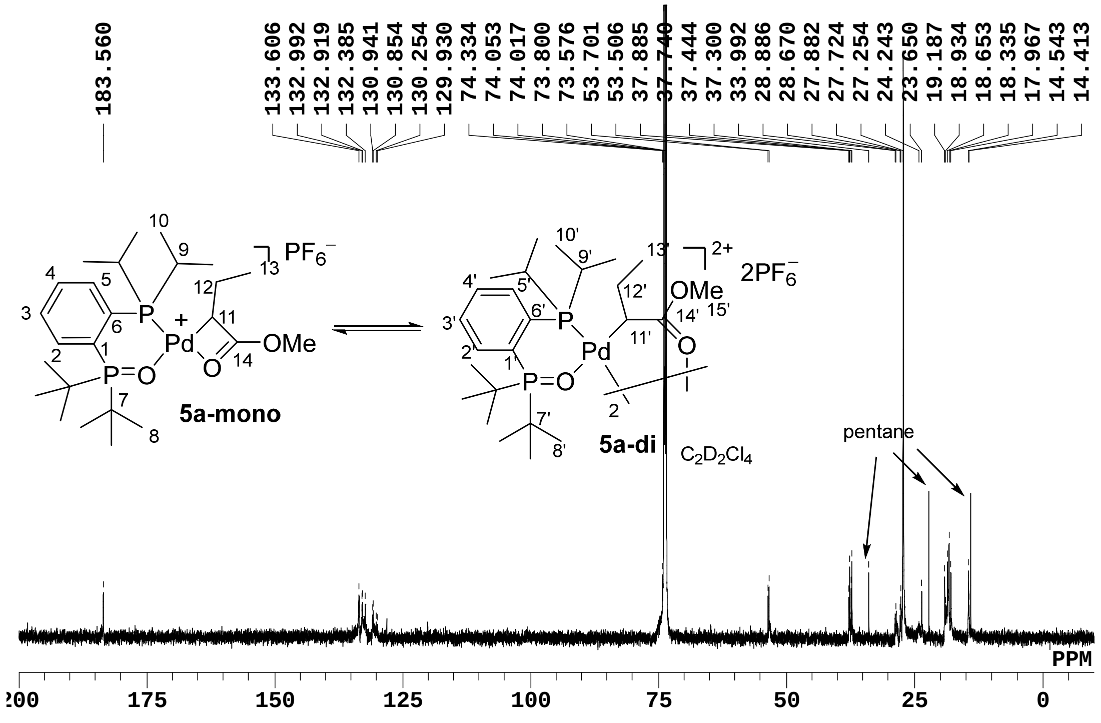

Figure S48. $^{13}C\{^{1}H\}$ NMR spectrum ($C_2D_2Cl_4$, 500 MHz) of **5a**. Equilibrium between the monomer (**5a-mono**) and its dimer state (**5a-di**) exists.

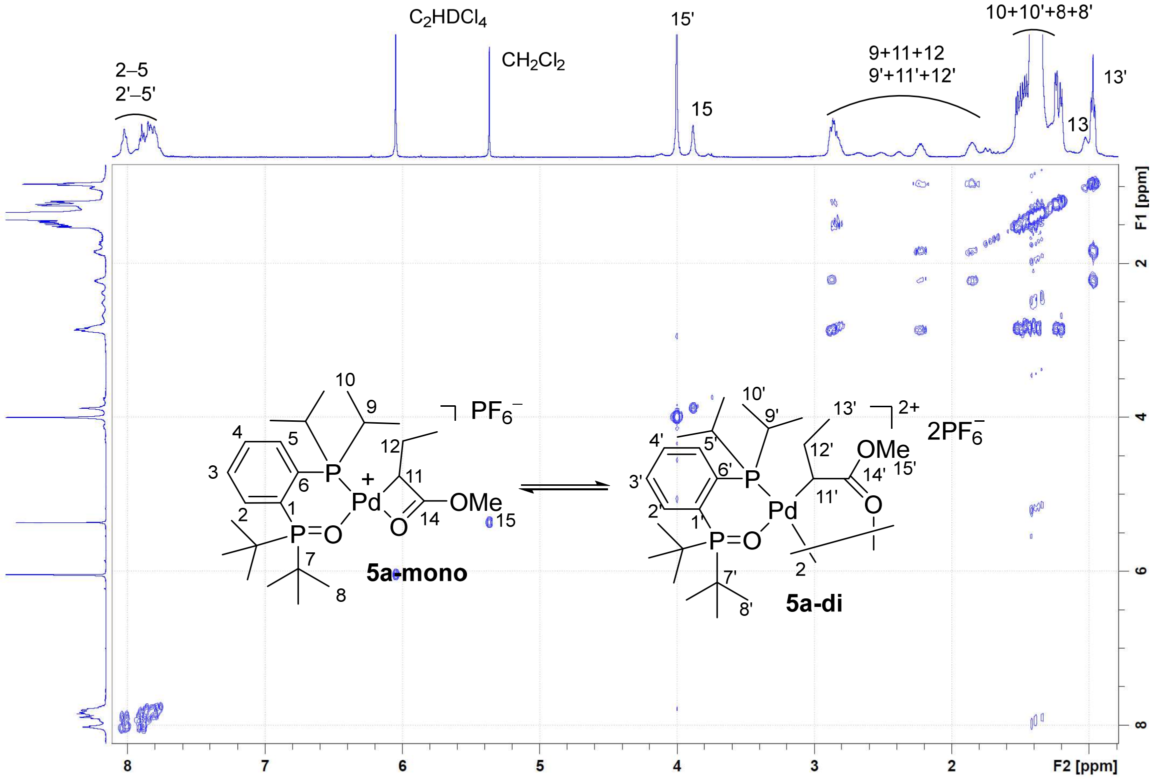

Figure S49. ^{1}H–^{1}H COSY spectrum ($C_2D_2Cl_4$, 500 MHz) of **5a** (3.0×10^{-2} M) at 5 °C. Equilibrium between the monomer (**5a-mono**) and its dimer state (**5a-di**) exists.

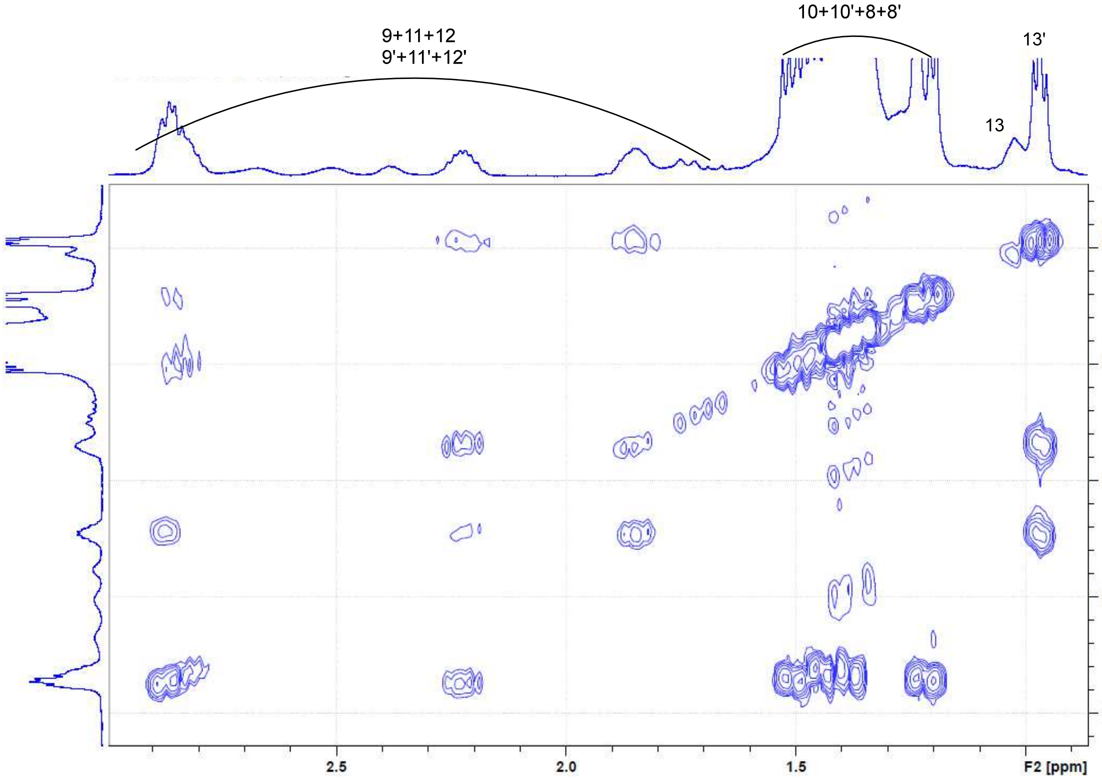

Figure S50. High field region of ^{1}H–^{1}H COSY ($C_2D_2Cl_4$, 500 MHz) spectrum of **5a** (3.0×10^{-2} M) at 5 °C. Equilibrium between the monomer (**5a-mono**) and its dimer state (**5a-di**) exists.

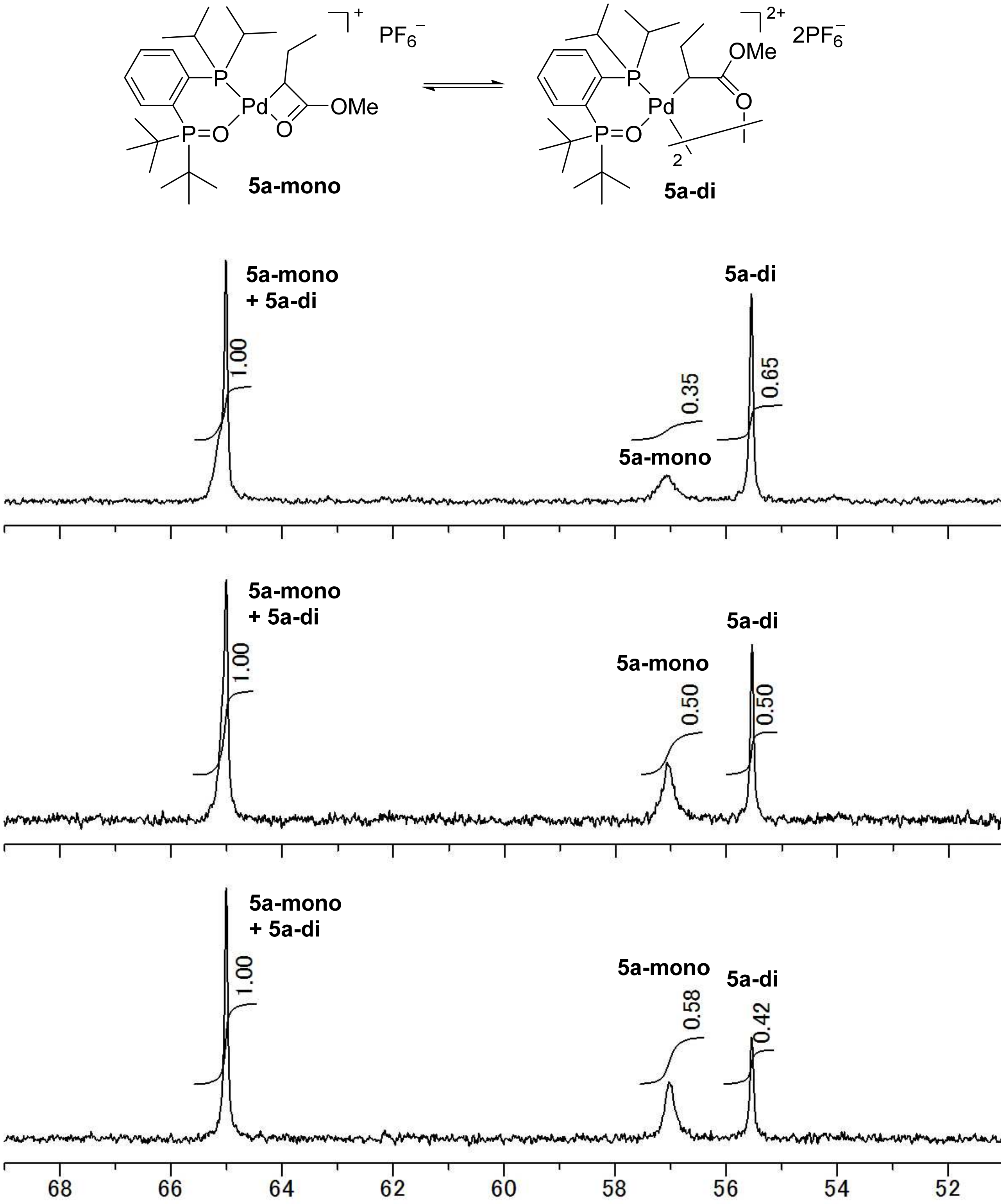

Figure S51. $^{31}P\{^1H\}$ NMR spectra ($C_2D_2Cl_4$, 202 MHz) of **5a-mono/5a-di**. (a) 3.0×10^{-2} M, (b) 1.5×10^{-2} M, (c) 1.0×10^{-2} M.

Discussion

Three signals observed in Figure S51 were assigned to **5a-mono** and **5a-di** based on the following observations. First, the integral ratio of signals at 57.1 ppm : 55.5 ppm in $C_2D_2Cl_4$ changed as 35:65 (3.0×10^{-2} *M*), 50:50 (1.5×10^{-2} *M*), and 58:42 (1.0×10^{-2} M). If two products or two rotamers exist in the solution and do not interconvert each other on the NMR time scale, the integral ratio of the signals should be constant at any concentration. However, the ratio was dependent on the concentration, suggesting the presence of equilibrium between a monomer and a dimer. Since the monomer state should be favored over its dimer state under dilute conditions, we assigned the signal at 57.1 ppm to **5a-mono** and the signal at 55.5 ppm to **5a-di**. Second, the addition of pyridine to a solution of **5a** in DCM resulted in the observation of only signals of **5a-py** (Figure S54). This is probably due to the suppression of equilibrium between a monomer and a dimer by the coordination of pyridine. Third, thermolysis of **5a** afforded methyl crotonate in good yield (82%) (Figure S35). ^{1}H–^{1}H COSY spectrum also supports the structure of **5a-mono** and **5a-di**.

4-3 NMR Spectra of 5a-py

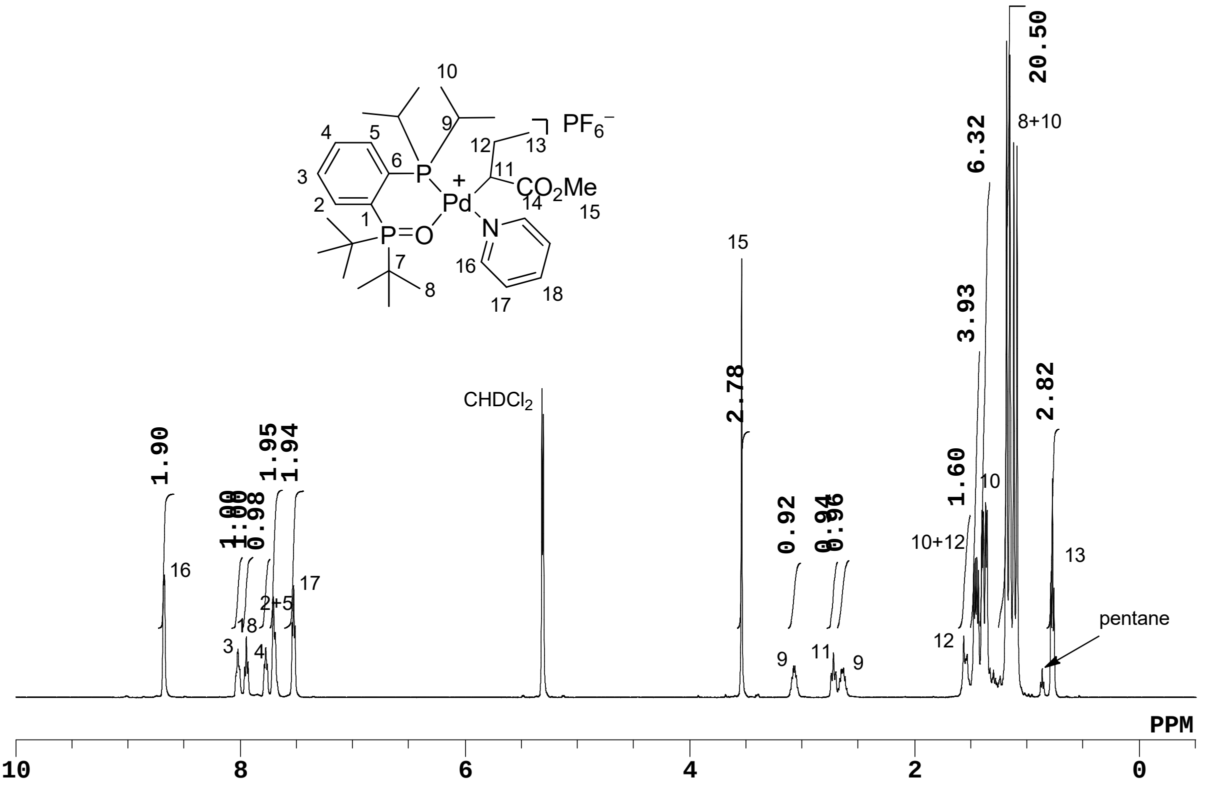

Figure S52. ^{1}H NMR spectrum of **5a-py** (CD_2Cl_2, 500 MHz) in CD_2Cl_2.

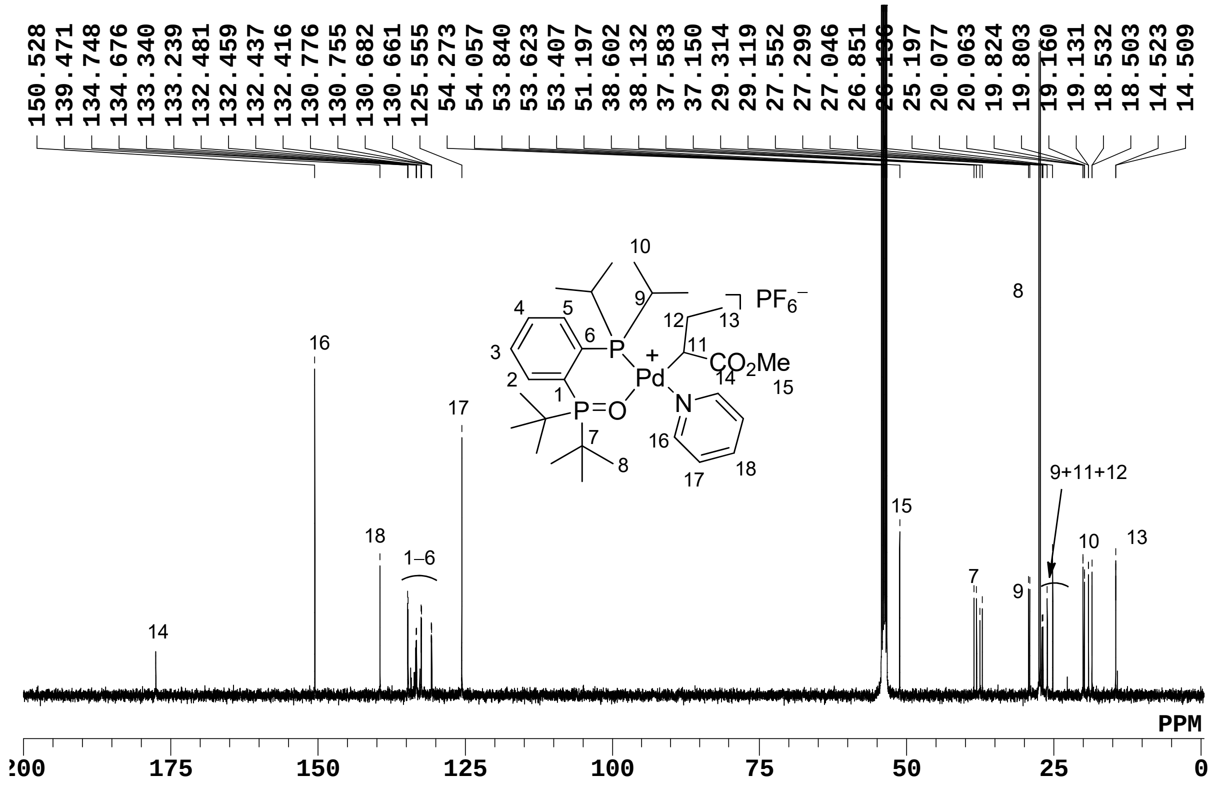

Figure S53. $^{13}C\{^{1}H\}$ NMR spectrum (CD_2Cl_2, 126 MHz) of **5a-py**.

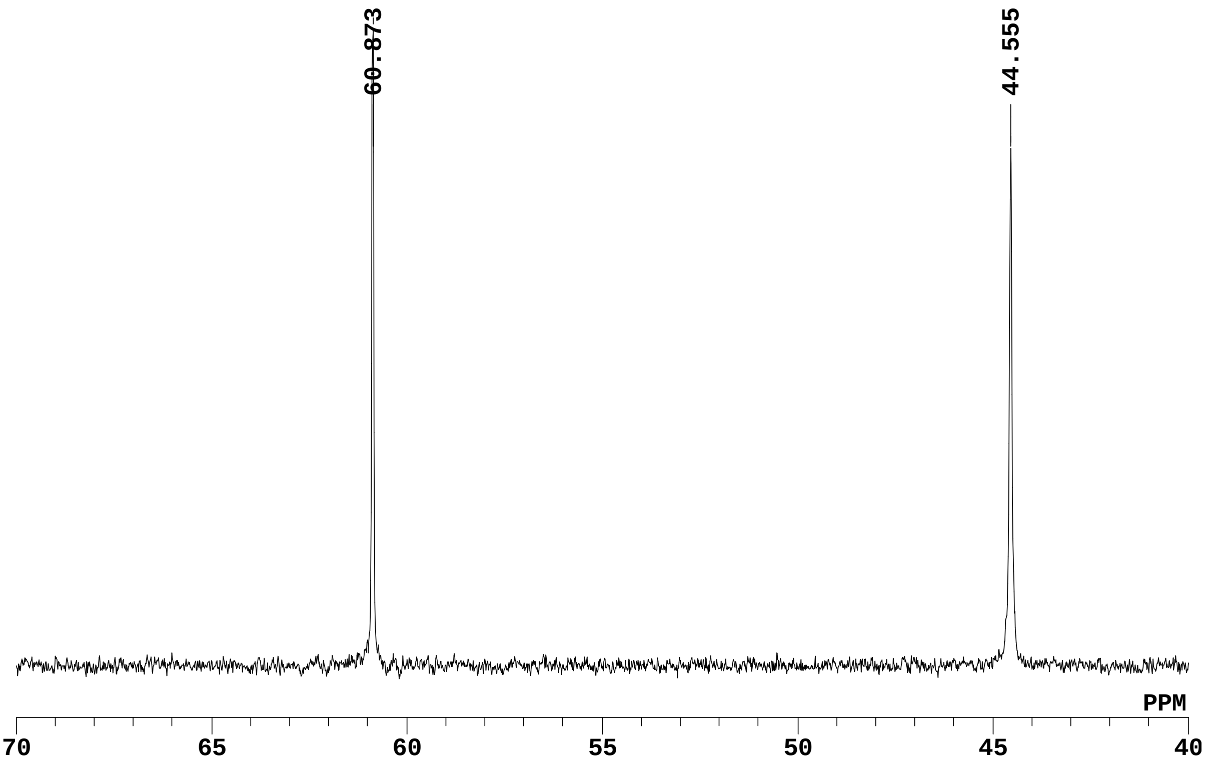

Figure S54. $^{31}P\{^{1}H\}$ NMR spectrum (CD_2Cl_2, 202 MHz) of **5a-py**.

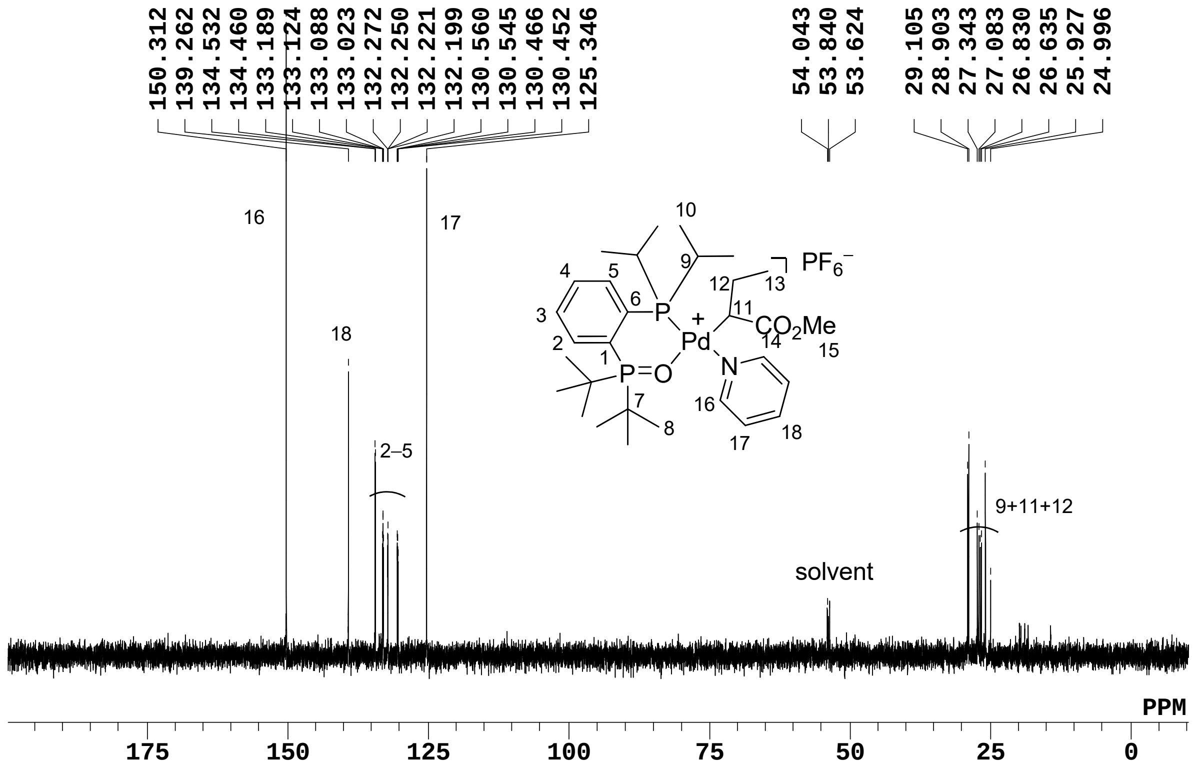

Figure S55. ^{13}C DEPT90 NMR spectrum (CD_2Cl_2, 126 MHz) of **5a-py**.

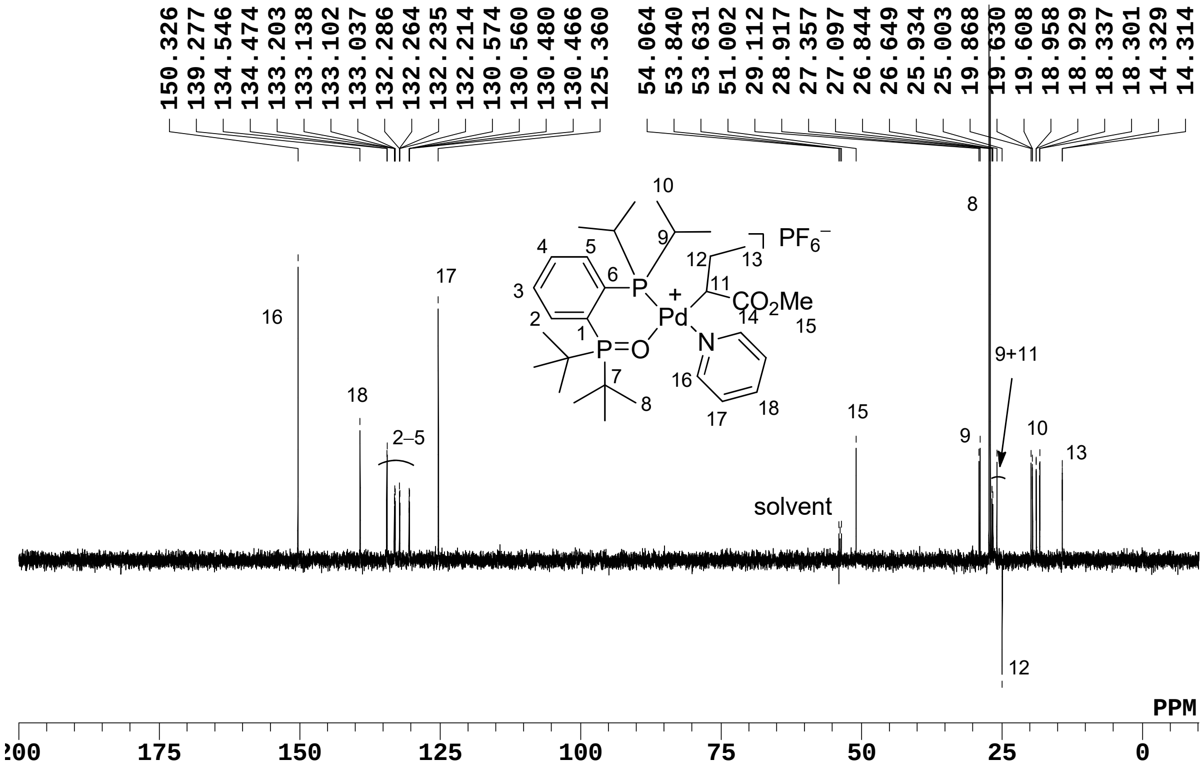

Figure S56. ^{13}C DEPT135 NMR spectrum (CD_2Cl_2, 126 MHz) of **5a-py**.

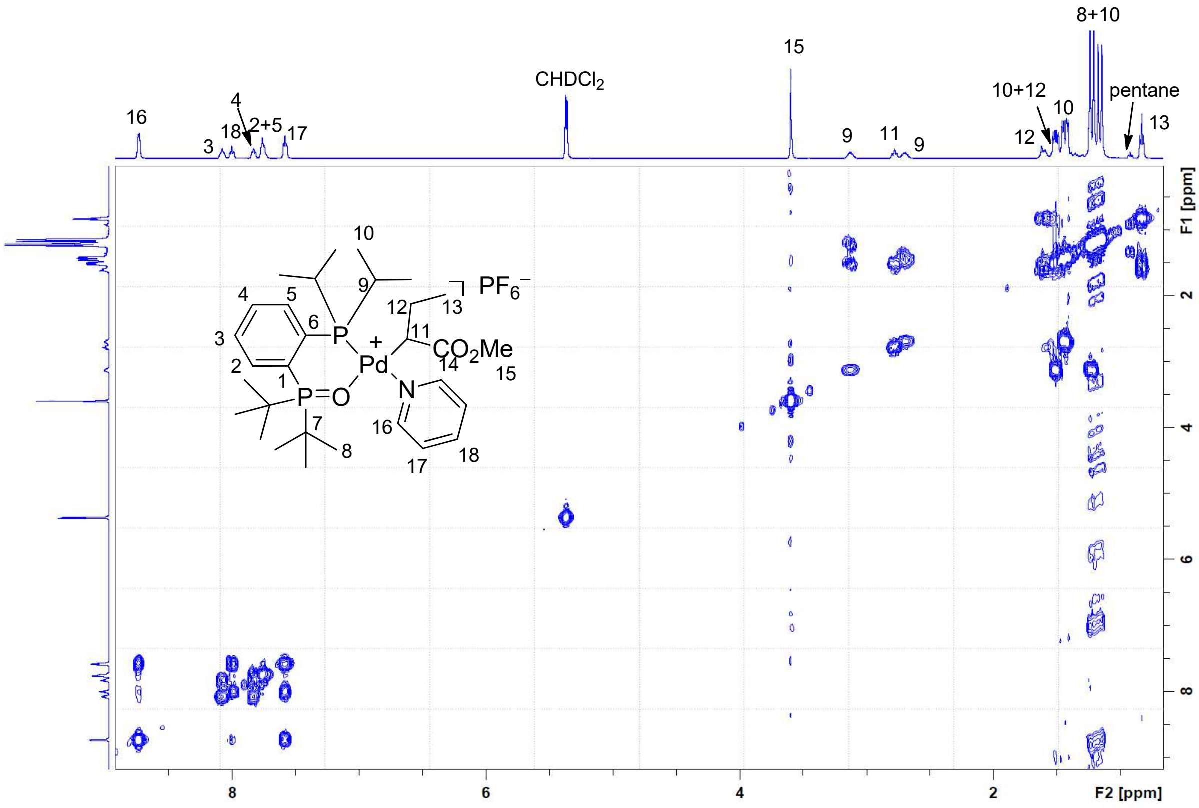

Figure S57. ^{1}H–^{1}H COSY spectrum (CD_2Cl_2, 500 MHz) of **5a-py**.

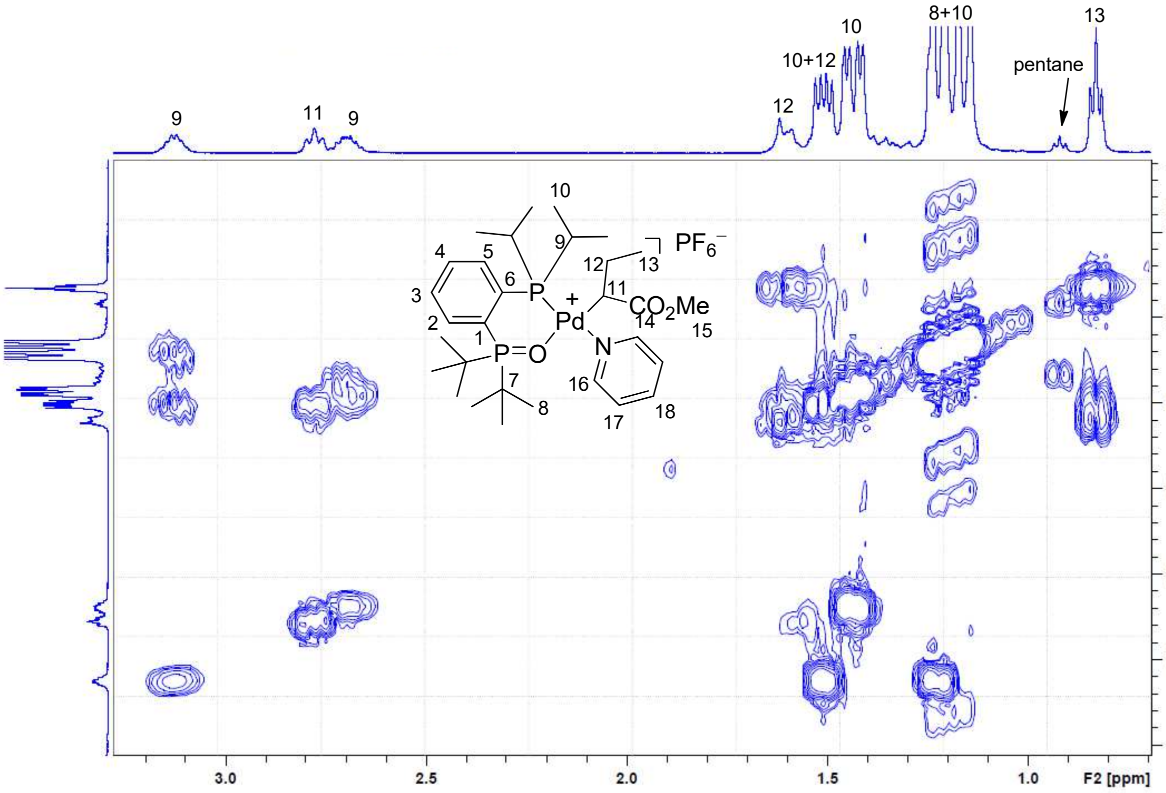

Figure S58. High field region of ^{1}H–^{1}H COSY spectrum (CD_2Cl_2, 500 MHz) of **5a-py**.

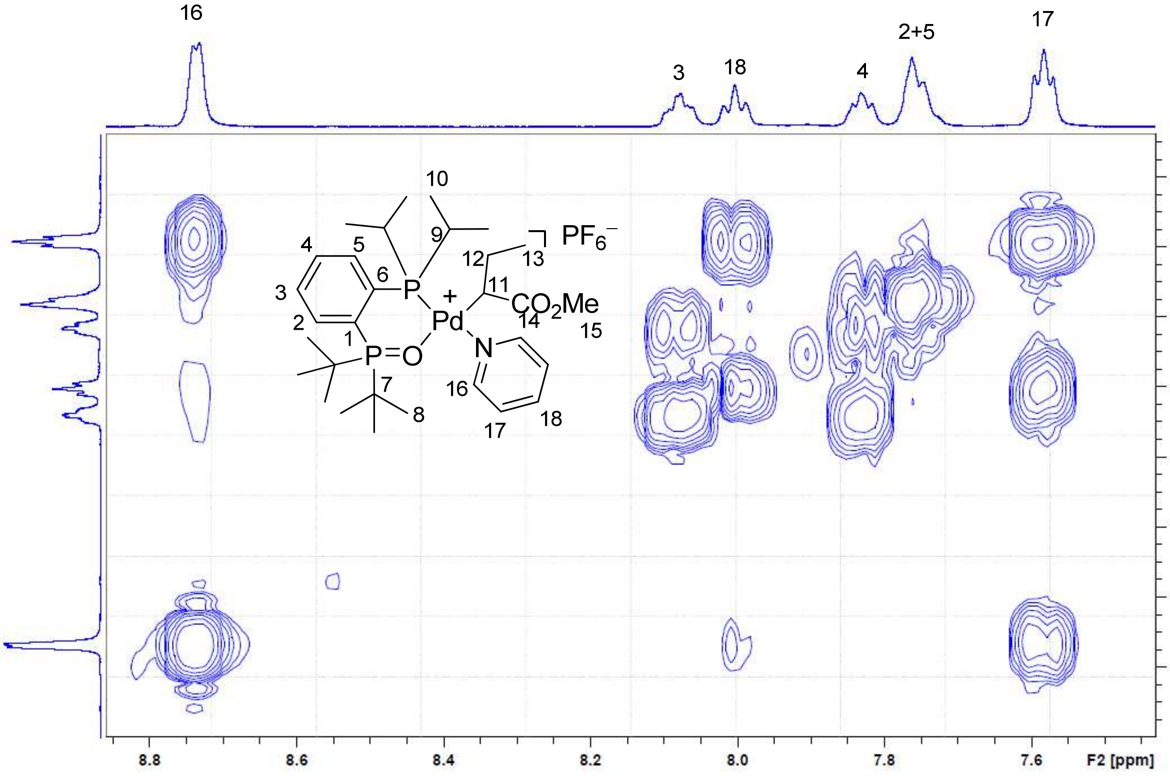

Figure S59. Low field region of ^{1}H–^{1}H COSY spectrum (CD_2Cl_2, 500 MHz) of **5a-py**.

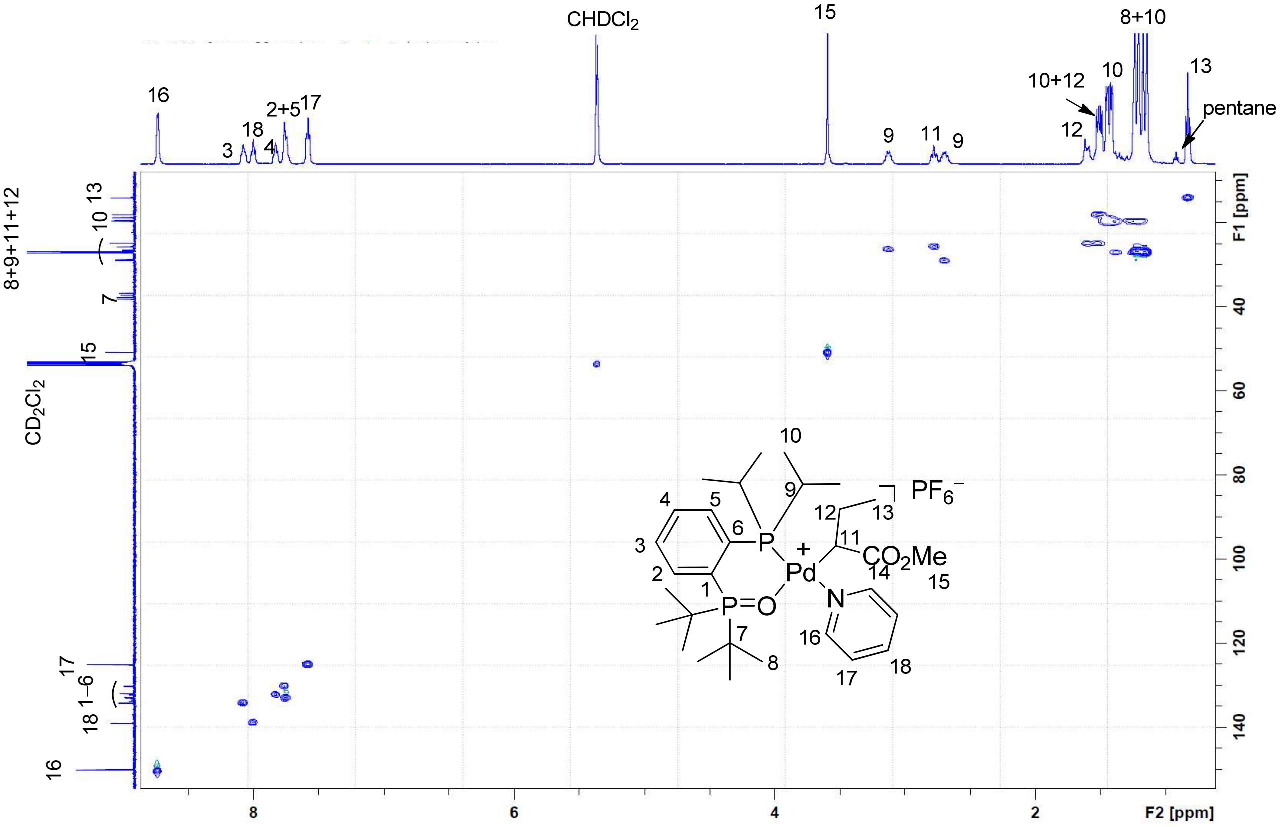

Figure S60. ^1H–^{13}C HSQC spectrum (CD_2Cl_2, 500 MHz for ^1H, 126 MHz for ^{13}C) of **5a-py**.

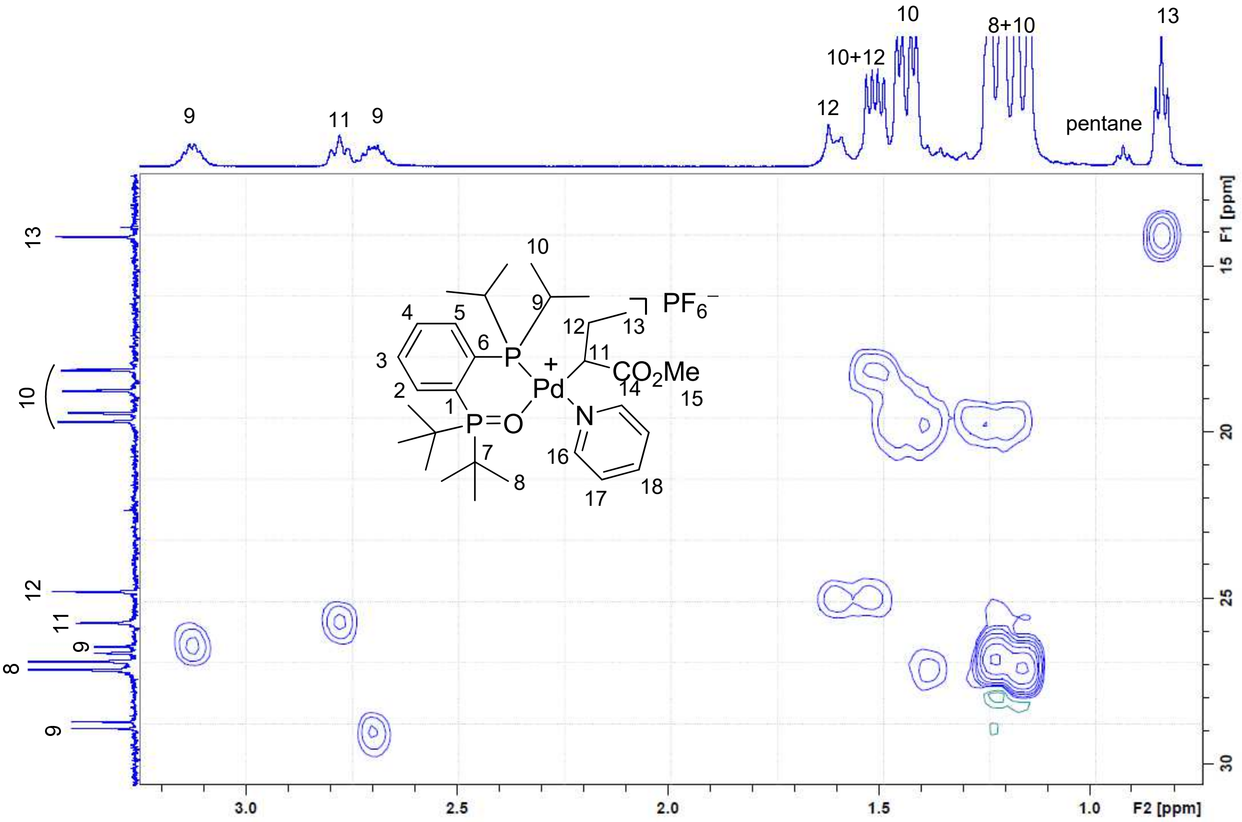

Figure S61. High field region of ^1H–^{13}C HSQC spectrum (CD_2Cl_2, 500 MHz for ^1H, 126 MHz for ^{13}C) of **5a-py**.

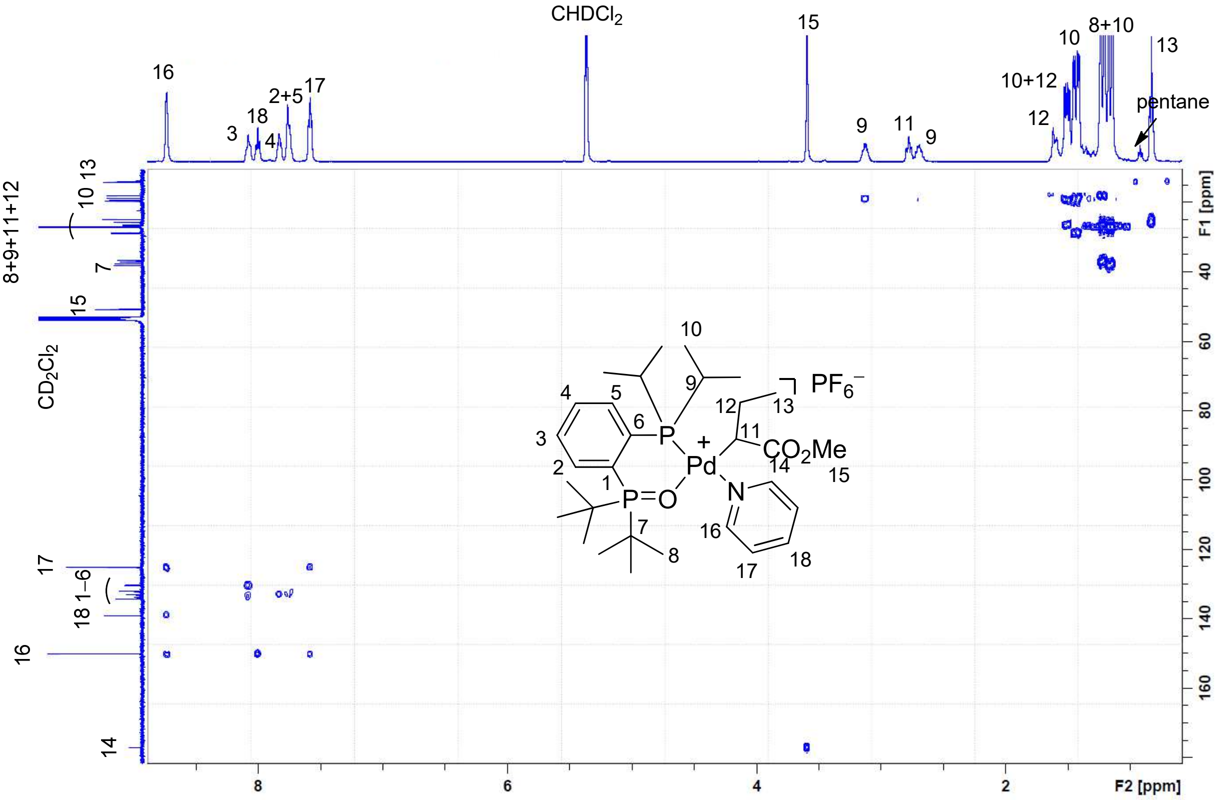

Figure S62. ^{1}H, ^{13}C HMBC spectrum (CD_2Cl_2, 500 MHz for ^{1}H, 126 MHz for ^{13}C) of **5a-py**.

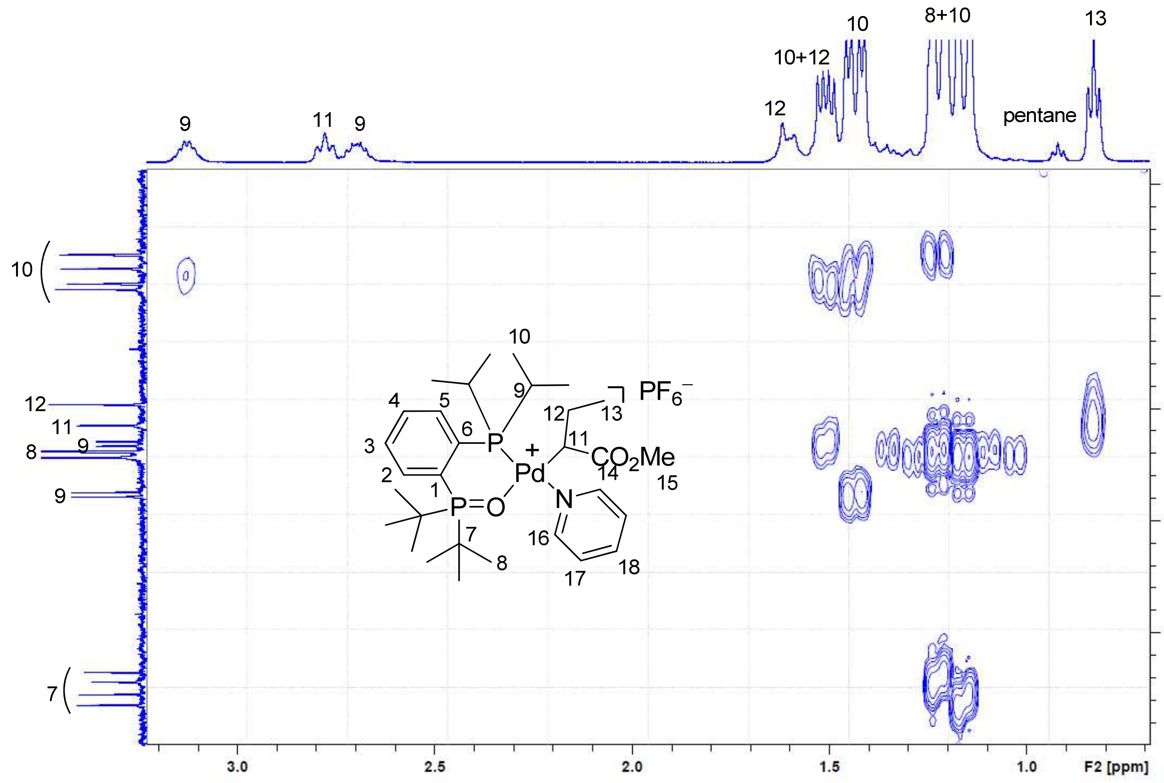

Figure S63. High field region of ^{1}H, ^{13}C HMBC spectrum (CD_2Cl_2, 500 MHz for ^{1}H, 126 MHz for ^{13}C) of **5a-py**.

4-4 NMR Spectra of 5c

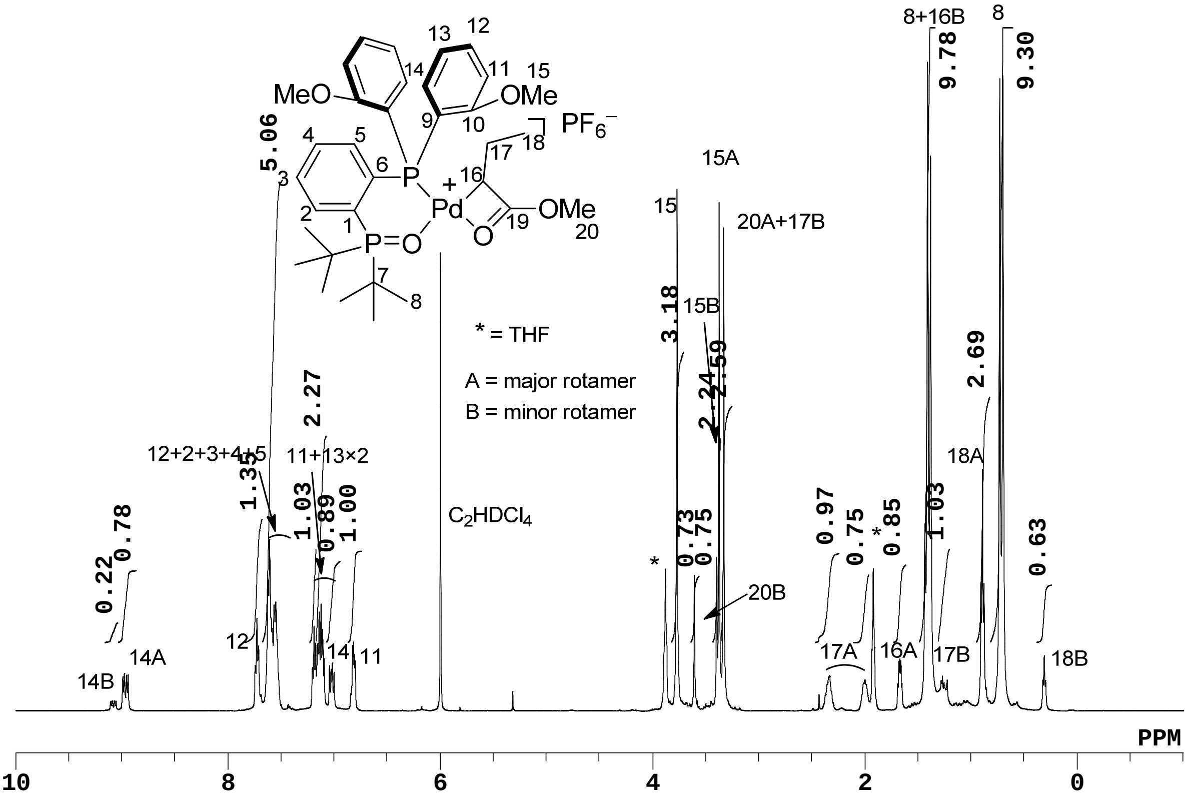

Figure S64. ^{1}H NMR spectrum ($C_2D_2Cl_4$, 500 MHz, 5 °C) of **5c**.

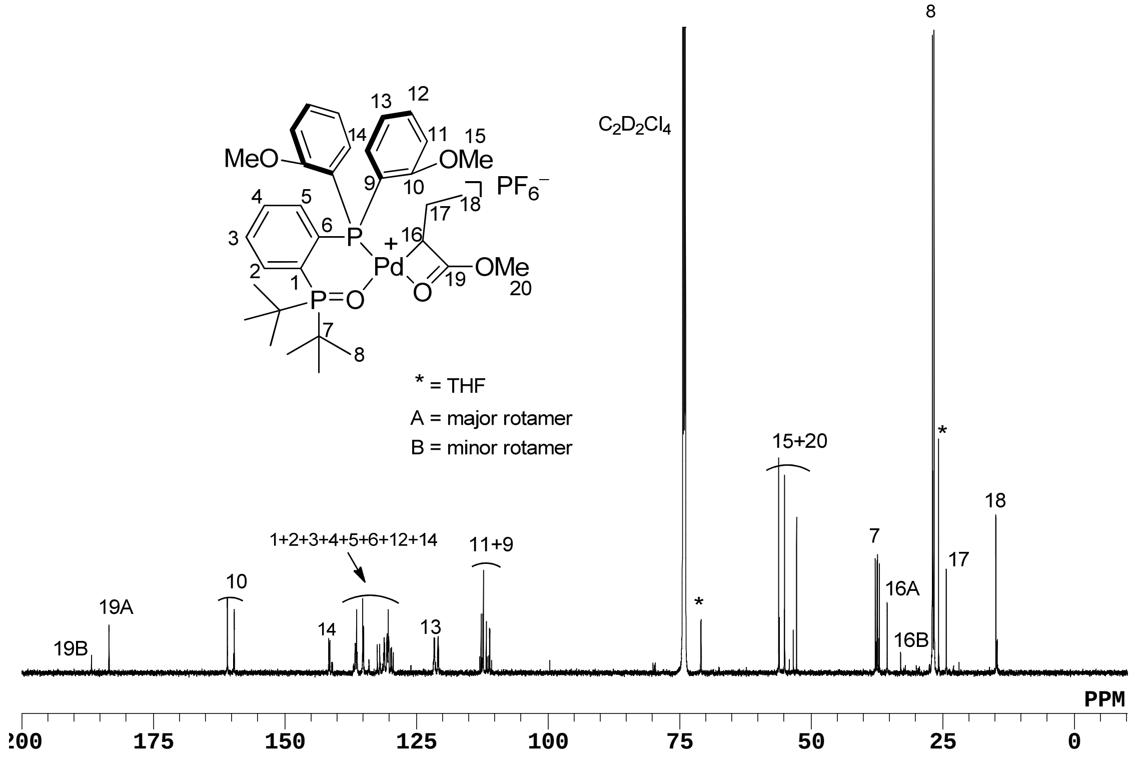

Figure S65. $^{13}C\{^1H\}$ NMR spectrum ($C_2D_2Cl_4$, 126 MHz, 5 °C) of **5c**.

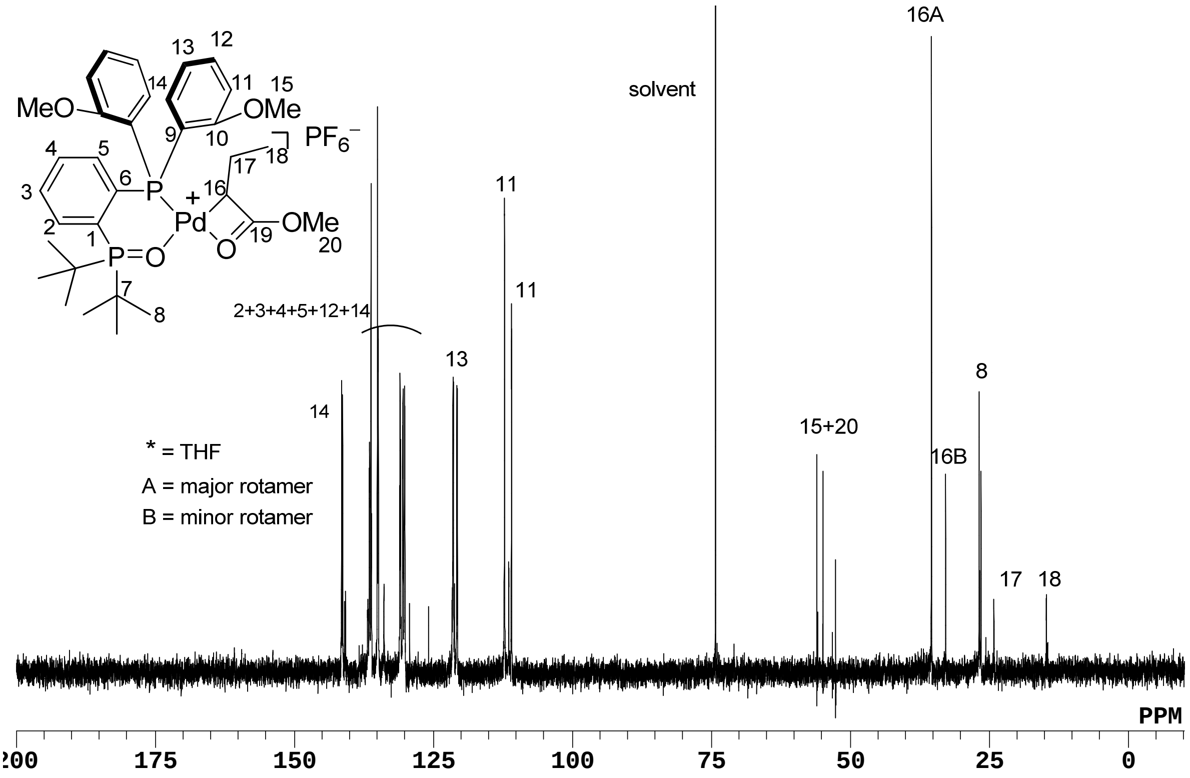

Figure S66. ^{13}C DEPT90 NMR spectrum ($C_2D_2Cl_4$, 126 MHz, 5 °C) of **5c**.

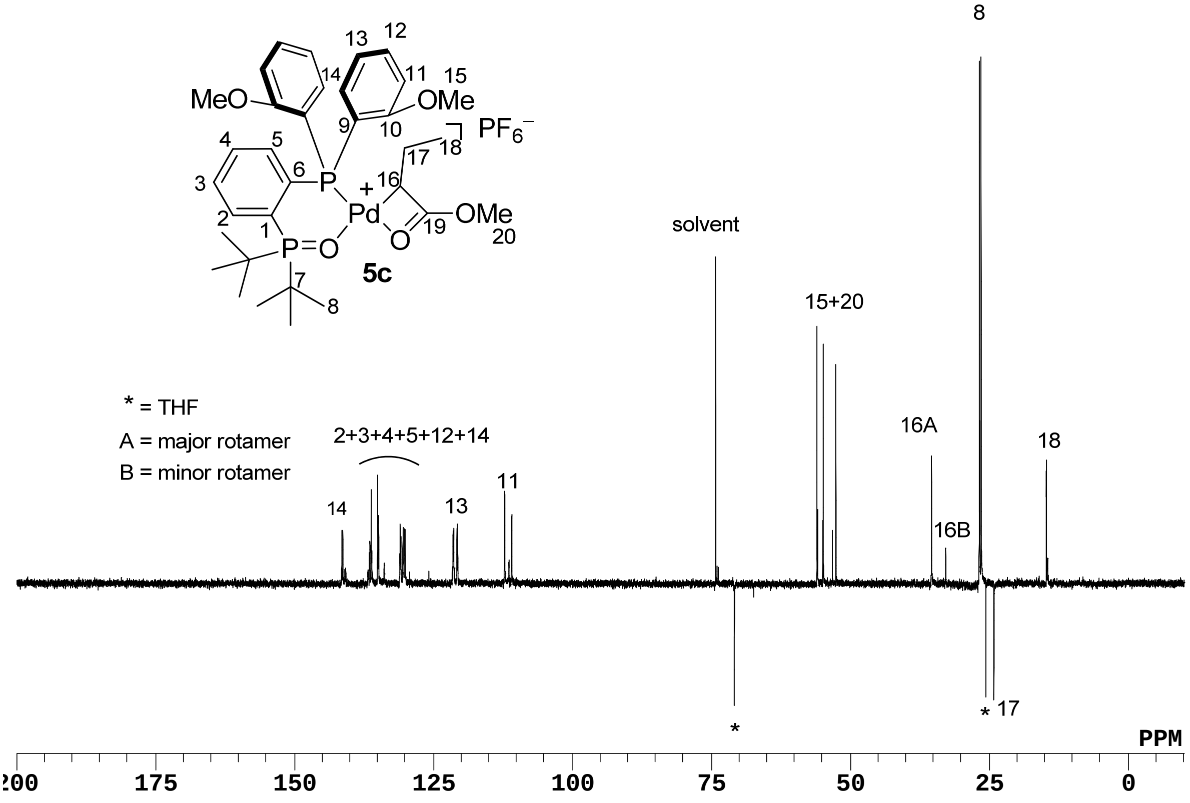

Figure S67. ^{13}C DEPT135 NMR spectrum ($C_2D_2Cl_4$, 126 MHz, 5 °C) of **5c**.

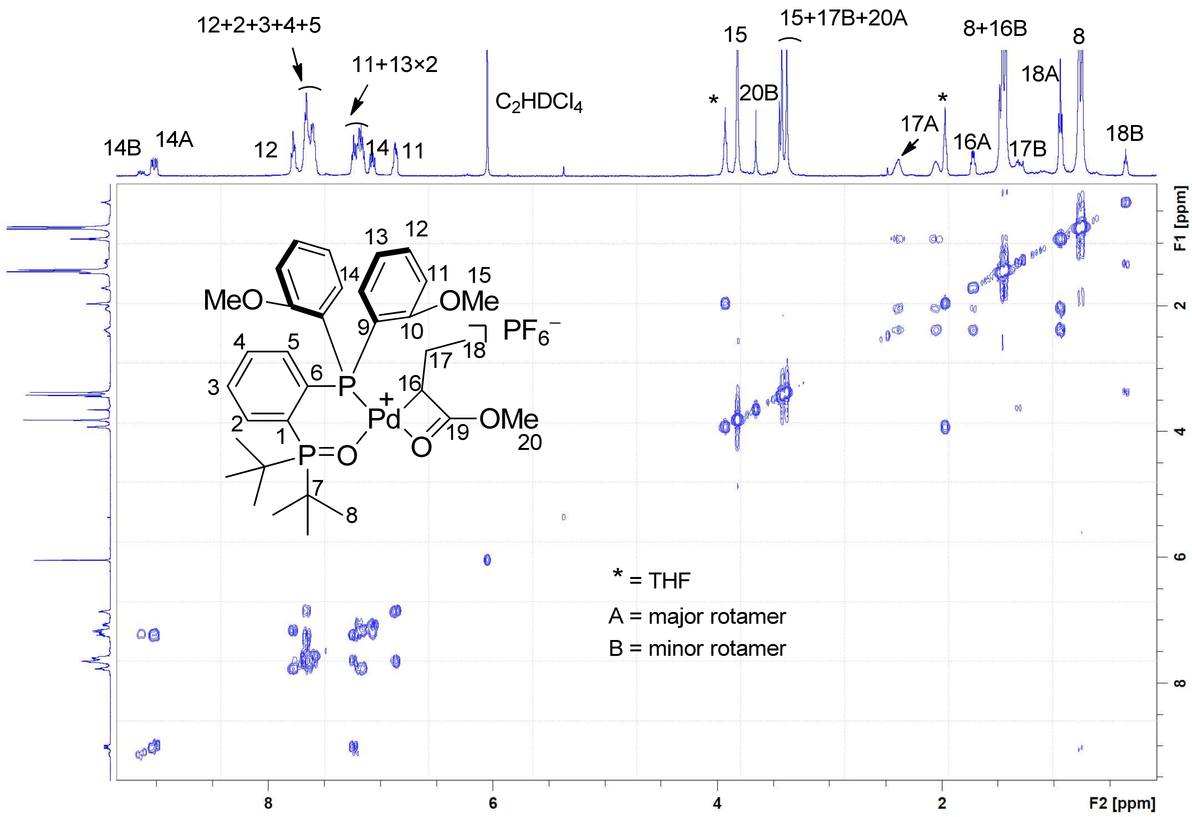

Figure S68. ${}^{1}H$–${}^{1}H$ COSY spectrum ($C_2D_2Cl_4$, 500 MHz, 5 °C) of **5c**.

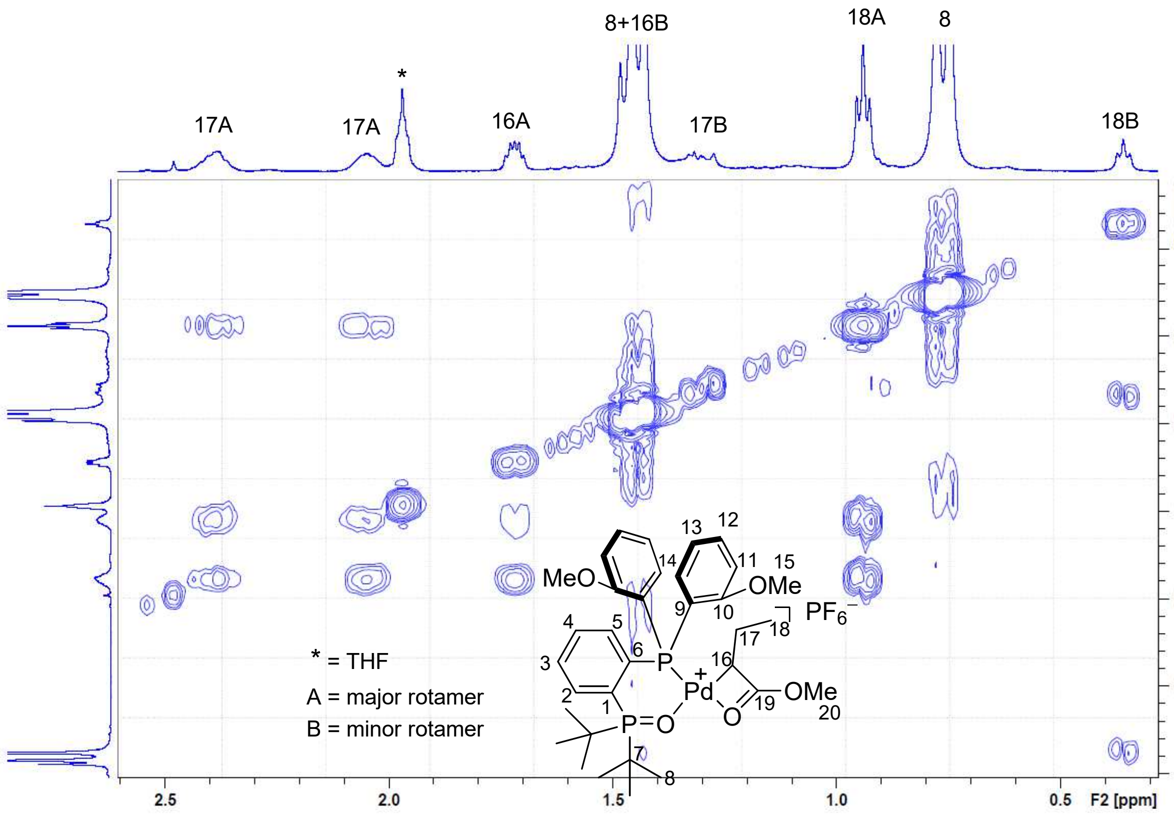

Figure S69. High field region of ${}^{1}H$–${}^{1}H$ COSY spectrum ($C_2D_2Cl_4$, 500 MHz, 5 °C) of **5c**.

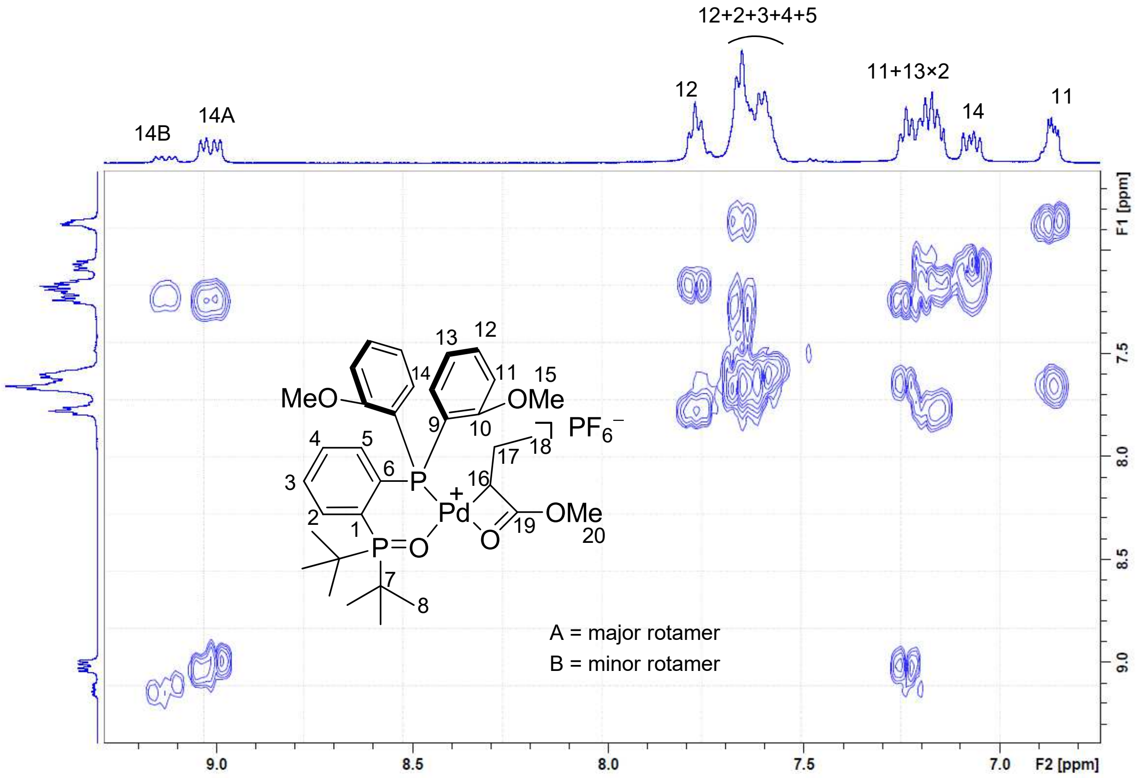

Figure S70. Low field region of ^1H–^1H COSY spectrum ($C_2D_2Cl_4$, 500 MHz, 5 °C) of **5c**.

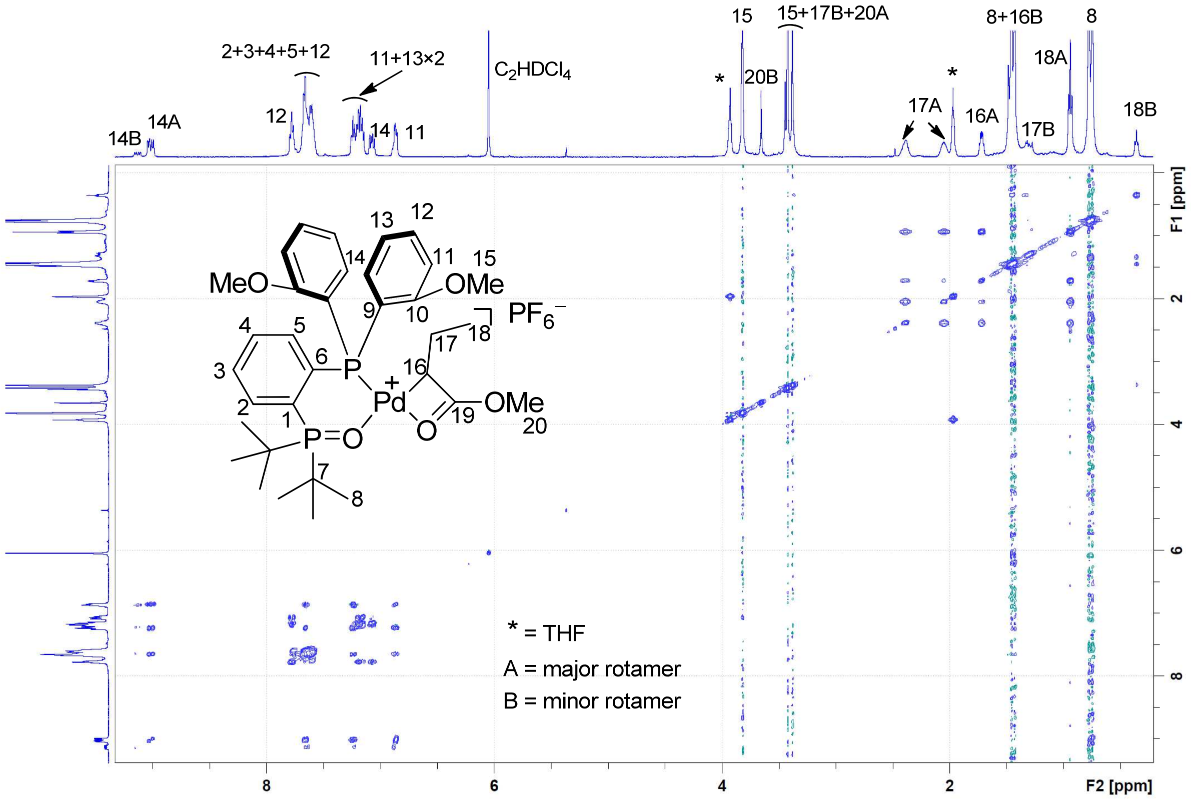

Figure S71. ^1H–^1H TOCSY spectrum ($C_2D_2Cl_4$, 500 MHz, 5 °C) of **5c**.

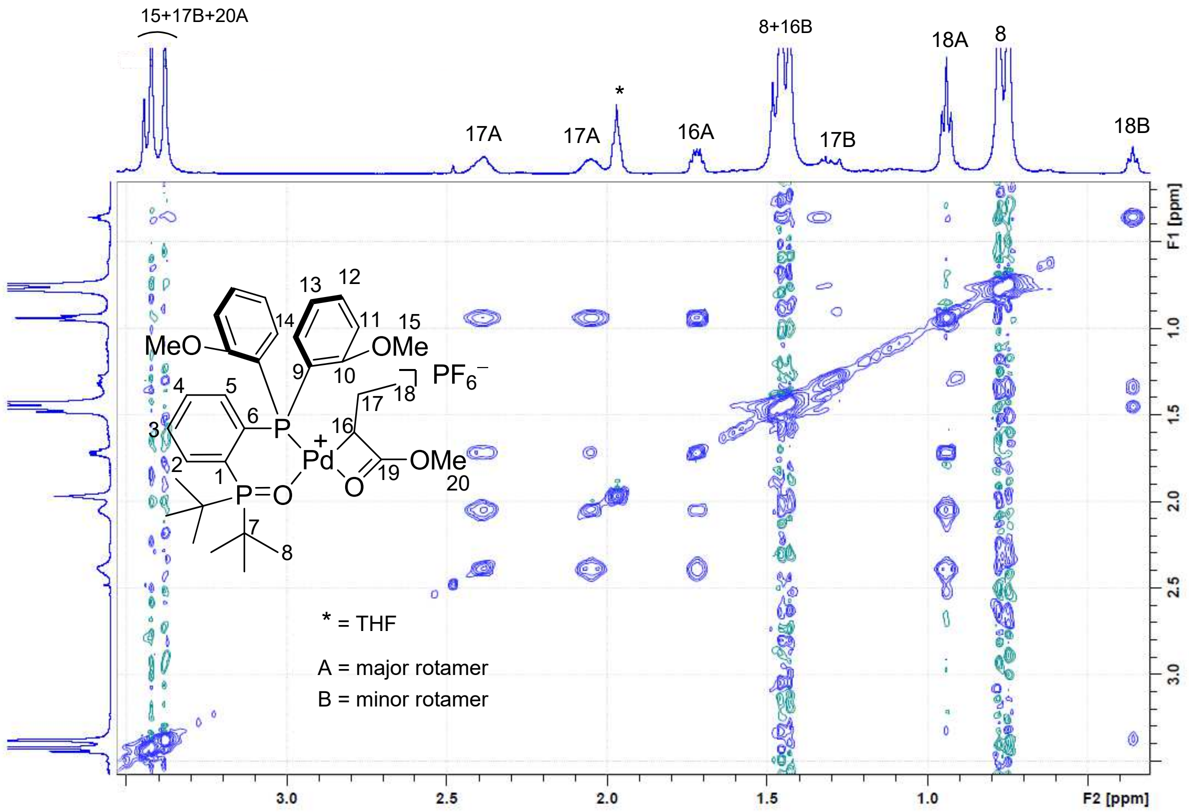

Figure S72. High field region of ^1H–^1H TOCSY spectrum ($C_2D_2Cl_4$, 500 MHz, 5 °C) of **5c**.

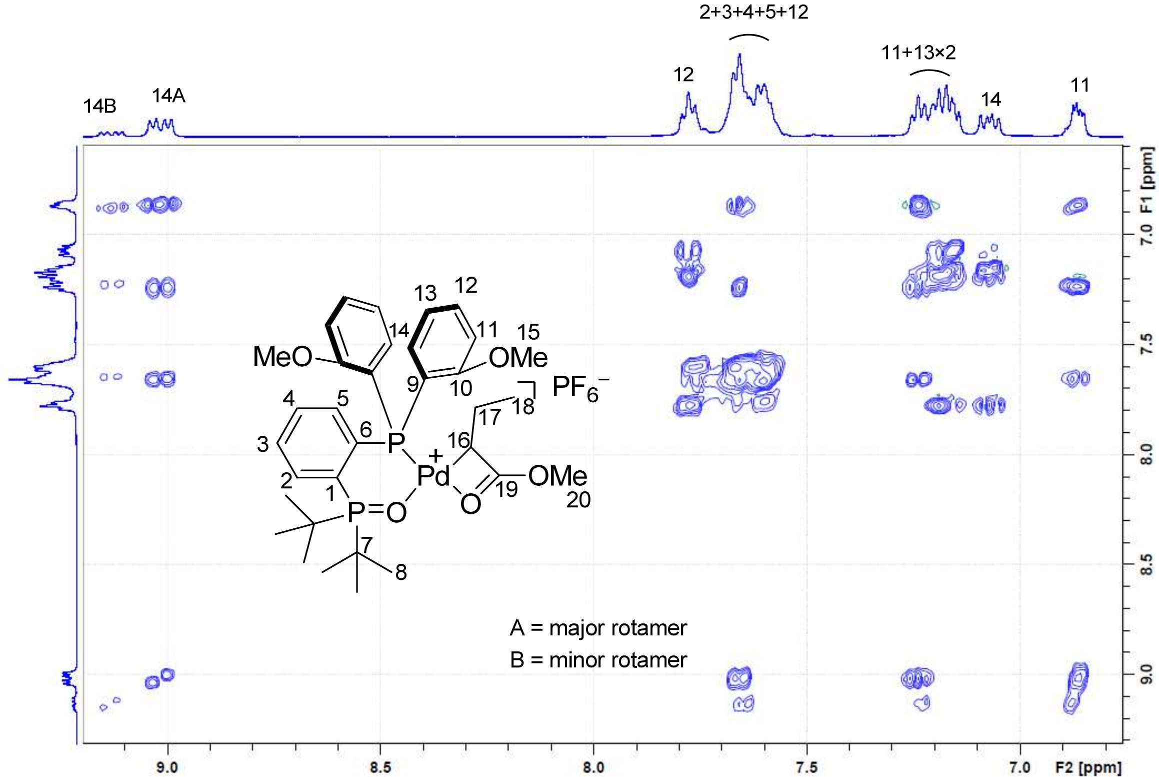

Figure S73. Low field region of ^1H–^1H TOCSY ($C_2D_2Cl_4$, 500 MHz, 5 °C) spectrum of **5c**.

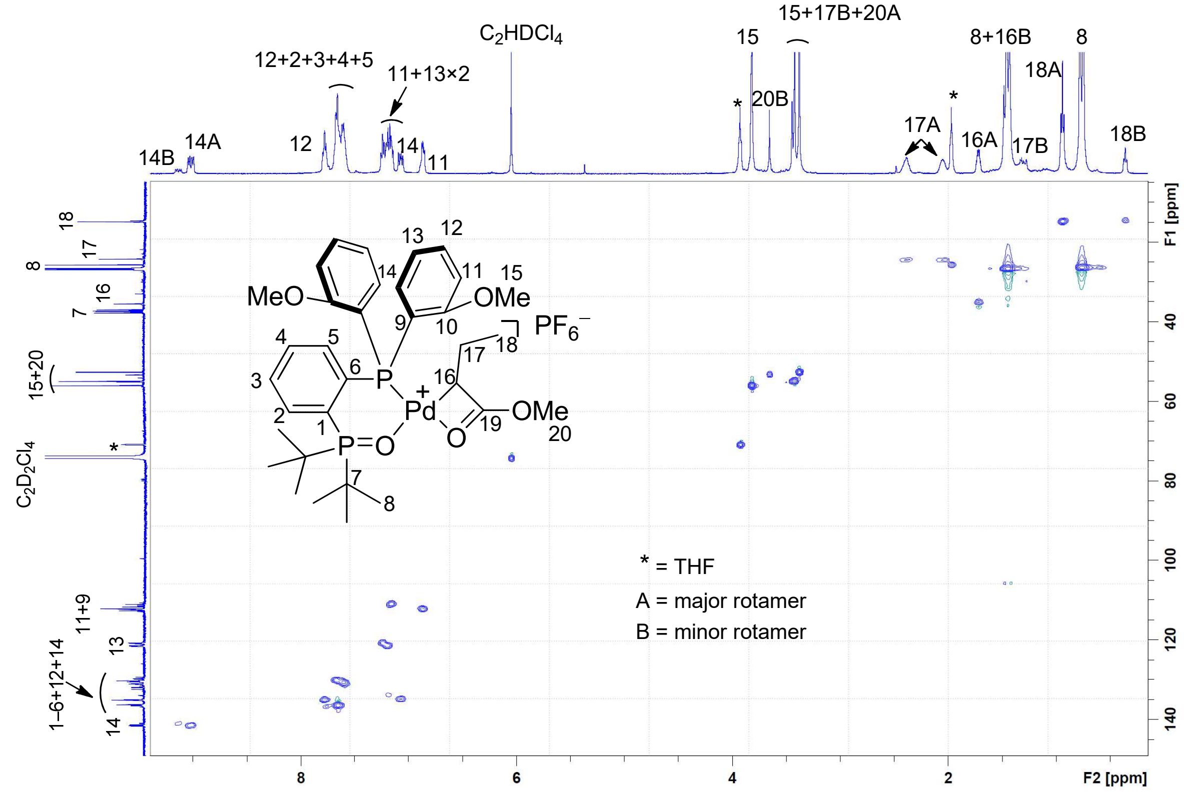

Figure S74. ^{1}H–^{13}C HSQC spectrum ($C_2D_2Cl_4$, 500 MHz for ^{1}H, 126 MHz for ^{13}C, 5 °C) of **5c.**

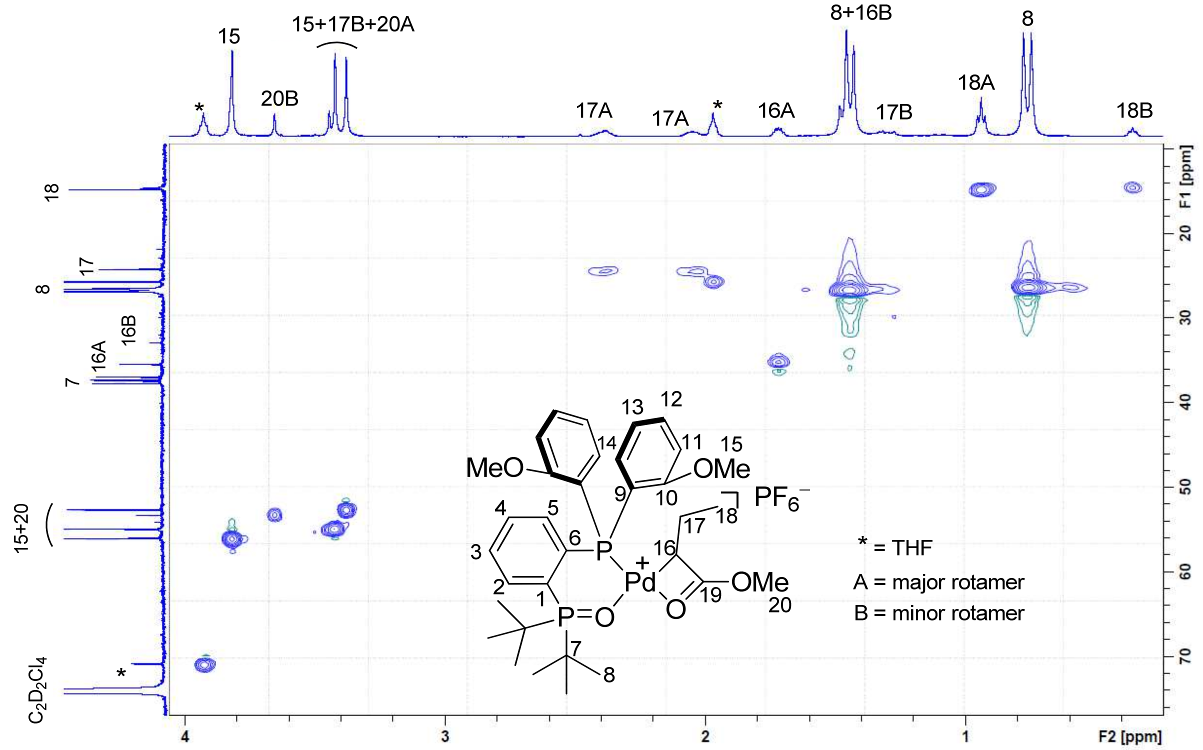

Figure S75. High field region of ^{1}H–^{13}C HSQC spectrum ($C_2D_2Cl_4$, 500 MHz for ^{1}H, 126 MHz for ^{13}C, 5 °C) of **5c**.

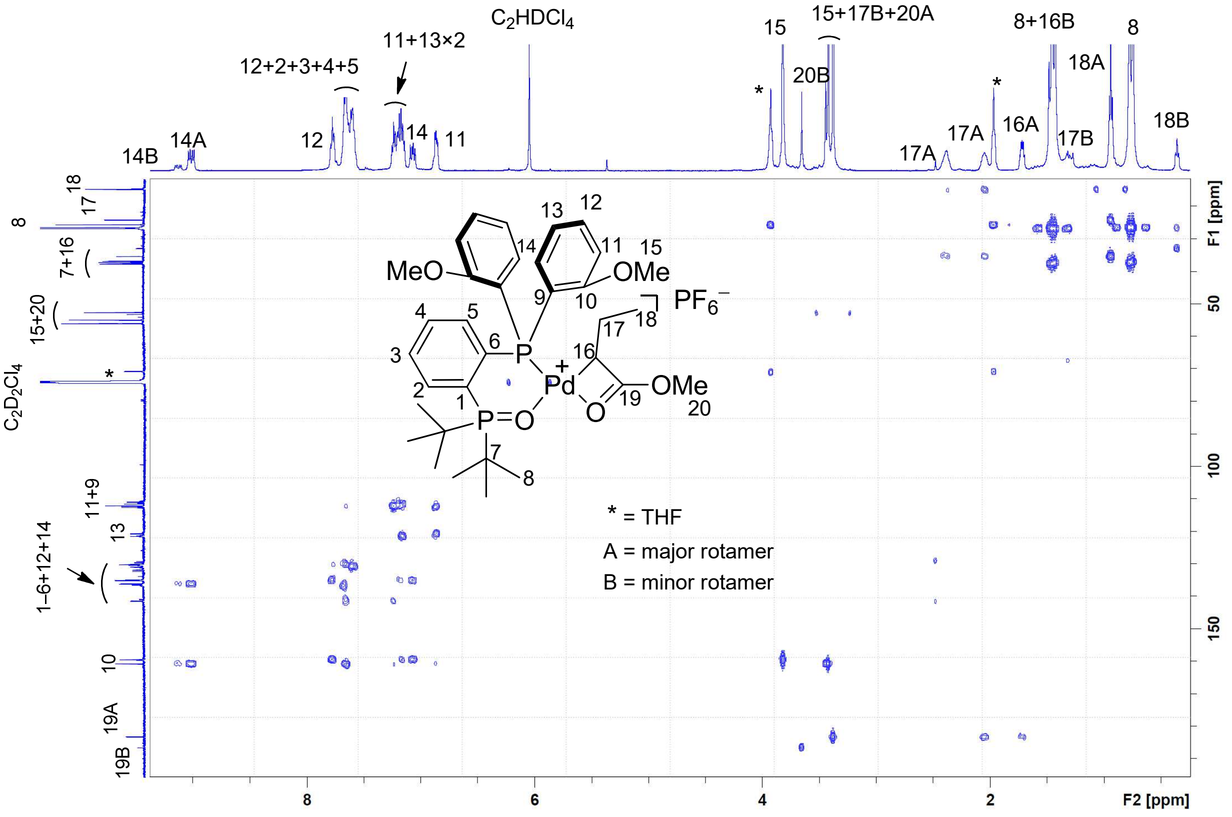

Figure S76. ^{1}H–^{13}C HMBC spectrum ($C_2D_2Cl_4$, 500 MHz for ^{1}H, 126 MHz for ^{13}C, 5 °C) of **5c**.

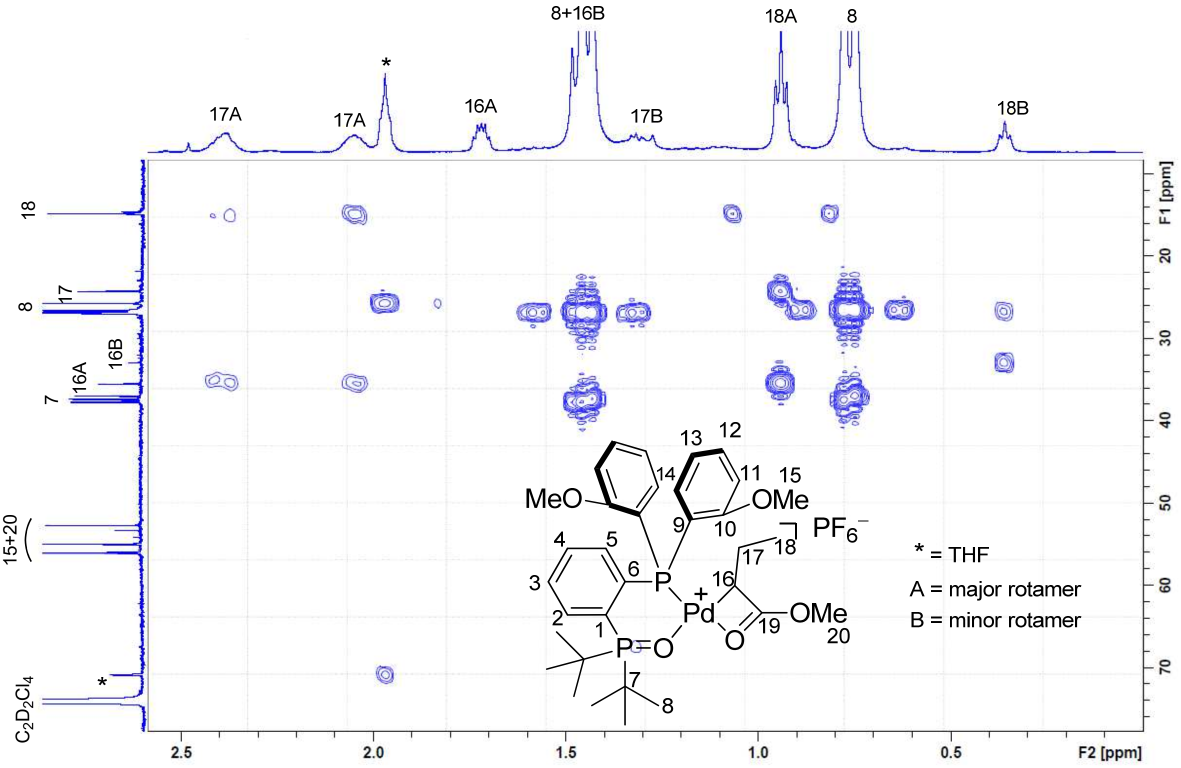

Figure S77. High field region of ^{1}H–^{13}C HMBC spectrum ($C_2D_2Cl_4$, 500 MHz for ^{1}H, 126 MHz for ^{13}C, 5 °C) of **5c**.

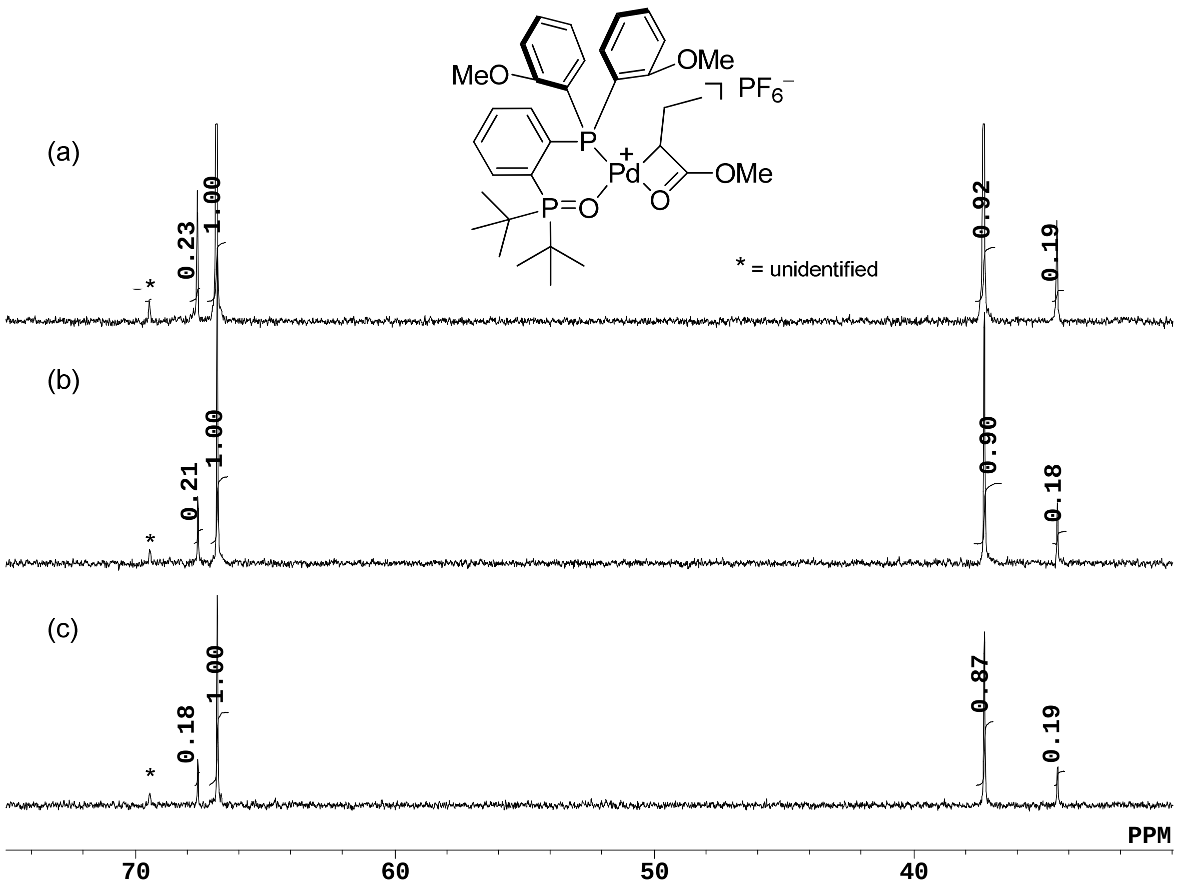

Figure S78. $^{31}P\{^{1}H\}$ NMR spectra ($C_2D_2Cl_4$, 202 MHz, 25 °C) of **5c**. (a) 2.6 × 10^{-2} *M*. (b) 1.3 × 10^{-2} *M*, (c) 8.5 × 10^{-3} *M*.

4-5 NMR Spectra of 5c-py

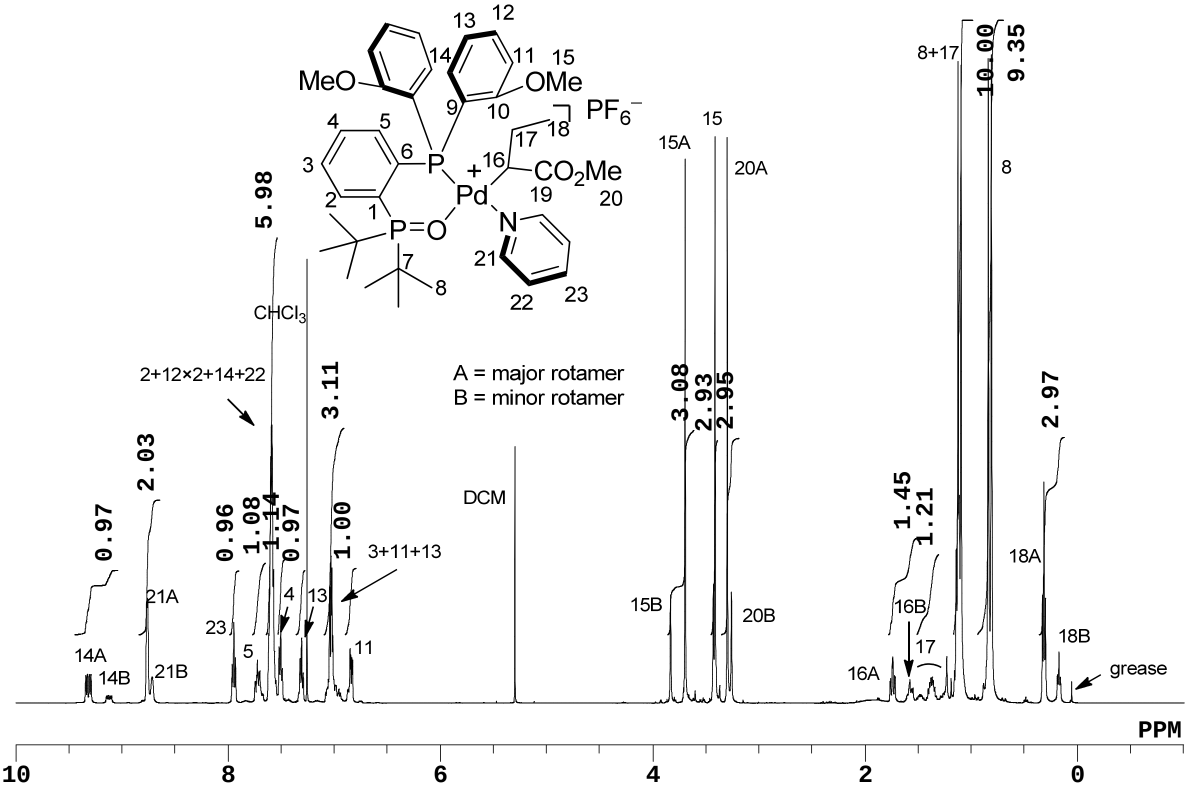

Figure S79. ^{1}H NMR spectrum ($CDCl_3$, 500 MHz, 5 °C) of **5c-py**.

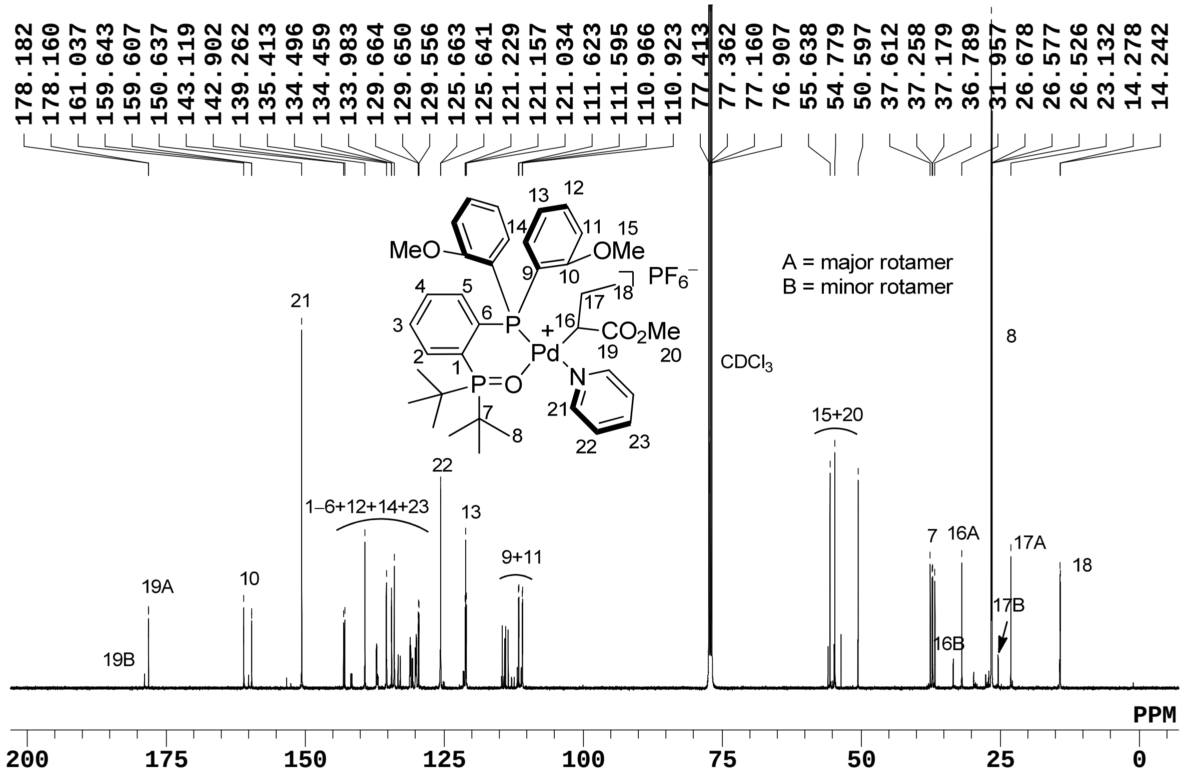

Figure S80. $^{13}C\{^{1}H\}$ NMR spectrum ($CDCl_3$, 126 MHz, 5 °C) of **5c-py**.

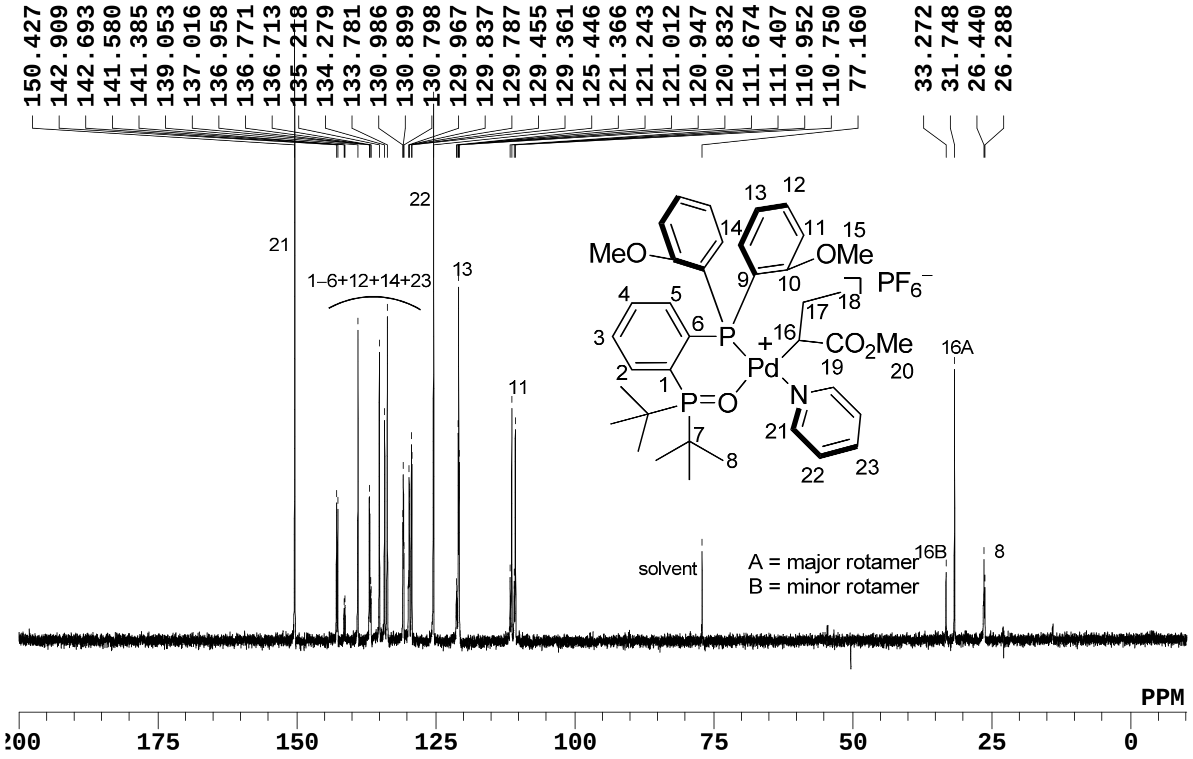

Figure S81. ^{13}C DEPT90 NMR spectrum (CDCl3, 126 MHz, 5 °C) of **5c-py**.

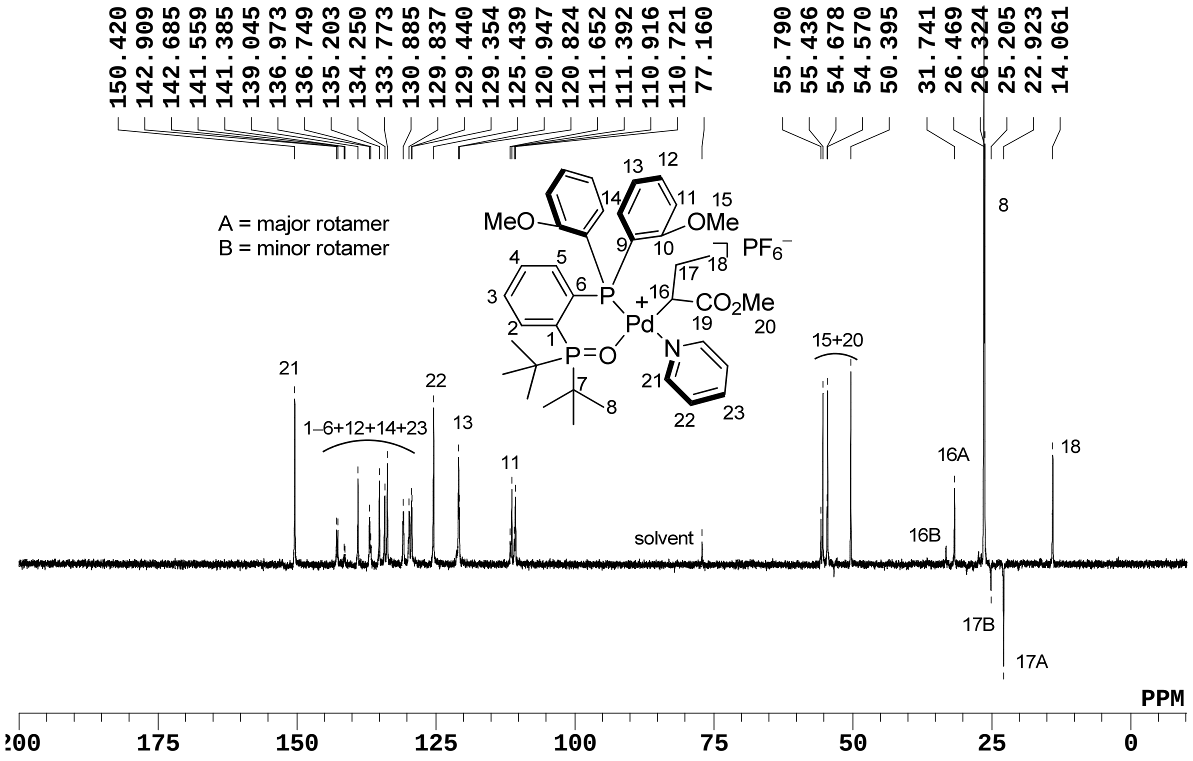

Figure S82. ^{13}C DEPT135 NMR spectrum (CDCl3, 126 MHz, 5 °C) of **5c-py**.

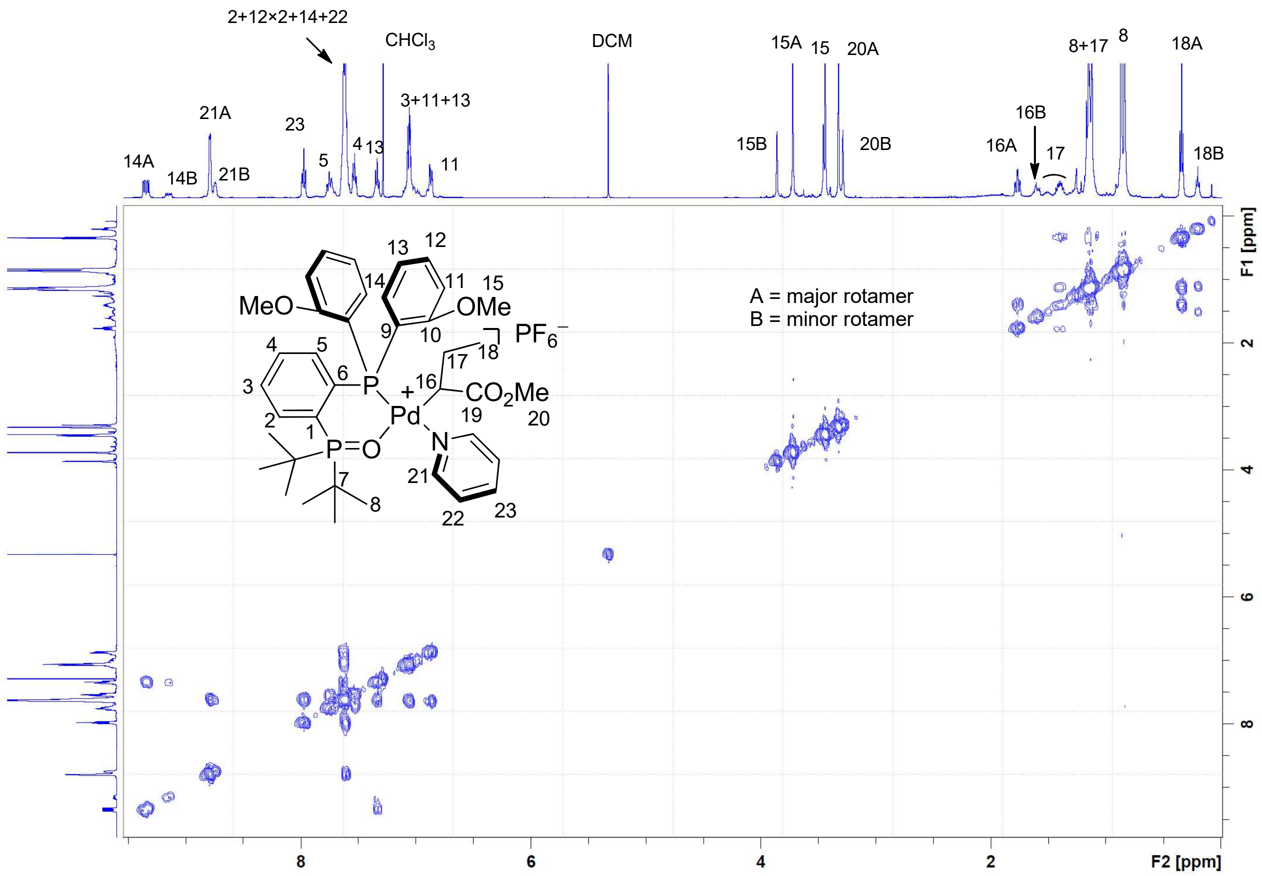

Figure S83. ^{1}H–^{1}H COSY spectrum ($CDCl_3$, 500 MHz, 5 °C) of **5c-py**.

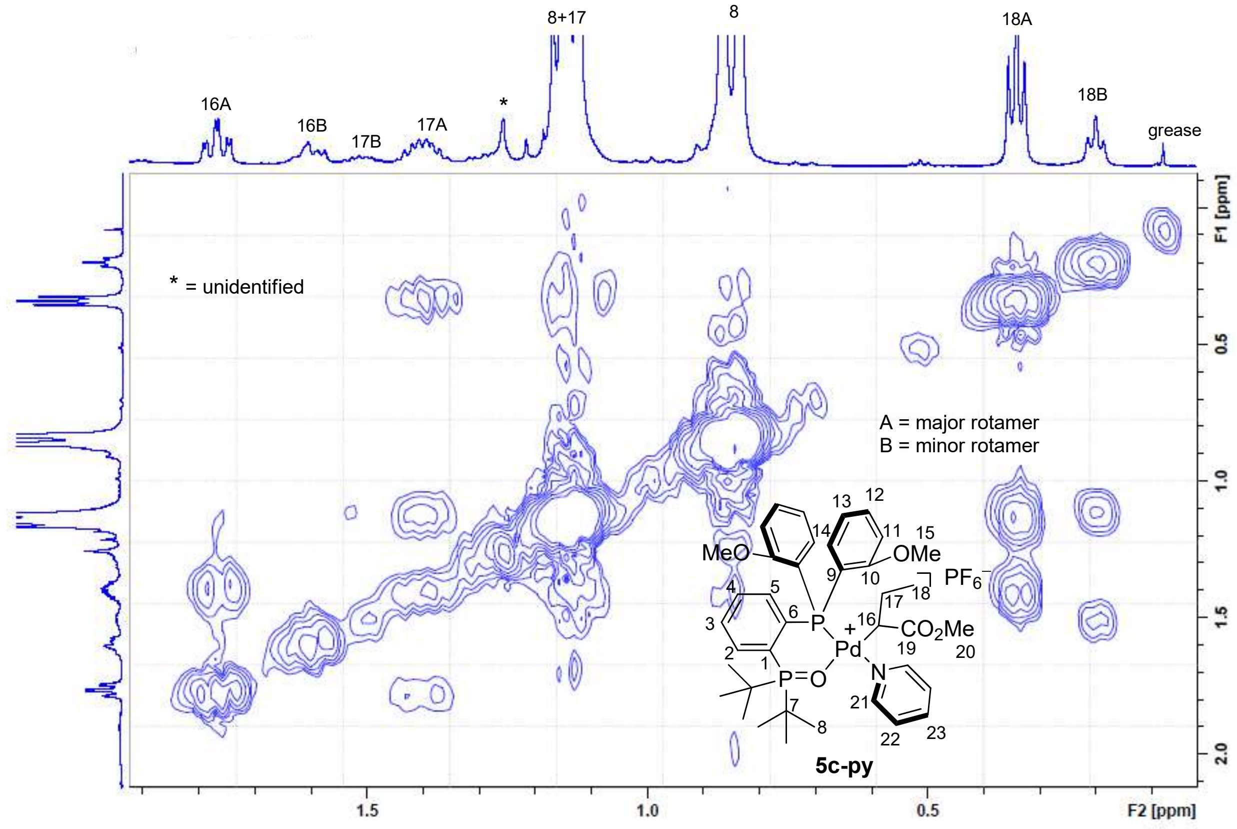

Figure S84. High field region of ^{1}H–^{1}H COSY spectrum ($CDCl_3$, 500 MHz, 5 °C) of **5c-py**.

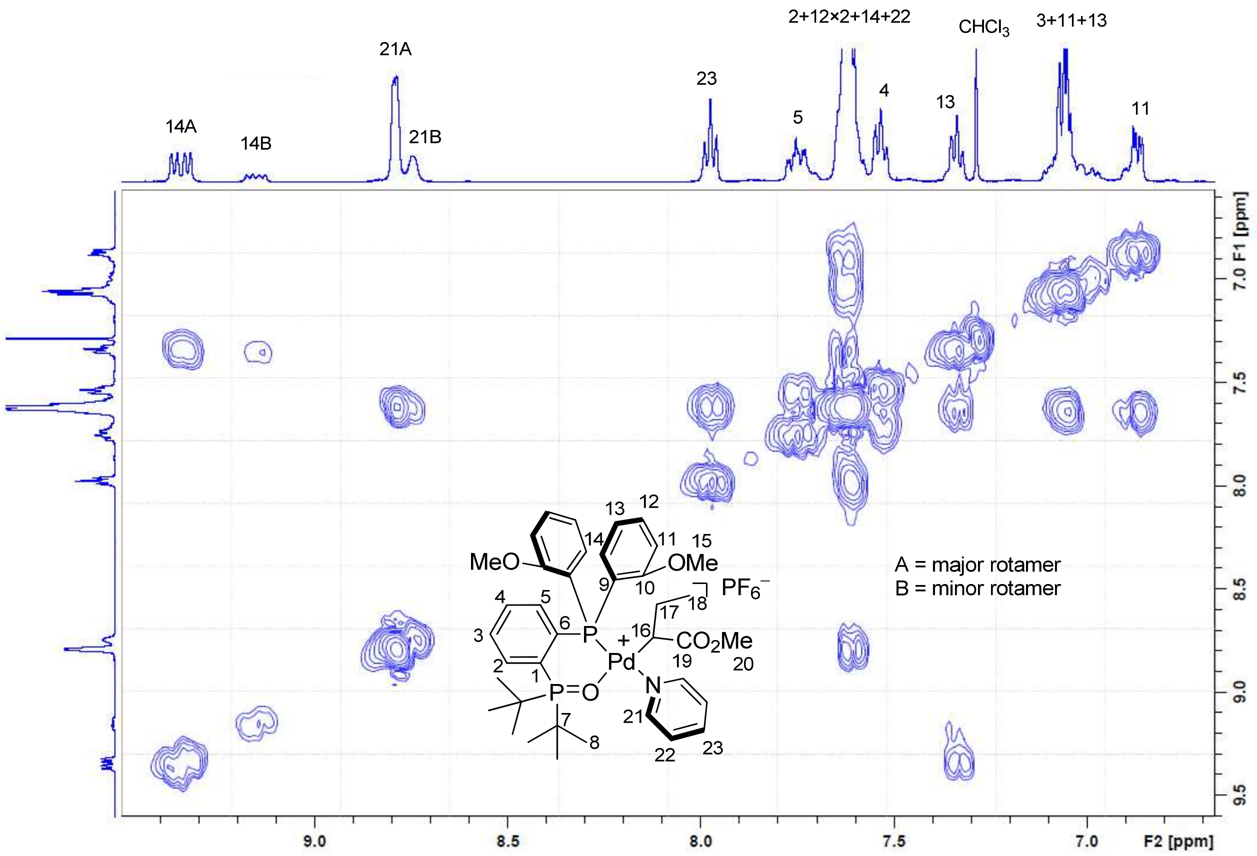

Figure S85. Low field region of ^{1}H–^{1}H COSY spectrum ($CDCl_3$, 500 MHz, 5 °C) of **5c-py**.

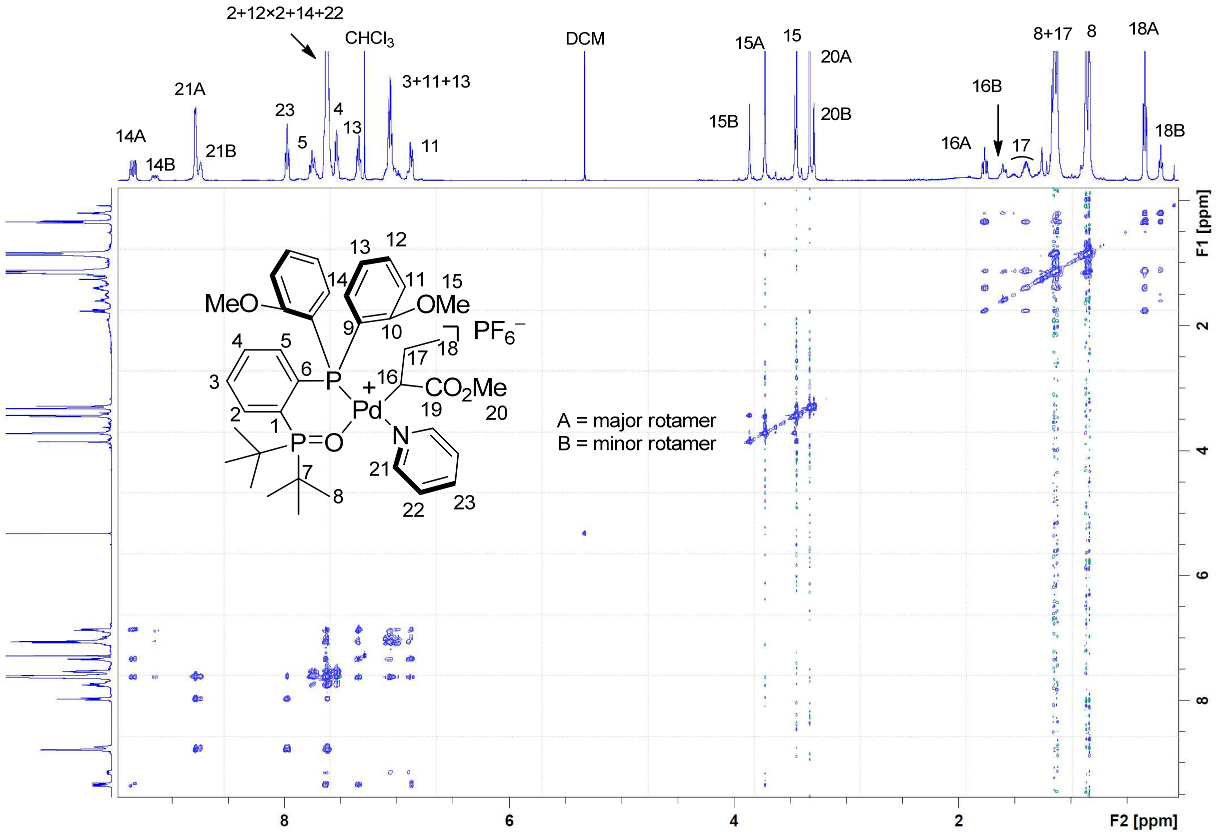

Figure S86. ^{1}H–^{1}H TOCSY spectrum ($CDCl_3$, 500 MHz, 5 °C) of **5c-py**.

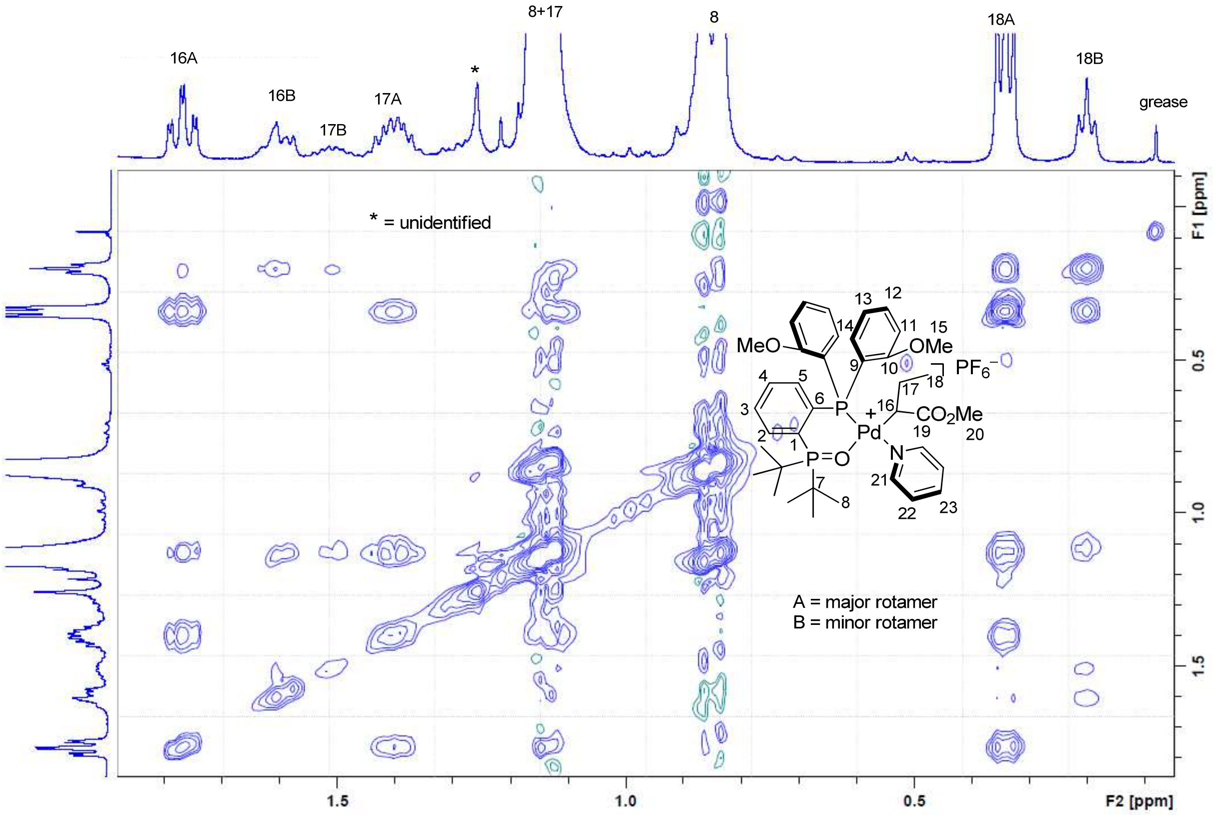

Figure S87. High field region of ^1H–^1H TOCSY spectrum ($CDCl_3$, 500 MHz, 5 °C) of **5c-py**.

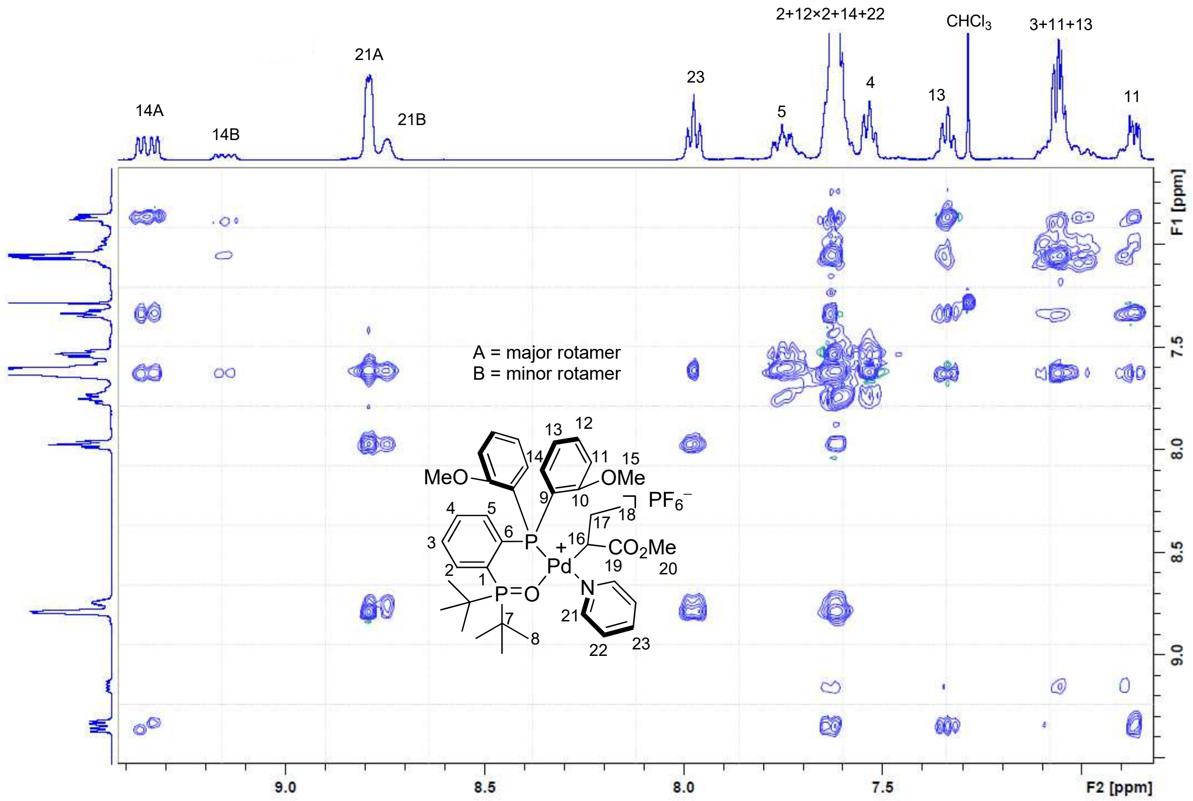

Figure S88. Low field region of ^1H–^1H TOCSY spectrum ($CDCl_3$, 500 MHz, 5 °C) of **5c-py**.

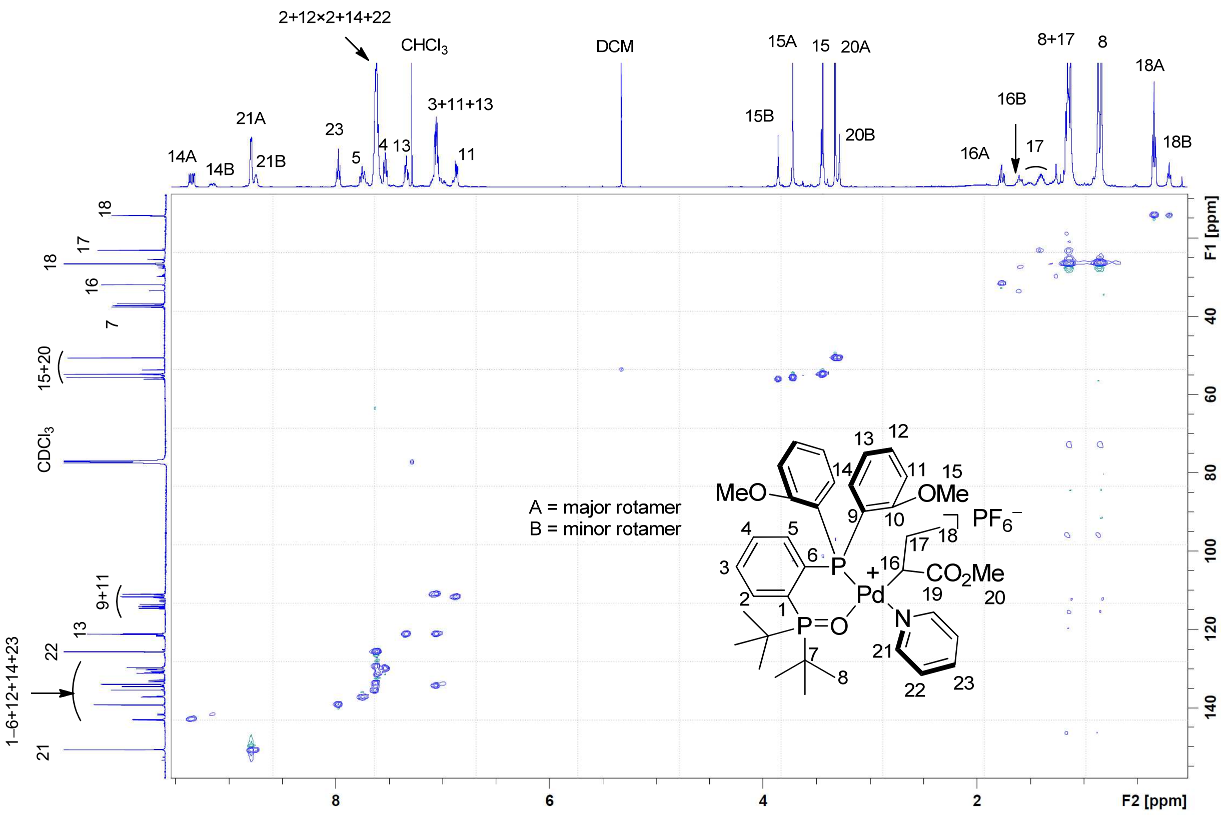

Figure S89. ^{1}H–^{13}C HSQC spectrum ($CDCl_3$, 500 MHz for ^{1}H, 126 MHz for ^{13}C, 5 °C) of **5c-py**.

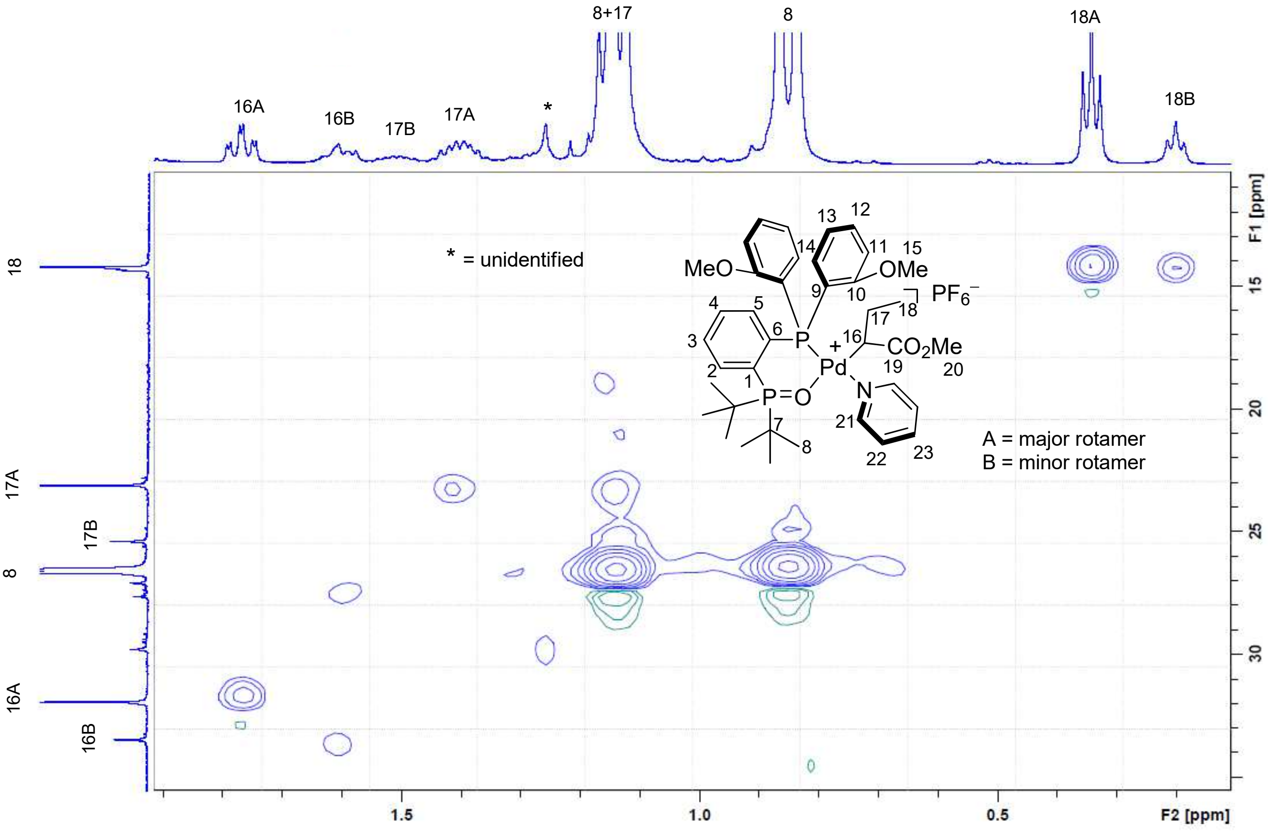

Figure S90. High field region of ^{1}H–^{13}C HSQC spectrum ($CDCl_3$, 500 MHz for ^{1}H, 126 MHz for ^{13}C, 5 °C) of **5c-py**.

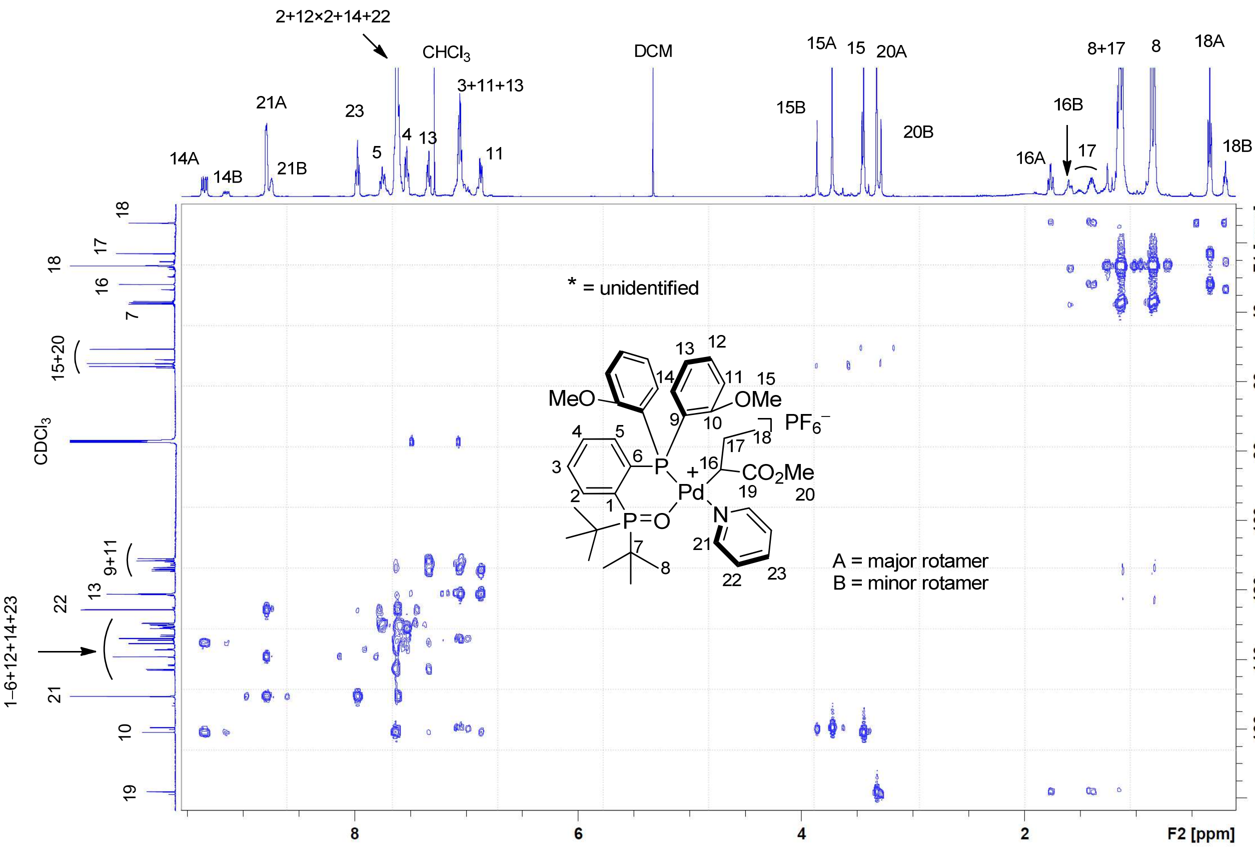

Figure S91. ^{1}H–^{13}C HMBC spectrum ($CDCl_3$, 500 MHz for ^{1}H, 126 MHz for ^{13}C, 5 °C) of **5c-py**.

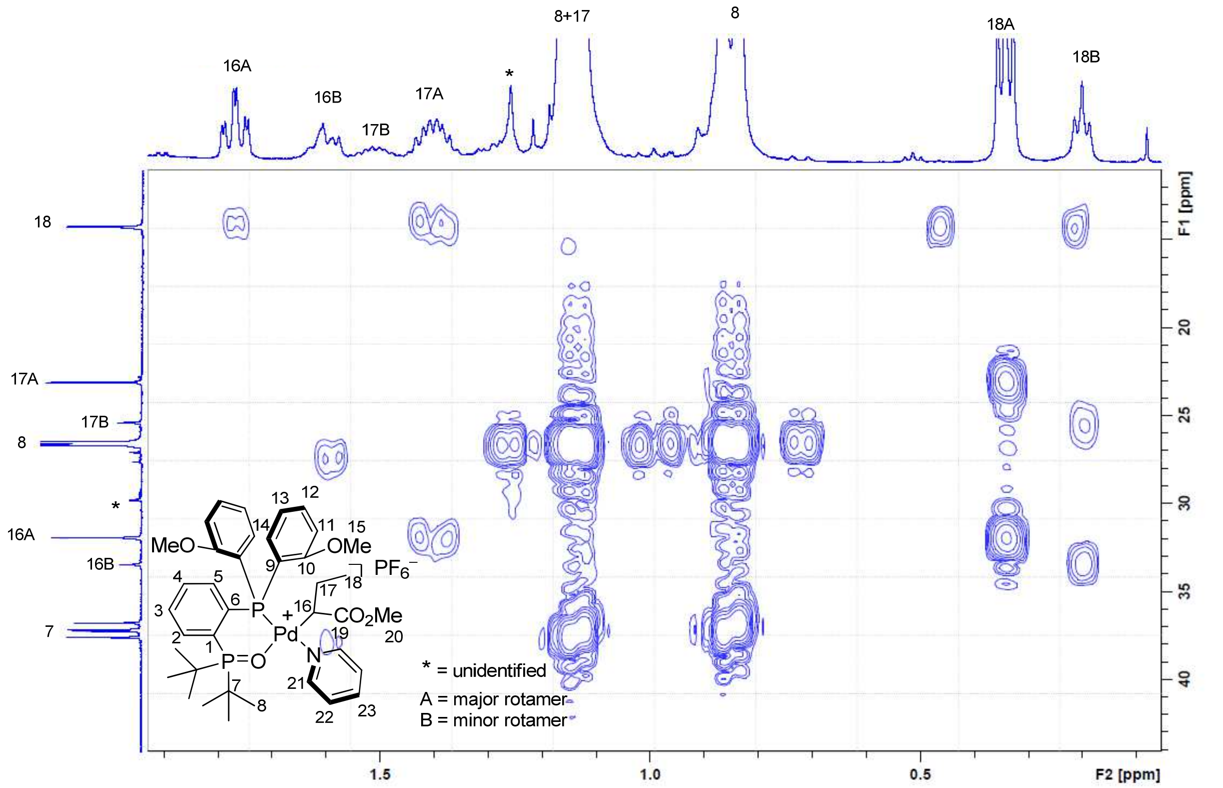

Figure S92. High field region of ^{1}H–^{13}C HMBC spectrum ($CDCl_3$, 500 MHz for ^{1}H, 126 MHz for ^{13}C, 5 °C) of **5c-py**.

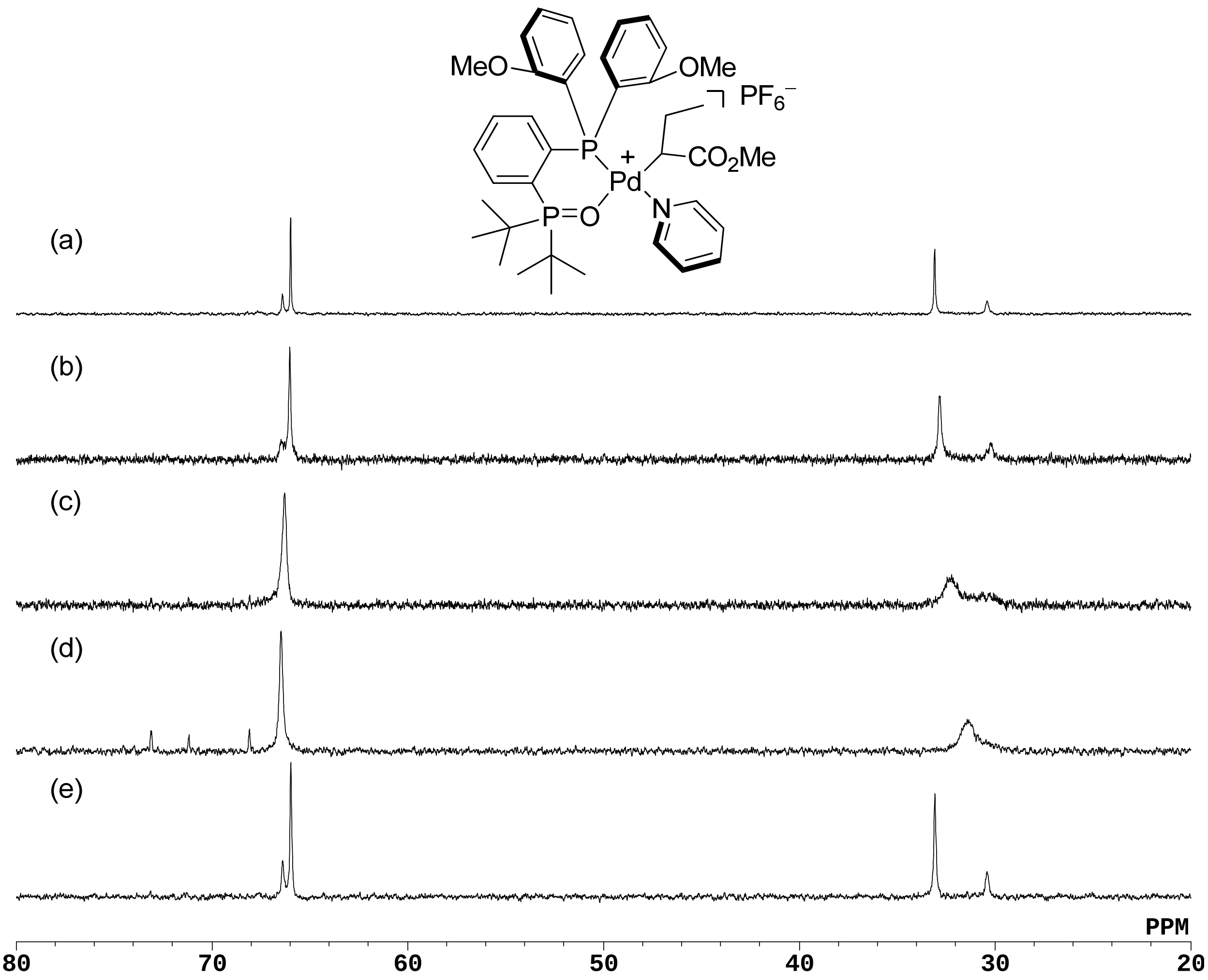

Figure S93. $^{31}P\{^{1}H\}$ NMR spectra ($C_2D_2Cl_4$, 202 MHz) of **5c-py** at (a) 20 °C, (b) 50 °C, (c) 80 °C, (d) 100 °C, (e) cooled to 20 °C after heating to 100 °C.

4-6 NMR Spectra of 5d-py

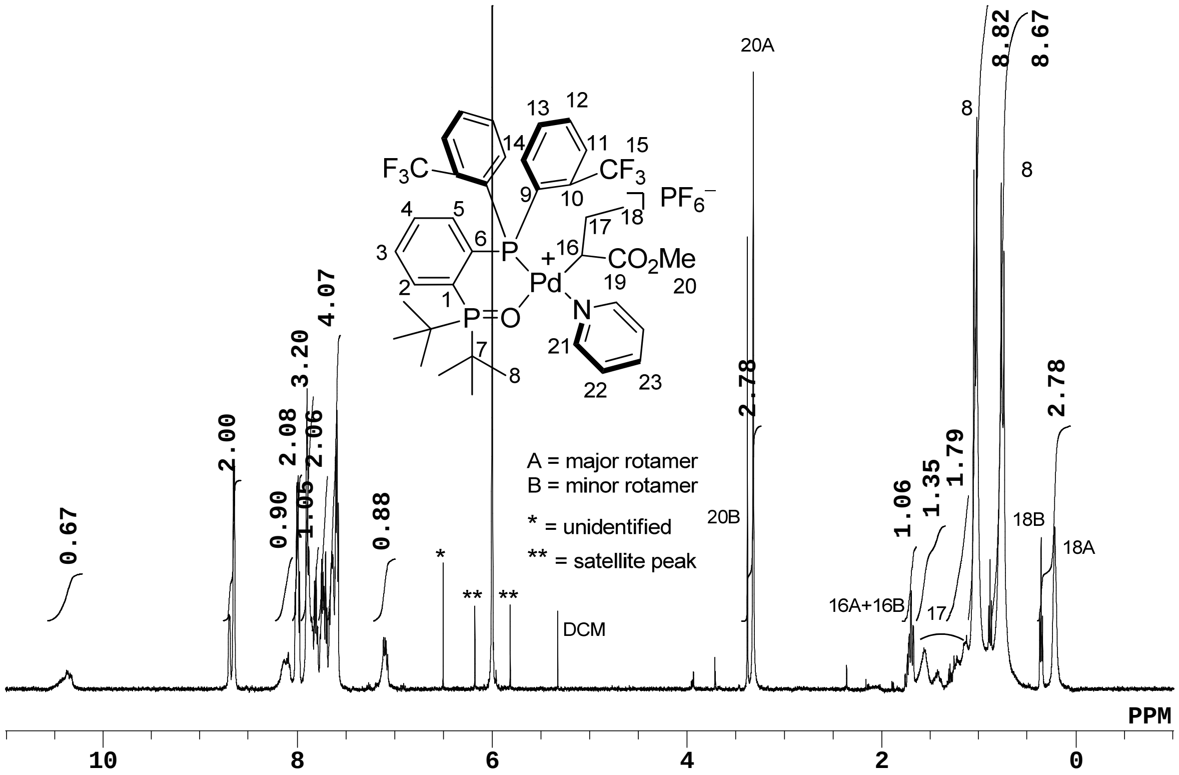

Figure S94. ^{1}H NMR spectrum ($C_2D_2Cl_4$, 500 MHz) of **5d-py**.

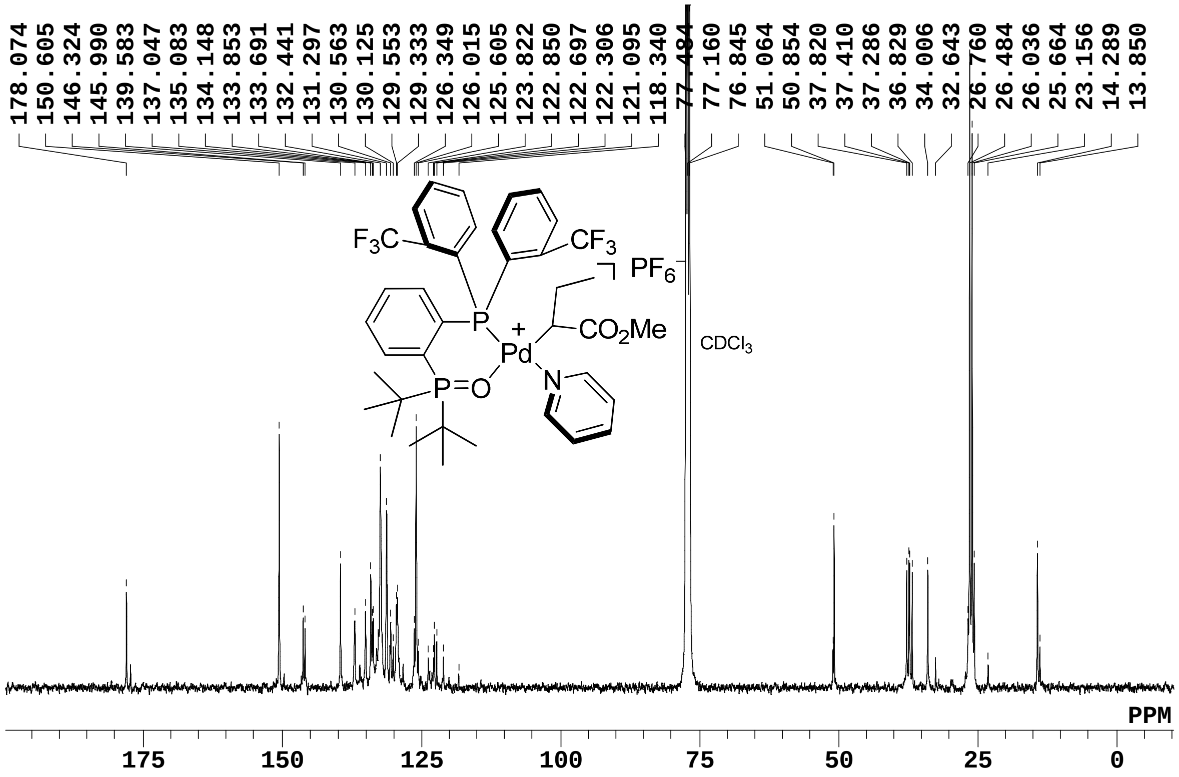

Figure S95. $^{13}C\{^{1}H\}$ NMR spectrum ($CDCl_3$, 101 MHz, –50 °C) of **5d-py**.

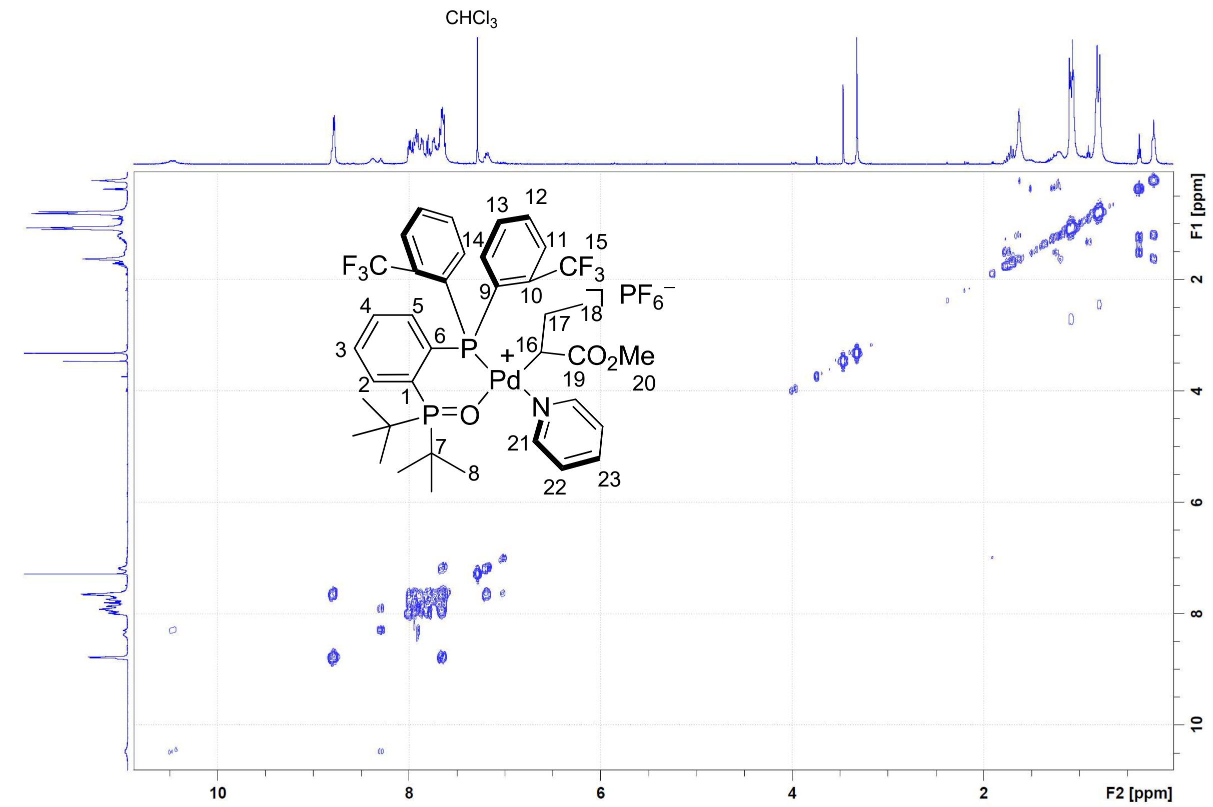

Figure S96. ^1H–^1H COSY spectrum ($CDCl_3$. 500 MHz) of **5d-py**.

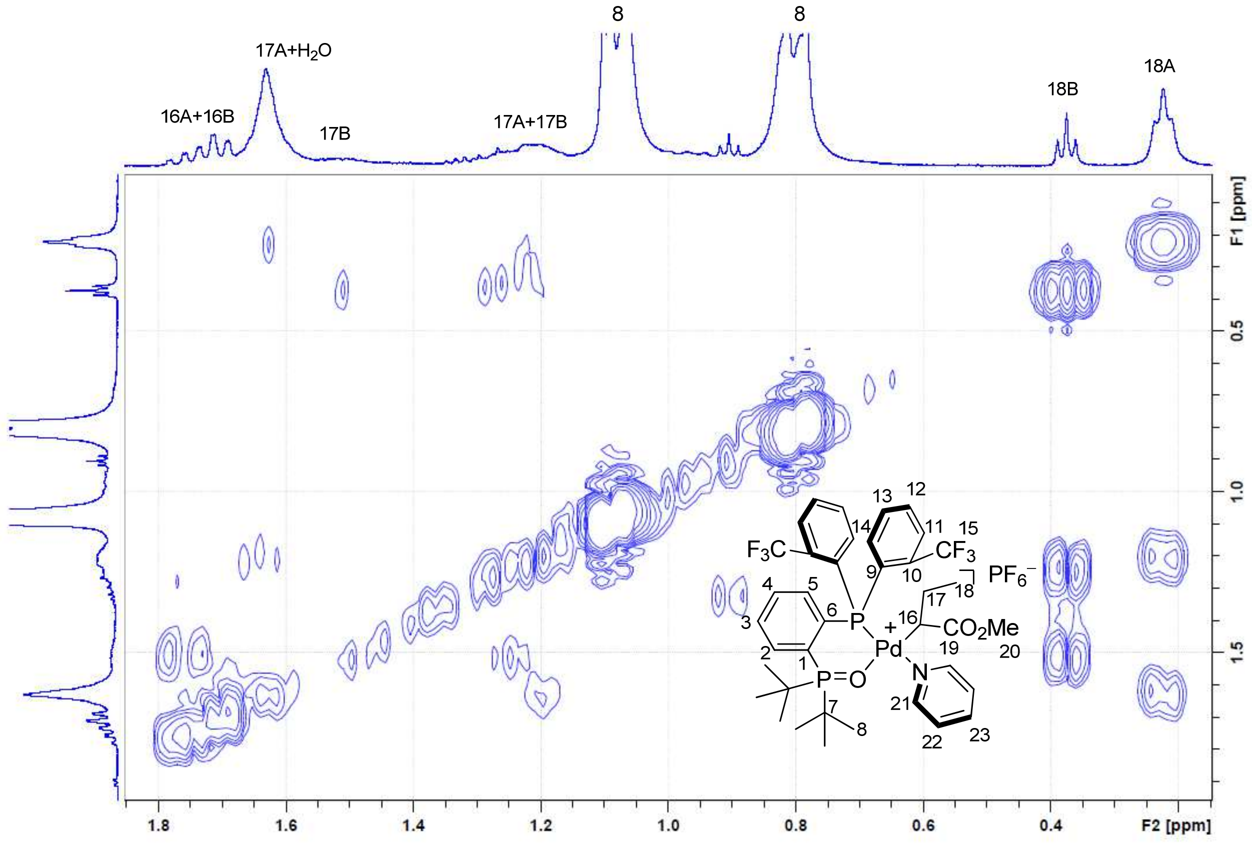

Figure S97. High field region of ^1H–^1H COSY spectrum ($CDCl_3$. 500 MHz) of **5d-py**.

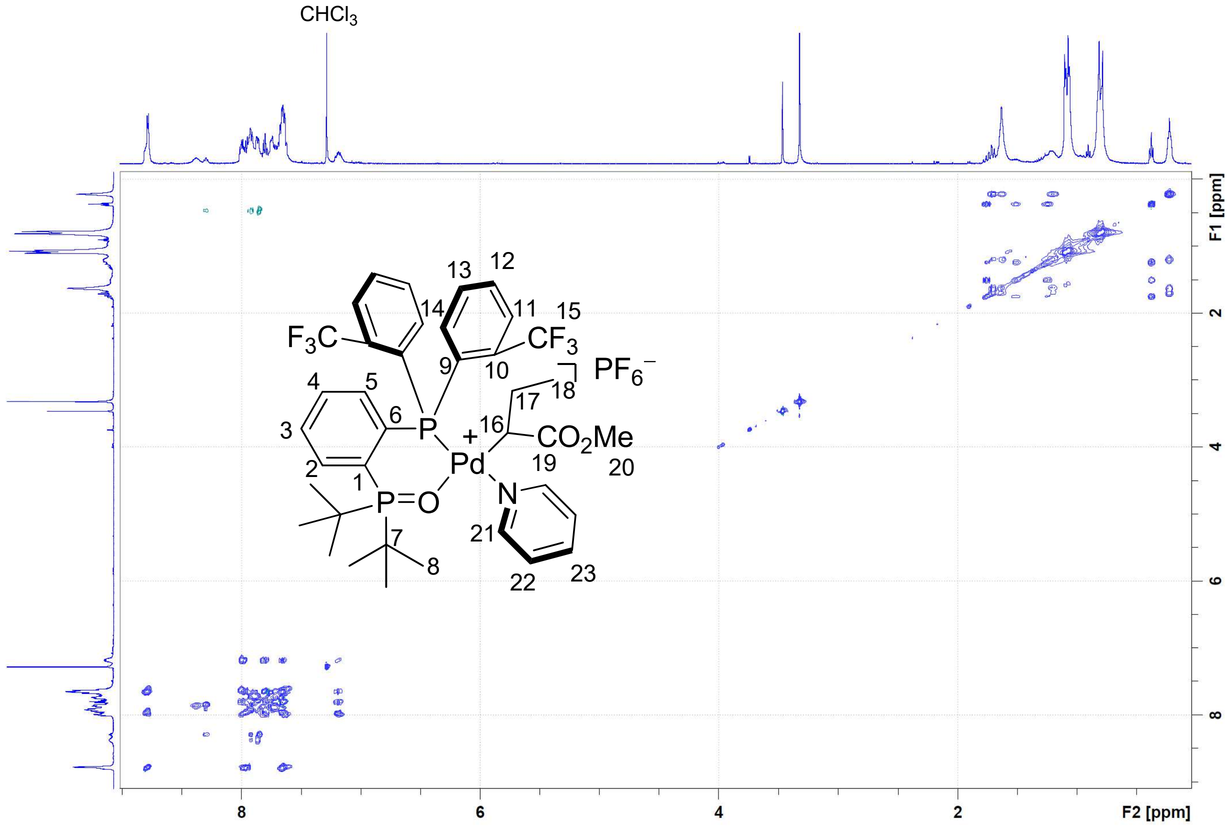

Figure S98. ^{1}H–^{1}H TOCSY spectrum (CDCl3. 500 MHz) of **5d-py**.

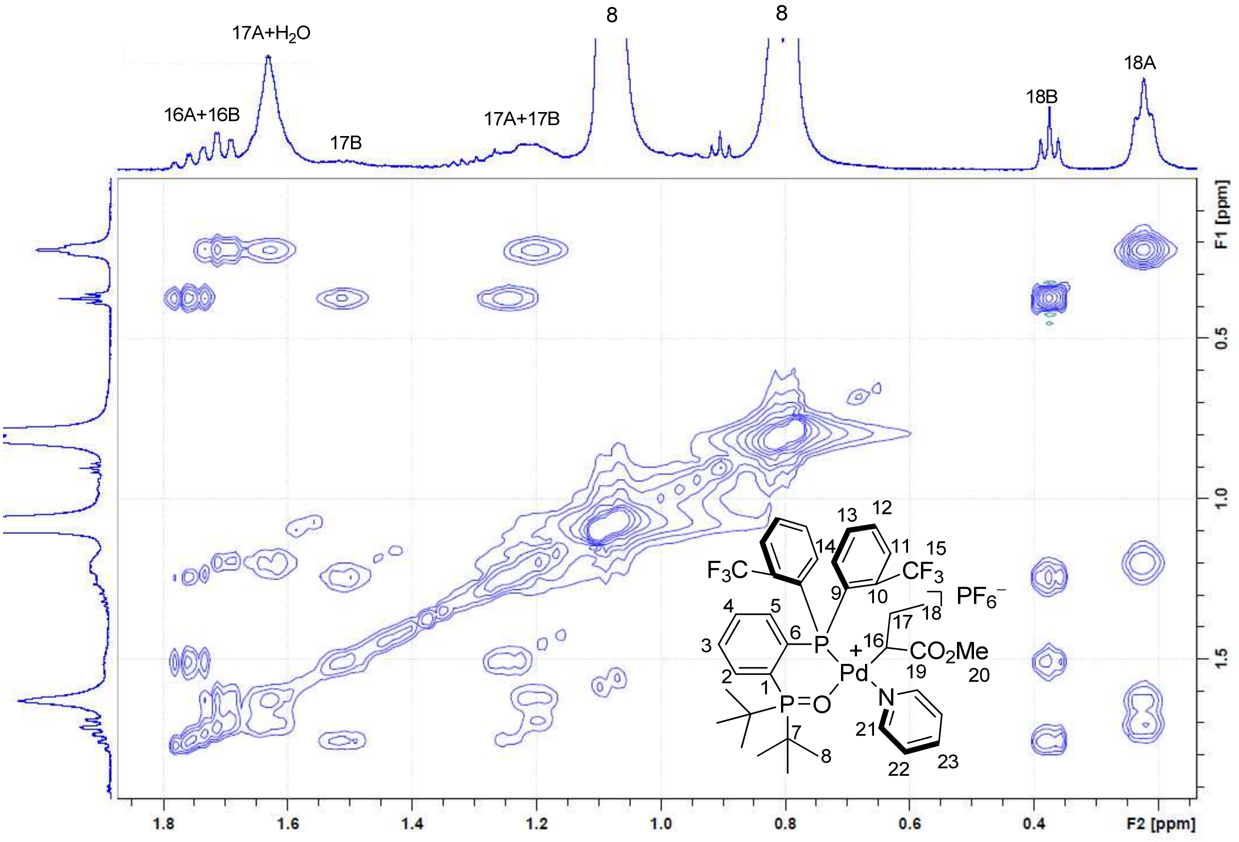

Figure S99. High field region of ^{1}H–^{1}H TOCSY spectrum (CDCl3. 500 MHz) of **5d-py**.

Discussion for 5c, 5c-py and 5d-py

For **5c**, **5c-py**, and **5d-py**, four signals were observed in their $^{31}P\{^{1}H\}$ NMR spectra. We concluded that two rotamers exist for all complexes.

For **5c-py**, four signals were observed by $^{31}P\{^{1}H\}$ NMR at 20 °C (Figure S93a). As the temperature increases, the signals started to be fused and coalesced at 100 °C (Figures S93b–d). After heating to 100 °C, the sample was cooled to 20 °C and analysed by NMR at 20 °C (Figure S93e). Initial four signals were observed again, which indicated that transformation of **5c-py** to another species did not occur during the variable-temperature measurements. Thus, the four signals observable at 20 °C originates from the sole product having two rotamers. Also, all the NMR spectra are consistent with the structure of **5c-py**.

For **5c**, we examined variable-temperature NMR experiments for **5c-py**, but **5c** decomposed before coalescence of signals. Next, $^{31}P\{^{1}H\}$ NMR spectra were taken at various concentrations (Figure S78). Unlike to **5a-mono/5a-di**, the integral ratio of the signals was almost constant at any concentration. This observation excludes the possibility of the presence of equilibrium between a monomer and a dimer. It is possible that two different products exist in the system, but all the NMR spectra are consistent with the structure of **5c**. Considering that **5c-py** also has two rotamers, it is probable that **5c** has also two rotamers, although it is unclear whether **5c** exists as a monomer or a dimer in the solution.

5d-py decomposed at high temperature before coalescence of signals. Thermolysis of **5d-py** afforded methyl crotonate in 82% yield (Figure S36), indicating the observed species in the NMR spectra of **5d-py** were not a mixture of two products. Also, ^{1}H–^{1}H COSY and TOCSY spectra support the structure of **5d-py** and presence of two isomers. Considering the afore-mentioned observations, we concluded that **5d-py** has two isomers.

5. NMR Spectra of Polymers

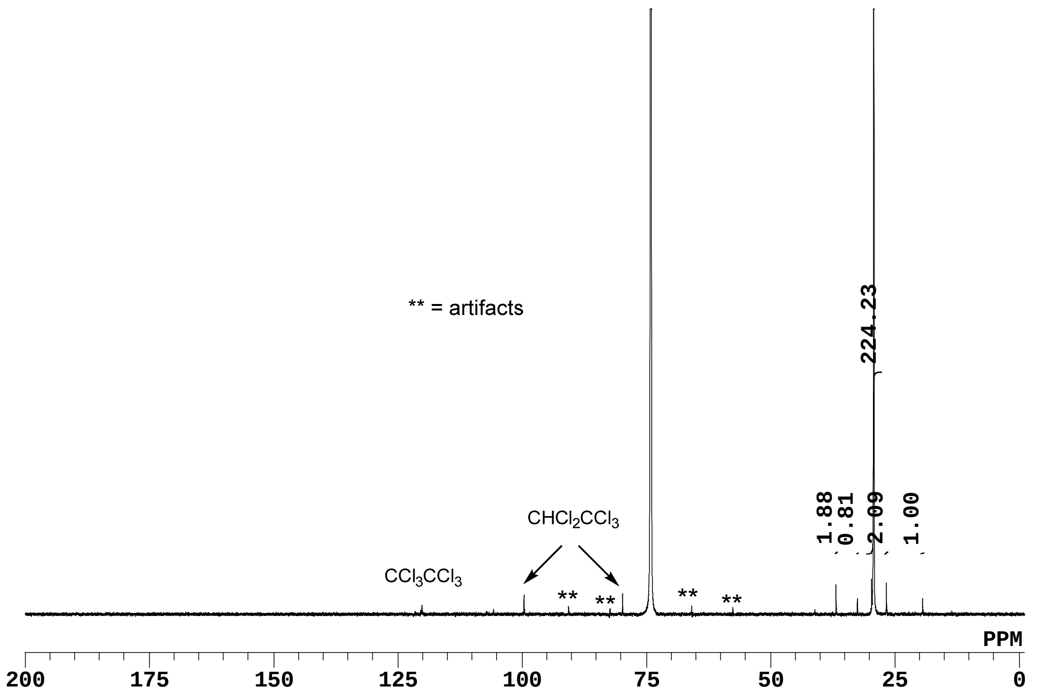

Figure S100. Quantitative ^{13}C NMR spectrum (1,1,2,2-tetrachloroethane, 126 MHz, 120 °C) of the polyethylene in Table 1, entry 1.

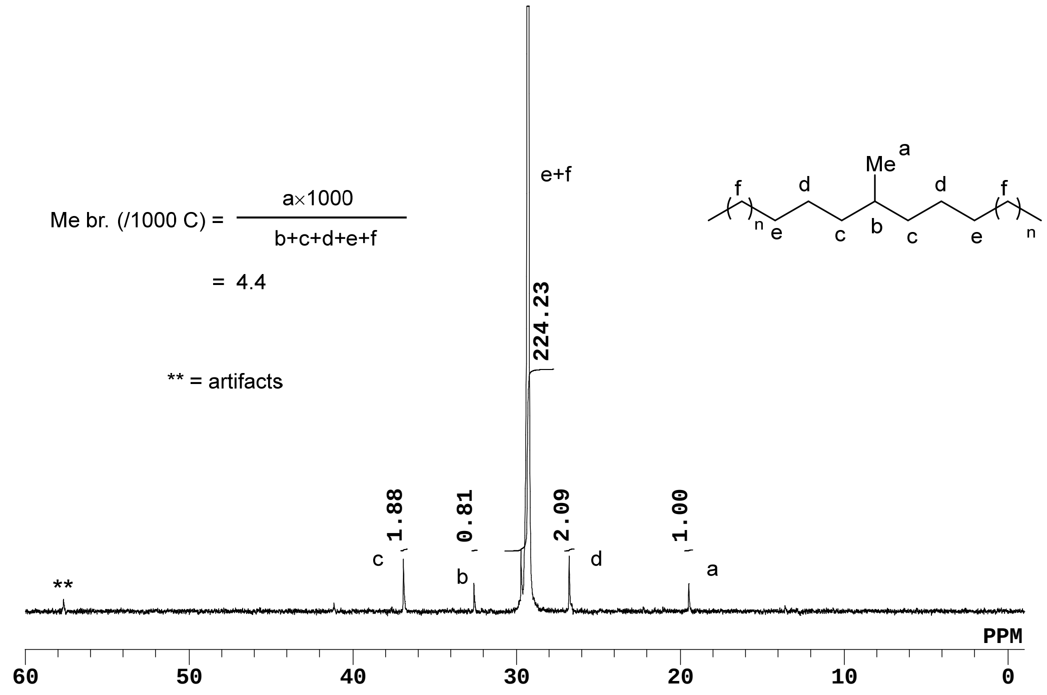

Figure S101. High field region of quantitative ^{13}C NMR spectrum (1,1,2,2-tetrachloroethane, 126 MHz, 120 °C) of the polyethylene in Table 1, entry 1.

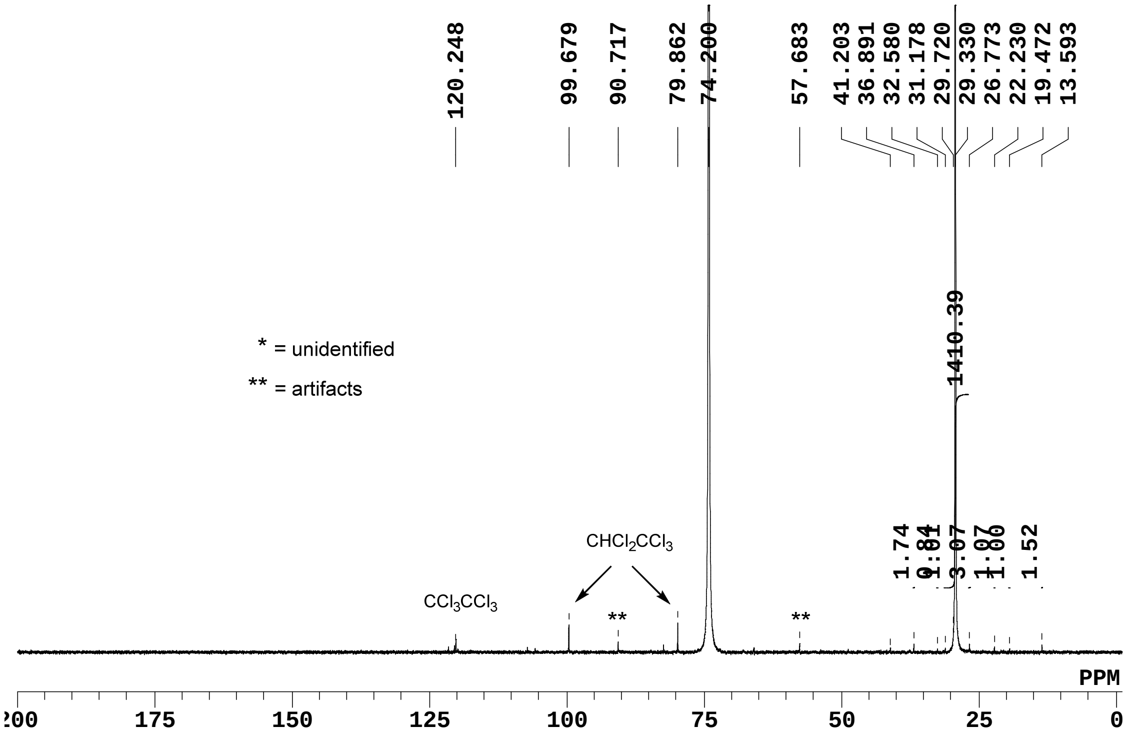

Figure S102. Quantitative ^{13}C NMR spectrum (1,1,2,2-tetrachloroethane, 126 MHz, 120 °C) of the polyethylene obtained in Table 1, entry 2.

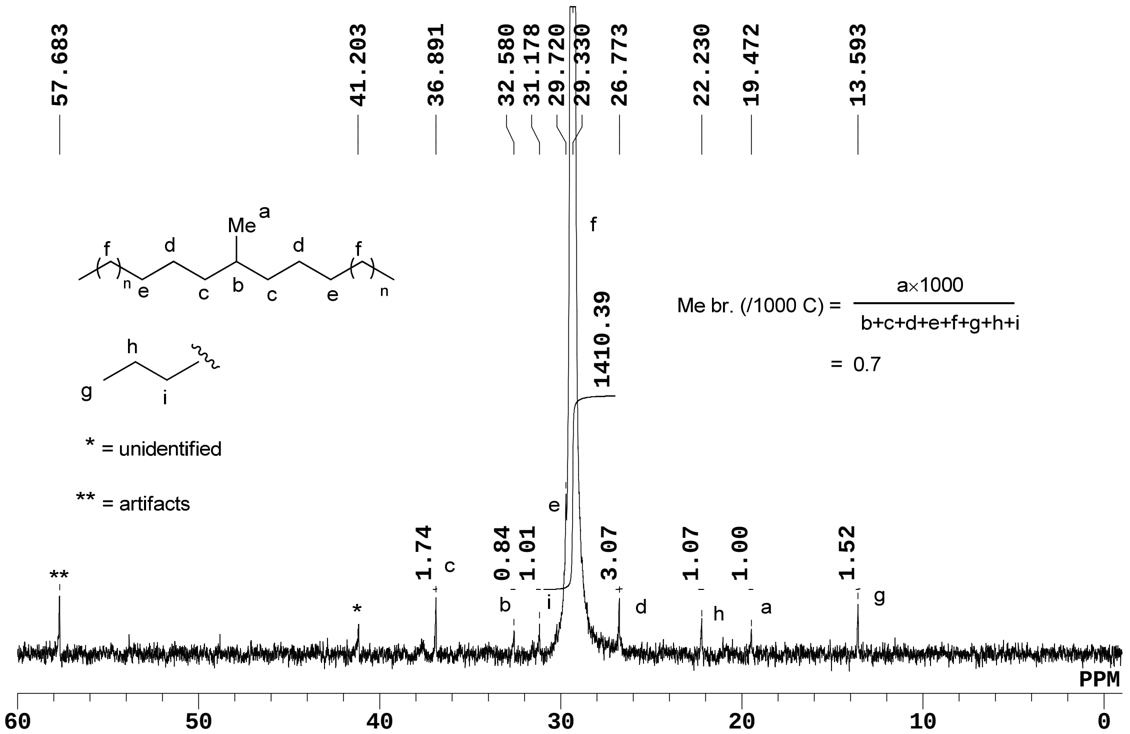

Figure S103. High field region of quantitative ^{13}C NMR spectrum (1,1,2,2-tetrachloroethane, 126 MHz, 120 °C) of the polyethylene obtained in Table 1, entry 2.

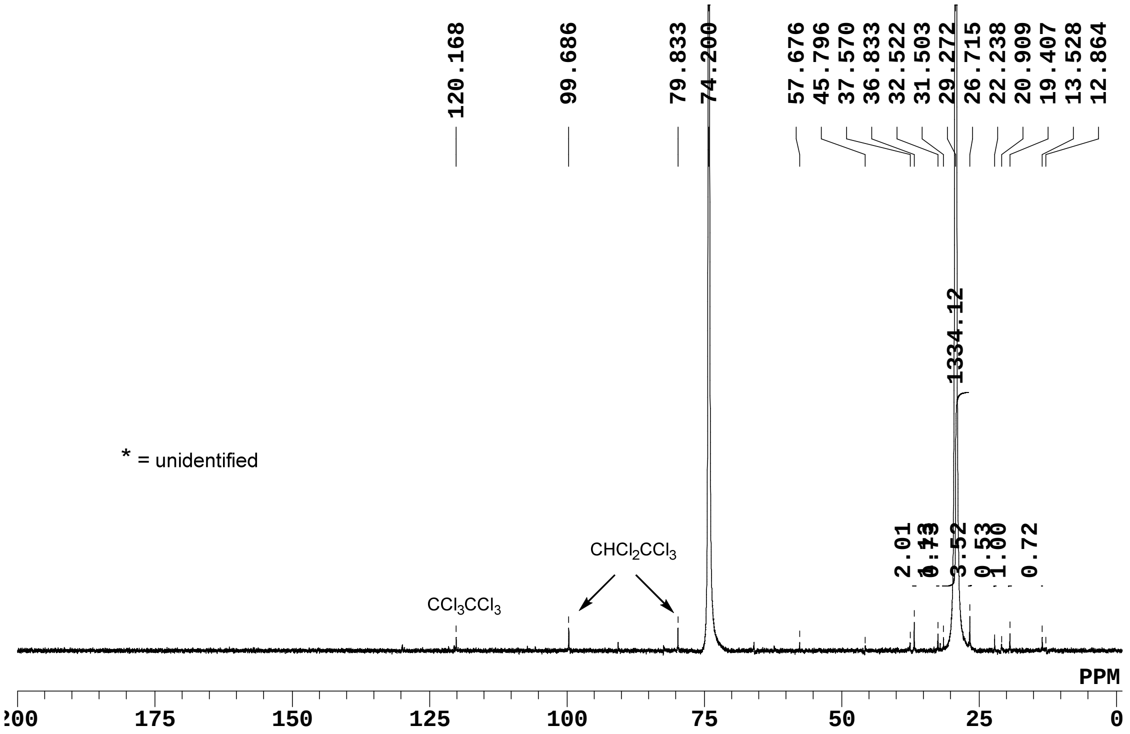

Figure S104. Quantitative ^{13}C NMR spectrum (1,1,2,2-tetrachloroethane, 126 MHz, 120 °C) of the polyethylene obtained in Table 1, entry 3.

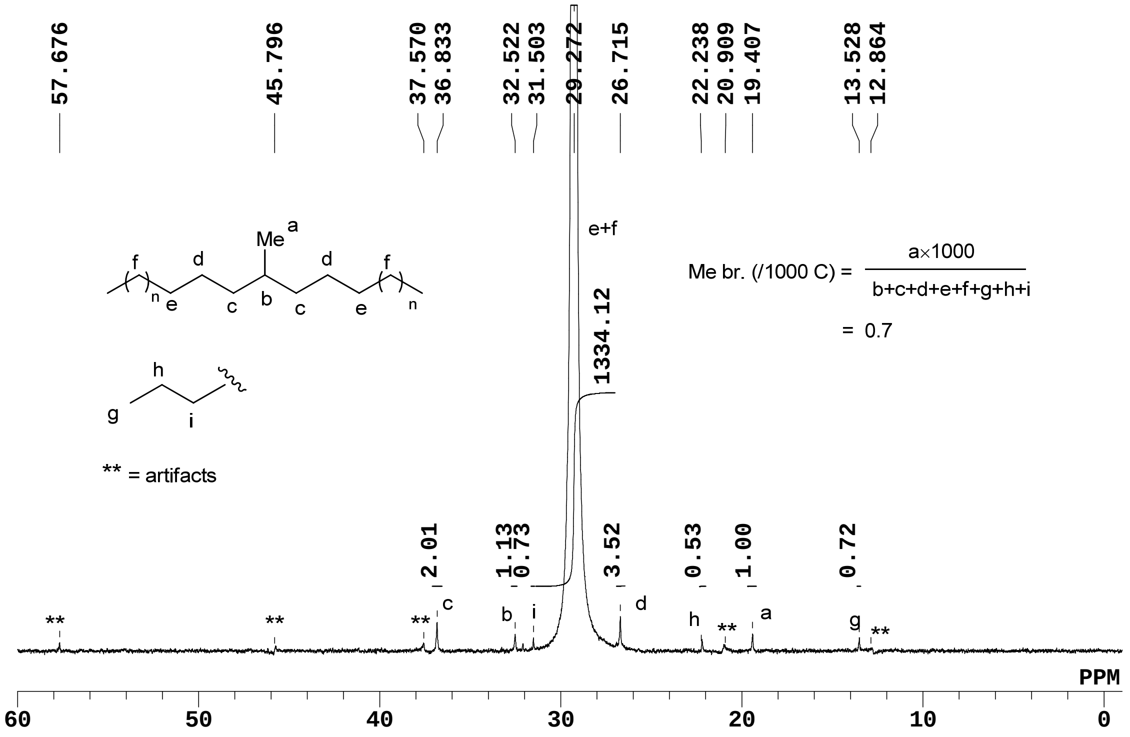

Figure S105. High field region of quantitative ^{13}C NMR spectrum (1,1,2,2-tetrachloroethane, 126 MHz, 120 °C) of the polyethylene obtained in Table 1, entry 3.

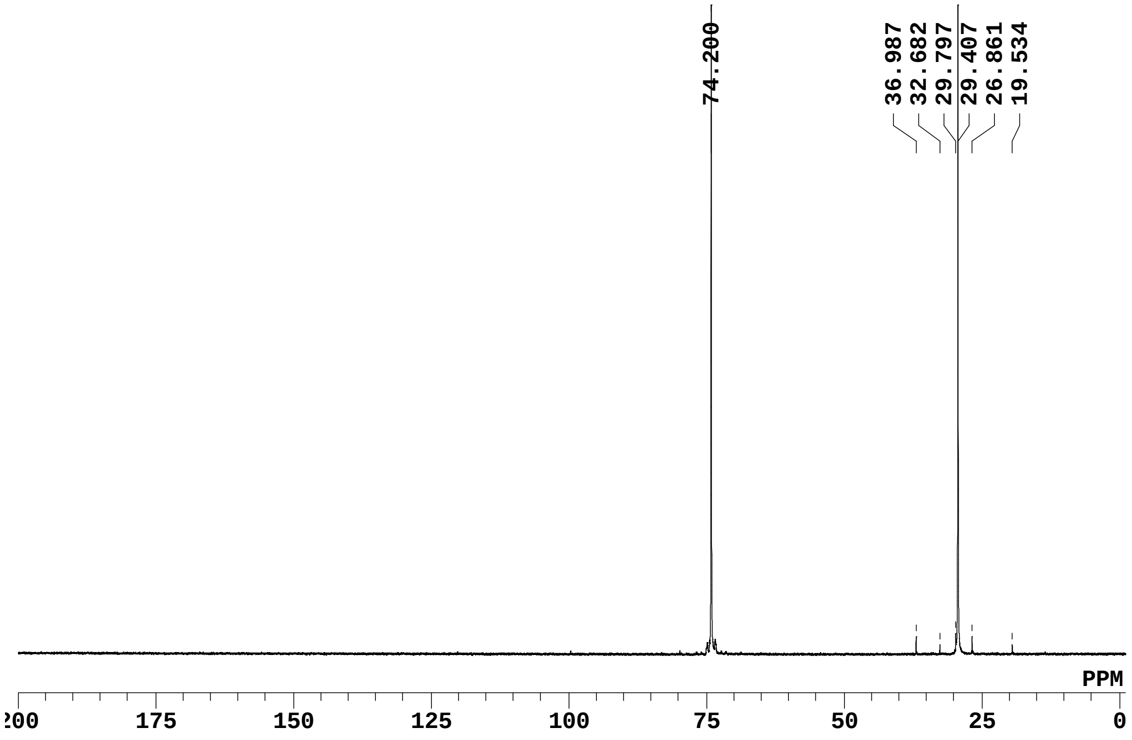

Figure S106. Quantitative ^{13}C NMR spectrum (1,1,2,2-tetrachloroethane, 101 MHz, 130 °C) of the polyethylene obtained in Table 2, entry 1.

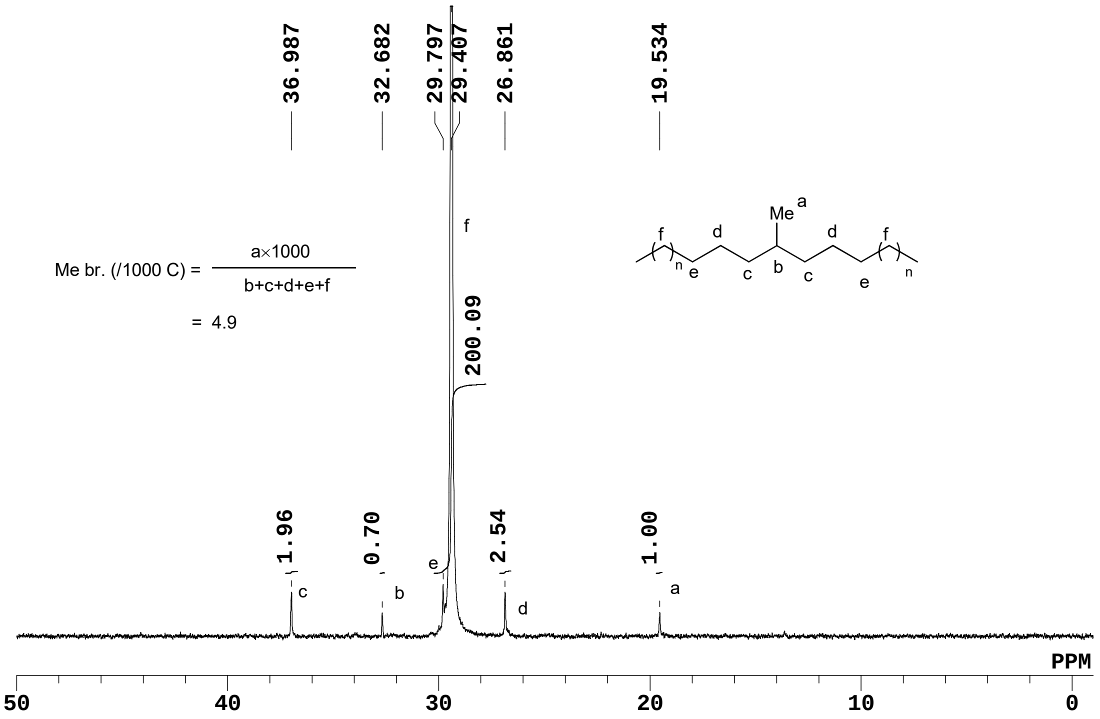

Figure S107. High field region of quantitative ^{13}C NMR spectrum (1,1,2,2-tetrachloroethane, 101 MHz, 130 °C) of the polyethylene obtained in Table 2, entry 1.

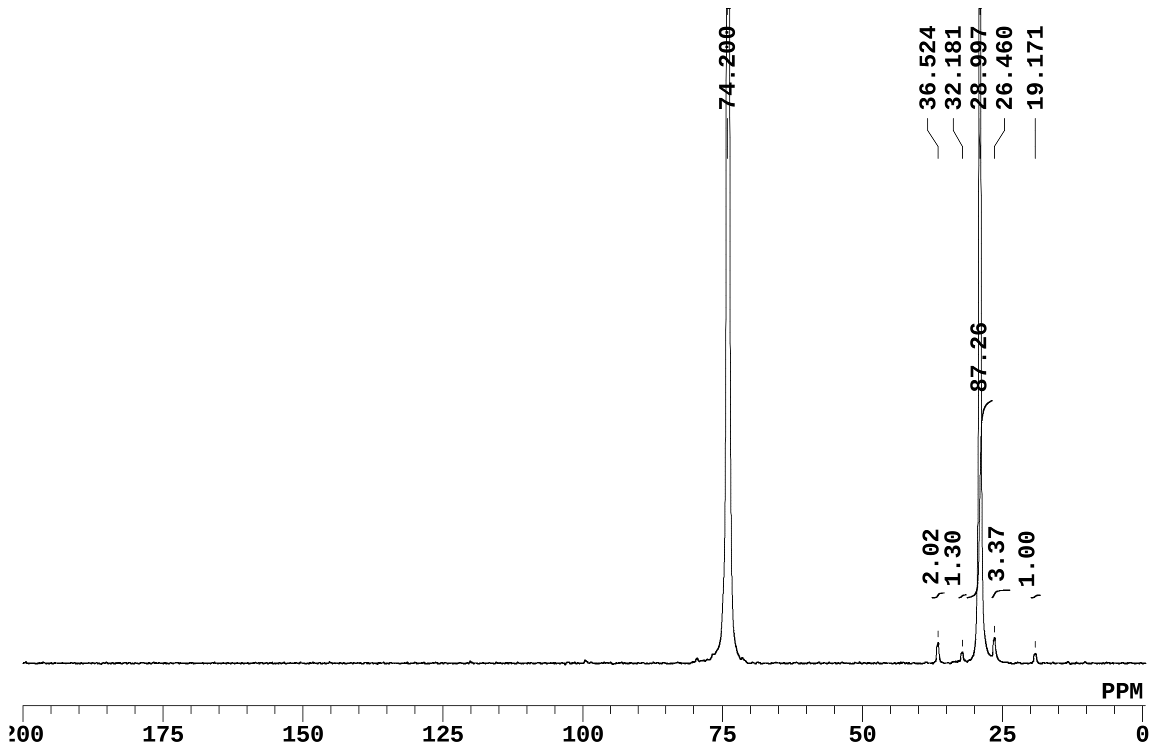

Figure S108. Quantitative ^{13}C NMR spectrum (1,1,2,2-tetrachloroethane, 101 MHz, 120 °C) of the polyethylene obtained in Table 2, entry 2.

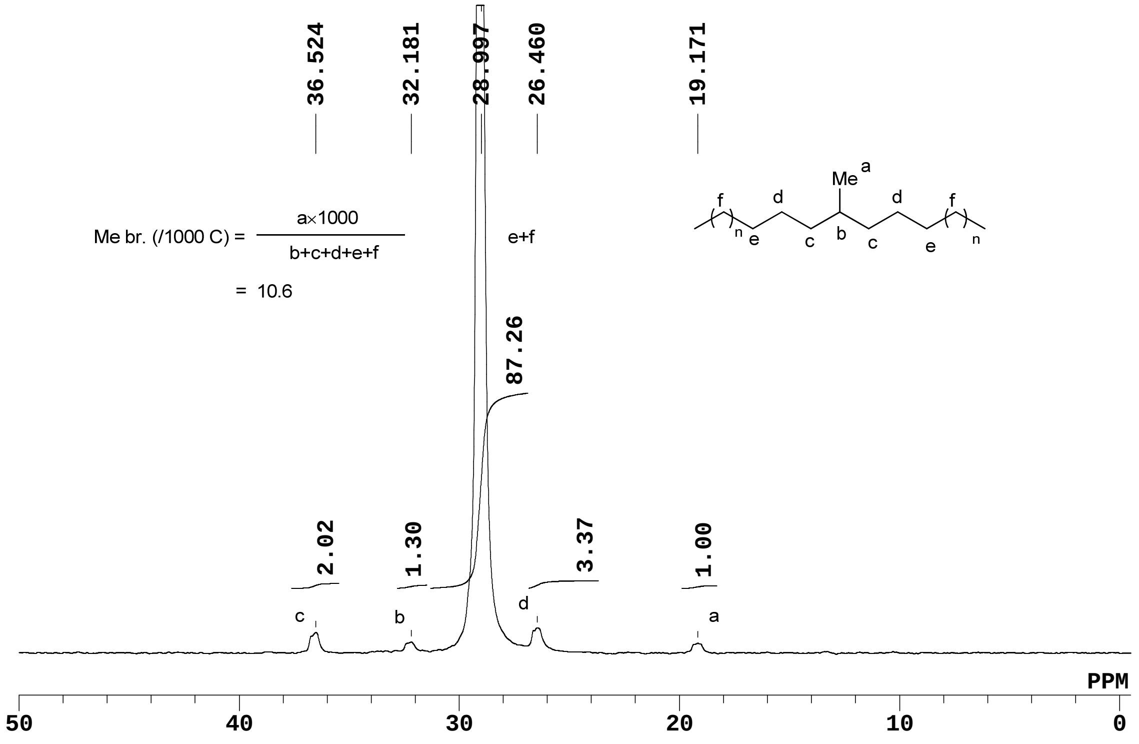

Figure S109. High field region of quantitative ^{13}C NMR spectrum (1,1,2,2-tetrachloroethane, 101 MHz, 120 °C) of the polyethylene obtained in Table 2, entry 2.

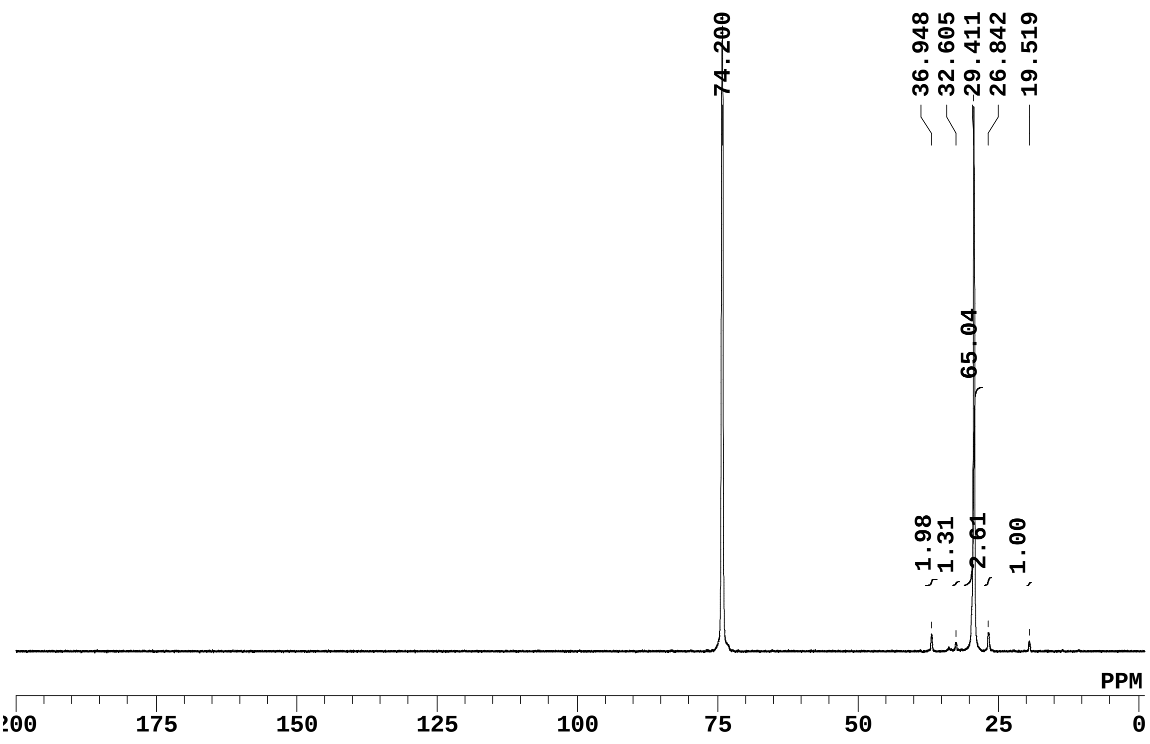

Figure S110. Quantitative ^{13}C NMR spectrum (1,1,2,2-tetrachloroethane, 101 MHz, 120 °C) of the polyethylene obtained in Table 2, entry 3.

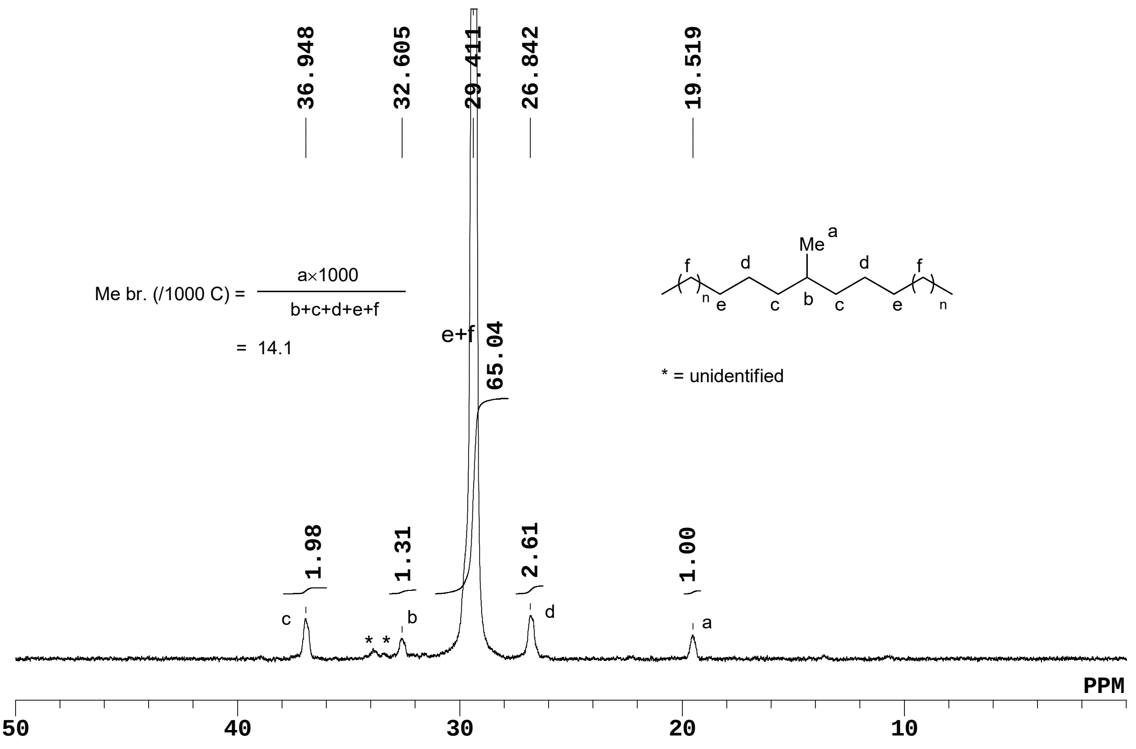

Figure S111. High field region of quantitative ^{13}C NMR spectrum (1,1,2,2-tetrachloroethane, 101 MHz, 120 °C) of the polyethylene obtained in Table 2, entry 3.

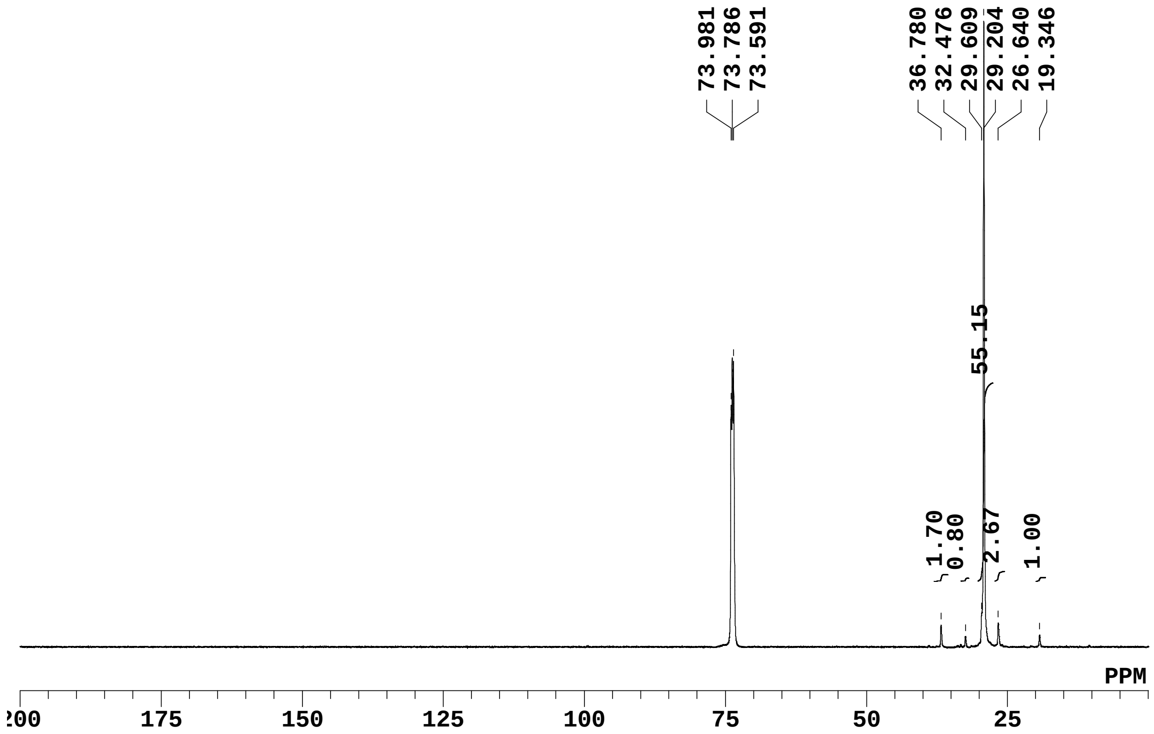

Figure S112. Quantitative ^{13}C NMR spectrum ($C_2D_2Cl_4$, 126 MHz, 120 °C) of the polyethylene obtained in Table 2, entry 4.

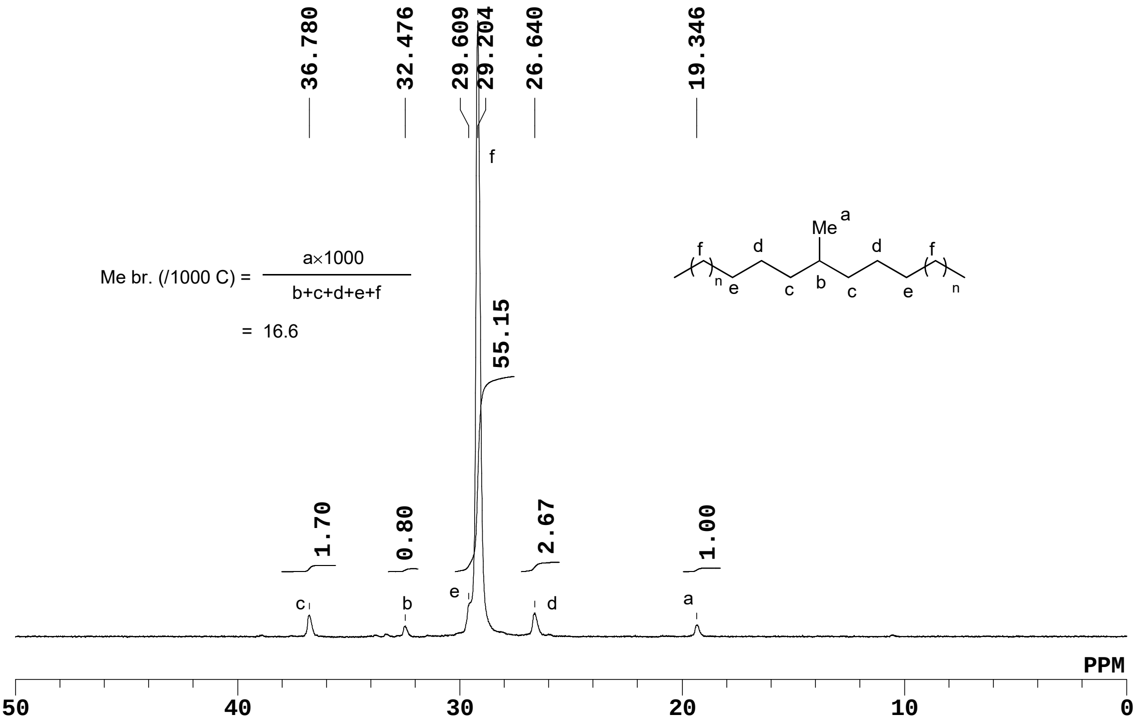

Figure S113. High field region of quantitative ^{13}C NMR spectrum ($C_2D_2Cl_4$, 126 MHz, 120 °C) of the polyethylene obtained in Table 2, entry 4.

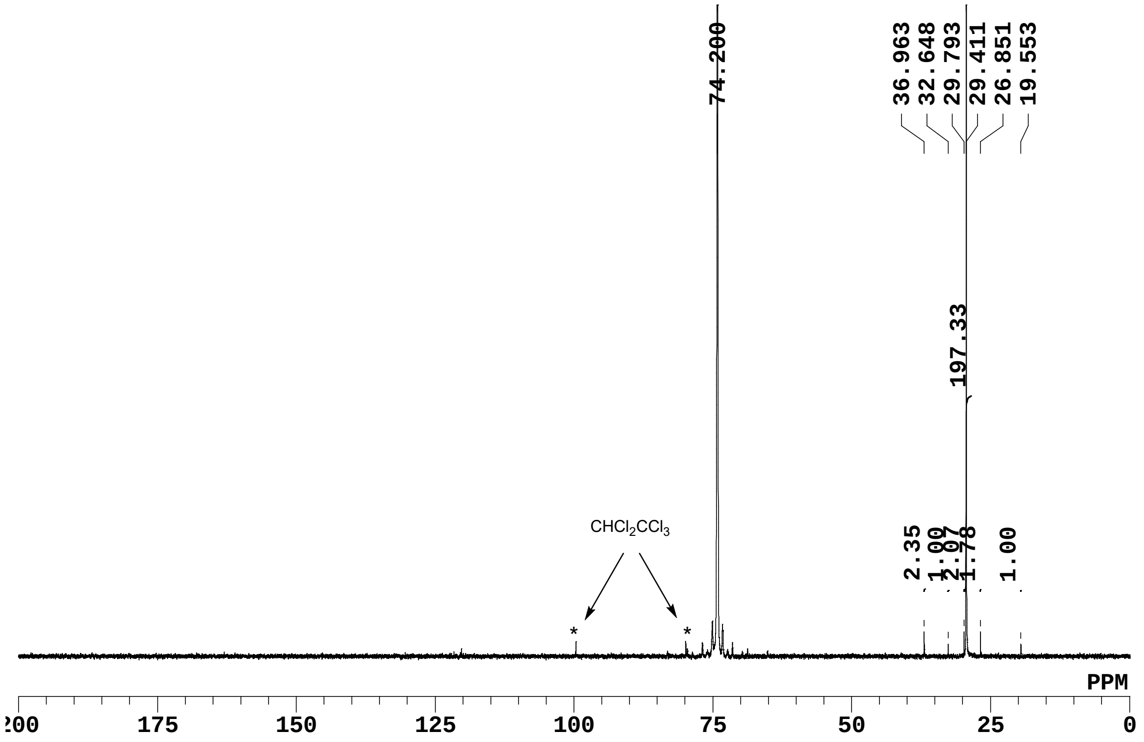

Figure S114. Quantitative ^{13}C NMR spectrum (1,1,2,2-tetrachloroethane, 101 MHz, 120 °C) of the polyethylene obtained in Table 2, entry 5.

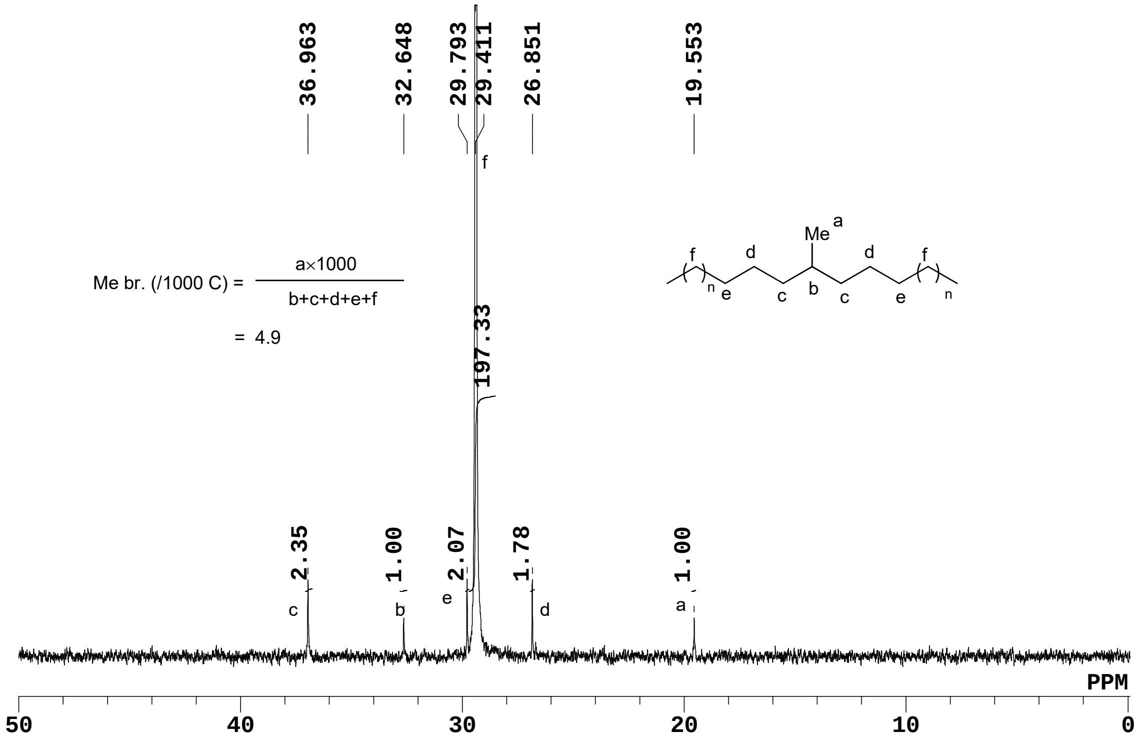

Figure S115. High field region of quantitative ^{13}C NMR spectrum (1,1,2,2-tetrachloroethane, 101 MHz, 120 °C) of the polyethylene obtained in Table 2, entry 5.

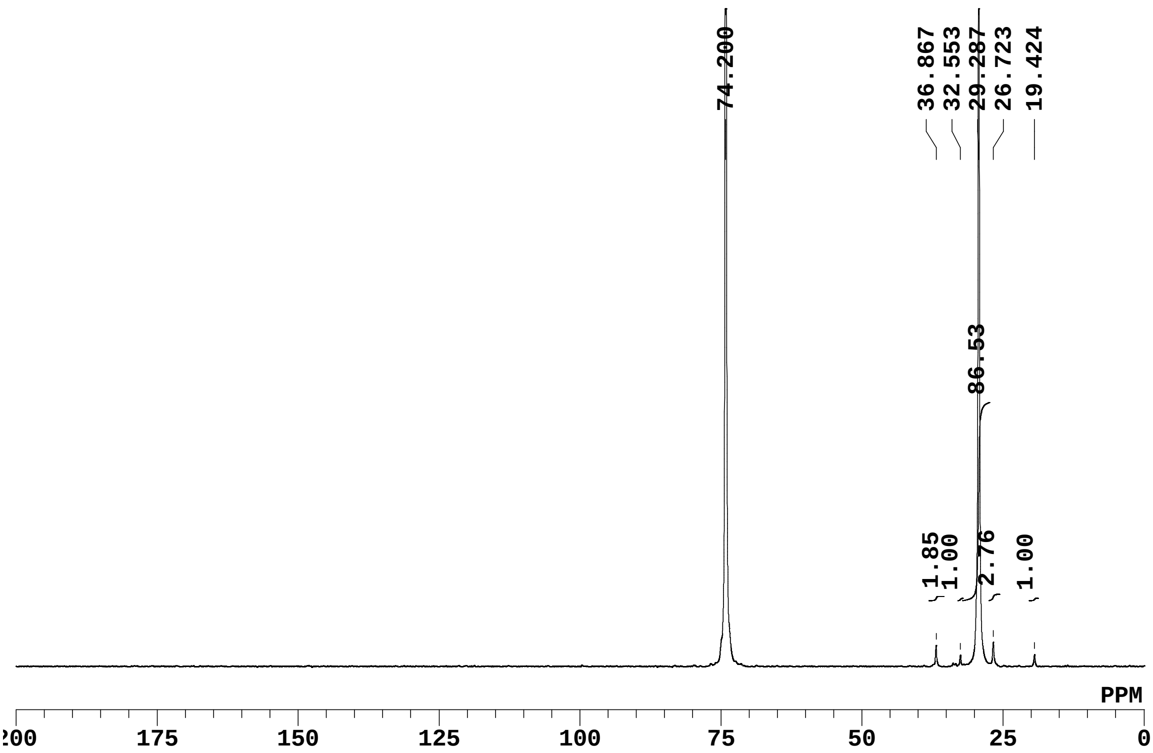

Figure S116. Quantitative ^{13}C NMR spectrum of the polyethylene (1,1,2,2-tetrachloroethane, 101 MHz, 130 °C) obtained in Table 2, entry 6.

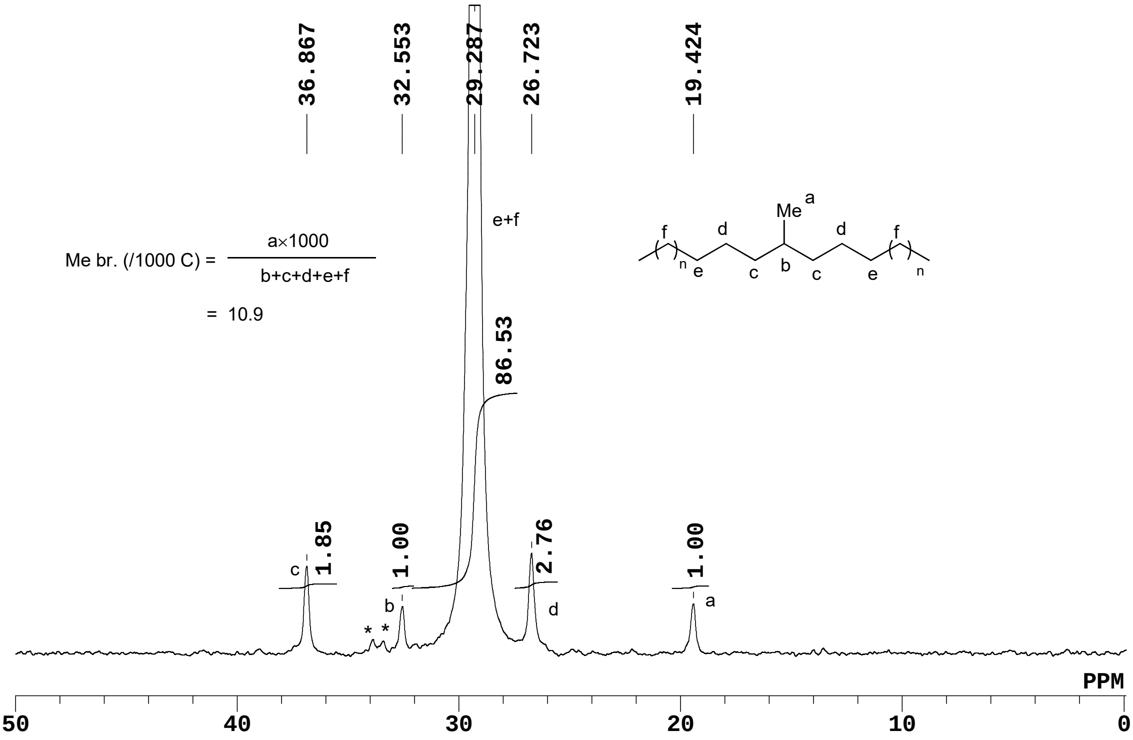

Figure S117. High field region of quantitative ^{13}C NMR spectrum (1,1,2,2-tetrachloroethane, 101 MHz, 130 °C) of the polyethylene obtained in Table 2, entry 6.

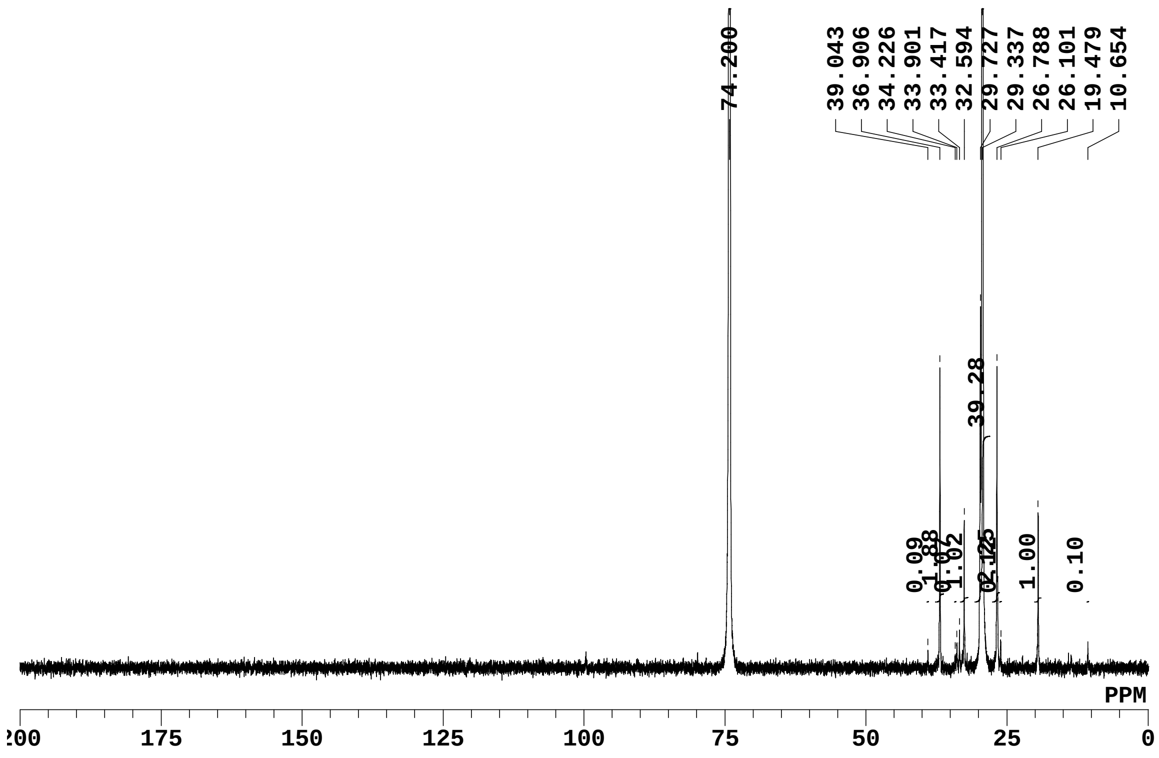

Figure S118. Quantitative ^{13}C NMR spectrum (1,1,2,2-tetrachloroethane, 126 MHz, 120 °C) of the polyethylene obtained in Table 2, entry 7.

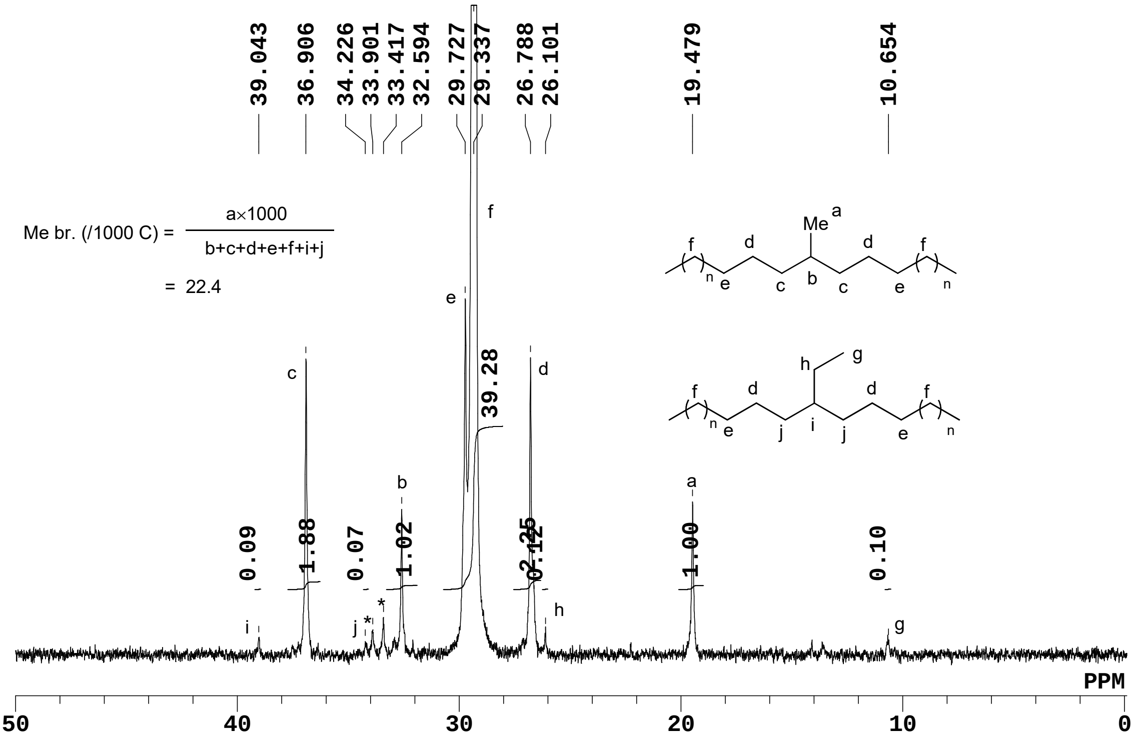

Figure S119. High field region of quantitative ^{13}C NMR spectrum (1,1,2,2-tetrachloroethane, 126 MHz, 120 °C) of the polyethylene obtained in Table 2, entry 7.

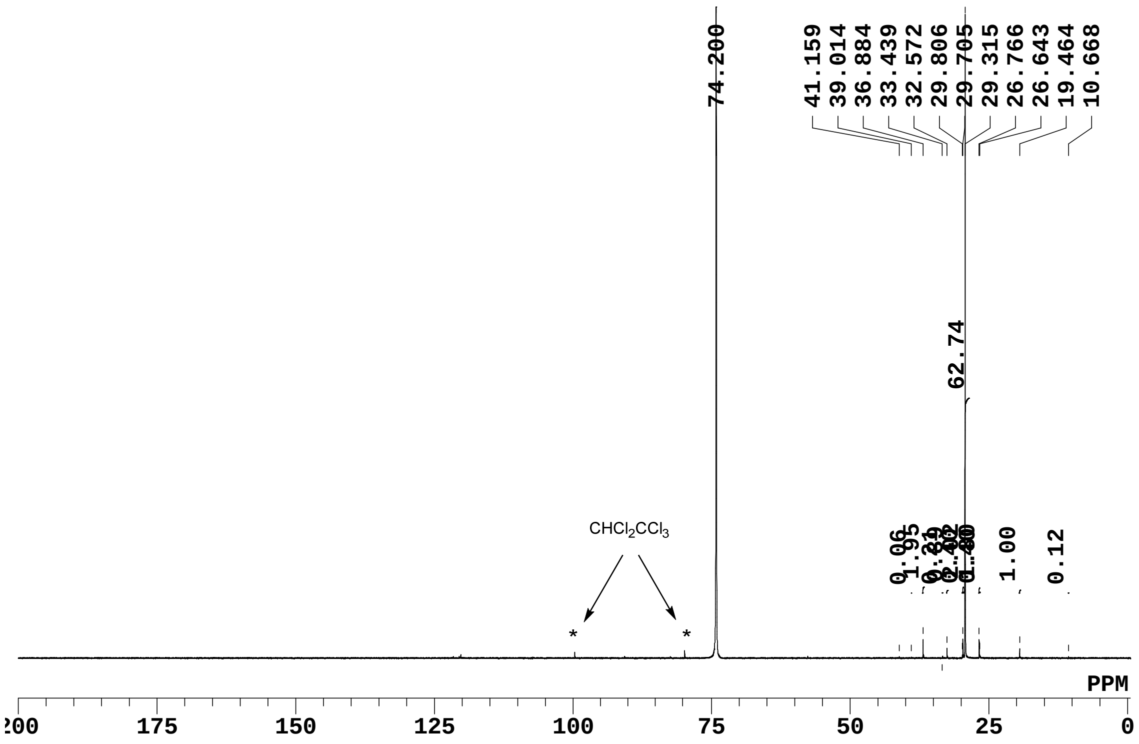

Figure S120. Quantitative ^{13}C NMR spectrum (1,1,2,2-tetrachloroethane, 126 MHz, 120 °C) of the polyethylene obtained in Table 2, entry 8.

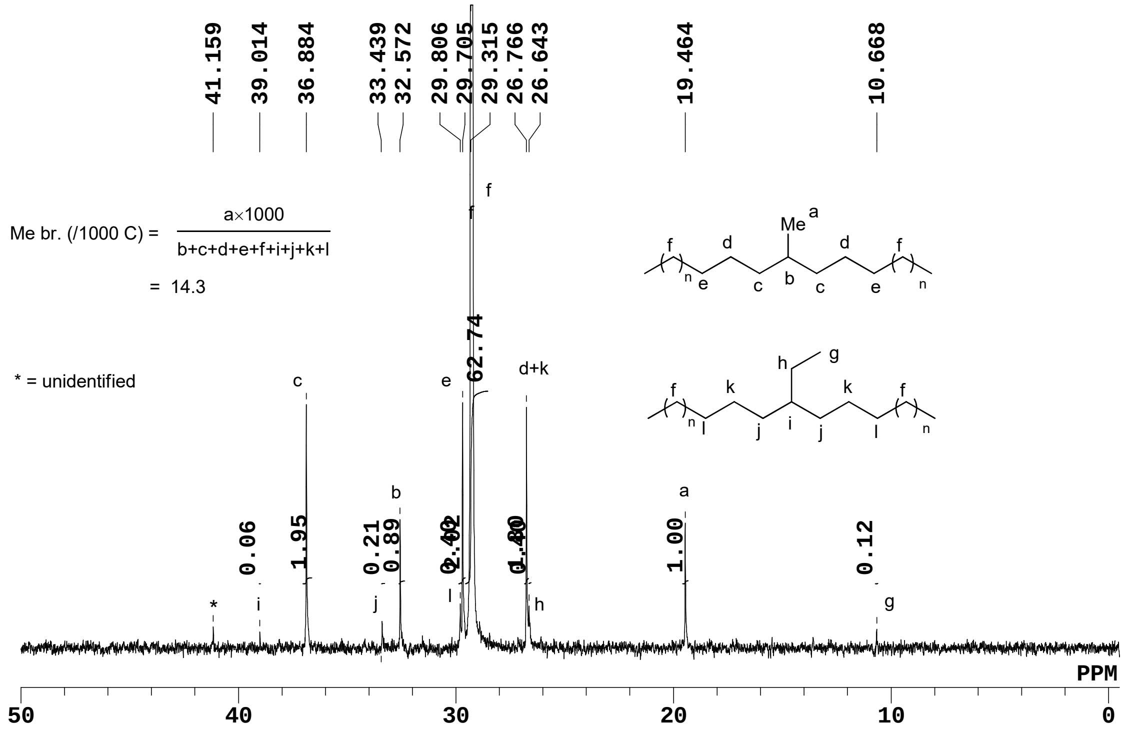

Figure S121. High field region of quantitative ^{13}C NMR spectrum (1,1,2,2-tetrachloroethane, 126 MHz, 120 °C) of the polyethylene obtained in Table 2, entry 8.

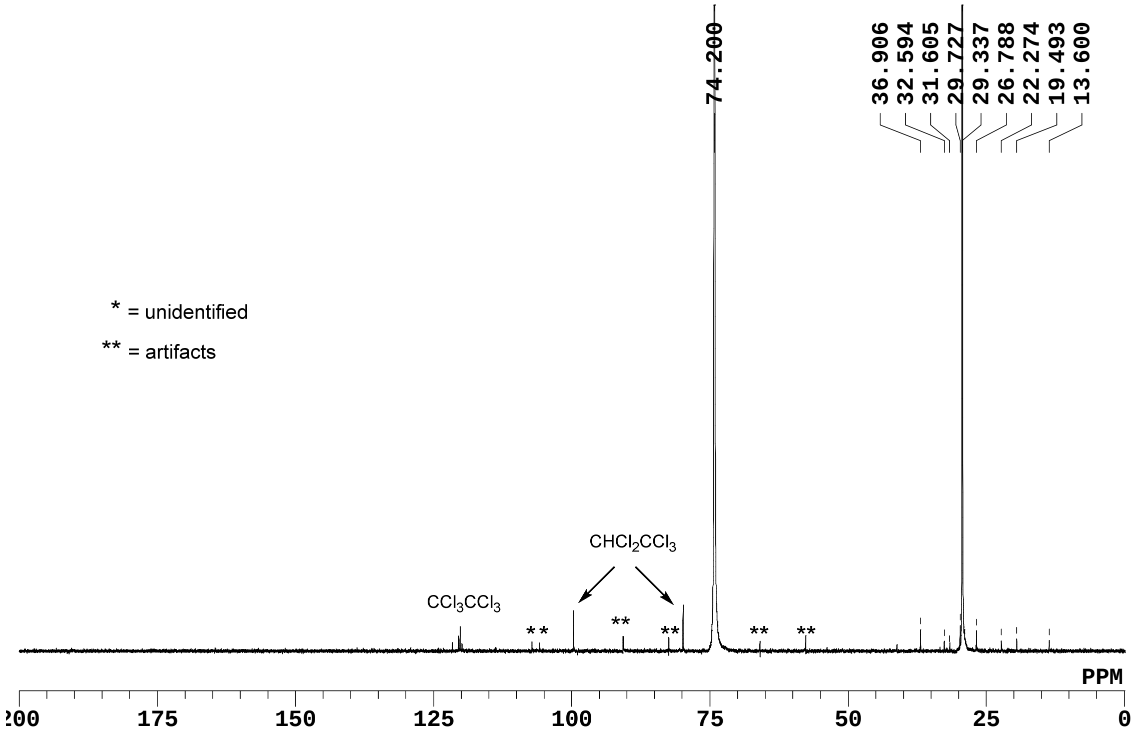

Figure S122. Quantitative ^{13}C NMR spectrum (1,1,2,2-tetrachloroethane, 126 MHz, 120 °C) of the polyethylene obtained in Table 2, entry 9.

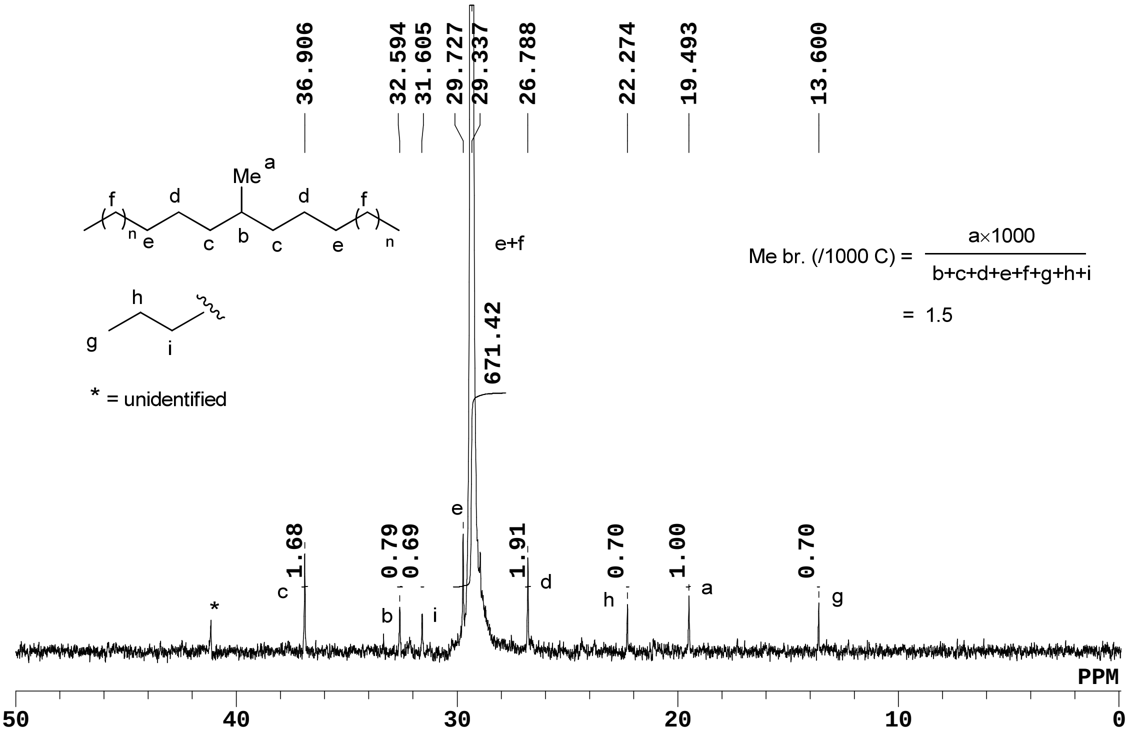

Figure S123. High field region of quantitative ^{13}C NMR spectrum (1,1,2,2-tetrachloroethane, 126 MHz, 120 °C) of the polyethylene obtained in Table 2, entry 9.

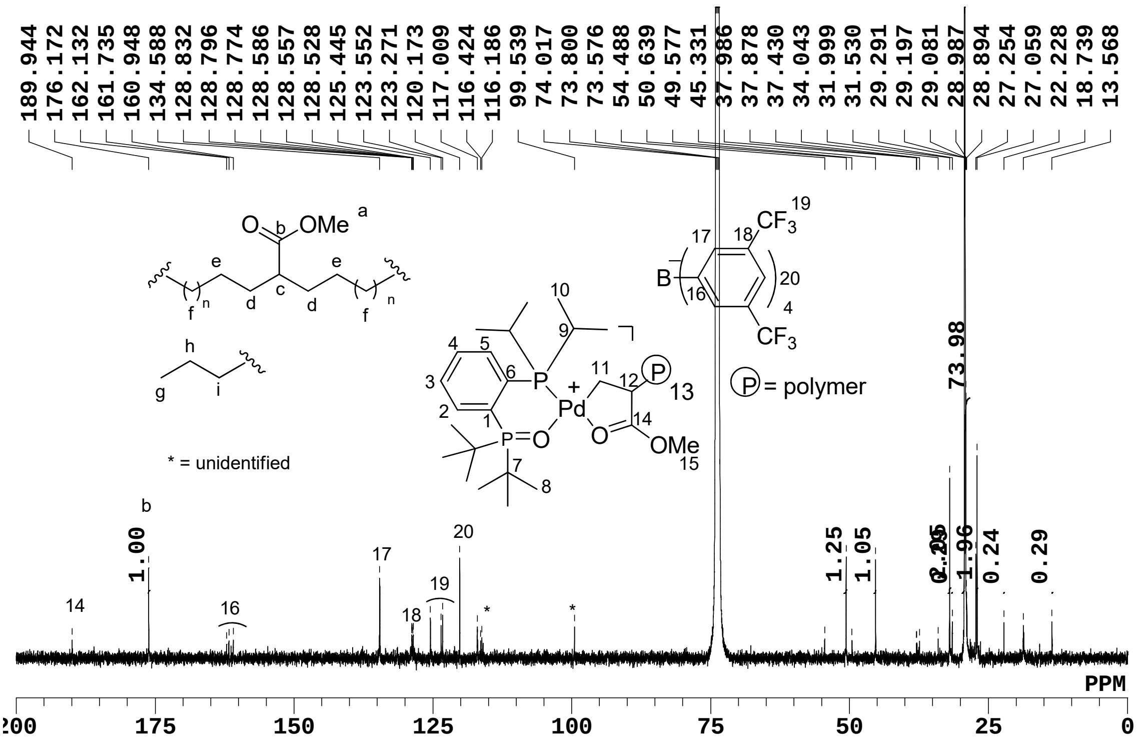

Figure S124. Quantitative ^{13}C NMR spectrum ($C_2D_2Cl_4$, 126 MHz, 120 °C) of the ethylene/methyl acrylate copolymer obtained in Table 3, entry 1.

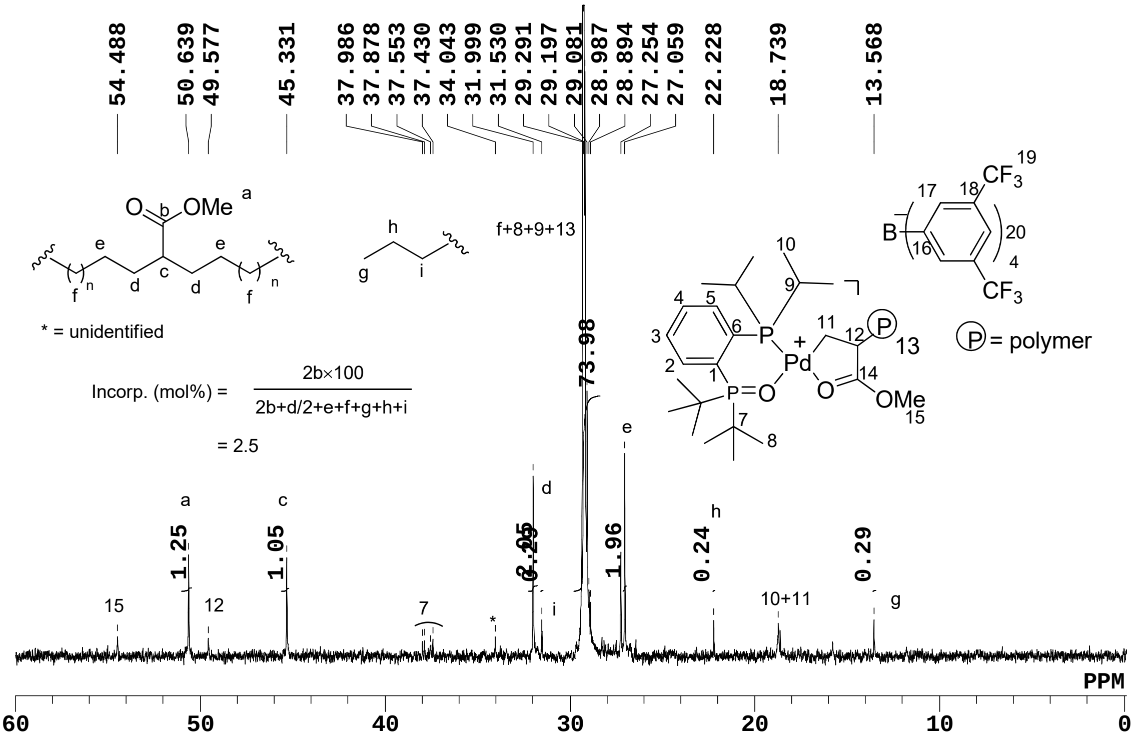

Figure S125. High field region of quantitative ^{13}C NMR spectrum ($C_2D_2Cl_4$, 126 MHz, 120 °C) of the polyethylene obtained in Table 3, entry 1.

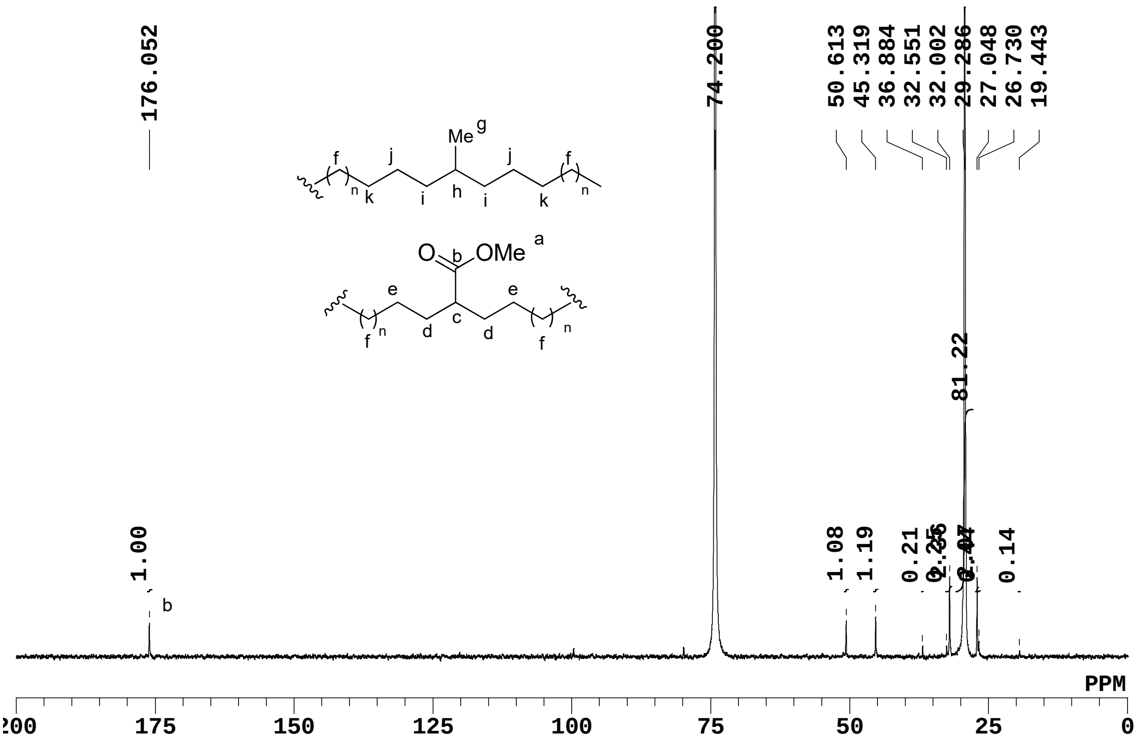

Figure S126. Quantitative ^{13}C NMR spectrum (1,1,2,2-tetrachloroethane, 126 MHz, 120 °C) of the polyethylene obtained in Table 3, entry 3.

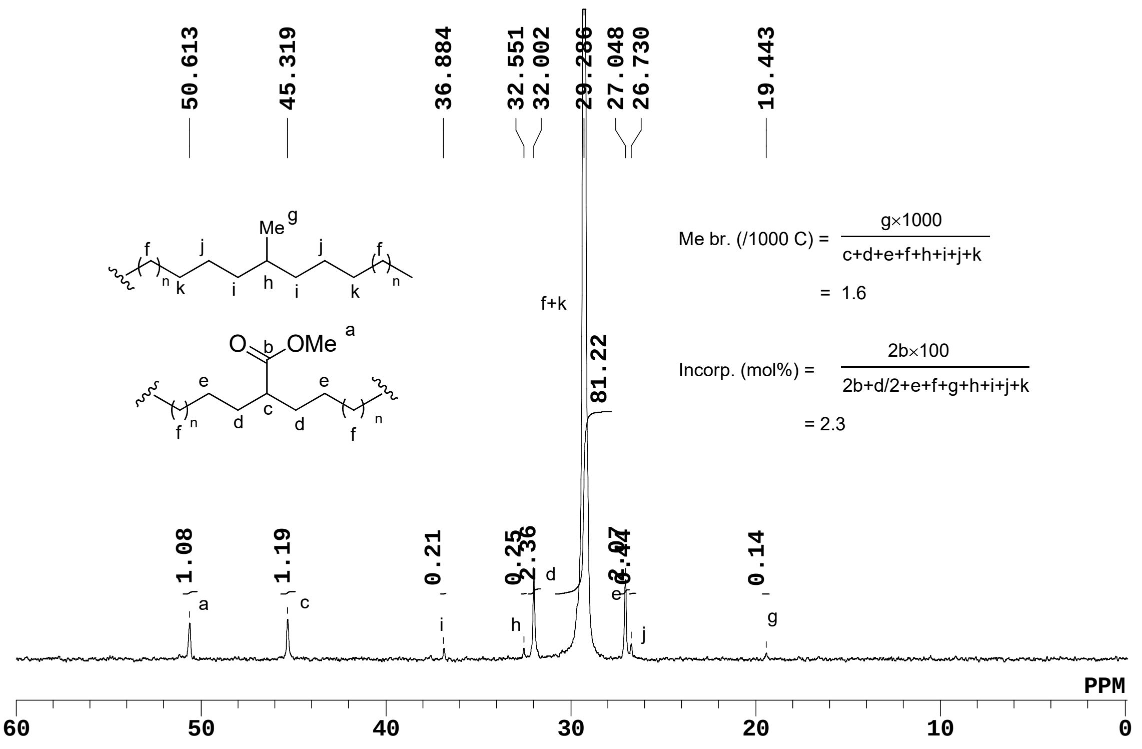

Figure S127. High field region of quantitative ^{13}C NMR spectrum (1,1,2,2-tetrachloroethane, 126 MHz, 120 °C) of the polyethylene obtained in Table 3, entry 3.

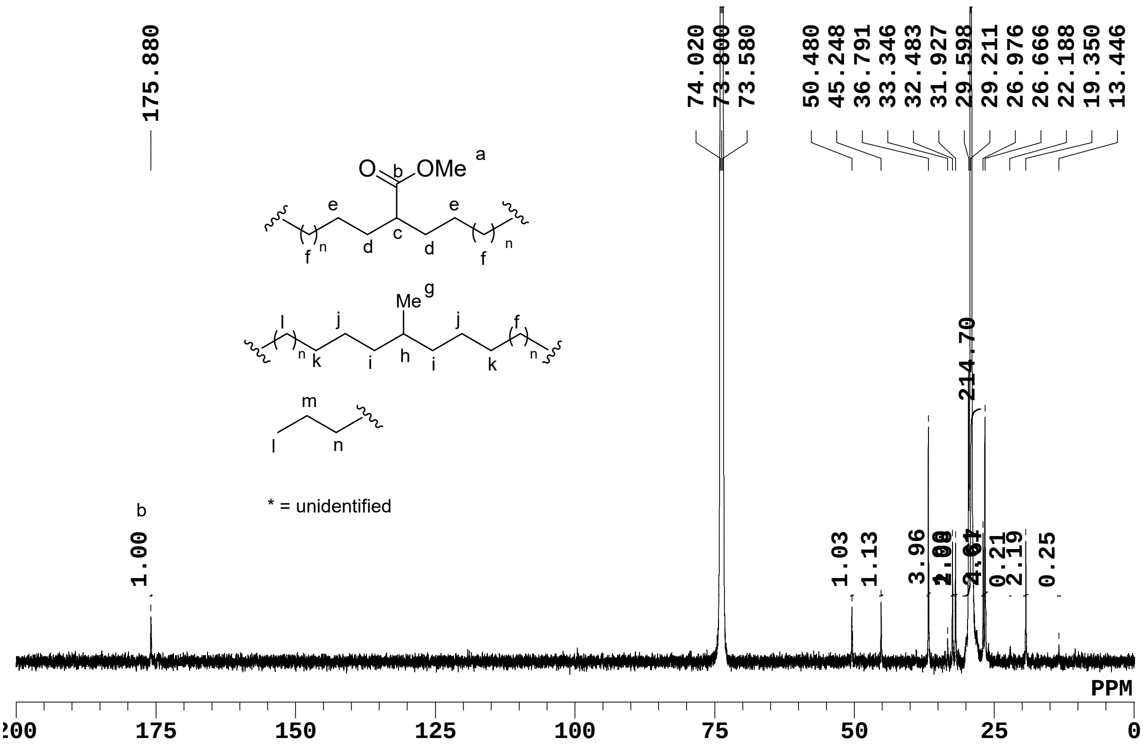

Figure S128. Quantitative ^{13}C NMR spectrum ($C_2D_2Cl_4$, 126 MHz, 120 °C) of the ethylene/methyl acrylate copolymer obtained in Table 3, entry 4.

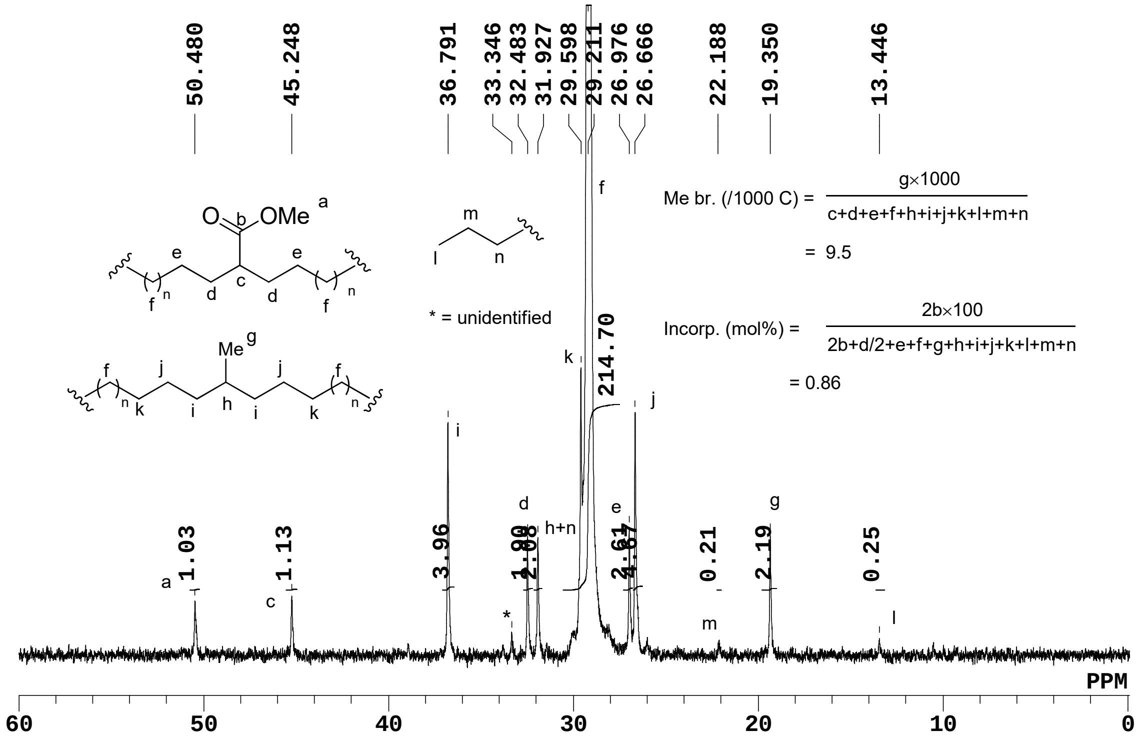

Figure S129. High field region of the quantitative ^{13}C NMR spectrum ($C_2D_2Cl_4$, 126 MHz, 120 °C) of the ethylene/methyl acrylate copolymer obtained in Table 3, entry 4.

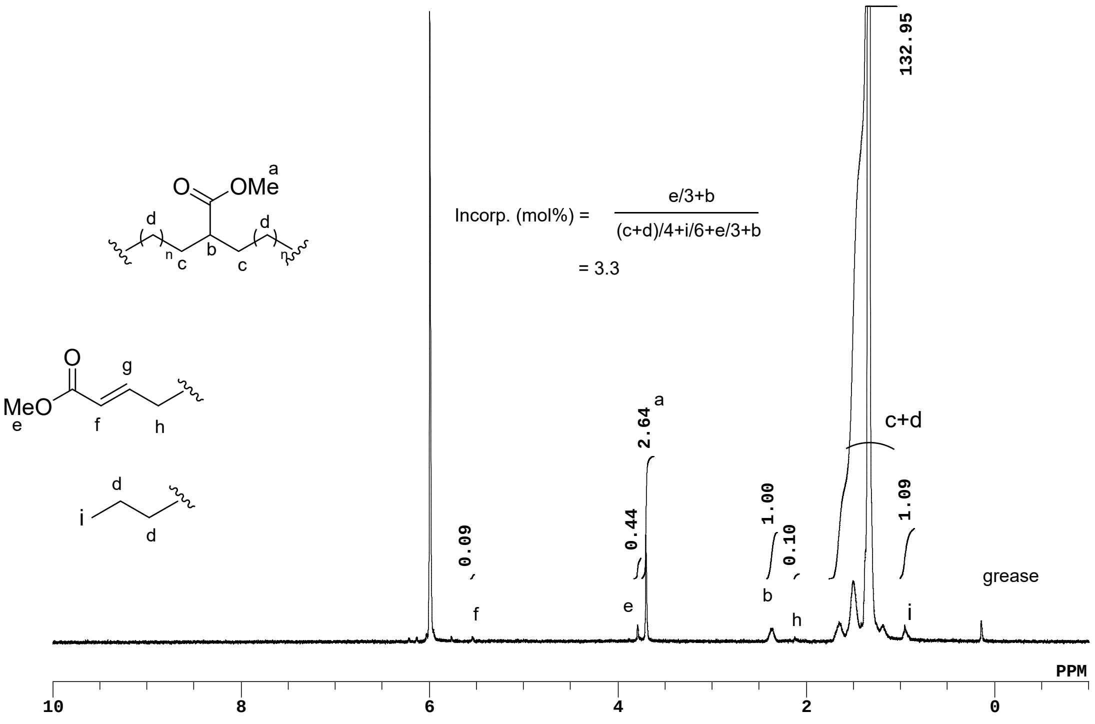

Figure S130. ^{1}H NMR spectrum ($C_2D_2Cl_4$, 126 MHz, 120 °C) of the ethylene/methyl acrylate copolymer obtained in Table 3, entry 5.

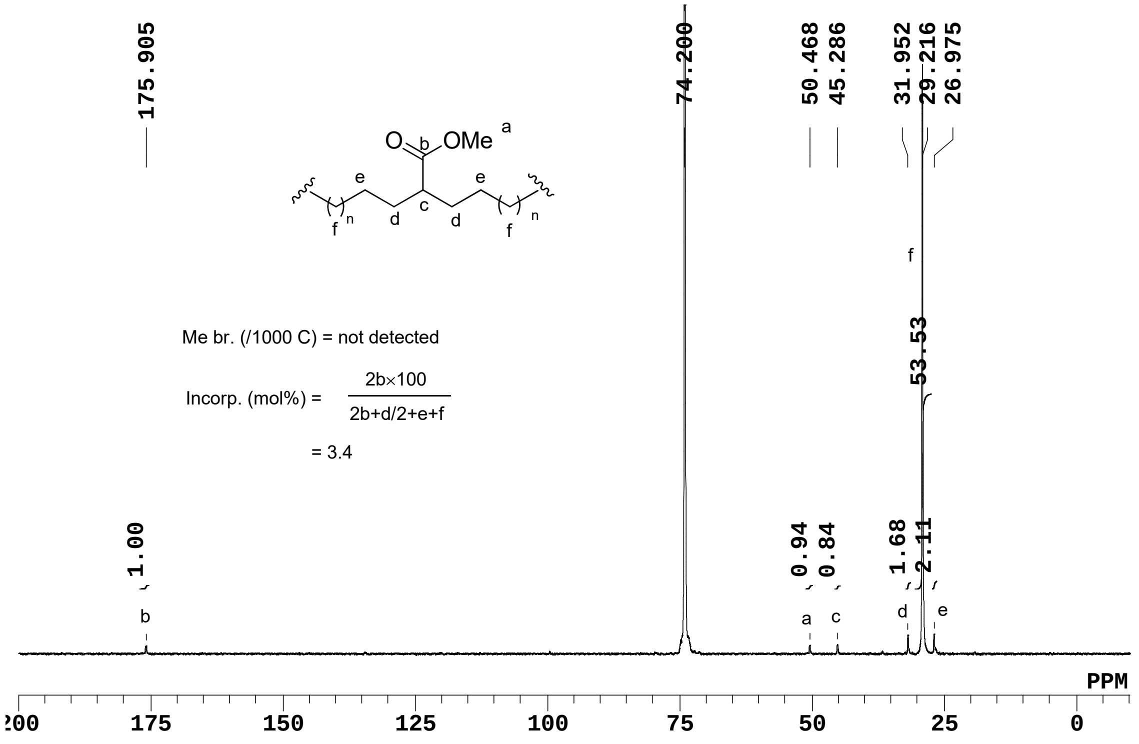

Figure S131. Quantitative ^{13}C NMR spectrum (1,1,2,2-tetrachloroethane, 101 MHz, 120 °C)of the ethylene/methyl acrylate copolymer obtained in Table 3, entry 6.

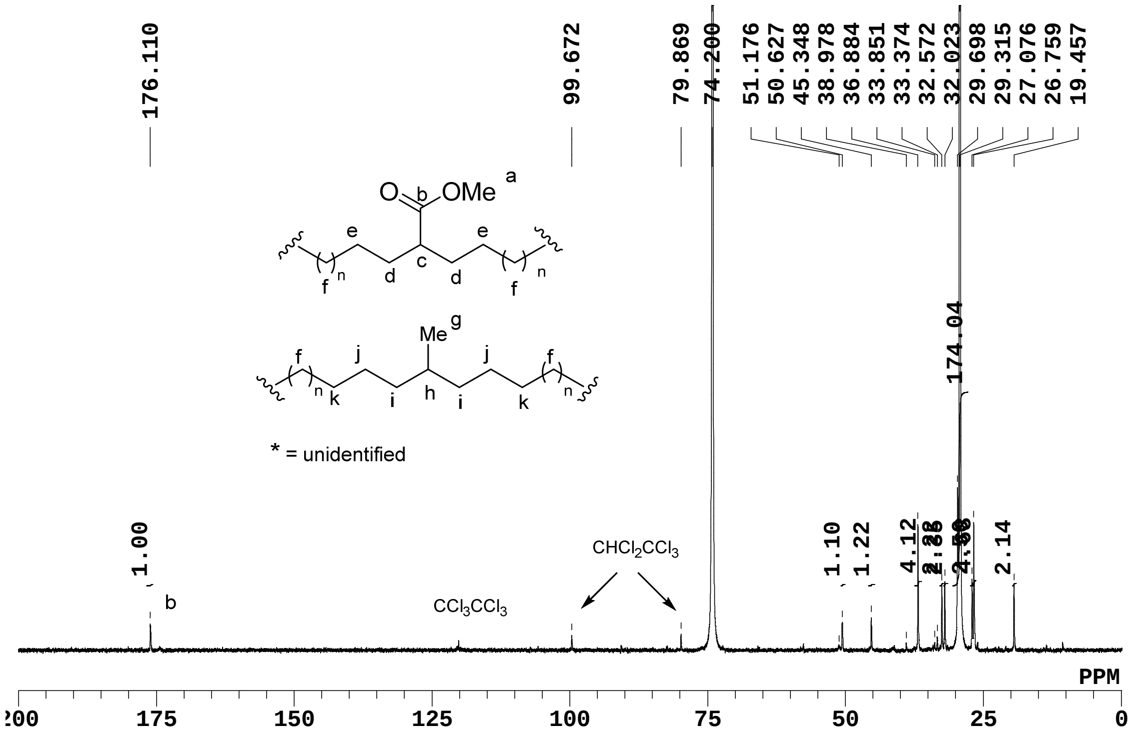

Figure S132. Quantitative ^{13}C NMR spectrum (1,1,2,2-tetrachloroethane, 126 MHz, 120 °C) of the ethylene/methyl acrylate copolymer obtained in Table 3, entry 7.

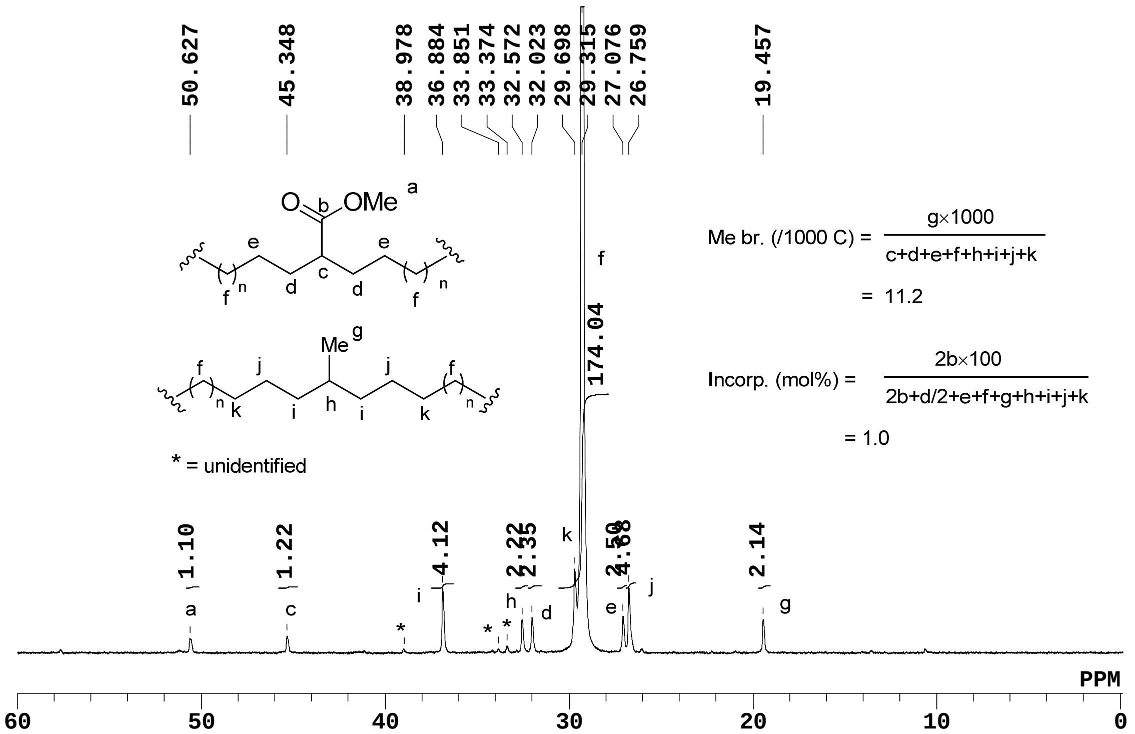

Figure S133. High field region of quantitative ^{13}C NMR spectrum (1,1,2,2-tetrachloroethane, 126 MHz, 120 °C) of the ethylene/methyl acrylate copolymer obtained in Table 3, entry 7.

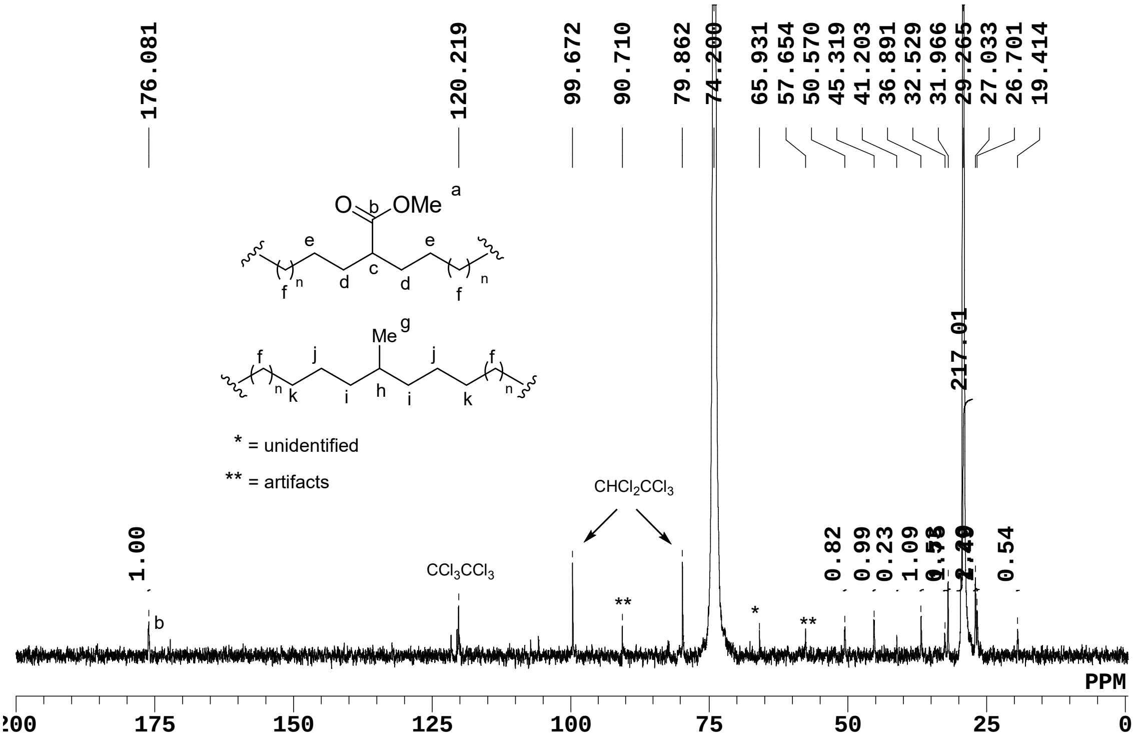

Figure S134. Quantitative ^{13}C NMR spectrum (1,1,2,2-tetrachloroethane, 126 MHz, 120 °C) of the ethylene/methyl acrylate copolymer obtained in Table 3, entry 8.

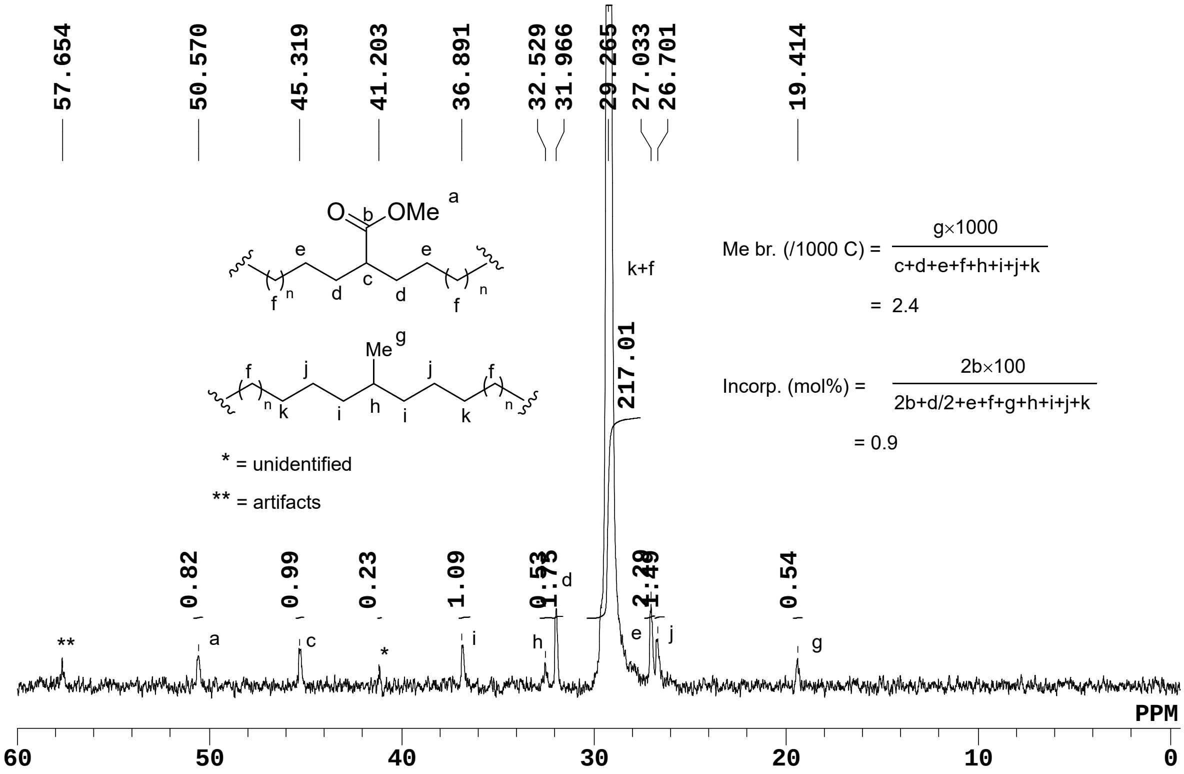

Figure S135. High field region of quantitative ^{13}C NMR spectrum (1,1,2,2-tetrachloroethane, 126 MHz, 120 °C) of the ethylene/methyl acrylate copolymer obtained in Table 3, entry 8.

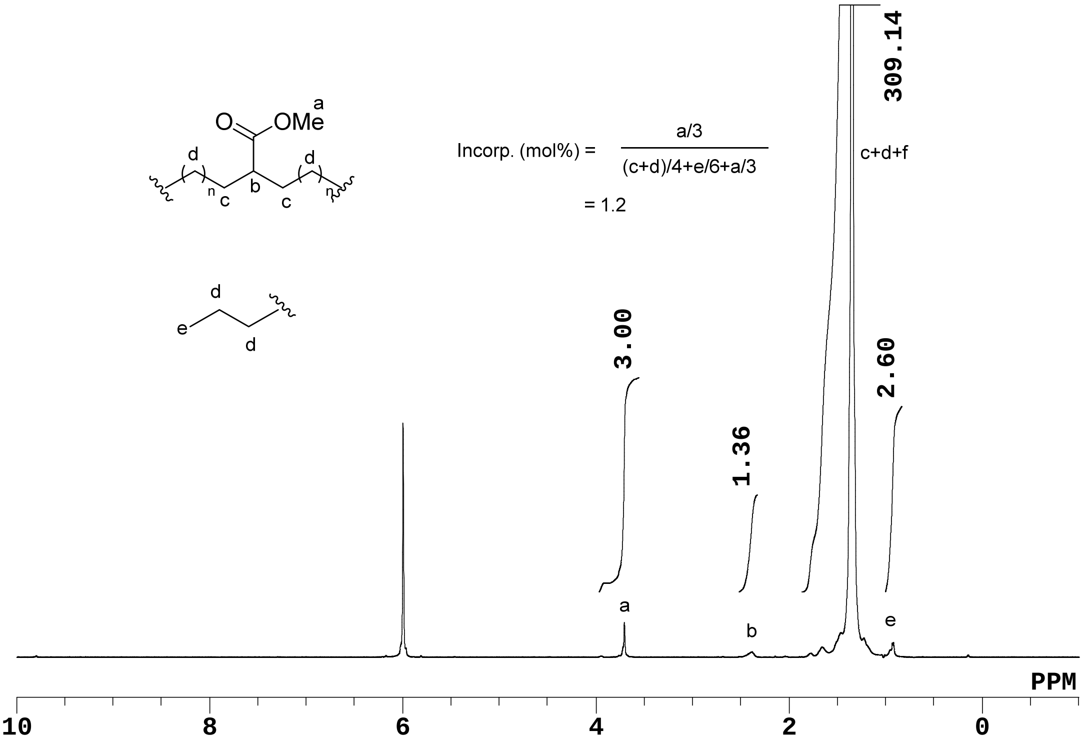

Figure S136. ^{1}H NMR spectrum ($C_2D_2Cl_4$, 500 MHz, 120 °C) of the ethylene/methyl acrylate copolymer obtained in Table 3, entry 9.

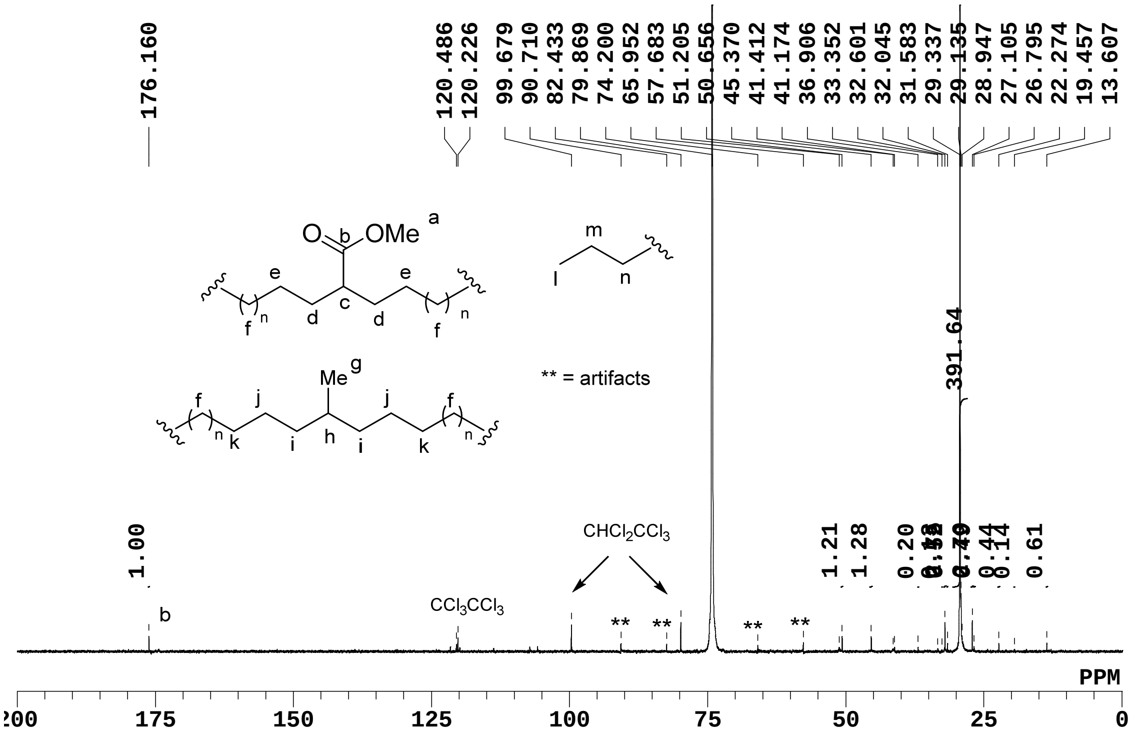

Figure S137. Quantitative ^{13}C NMR spectrum (1,1,2,2-tetrachloroethane, 126 MHz, 120 °C) of the ethylene/methyl acrylate copolymer obtained in Table 3, entry 10.

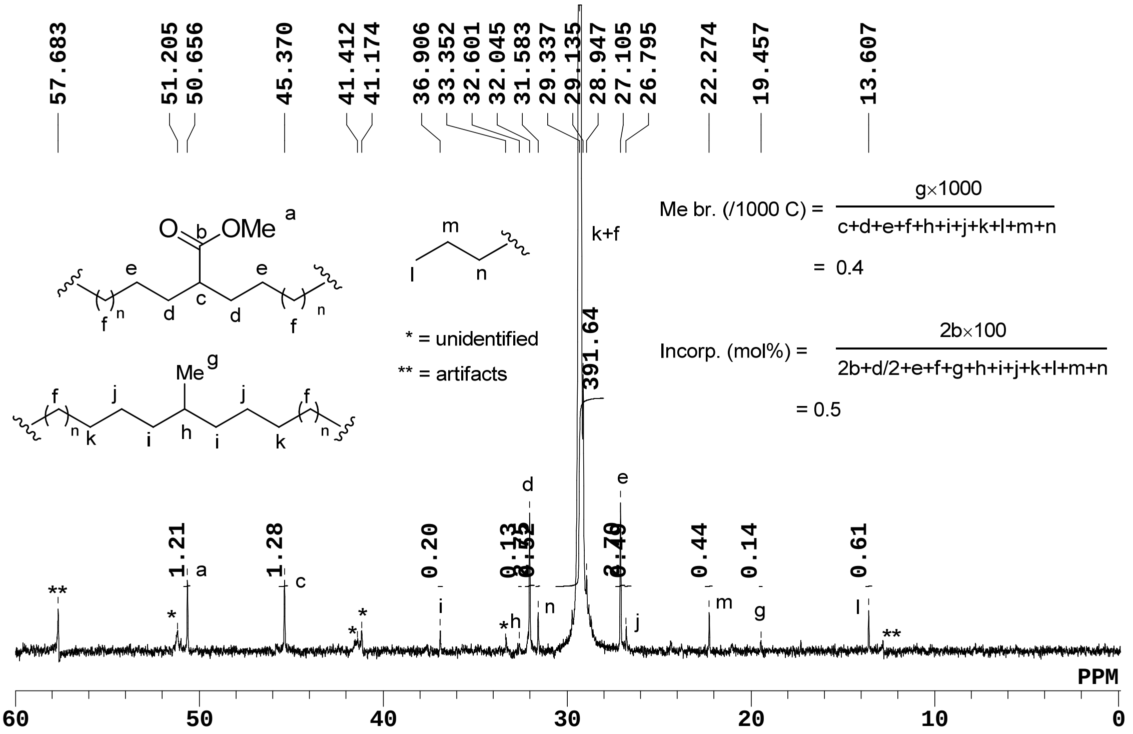

Figure S138. High field region of quantitative ^{13}C NMR spectrum (1,1,2,2-tetrachloroethane, 126 MHz, 120 °C) of the ethylene/methyl acrylate copolymer obtained in Table 3, entry 10.

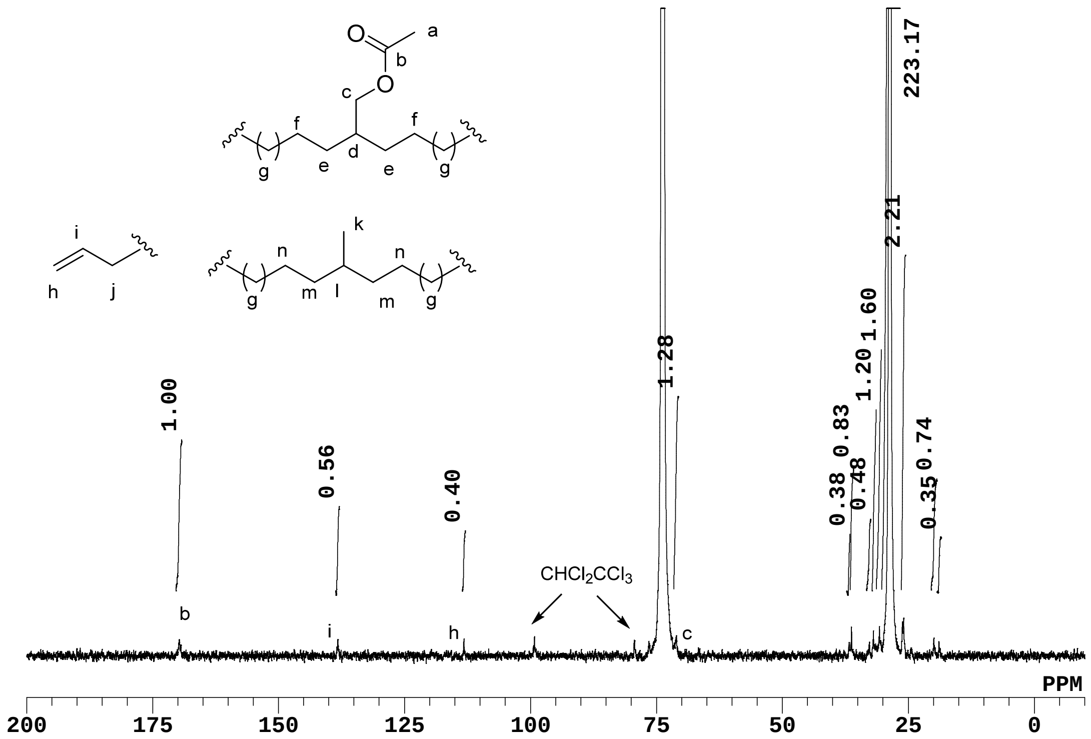

Figure S139. Quantitative ^{13}C NMR spectrum (1,1,2,2-tetrachloroethane, 101 MHz, 120 °C) of the ethylene/allyl acetate copolymer obtained in Table 4, entry 1.

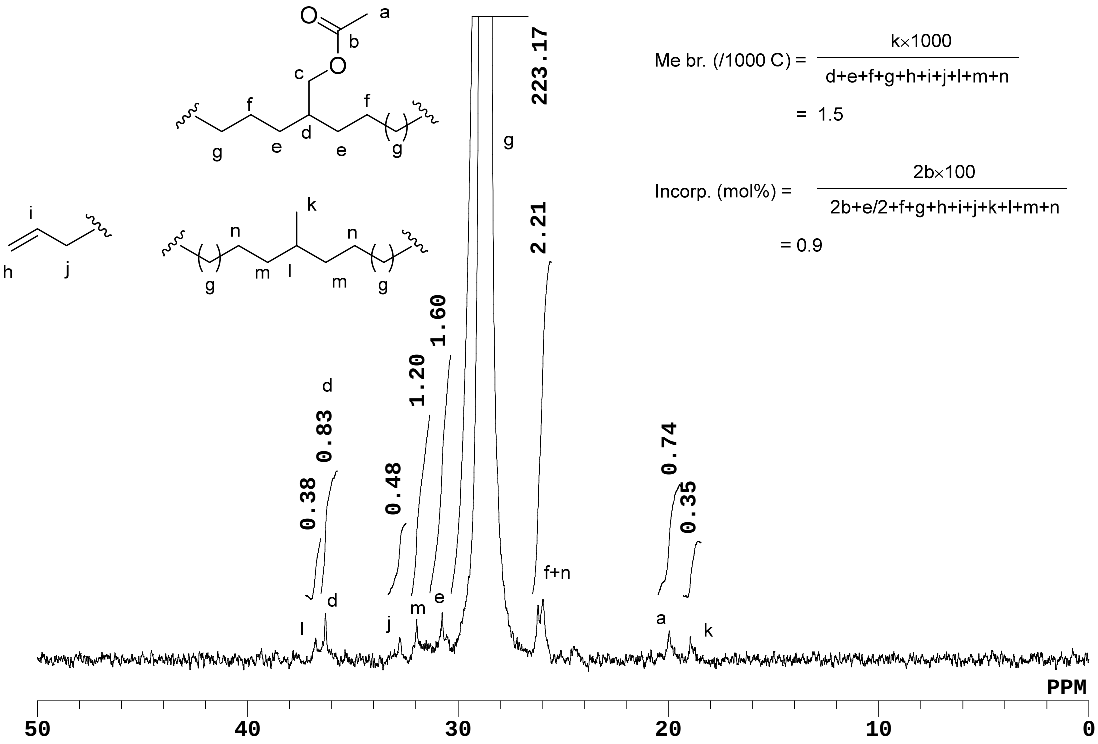

Figure S140. High field region of quantitative ^{13}C NMR spectrum (1,1,2,2-tetrachloroethane, 101 MHz, 120 °C) of the ethylene/allyl acetate copolymer obtained in Table 4, entry 1.

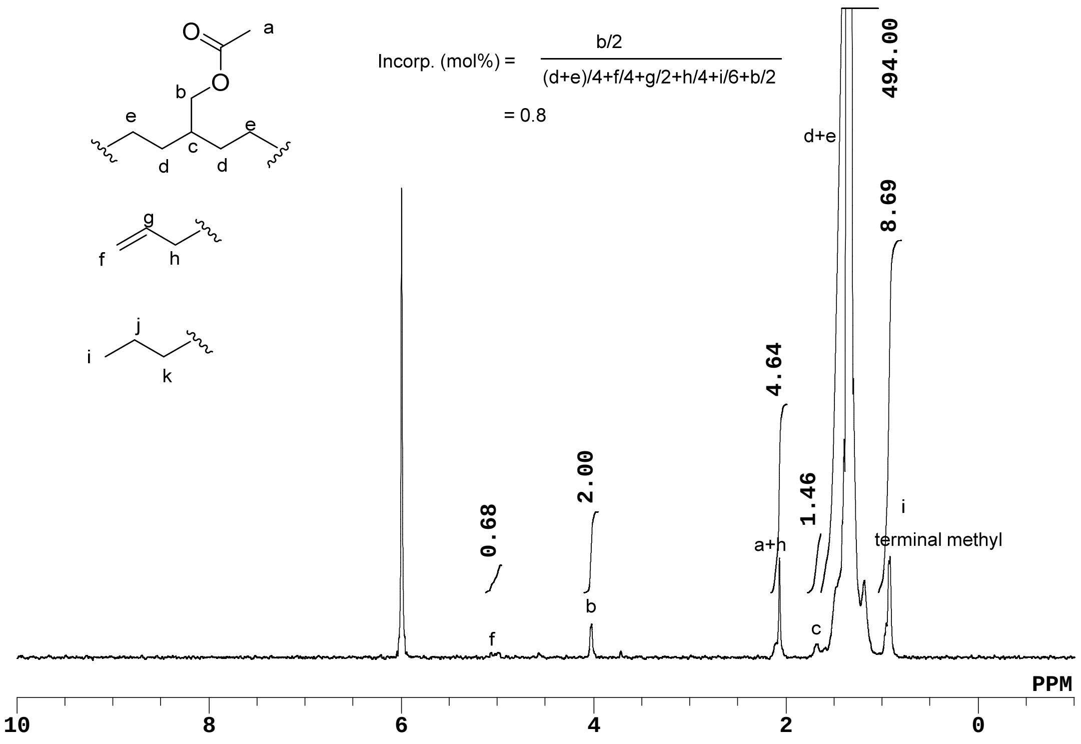

Figure S141. ^{1}H NMR spectrum ($C_2D_2Cl_4$, 400 MHz, 120 °C) of the ethylene/allyl acetate copolymer obtained in Table 4, entry 2.

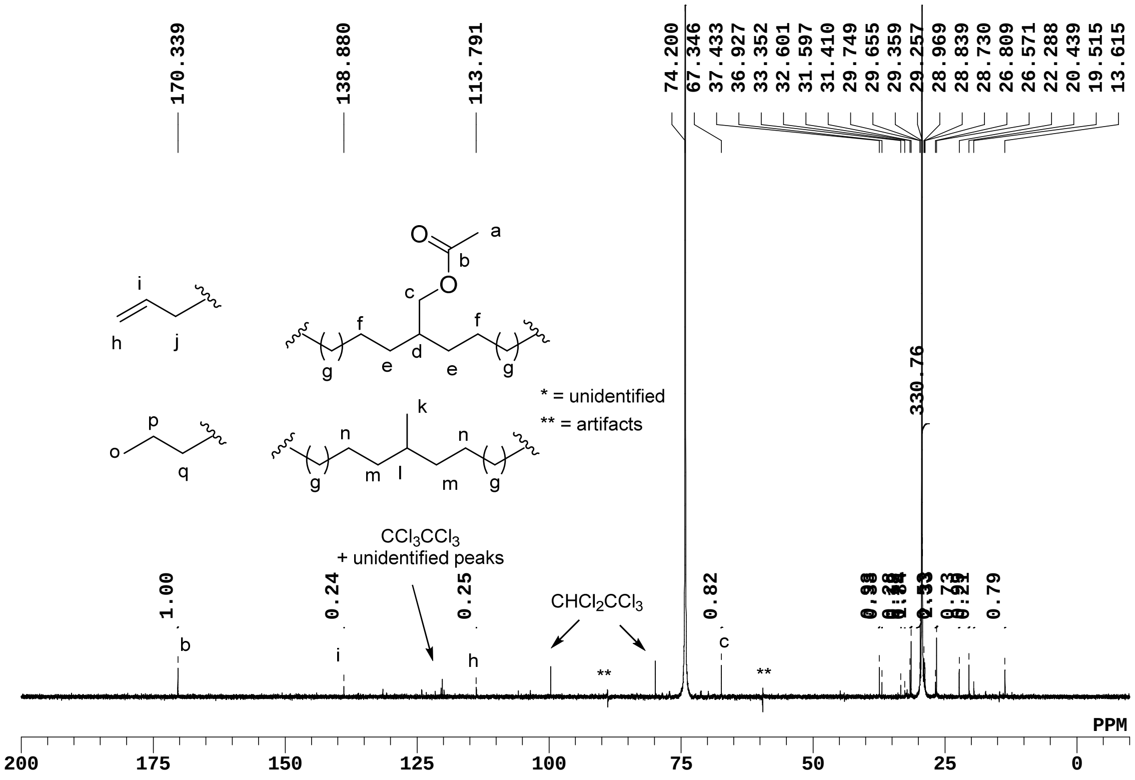

Figure S142. Quantitative ^{13}C NMR spectrum (1,1,2,2-tetrachloroethane, 126 MHz, 120 °C) of the ethylene/allyl acetate copolymer obtained in Table 4, entry 3.

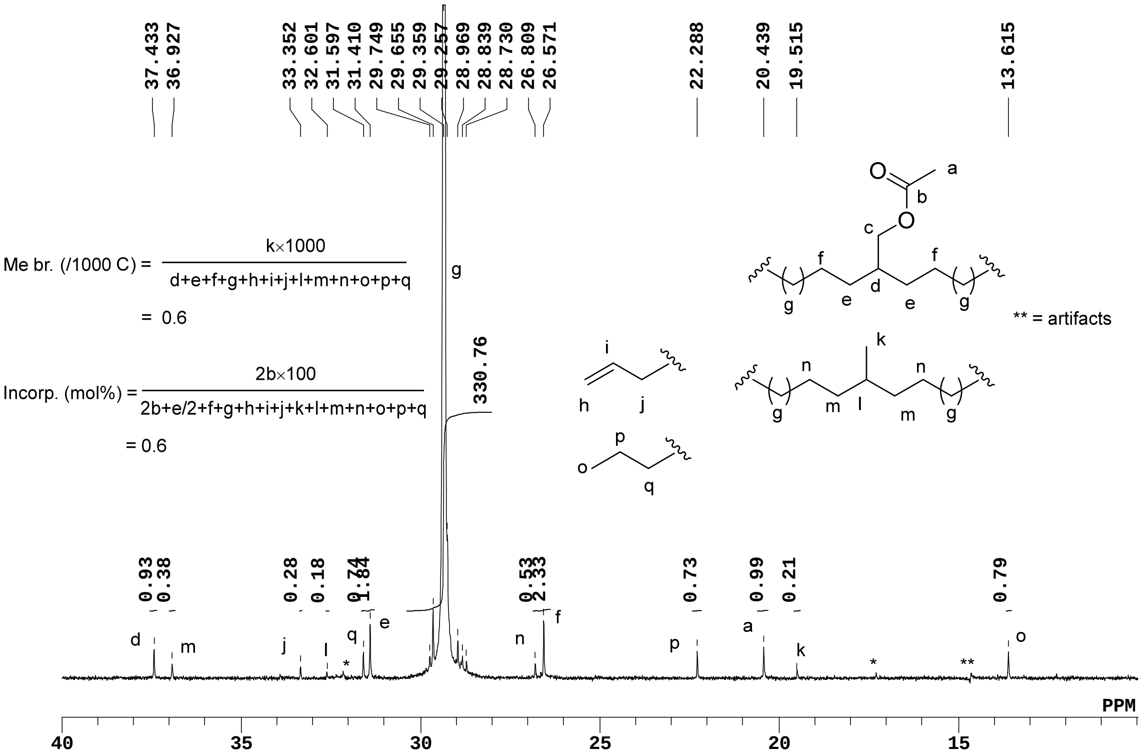

Figure S143. High field region of quantitative ^{13}C NMR spectrum (1,1,2,2-tetrachloroethane, 126 MHz, 120 °C) of the ethylene/allyl acetate copolymer obtained in Table 4, entry 3.

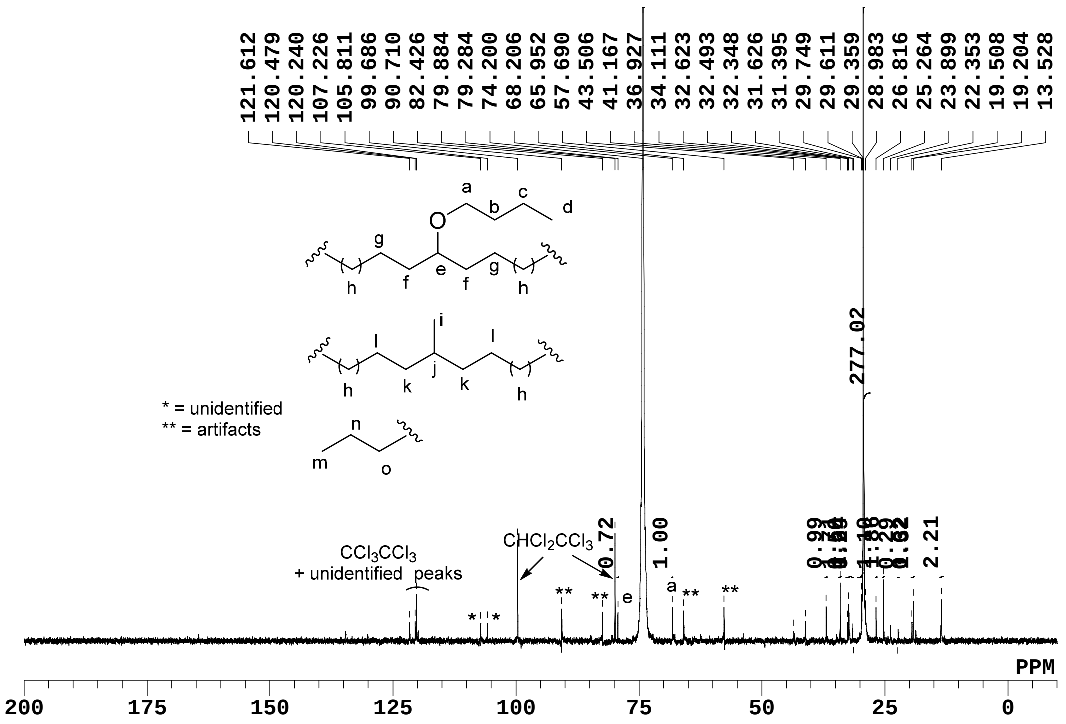

Figure S144. Quantitative ^{13}C NMR spectrum (1,1,2,2-tetrachloroethane, 126 MHz, 120 °C) of the ethylene/butyl vinyl ether copolymer obtained in Table 4, entry 4.

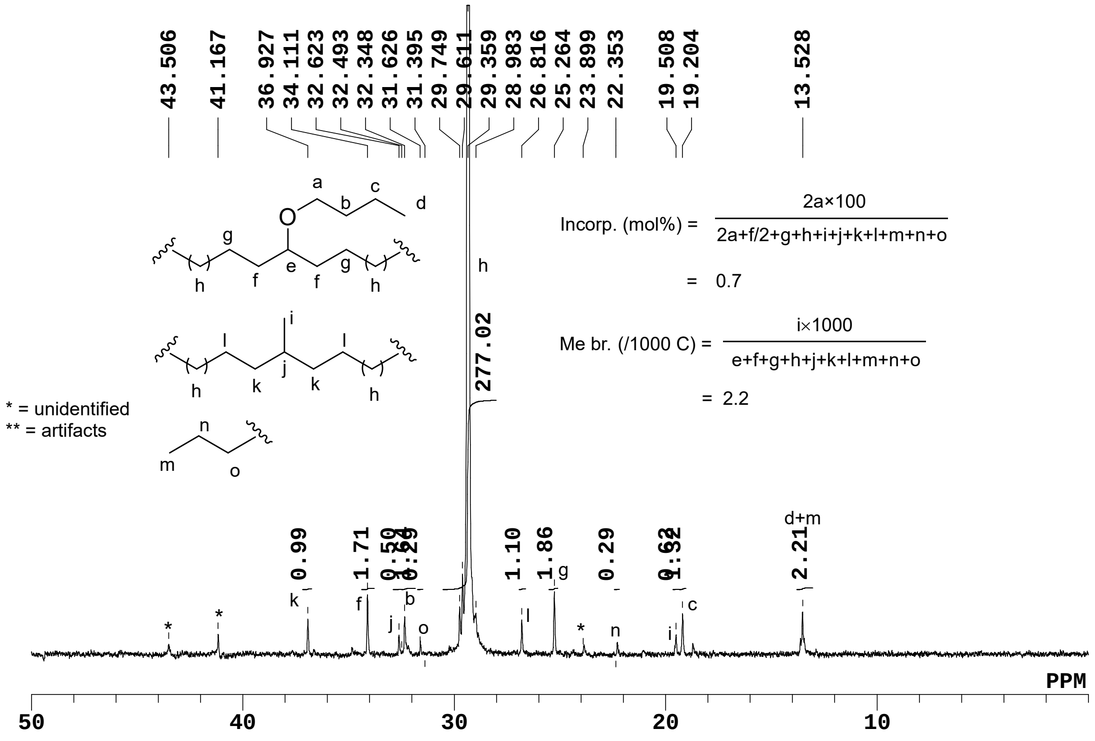

Figure S145. High field region of quantitative ^{13}C NMR spectrum (1,1,2,2-tetrachloroethane, 126 MHz, 120 °C) of the ethylene/butyl vinyl ether copolymer obtained in Table 4, entry 4.

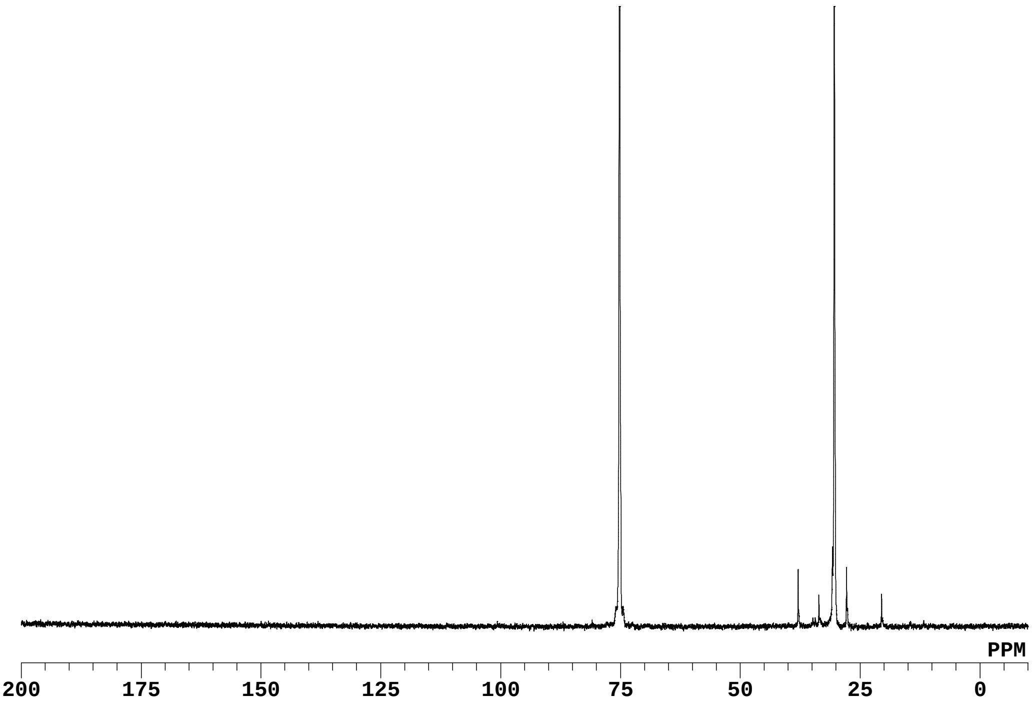

Figure S146. Quantitative ^{13}C NMR spectrum (1,1,2,2-tetrachloroethane, 101 MHz, 120 °C) of the polyethylene obtained in Table 4, entry 5.

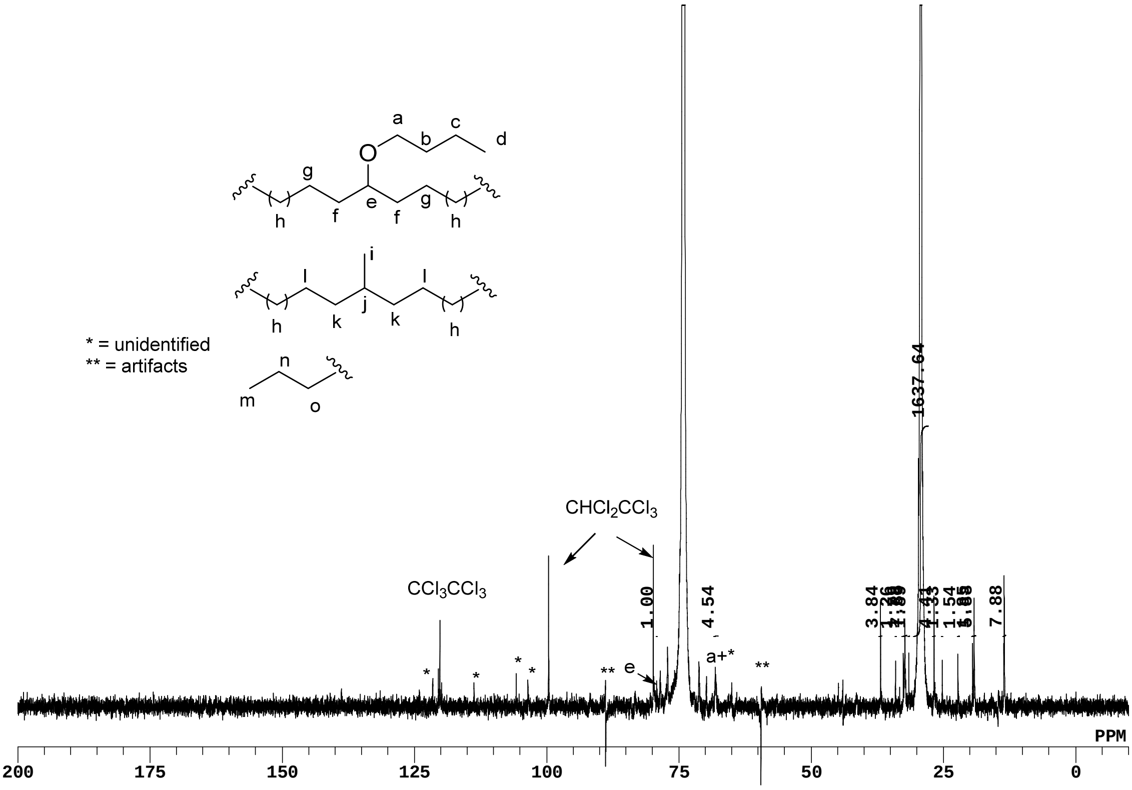

Figure S147. Quantitative ^{13}C NMR spectrum (1,1,2,2-tetrachloroethane, 126 MHz, 120 °C) of the ethylene/butyl vinyl ether copolymer obtained in Table 4, entry 6.

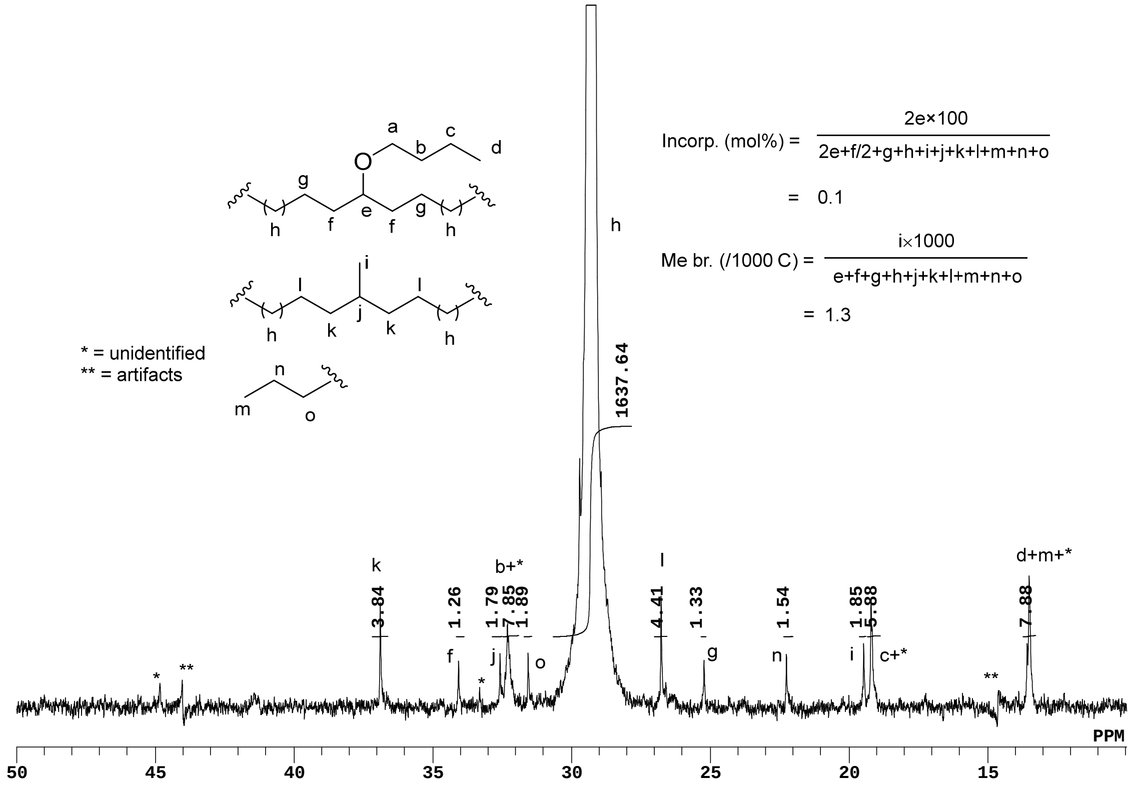

Figure S148. High field region of quantitative ^{13}C NMR spectrum (1,1,2,2-tetrachloroethane, 126 MHz, 120 °C) of the ethylene/butyl vinyl ether copolymer obtained in Table 4, entry 6.

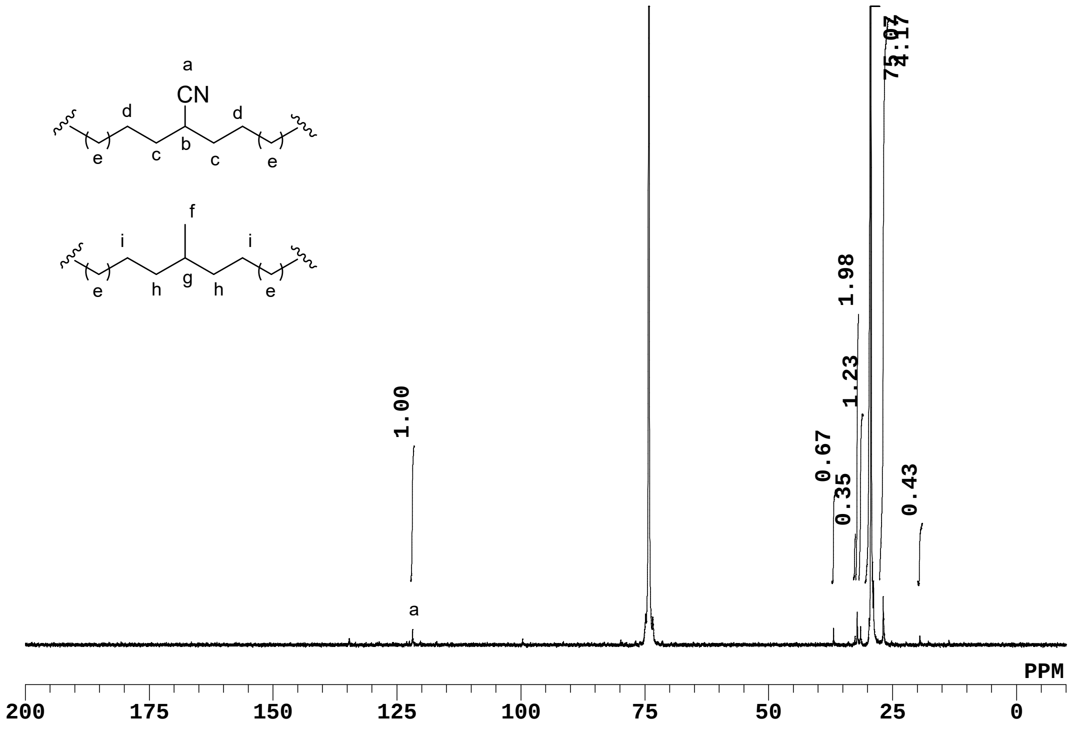

Figure S149. Quantitative ^{13}C NMR spectrum (1,1,2,2-tetrachloroethane, 101 MHz, 120 °C) of the ethylene/acrylonitrile copolymer obtained in Table 4, entry 7.

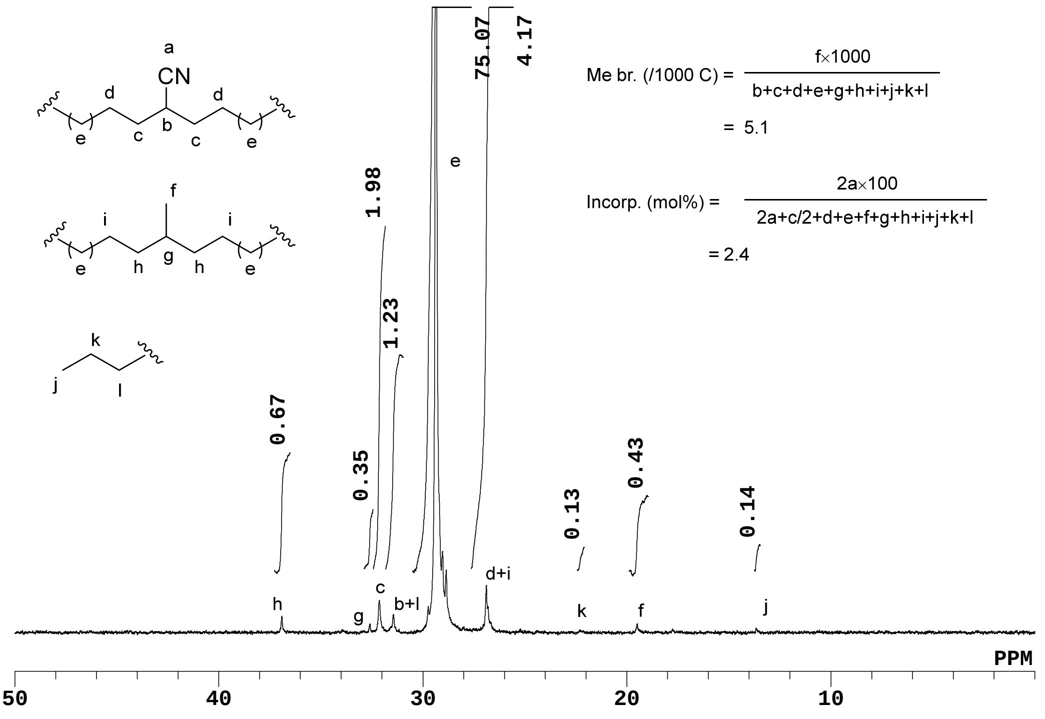

Figure S150. Quantitative ^{13}C NMR spectrum (1,1,2,2-tetrachloroethane, 101 MHz, 120 °C) of the ethylene/acrylonitrile copolymer obtained in Table 4, entry 7.

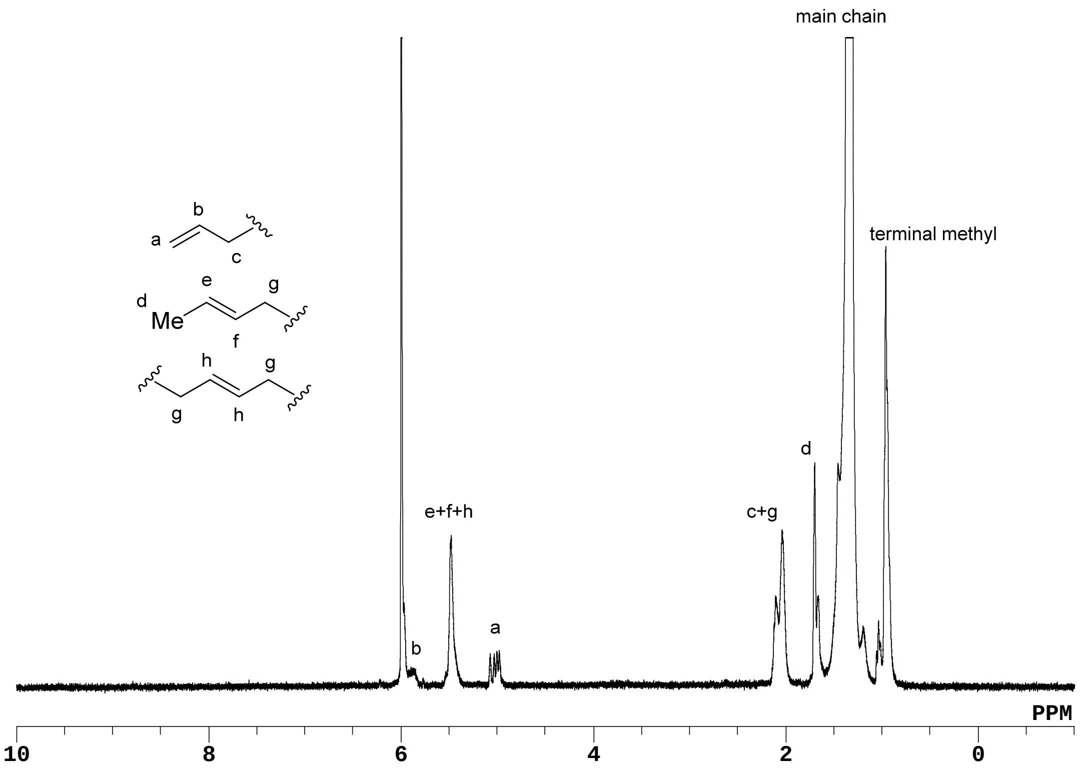

Figure S151. ^{1}H NMR spectrum ($C_2D_2Cl_4$, 400 MHz, 120 °C) of the polyethylene obtained in Table 4, entry 8.

6. Analysis of Reaction Mixture After Copolymerisation of Ethylene and MA by 1a

6-1. NMR Analysis

A mixture of **1a** (43.7 mg, 30 μmol), toluene (36 mL), and methyl acrylate (9.0 mL) was added into the autoclave. The autoclave was then sealed, charged with ethylene (3.0 MPa), and stirred in an isothermal heating block for 15 h at 80 °C. After cooling to room temperature, the volatile materials were removed *in vacuo* to afford solids (105.6 mg). A portion (10.2 mg) of the obtained solids, 1,4-bistrimethylsilylbenzene (4.8 mg, 22 μmol) and $C_2D_2Cl_4$ (0.60 mL) were added into NMR tube and then sealed (Figure S152).

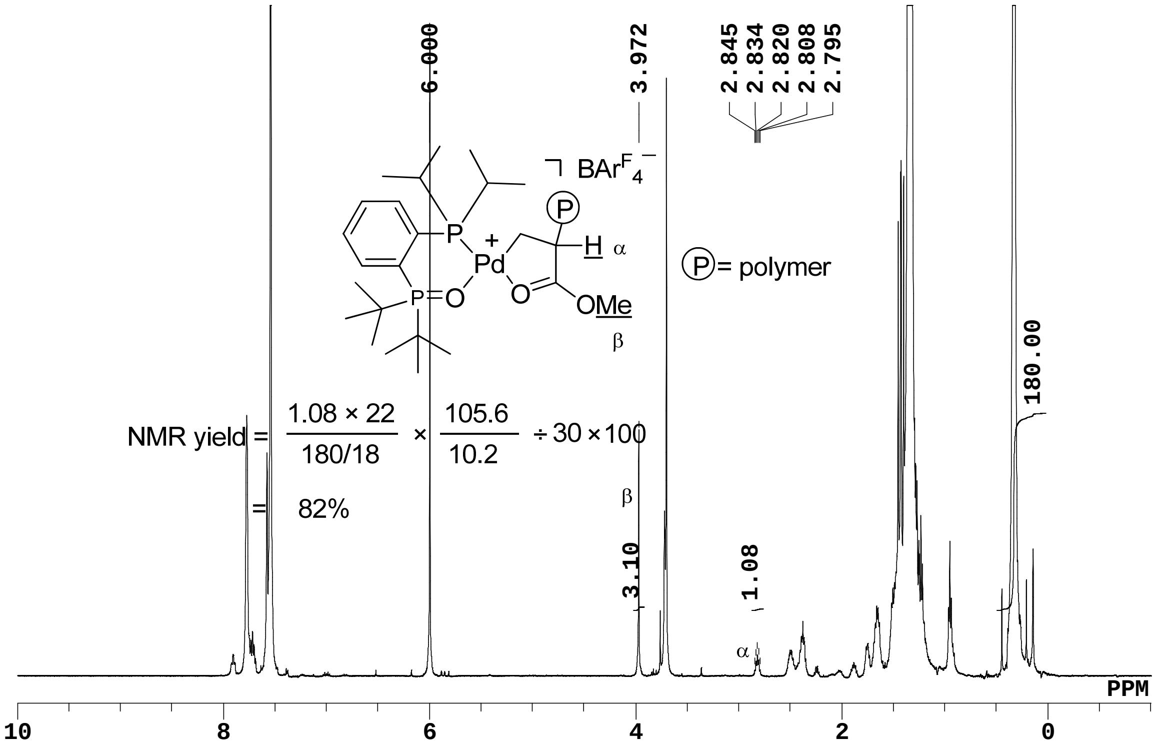

Figure S152. ^{1}H NMR spectrum ($C_2D_2Cl_4$, 500 MHz, 100 °C) of the residue after copolymerisation of ethylene with methyl acrylate. Signals at 2.82 ppm (1H, dddd, J = 6, 6, 6, 6 Hz, α) and 3.97 ppm (3H, s, β) were assigned to the 5-membered palladacycle. NMR yields were calculated based on the intensity of α-proton at 2.82 ppm using 1,4-bis(trimethylsilyl)benzene as an internal standard.

6-2. ESI-MS analysis

A mixture of **1a** (14.4 mg, 10 μmol), toluene (12 mL), and methyl acrylate (3.0 mL) was added into the autoclave. The autoclave was then sealed, charged with ethylene (3.0 MPa), and stirred in an isothermal heating block for 15 h at 80 °C. After cooling to room temperature, the reaction mixture (ca. 5.0 mL) was filtered through a syringe filter and the resulting solution was analysed by ESI-MS (Figure S153).

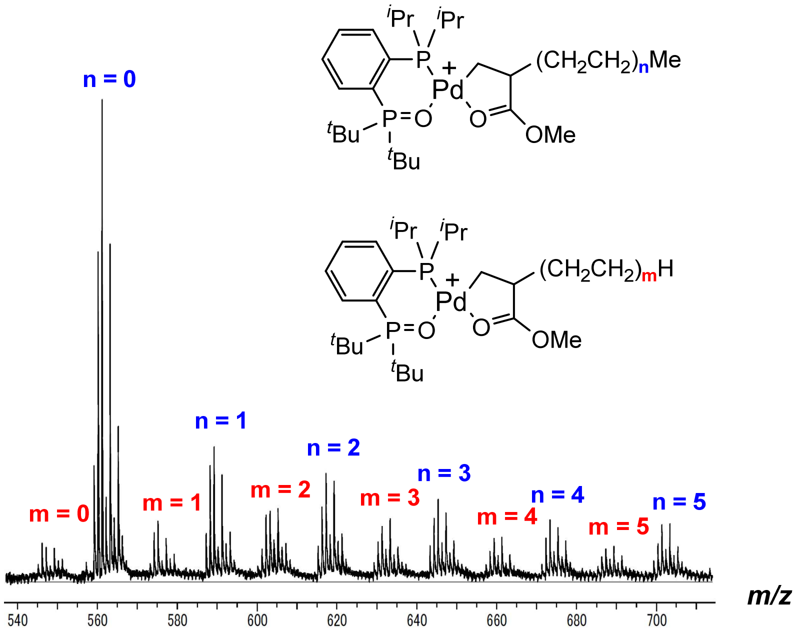

Figure S153. Mass spectrum (ESI-TOF) of the reaction mixture after copolymerisation. Signals up to n,m = 5 are shown.

Each signal corresponds to complexes formed by insertion of one methyl acrylate after several consecutive insertion of ethylene. We assigned these signals to 5-membered palladacycles formed by 1,2-insertion of methyl acrylate based on the experimental results that 5-membered palladacycle works as a kinetic trap during catalysis.

6-3. Gas Chromatography (GC) and GC-Mass Spectrometry (GC-MS) analysis

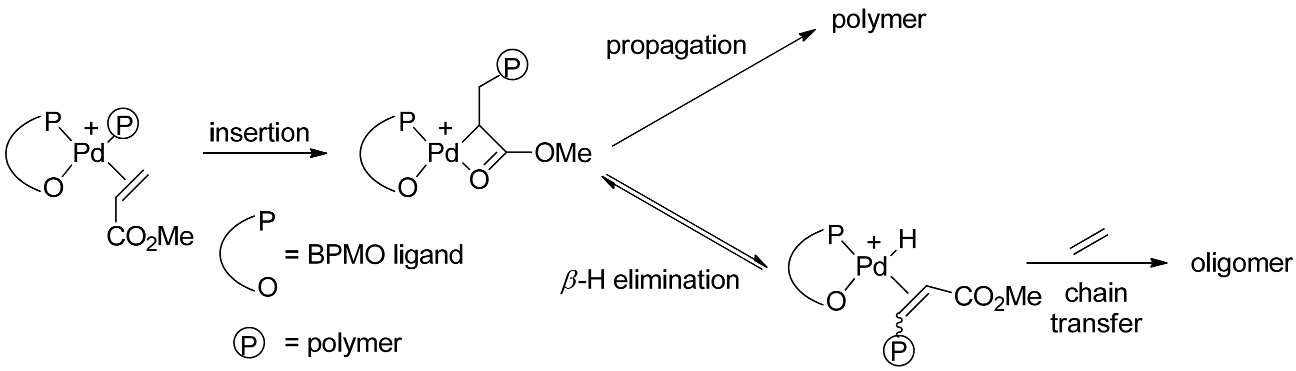

Scheme S1. β-H elimination and subsequent chain transfer after 2,1-insertion of methyl acrylate into the palladium-carbon bond.

It is possible that β-H elimination after insertion of methyl acrylate into the palladium–carbon bond and subsequent chain transfer prevent the chain propagation reaction (Scheme S1). If that is the case, a significant amount of oligomers should be detected in the reaction mixture after the

copolymerisation of ethylene with methyl acrylate. To confirm whether the process of methyl acrylate insertion/β-H elimination/chain transfer occurs or not, the reaction mixture after copolymerisation was analysed by GC and GC-MS.

A mixture of **1a** (14.6 mg, 10 μmol), toluene (12 mL), and methyl acrylate (3.0 mL) was added into the autoclave. The autoclave was then sealed, charged with ethylene (3.0 MPa), and stirred in an isothermal heating block for 15 h at 80 °C. Then, decane (30 μL) as an internal standard was added into the reaction mixture and the reaction mixture (ca. 1.5 mL) was filtered through a syringe filter packed with Florisil® to remove metals and insoluble materials. The resulting solution was analysed by GC and GC-MS without dilution (Figure S154). The GC and GC-MS analyses revealed that there was no detectable amount of oligomers in the reaction mixture after copolymerisation.

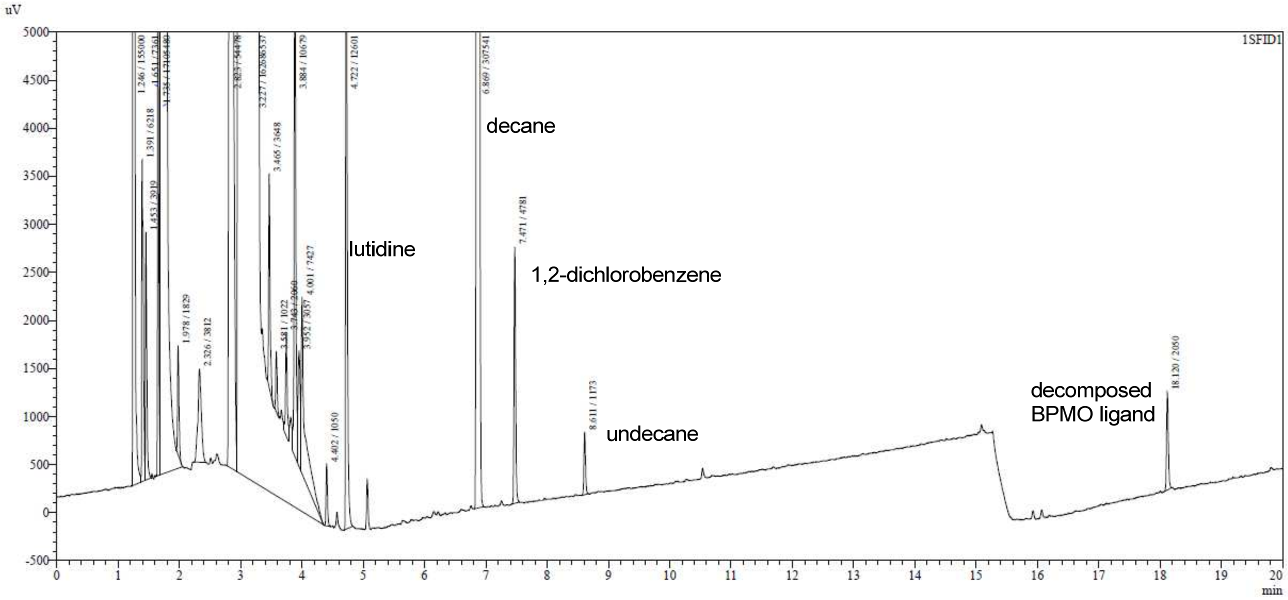

Figure S154. Gas chromatography analysis of reaction mixture after copolymerisation of ethylene with MA. Signals were assigned based on the results of GC-MS analysis.

Next, detection threshold of our GC for oligomers were investigated. Detection threshold of methyl 2-*trans*-octenoate, which can be formed during catalysis, was determined to be ca. 1.2 × 10^{-5} M. Thus, the amount of methyl 2-*trans*-octenoate in the reaction mixture (15 mL) should be less than 0.18 μmol (1.2 × 10^{-5} × 0.015). The amount of other oligomers in the reaction mixture could be roughly calculated by applying the concept of the effective carbon number (ECN)[x], which enables the estimation of relative response of compounds for flame ionisation detector and the results are summarised in Table S2. It was found that the amount of all oligomers in the reaction mixture should be less than 0.43 μmol. Considering that 10 μmol of catalyst was used for

[x] J. T. Scanlon, D. E. Willis, *J. Chromatogr. Sci.*, 1985, **23**, 333–340.

polymerisation, these amount of oligomers was negligibly small if oligomerisation takes place through the catalysis without the deactivation of the catalyst. Hence, we concluded that the process of methyl acrylate insertion/β-H elimination/chain transfer was not a major problem in our system.

Table S2. Predicted maximum amount of selected oligomers in the reaction mixture after copolymerisation.

compound	ECN[a]	amount[b] (μmol)
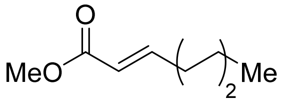	6.9	<0.18
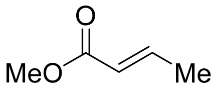	2.9	<0.43
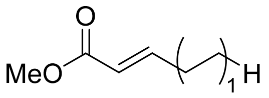	3.9	<0.32
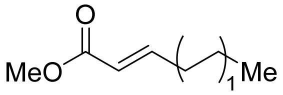	4.9	<0.25
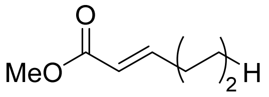	5.9	<0.21
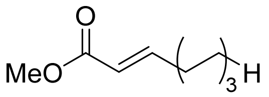	7.9	<0.16

[a]ECN was calculated based on following values: alkane = 1.0, olefin = 0.95, ester = –1.0
[b]Predicted maximum amount of oligomers in the reaction mixture. These values were calculated based on following equation: amount = 0.18×6.9/(ECN of target)

7. Discussion on Regioselectivity of MA Insertion

Table S3. %V_{bur} values of BPMO ligands.[xi]

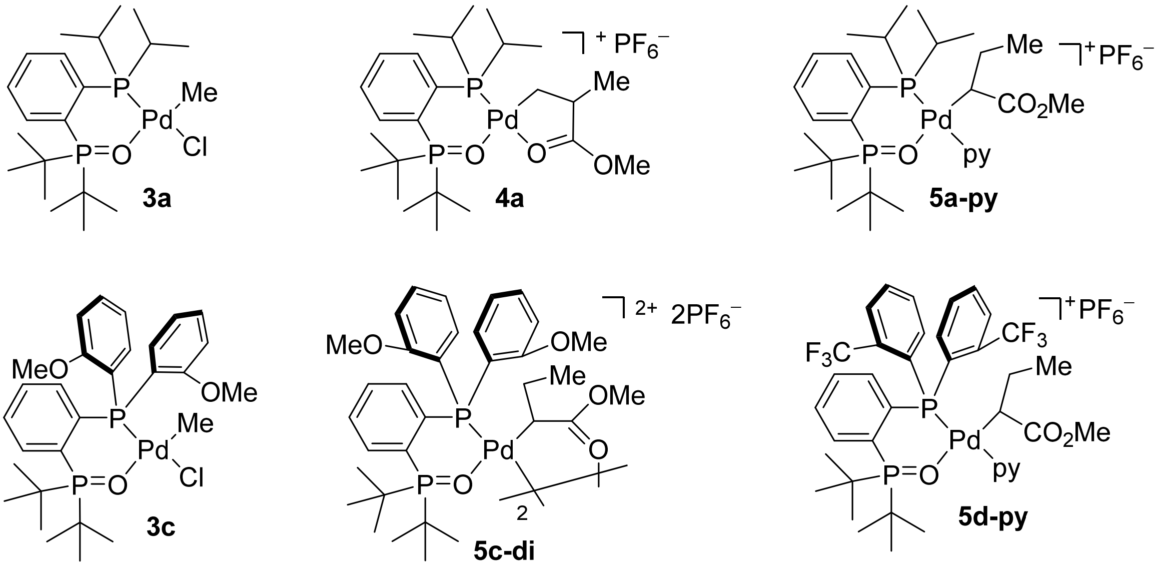

	3a	4a	5a-py	3c	5c-di	5d-py
%V_{bur}	49.2	51.7	51.1	47.8	51.9	53.2

In order to get insight into the reason why complex **3a** promoted 1,2-insertion of MA while complexes **3c** and **3d** did not, %V_{bur} values of BPMO ligands were calculated based on the X-ray structure of BPMO-Pd complexes (Table S3), since it was previously reported that regioselectivity of MA insertion was dependent on the steric bulkiness of ligands in the case of palladium complexes ligated by a phosphine–sulfonate.[xii,xiii] We found that direct comparison of %V_{bur} of **3a** and **3c** was difficult because the conformation of isopropyl group is flexible. In the X-ray structure of **3a**, both protons of isopropyl groups are oriented toward the phenylene backbone. In the X-ray structures of **4a** and **5a-py**, on the other hand, one proton is oriented toward the phenylene backbone and the other proton is oriented toward the opposite direction. In the solution state, steric bulkiness of diisopropylphosphino moiety should be explained by the average of the steric hindrance of both conformations. Thus, calculation of only one conformation of diisopropyl-phosphino moiety observed in the solid state can lead to inappropriately low or high %V_{bur} values.

[xi] For X-ray structure of **3a**, see ref. ix.
[xii] For %V_{bur} value, see (a) A. C. Hillier, W. J. Sommer, B. S. Yong, J. L. Petersen, L. Cavallo, S. P. Nolan, *Organometallics*, 2003, **22**, 4322–4326. (b) A. Poater, B. Cosenza, A. Correa, S. Giudice, F. Ragone, V. Scarano, L. Cavallo, *Eur. J. Inorg. Chem.*, 2009, 1759–1766.
[xiii] For regioselectivity of MA insertion, see (a) P. Wucher, L. Caporaso, P. Roesle, F. Ragone, L. Cavallo, S. Mecking, I. Göttker-Schnetmann, *Proc. Natl. Acad. Sci. U.S.A.*, 2011, **108**, 8955–8959. (b) P. Wucher, P. Roesle, L. Falivene, L. Cavallo, L. Caporaso, I. Göttker-Schnetmann, S. Mecking, *Organometallics*, 2012, **31**, 8505–8515.

Instead, we calculated the $\%V_{bur}$ values of all the complexes whose X-ray structures were obtained during our experiments and compared each $\%V_{bur}$ value. The $\%V_{bur}$ values of BPMO-ligand with diisopropylphosphino moiety were calculated based on the X-ray structure of **3a**, **4a**, and **5a-py**. These values are slightly dependent on the X-ray structure and average $\%V_{bur}$ value is 50.7. The $\%V_{bur}$ value of BPMO ligand in **3c** is 47.8 which is the lowest value among calculated $\%V_{bur}$ values. This result may explain the reason why 1,2-insertion of MA was not detected for **3c**, but the $\%V_{bur}$ value of the same BPMO ligand in **5c-di** is 51.9 and this value exceeds all the $\%V_{bur}$ values of ligand with diisopropylphosphino moiety. Average $\%V_{bur}$ value of ligand with di(2-methoxyphenyl)phosphino moiety is 49.9 and this value is similar to the average $\%V_{bur}$ value of BPMO ligand with diisopropylphosphino moiety. Thus, it seems that difference of steric bulkiness of BPMO ligands in **3a** and **3c** is not appropriate to explain the observed difference of MA insertion regioselectivitiy. We also calculated the $\%V_{bur}$ value of BPMO ligand in **5d-py** to be 53.2 and this is the highest value among the calculated six $\%V_{bur}$ values, which is also inconsistent with our experimental results. At the current stage, attempts to explain regioselectivity of MA insertion based on steric hindrance of BPMO ligands resulted in failure and the reason why complex **3a** promoted 1,2-insertion of MA while complexes **3c** and **3d** did not is unclear.

8. X-ray Crystallographic Analyses

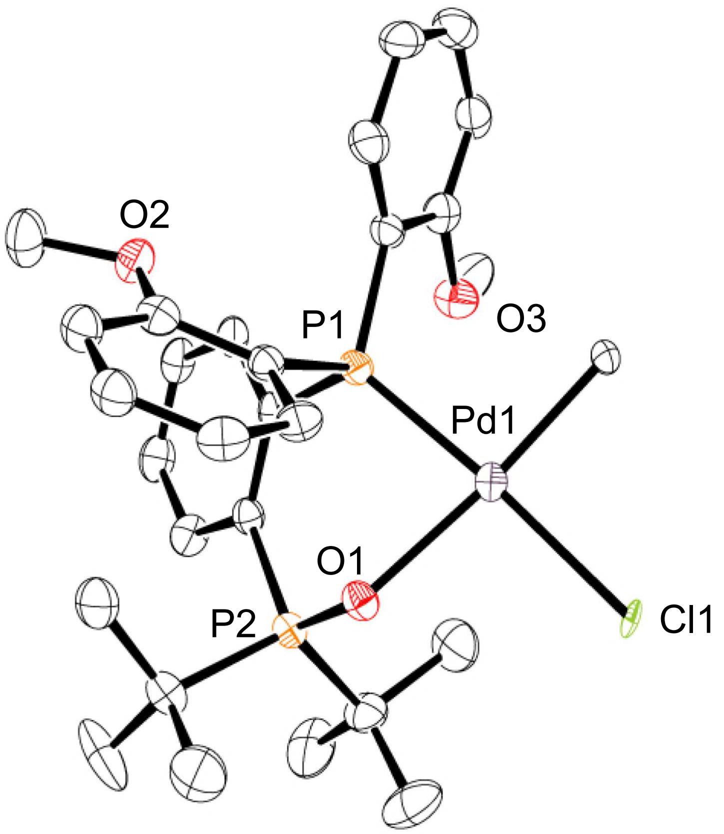

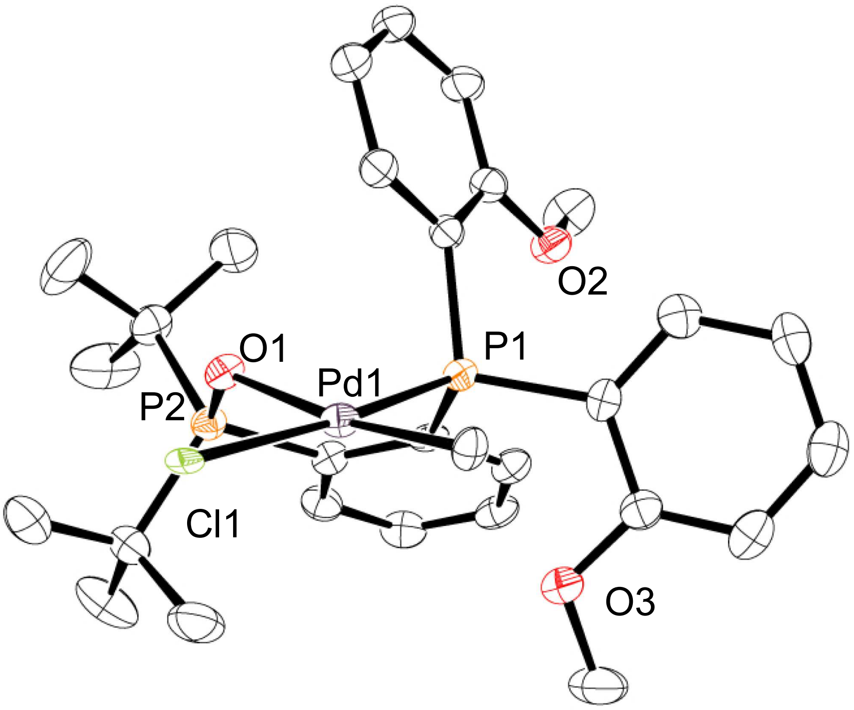

Figure S155. X-ray structure of **3c**. Thermal ellipsoids represent 50% probability. Hydrogen atoms and a hexafluorophosphate anion are omitted for clarity.

Table S4. Crystal data and structure refinement for **3c**.

CCDC number	1408864	
Empirical formula	$C_{29}H_{39}ClO_3P_2Pd$	
Formula weight	639.39	
Temperature	103 K	
Wavelength	0.71075 Å	
Crystal system	Orthorhombic	
Space group	$P2_12_12_1$	
Unit cell dimensions	a = 10.278(2) Å	α= 90°
	b = 15.524(4) Å	β= 90°
	c = 18.474(4) Å	γ = 90°
Volume	2947.8(11) $Å^3$	
Z	4	
Density (calculated)	1.441 Mg/m^3	
Absorption coefficient	0.857 mm^{-1}	
F(000)	1320	
Crystal size	0.400 × 0.350 × 0.250 mm^3	
Theta range for data collection	2.205 to 25.999°.	
Index ranges	–9<=h<=12, –19<=k<=19, –22<=l<=22	
Reflections collected	20838	
Independent reflections	5787 [R(int) = 0.0282]	
Completeness to theta = 25.242°	100.0 %	
Absorption correction	Semi-empirical from equivalents	
Max. and min. transmission	1.0000 and 0.8790	
Refinement method	Full-matrix least-squares on F^2	
Data / restraints / parameters	5787 / 0 / 334	
Goodness-of-fit on F^2	1.060	
Final R indices [I>2σ(I)]	R_1 = 0.0363, wR_2 = 0.1018	
R indices (all data)	R_1 = 0.0372, wR_2 = 0.1027	
Absolute structure parameter	0.114(14)	
Extinction coefficient	n/a	
Largest diff. peak and hole	1.827 and –0.694 e.$Å^{-3}$	

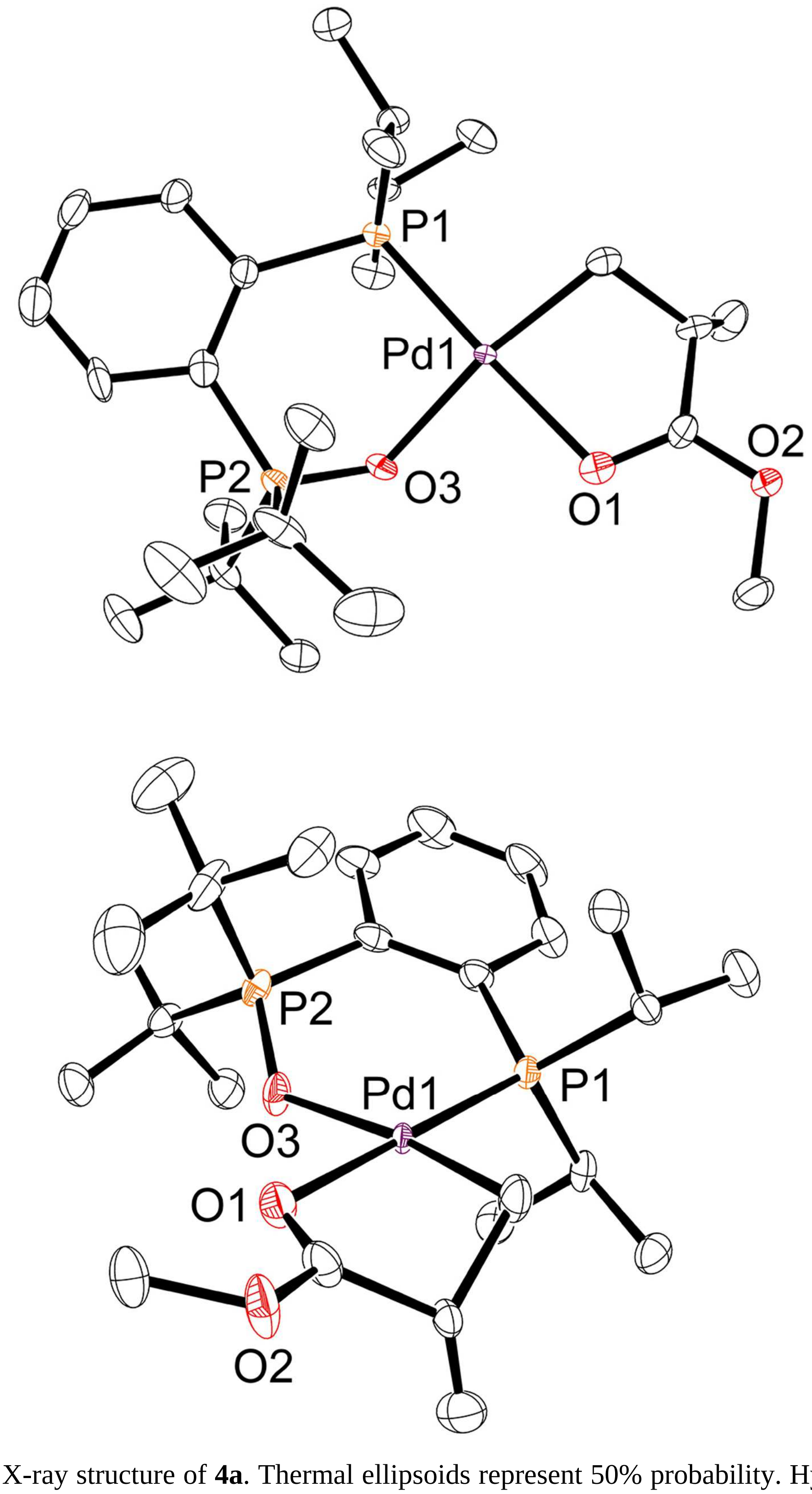

Figure S156. X-ray structure of **4a**. Thermal ellipsoids represent 50% probability. Hydrogen atoms and a hexafluorophosphate anion are omitted for clarity.

Table S5. Crystal data and structure refinement for **4a**.

CCDC number	1408865	
Empirical formula	$C_{25}H_{45}F_6O_3P_3Pd$	
Formula weight	706.92	
Temperature	93 K	
Wavelength	0.71075 Å	
Crystal system	Triclinic	
Space group	P-1	
Unit cell dimensions	a = 8.338(4) Å	α = 81.992(15)°
	b = 13.576(6) Å	β = 83.829(16)°
	c = 14.319(7) Å	γ = 75.466(16)°
Volume	1549.2(13) $Å^3$	
Z	2	
Density (calculated)	1.515 Mg/m^3	
Absorption coefficient	0.815 mm^{-1}	
F(000)	728	
Crystal size	0.060 × 0.060 × 0.010 mm^3	
Theta range for data collection	2.636 to 25.996°.	
Index ranges	–10<=h<=10, –16<=k<=16, –12<=l<=17	
Reflections collected	22424	
Independent reflections	5971 [R(int) = 0.0938]	
Completeness to theta = 25.242°	98.4 %	
Absorption correction	Semi-empirical from equivalents	
Max. and min. transmission	1.000 and 0.831	
Refinement method	Full-matrix least-squares on F^2	
Data / restraints / parameters	5971 / 342 / 430	
Goodness-of-fit on F^2	1.229	
Final R indices [I>2σ(I)]	R_1 = 0.0879, wR_2 = 0.1410	
R indices (all data)	R_1 = 0.0997, wR_2 = 0.1469	
Extinction coefficient	n/a	
Largest diff. peak and hole	1.083 and –1.451 e.$Å^{-3}$	

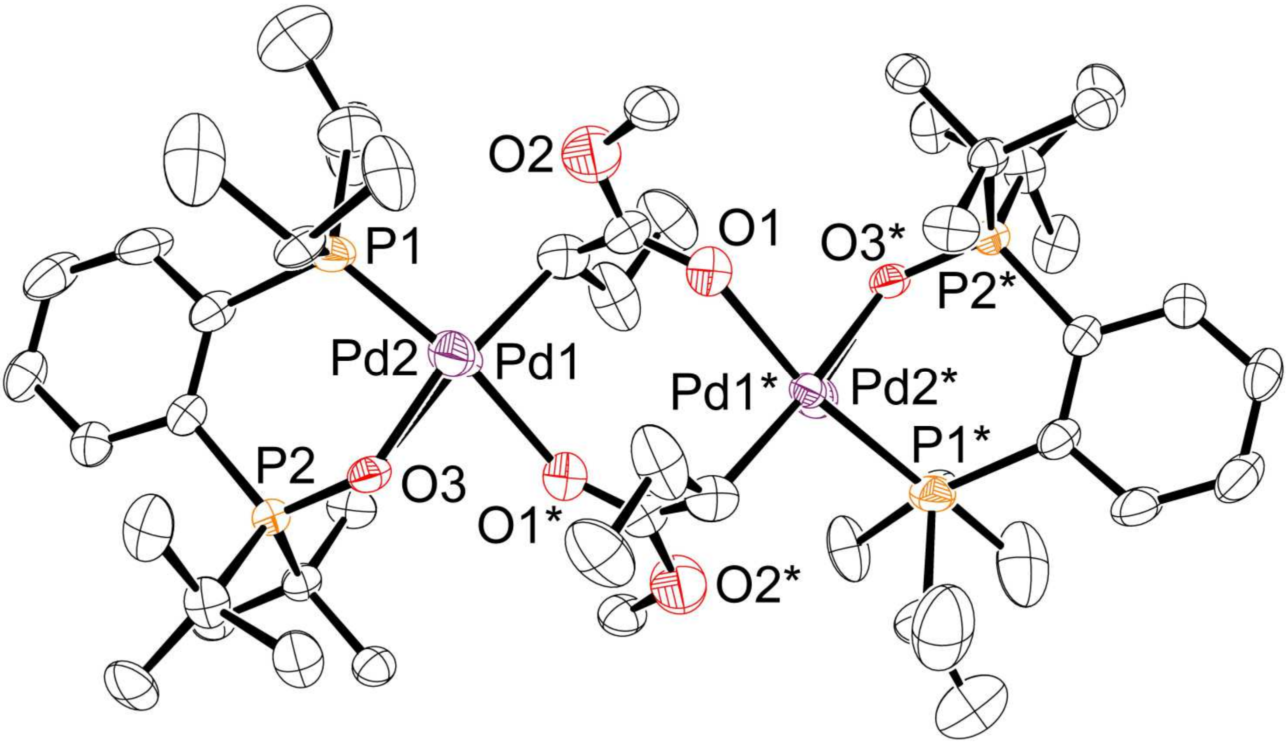

Figure S157. Preliminary X-ray structure of **5a-di**. The structure could not be fully solved due to heavy disorder of the 8-membered ring containing the palladium atoms. Hydrogen atoms and hexafluorophosphate anions are omitted for clarity.

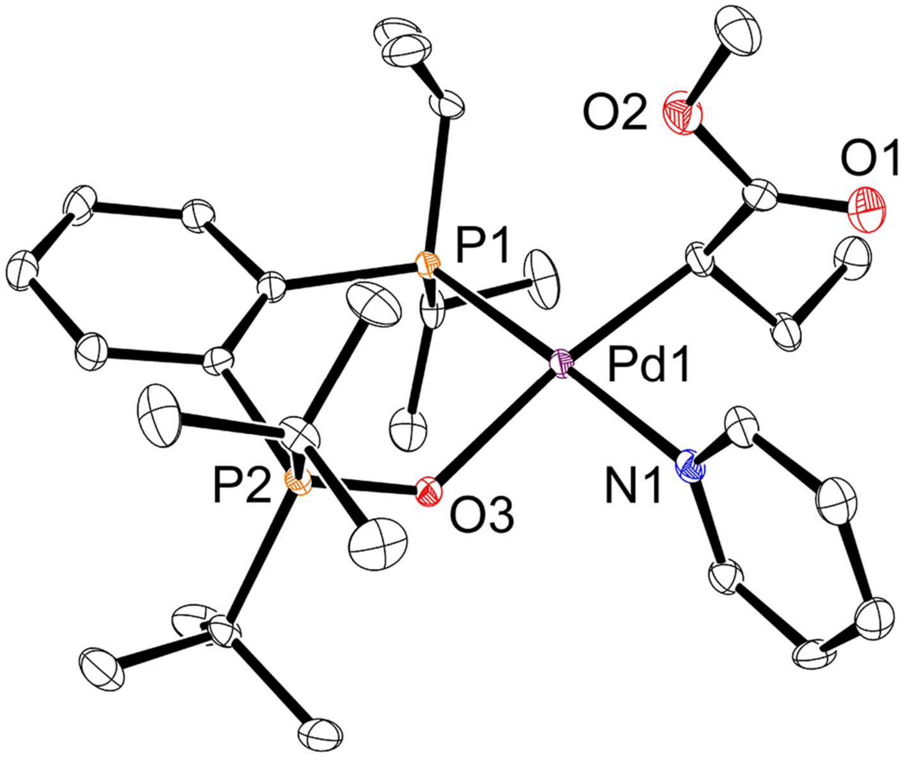

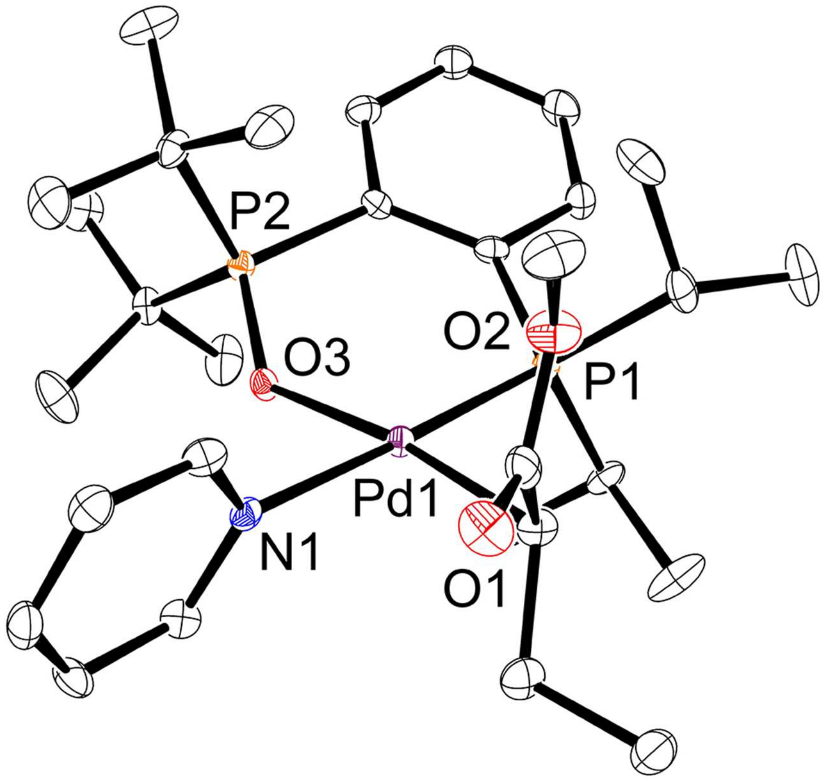

Figure S158. X-ray structure of **5a-py**·CH_2Cl_2. Thermal ellipsoids represent 50% probability. Hydrogen atoms, solvent, and a hexafluorophosphate anion are omitted for clarity.

Table S6. Crystal data and structure refinement for **5a-py**·CH_2Cl_2.

CCDC number	1408866	
Empirical formula	$C_{30}H_{49}F_6NO_3P_3Pd{\cdot}CH_2Cl_2$	
Formula weight	869.93	
Temperature	93 K	
Wavelength	0.71075 Å	
Crystal system	Monoclinic	
Space group	Cc	
Unit cell dimensions	a = 8.2128(13) Å	α = 90°
	b = 22.725(4) Å	β = 100.2994(19)°
	c = 20.504(4) Å	γ = 90°
Volume	3765.1(12) $Å^3$	
Z	4	
Density (calculated)	1.535 Mg/m^3	
Absorption coefficient	0.824 mm^{-1}	
F(000)	1788	
Crystal size	0.450 × 0.100 × 0.050 mm^3	
Theta range for data collection	3.069 to 25.998°.	
Index ranges	–8<=h<=10, –28<=k<=25, –25<=l<=25	
Reflections collected	13465	
Independent reflections	7013 [R(int) = 0.0262]	
Completeness to theta = 25.242°	99.8 %	
Absorption correction	Semi-empirical from equivalents	
Max. and min. transmission	1.000 and 0.898	
Refinement method	Full-matrix least-squares on F^2	
Data / restraints / parameters	7013 / 2 / 436	
Goodness-of-fit on F^2	1.032	
Final R indices [I>2σ(I)]	R_1 = 0.0256, wR_2 = 0.0569	
R indices (all data)	R_1 = 0.0269, wR_2 = 0.0581	
Absolute structure parameter	0.037(11)	
Extinction coefficient	n/a	
Largest diff. peak and hole	0.543 and –0.438 e.$Å^{-3}$	

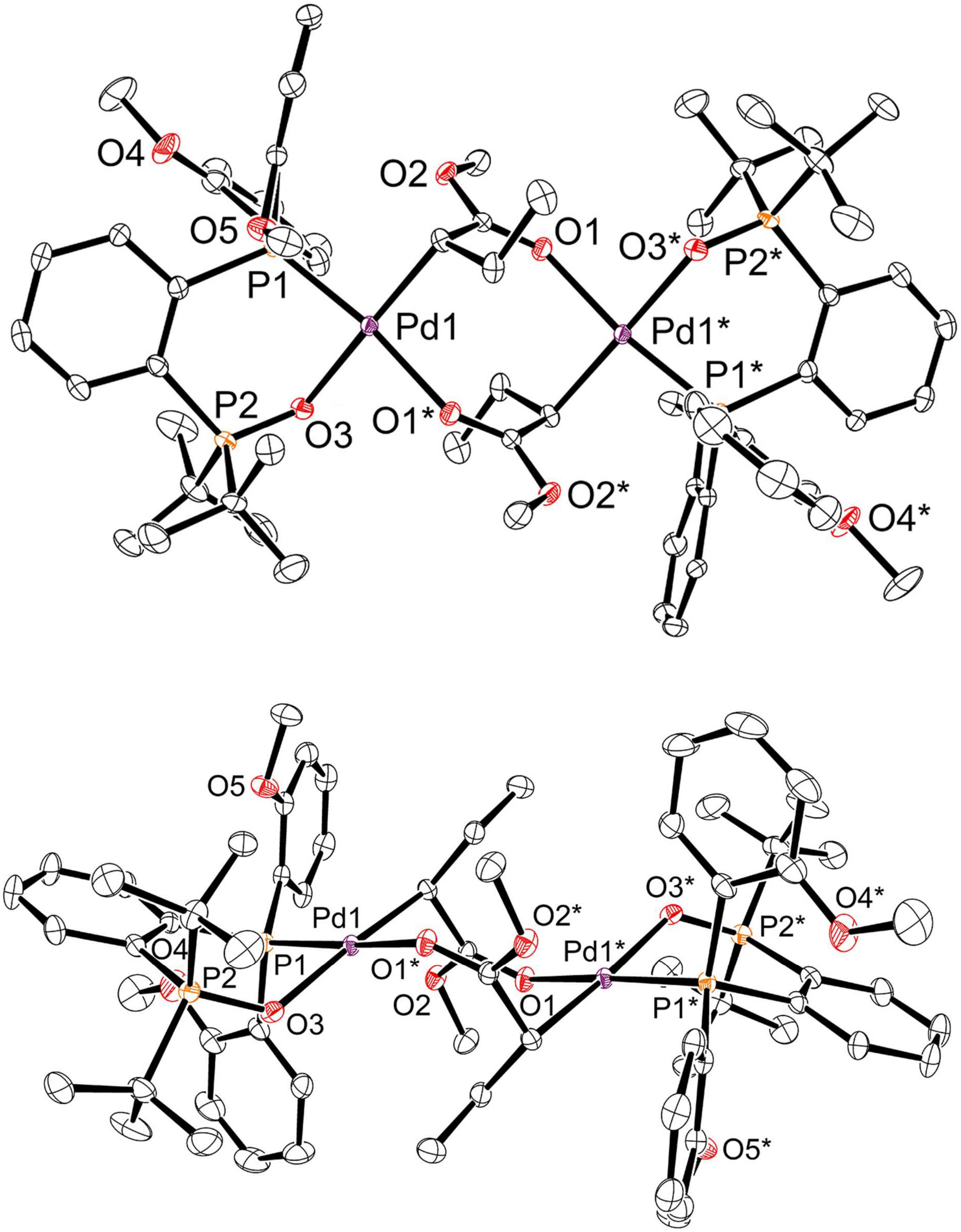

Figure S159. X-ray structure of **5c-di**·$CHCl_3$·$CHCl_2$. Thermal ellipsoids represent 50% probability. Hydrogen atoms, solvent, and a hexafluorophosphate anion are omitted for clarity.

Table S7. Crystal data and structure refinement for **5c-di**$\cdot CHCl_3 \cdot CHCl_2$.

CCDC number	1408867	
Empirical formula	$C_{33}H_{45}F_6O_5P_3Pd \cdot CHCl_3 \cdot CHCl_2$	
Formula weight	1038.28	
Temperature	93 K	
Wavelength	0.71075 Å	
Crystal system	Monoclinic	
Space group	$P2_1/c$	
Unit cell dimensions	a = 11.521(3) Å	α = 90°
	b = 16.475(4) Å	β = 99.836(4)°
	c = 22.564(6) Å	γ = 90°
Volume	4219.9(19) $Å^3$	
Z	4	
Density (calculated)	1.634 Mg/m^3	
Absorption coefficient	0.937 mm^{-1}	
F(000)	2108	
Crystal size	0.300 × 0.120 × 0.120 mm^3	
Theta range for data collection	2.473 to 25.997°	
Index ranges	–14<=h<=11, –20<=k<=20, –27<=l<=27	
Reflections collected	44666	
Independent reflections	8269 [R(int) = 0.0471]	
Completeness to theta = 25.242°	99.7 %	
Absorption correction	Semi-empirical from equivalents	
Max. and min. transmission	1.000 and 0.869	
Refinement method	Full-matrix least-squares on F^2	
Data / restraints / parameters	8269 / 6 / 516	
Goodness-of-fit on F^2	1.094	
Final R indices [I>2σ(I)]	R_1 = 0.0572, wR_2 = 0.1488	
R indices (all data)	R_1 = 0.0614, wR_2 = 0.1522	
Extinction coefficient	n/a	
Largest diff. peak and hole	0.711 and –2.736 e.$Å^{-3}$	

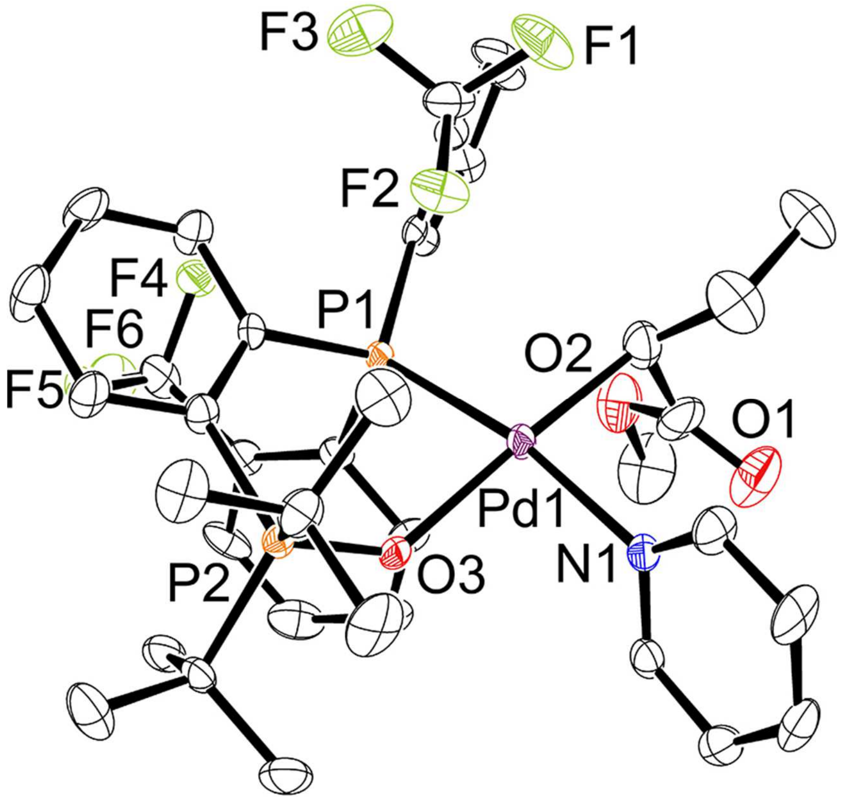

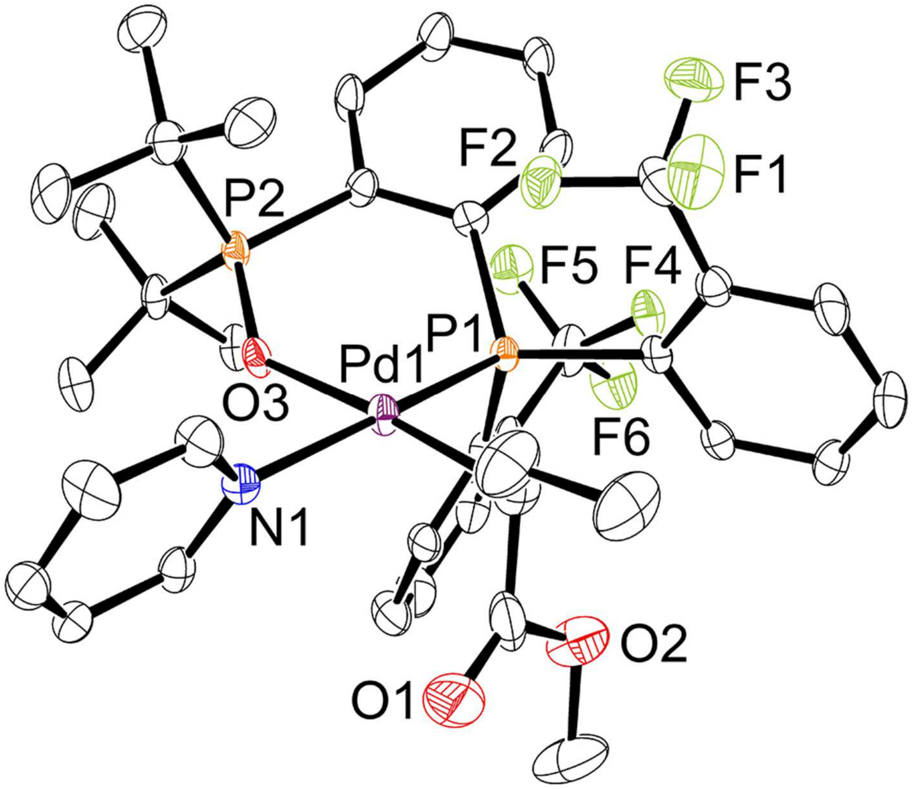

Figure S160. X-ray structure of **5d-py**. Thermal ellipsoids represent 50% probability. Hydrogen atoms and a hexafluorophosphate anion are omitted for clarity.

Table S8. Crystal structure and structure refinement for complex **5d-py**.

CCDC number	1408868	
Empirical formula	$C_{38}H_{44}F_{12}NO_3P_3Pd$	
Formula weight	990.05	
Temperature	93 K	
Wavelength	0.71075 Å	
Crystal system	Orthorhombic	
Space group	Pbca	
Unit cell dimensions	a = 14.361(3) Å	α = 90°
	b = 22.678(5) Å	β = 90°
	c = 25.532(6) Å	γ = 90°
Volume	8315(3) $Å^3$	
Z	8	
Density (calculated)	1.582 Mg/m^3	
Absorption coefficient	0.653 mm^{-1}	
F(000)	4016	
Crystal size	0.050 × 0.050 × 0.050 mm^3	
Theta range for data collection	2.135 to 26.000°	
Index ranges	–17<=h<=17, –27<=k<=26, –31<=l<=26	
Reflections collected	57538	
Independent reflections	8117 [R(int) = 0.0949]	
Completeness to theta = 25.242°	99.6 %	
Absorption correction	Semi-empirical from equivalents	
Max. and min. transmission	1.000 and 0.833	
Refinement method	Full-matrix least-squares on F^2	
Data / restraints / parameters	8117 / 115 / 590	
Goodness-of-fit on F^2	1.306	
Final R indices [I>2σ(I)]	R_1 = 0.0830, wR_2 = 0.1731	
R indices (all data)	R_1 = 0.0900, wR_2 = 0.1770	
Extinction coefficient	n/a	
Largest diff. peak and hole	1.281 and –0.807 e.$Å^{-3}$	
